# Influence of *exo*-Adamantyl Groups and *endo*-OH Functions on the Threading of Calix[6]arene Macrocycle

**DOI:** 10.1021/acs.joc.0c01769

**Published:** 2020-09-09

**Authors:** Veronica Iuliano, Carmen Talotta, Carmine Gaeta, Neal Hickey, Silvano Geremia, Ivan Vatsouro, Vladimir Kovalev, Placido Neri

**Affiliations:** † Laboratory of Supramolecular Chemistry, Department of Chemistry and Biology “A. Zambelli”, University of Salerno, Via Giovanni Paolo II 132, I-84084 Fisciano, Salerno, Italy; ‡ Centro di Eccellenza in Biocristallografia, Dipartimento di Scienze Chimiche e Farmaceutiche, Università di Trieste, Via L. Giorgieri 1, I-34127 Trieste, Italy; § Department of Chemistry, M. V. Lomonosov Moscow State University, Lenin’s Hills 1, 119991 Moscow, Russia

## Abstract

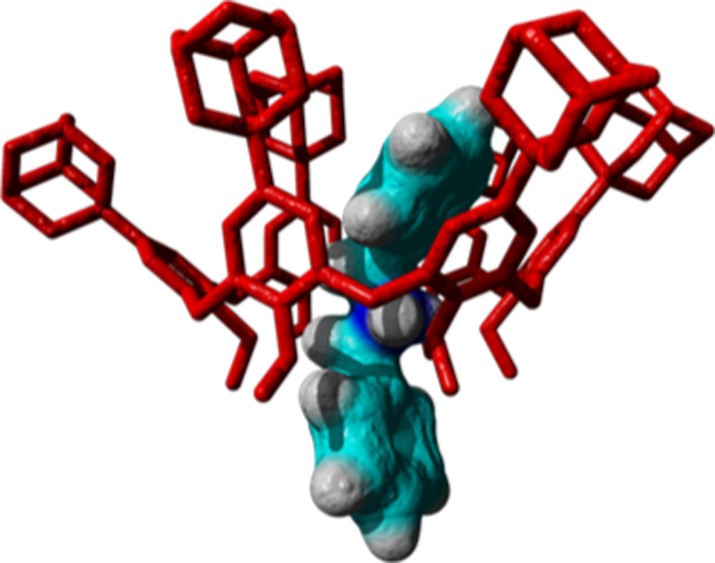

Calix[6]arenes bearing adamantyl groups at the *exo*-rim form pseudorotaxanes with dialkylammonium axles paired to the weakly coordinating [B(Ar^F^)_4_]^−^ anion. The *exo*-adamantyl groups give rise to a more efficient threading with respect to the *exo*-*tert*-butyl ones, leading to apparent association constants more than one order of magnitude higher. This improved stability has been ascribed to the more favorable van der Waals interactions of *exo*-adamantyls versus *exo*-*tert*-butyls with the cationic axle. Calix[6]arenes bearing *endo*-OH functions give rise to a less efficient threading with respect to the *endo*-OR ones, in line with what was known from the complexation of alkali metal cations.

## Introduction

**Figure 1:**
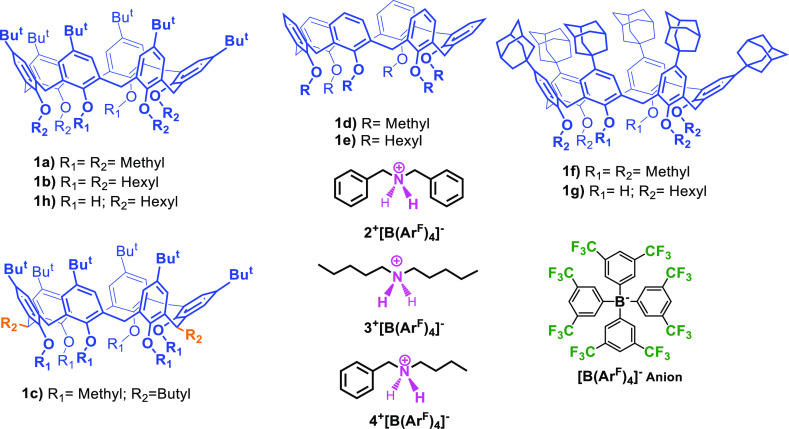
Chemical drawing of calix[6]arene wheels **1a–h**, ammonium cations **2**^**+**^**–4**^**+**^ and [B(Ar^F^)_4_]^−^ anion.

Mechanomolecules,^1a^ such as rotaxanes and catenanes, have become increasingly popular thanks to their aesthetical appeal and to their applications as molecular machines^1b^ or catalysts.^1c^ They are most frequently obtained by threading a rod-like guest (axle) inside a macrocyclic host molecule (wheel) to give an interpenetrated pseudorotaxane precursor.^1d^ Beginning with crown ethers,^2^ a series of macrocyclic classes has been used over the years as the wheel component, which includes cyclodextrins,^3^ cucurbiturils,^4^ macrolactams,^5^ calixarenes,^6^ and pillararenes.^7^ As concerns the calixarene threading, it has been actively investigated by us^8^ and by Arduini and co-workers^9^ mainly using dialkylammonium and viologen axles, respectively. In particular, 10 years ago we found that scarcely preorganized calix[6]arene ethers (e.g.: **1a,b**) can be threaded by dialkylammonium axles only when they are coupled to the weakly coordinating tetrakis[3,5-bis(trifluoromethyl)phenyl]borate ([B(Ar^F^)_4_]^−^) (Figure 1) “superweak” anion.^10^ During our studies, we have also found that the conformational mobility of the calix-wheel is strongly influencing the efficiency of the threading. In fact, the more preorganized hexahexyloxycalix[6]-wheel **1b** is threaded more efficiently than the more mobile hexamethoxy-**1a** analogue, by dialkylammonium axles **2**^**+**^**–4**^**+**^.^10c^ In accordance, we have recently^11^ evidenced that the presence of alkyl substituents at the methylene bridges (e.g., **1c**) also increases the threading efficiency as a result of the increased degree of preorganization. Another parameter strongly affecting the calix[6]arene threading is the nature of the substituents present at the exo-rim (commonly also called as the upper rim). Thus, the very common *p*-*tert*-butyl groups (like in **1a** or **1b**) give rise to more stable pseudorotaxane complexes than their simpler *p*-H-counterparts (**1d,e**), probably because of more extended favorable van der Waals interactions with the cationic axle.^10a,12^ On the basis of this knowledge, we were intrigued to know the effect of bigger and more encumbering groups at the exo-rim, such as the *p*-adamantyl ones of **1f**,**g**, on the threading efficiency. In addition, we wonder whether the substitution of a few of the OR groups at the endo-rim with the OH ones could also lead to thermodynamically stable pseudorotaxane complexes. Prompted by these questions, we have investigated the threading ability by dialkylammonium axles of some *p*-adamantylcalix[6]arene ethers, including some examples of derivatives bearing free OH functions at the endo-rim, and we report here the result of this study.

## Result and Discussion

### Synthesis and Conformational Properties of the Studied Hosts

The studied hosts were easily obtained by exploiting the classical procedures reported in the literature for the synthesis of calix[6]arene derivatives.^13−15^ In particular, *p*-adamantylcalix[6]arene hexamethyl ether **1f** was obtained in 47% yield by methylation of the parent *p*-adamantylcalix[6]arene-hexol^13^ with MeI, promoted by NaH. Its characterization was made easy by the sharp appearance of its ^1^H and ^13^C NMR signals because of the high conformational mobility of the macrocycle.

**Figure 2:**
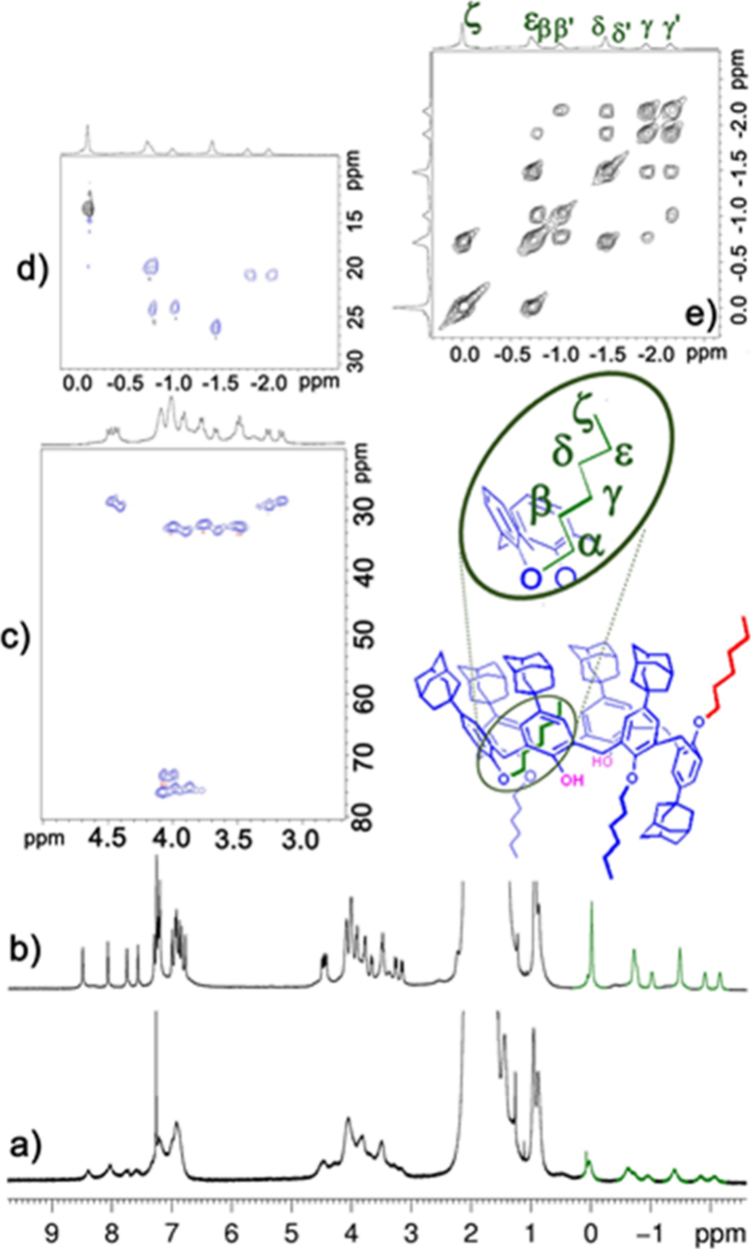
^1^H NMR spectra (CDCl_3_, 600 MHz) of pseudo[1]rotaxane derivative **1g** at 298 (a) and 243 K (b); (c,d) different portions of the HSQC spectrum of **1g** (CDCl_3_, 600 MHz, 243 K); (e) portion of the COSY-45 spectrum of **1g** (CDCl_3_, 600 MHz, 243 K).

In particular, a single set of singlets was observed (Figure 5a) for the six equivalent ArCH_2_ moieties (e.g., at 3.91 and 2.92 ppm for ArCH_2_Ar and OCH_3_ groups, respectively). The structure of **1f** was also confirmed by X-ray crystallography which also provided useful information about its preferred conformation (vide infra). The 1,2,4,5-tetrahexyloxy-*p*-adamantyl-calix[6]arene-diol derivative **1g** was obtained in 33% yield by an extension of the NaH-promoted 1,2,4,5-tetrasubstitution of calix[6]arenes originally reported by Gutsche.^14^ The characterization of **1g** was made less easy by the broad appearance of its ^1^H and ^13^C NMR signals at room temperature (Figure 2a), due to a conformational mobility close to the NMR time scale. Therefore, all the relevant information was acquired at a high temperature (373 K) in CDCl_2_CDCl_2_ (TCDE) (Supporting Information). It is worth to noting here that at the room temperature, the ^1^H NMR spectrum of **1g** in CDCl_3_ (Figure 2a) shows unusual signals in the negative region of the spectrum, which could be ascribed to one of the hexyl chains self-included inside the calix[6]-cavity (see chemical drawing of **1g** in Figure 2). Of course, this could be only possible if a peculiar conformation is assumed by **1g** in solution. Thanks to NMR characterization at 243 K (Figures 2b–e and S9–S11), it was possible to assign a partial-cone conformation to **1g** with one of the four hexyl chains self-included into the cavity to give a pseudo[1]rotaxane structure. Also in this case, conclusive proof of the structure and a confirmation of the peculiar conformation adopted by **1g** was obtained by X-ray crystallography (vide infra).

**Scheme 1:**
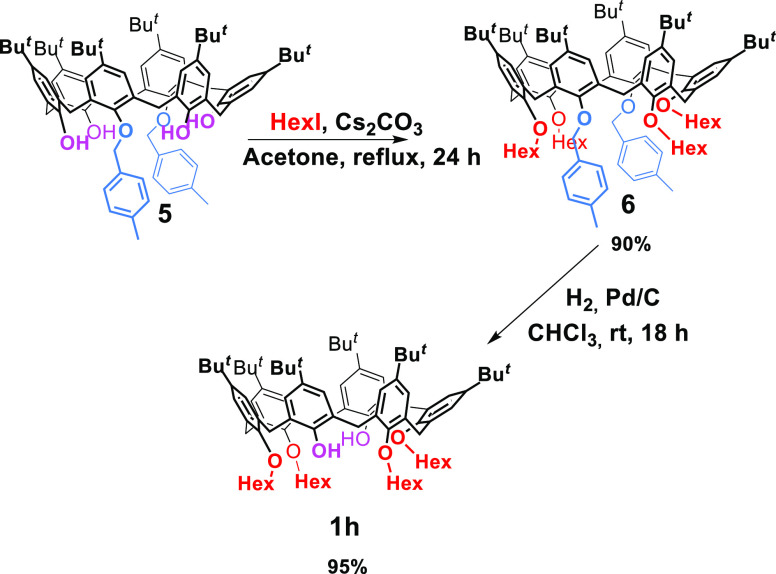
Synthesis of Derivative **1h**

1,2,4,5-Tetrahexyloxy-*p*-*tert*-butylcalix[6]arene-diol derivative **1h** was obtained by a protection–deprotection procedure (Scheme 1) starting from the known 1,4-dibenzyloxy-*p*-*tert*-butylcalix[6]arene-tetra-ol **5**,^15^ which was first tetralkylated with hexyl iodide (compound **6**; 90% yield) and then debenzylated with H_2_/Pd/C (95% yield).

The characterization of **1h** was very similar to that of **1g** for what concerns the broad appearance of its ^1^H and ^13^C NMR signals at room temperature, due to a conformational mobility close to the NMR time scale. The similarity was also extended to the unusual signals in the negative region of the spectrum because of the self-included hexyl chain. Also, in this case a characterization at low temperatures was performed that confirmed the peculiar pseudo[1]rotaxane partial-cone conformation (Figures S20–S22). This result leads to suppose that this conformational feature could be a characteristic of calix[6]arenes 1,2,4,5-tetrasubstituted with long alkyl chains.^16^

### X-ray Analysis of **1f** and **1g**

**Figure 3:**
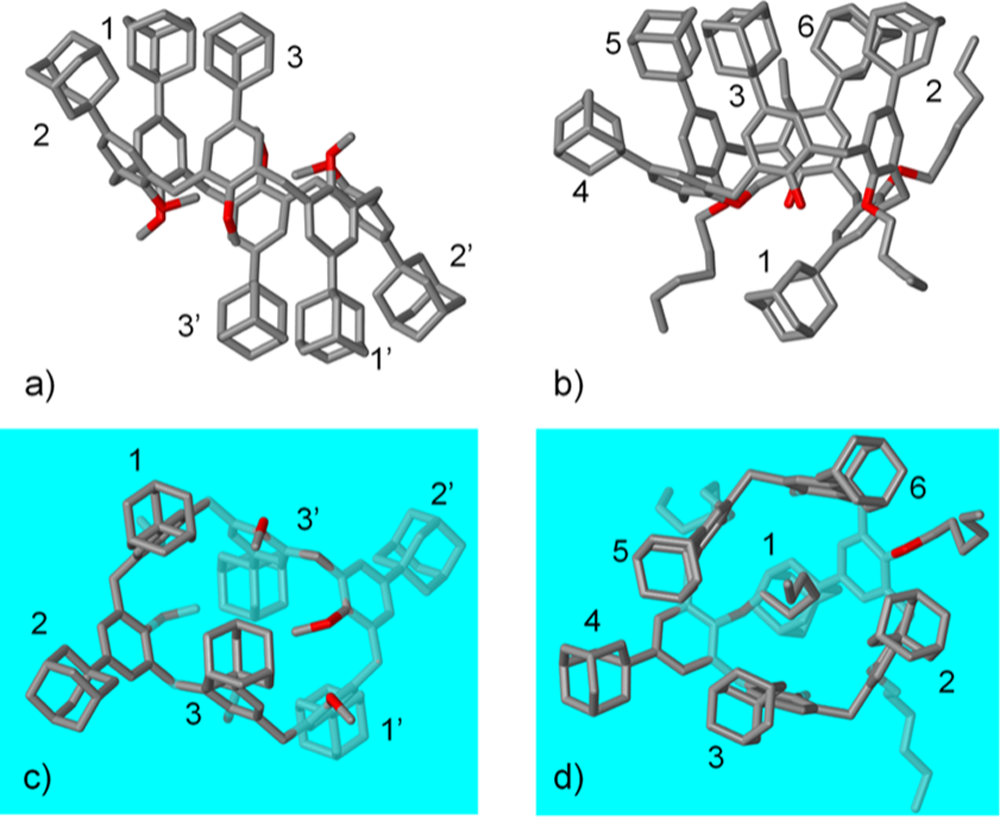
(a,c) Side and top views of the X-ray structure of **1f**. (b,d) Side and top views of the X-ray structure of **1g**. In blue, the ArCH_2_Ar mean plane. Hydrogen atoms, solvent, and disordered groups are omitted for clarity.

Small colorless single crystals of **1f** and **1g** suitable for X-ray structure determination were analyzed using synchrotron radiation and cryo-cooling techniques. Both molecules crystallized in the centrosymmetric triclinic space group. In the solid state, **1f** exhibits a centrosymmetric 1,2,3-alternate conformation (Figure 3a). The asymmetric unit consists of a 1/2 molecule of **1f**, which lies on an inversion center, and two CHCl_3_ solvent molecules outside of the macrocycle. **1f** exhibits a molecular *C*_*i*_ point symmetry.

The conformation of the adamantyl-substituted aryl rings is illustrated in Figure 3, where the molecules are viewed orthogonally (Figure 3c,d) with respect to the mean plane (in blue in Figure 3) of the calix[6]arene, as defined by the six methylene bridges. An absolute angle value greater/smaller than 90° indicates the outward/inward orientation of the adamantly group, while a negative sign indicates an inverted orientation of the adamantyl group with respect to a given orientation of the macrocycle. In the case of **1f**, the mean plane of one of the aryl rings (1) (Figure 3a), is almost perpendicular to the mean plane of the calix[6]arene, with a dihedral angle of 96°(Figure 3c). The adamantyl group thus leans slightly outward from the center of the macrocycle. The mean planes of the other two aryl rings make a large outward dihedral angle (2, 132°) and a slight inward angle (3, 72°), consequently, these adamantyl groups lean outward and inward, respectively. The symmetry of the molecule implies that the other three opposite phenyl groups of the macrocycle with an inverted orientation (1′, 2′, and 3′) have identical angles in the absolute value but with a negative sign. The methoxy groups are inward oriented for 2 (2′) and 3 (3′) and outward oriented for 1 (1′) (Figure 3c). The overall conformation, with two bulky adamantyl groups and four methoxy groups tilted toward the center of the macrocycle, results in a sealed molecular cavity (Figure 3a,c).

In the solid state, **1g** exhibits an asymmetric partial-cone conformation, with just one of the phenyl rings with an inverted orientation with respect to the other five (Figure 3b). This phenyl (1) is opposite to a phenyl group (4) bearing a hexyloxy chain self-included into the macrocycle. The partial-cone conformation, combined with the mixed hydroxy/hexyloxy substitution pattern at the lower rim (1,2,4,5-hexyloxy) results in an asymmetric *C*_1_ molecular point symmetry. As it crystallized in the *P*1̅ space group, the structure is therefore composed of a racemic mixture of inherently chiral **1g** molecules.

With regard to the overall conformation of **1g** (Figure 3d), for the purposes of the following discussion, the side with the five adamantyl substituents is defined as the upper side. The mean plane of the inverted phenyl ring, with the adamantyl group on the lower side (1), is acutely tilted inward with a dihedral angle of −47° with respect to the mean plane defined by the six methylene bridges. The mean plane of the phenyl ring (4), located directly opposite the inverted ring, makes a very large outward dihedral angle (158°) on the upper side. Consequently, the bulk adamantyl group is tilted far from the center of the macrocycle, while the hexyl chain occupies the cavity of the macroring.

The internal hexyl chain is disordered over two positions with equal occupancy factors. In the first conformer, the two central bonds both assume *gauche*^+^ conformations (*gauche*^–^ for the second conformer); while the other two relevant C–C bonds both assume an anti conformation (in both conformers). The mean planes of the other four phenyl groups 2 (hexyloxy-substituted), 3 (hydroxy-substituted), 5 (hexyloxy-substituted), and 6 (hydroxy-substituted) are all close to orthogonal with respect to the above-defined calix[6]arene mean plane; however, in all cases the adamantyl groups are tilted slightly outward, with angles of 94, 98, 96, and 98°, respectively. The conformation of these four phenyl groups is influenced by the formation of hydrogen bonds between the hydroxy group donors (3, 6) and the adjacent hexyloxy oxygen acceptor (2, 5) with O···O distances of 2.78 and 2.86 Å.

### Influence of *exo*-Adamantyl Groups on Calixarene Threading

**Figure 4:**
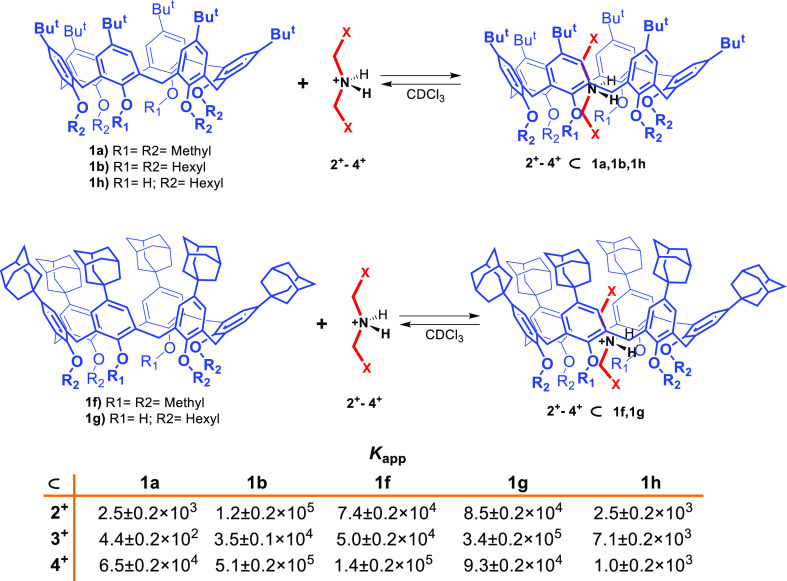
Threading of calix[6]-wheels and *K*_app_ values (M^–1^) measured for the formation of the corresponding pseudorotaxanes.

Initially, the influence of the *exo*-adamantyl groups was investigated by studying the threading abilities of *p*-adamantylcalix[6]arene hexamethyl ether **1f** by dibenzylammonium axle **2**^**+**^·[B(Ar^F^)_4_]^−^ (Figure 4).

**Figure 5:**
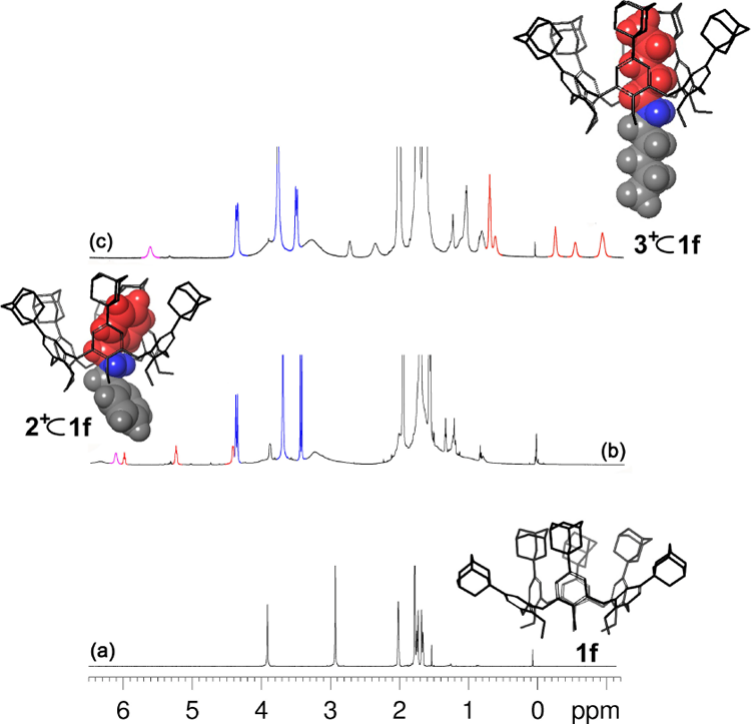
^1^H NMR spectra (CDCl_3_, 600 MHz, 298 K) of: **1f** (a); **2**^**+**^ ⊂ **1f** (b) and **3**^**+**^ ⊂ **1f** (c).

When this salt was added to a CDCl_3_ solution of **1f** (1:1 ratio) then significant changes appeared in the ^1^H NMR spectrum (Figure S24) indicative of the formation of pseudorotaxane **2**^**+**^ ⊂ **1f**. The first piece of information was the appearance of a well-spaced AX system (at 3.46/4.39 ppm, Δδ = 0.93 ppm) for the ArCH_2_Ar groups of the calix-wheel **1f** indicative of its cone conformation in pseudorotaxane **2**^**+**^ ⊂ **1f** (Figures 5b, S24, and S25).^17^ The threading of the dibenzylammonium axle was clearly evident by the presence of upfield-shifted resonances for the benzylic unit hosted inside the cavity at 4.44, 5.26, and 6.00 ppm (*ortho*-, *meta*-, and *para*-BnH, respectively) (Figure 5b); while the other outside the calix cavity was resonating at typical chemical shift values (7.90, 7.64, and 7.50 ppm, *ortho*-, *meta*-, and *para*-BnH, respectively). The threading equilibrium of **2**^**+**^ ⊂ **1f** was reached immediately after mixing and slow on the NMR time scale. The determination of the corresponding apparent association constant was carried out by means of a competition experiment^10b,10c^ (Figure S48) with the native hexahexyloxy-*p*-*tert*-butylcalix[6]arene **1b**. In particular, a 1:1 mixture of **1f** and **1b** (in CDCl_3_) was mixed with 1 equiv of **2**^**+**^·[B(Ar^F^)_4_]^−^ and equilibrated for 10 min at 298 K. The ^1^H NMR spectrum of the mixture indicated that the pseudorotaxane **2**^**+**^ ⊂ **1b** was favored over **2**^**+**^ ⊂ **1f** in a 1:0.8 ratio (Figure S48). An apparent association constant value of 7.4 ± 0.2 × 10^4^ M^–1^ was calculated from these data for the **2**^**+**^ ⊂ **1f** complex, which is significantly higher than that previously observed for the corresponding *tert*-butylated pseudorotaxane **2**^**+**^ ⊂ **1a** (2.5 ± 0.2 × 10^3^ M^–1^).^10a^ To confirm this result, a competition experiment was performed in which **2**^**+**^/**1a**/**1f** were mixed in an equimolar ratio (5.2 mM) in CDCl_3_, and the resulting ^1^H NMR spectrum of the mixture indicated that the **2**^**+**^ ⊂ **1f** pseudorotaxane was favored over **2**^**+**^ ⊂ **1a** (Figure S58). From this initial result, it is clear that the *exo*-adamantyl groups positively affect the efficiency of calix[6]arene threading probably due to more extensive favorable van der Waals interactions with the cationic guest. On the other hand, it is also evident that the bigger dimension of the *p*-adamantyl groups with respect to the *p*-*tert*-butyl ones does not hinder kinetically the equilibrium of the formation of the pseudorotaxane, being the equilibration time of **2**^**+**^ ⊂ **1f** similar to that of **2**^**+**^ ⊂ **1a**.

The evaluation of the threading properties of **1f** were then extended to the dipentylammonium axle **3**^**+**^ by using similar experimental conditions. Thus, the addition of the **3**^**+**^·[B(Ar^F^)_4_]^−^ salt to a CDCl_3_ solution of **1f** again caused the appearance of upfield-shifted resonances in the negative region of the ^1^H NMR spectrum (Figure 5c), demonstrating the formation of the pseudorotaxane **3**^**+**^ ⊂ **1f**, which was slowly exchanging in the NMR time scale. By means of a competition experiment^10a^ (Figure S49) with **1b**, an apparent association constant of 5.0 ± 0.2 × 10^4^ M^–1^ was found for **3**^**+**^ ⊂ **1f** pseudorotaxane, which was again higher than that of **3**^**+**^ ⊂ **1a** (4.4 ± 0.2 × 10^2^ M^–1^).^10b,10c^

**Figure 6:**
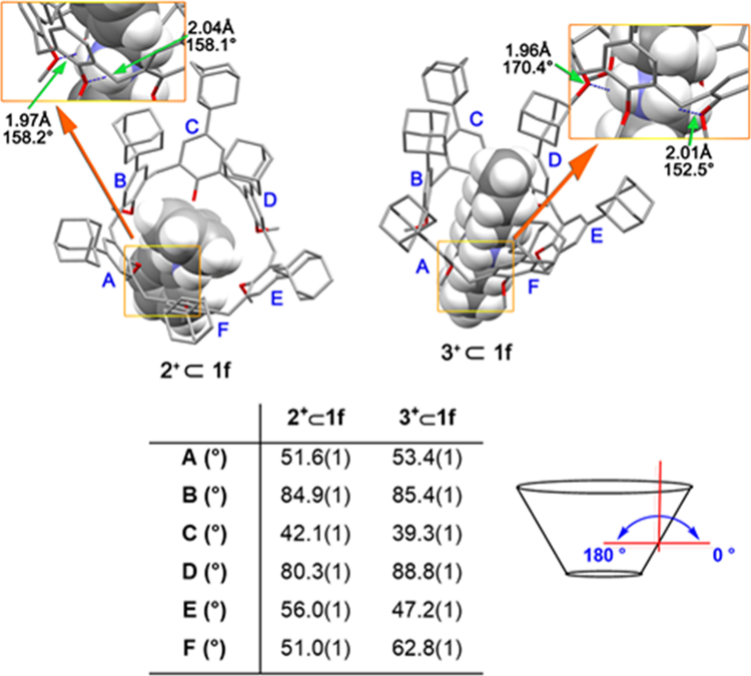
DFT-optimized structure of **2**^**+**^ ⊂ **1f** and **3**^**+**^ ⊂ **1f** pseudorotaxanes at the B3LYP/6-31G(d,p)-IOp(3/124 = 3) level of theory.

**Figure 7:**
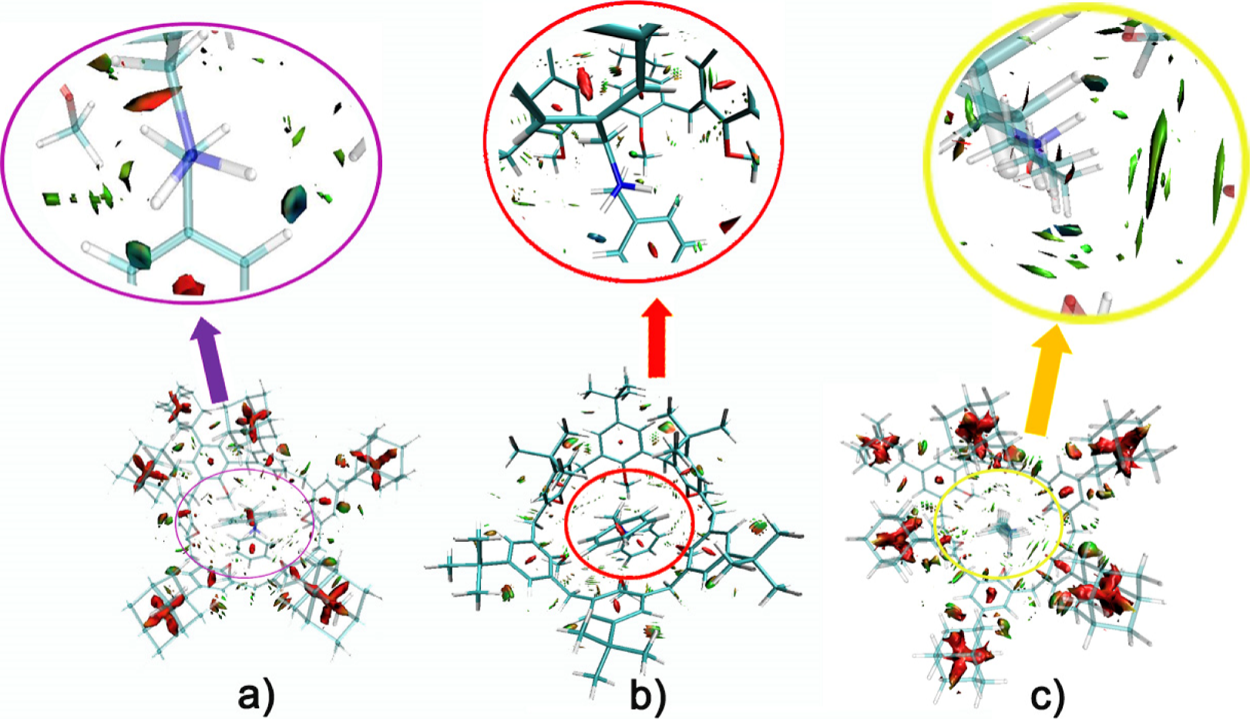
NCI plot by the sign of the second Hessian eigenvalue [gradient isosurfaces (*s* = 0.6 a.u.) for **2**^**+**^ ⊂ **1f** (a), **2**^**+**^ ⊂ **1a** (b) and **3**^**+**^ ⊂ **1f** (c)]. In the coloring isosurface, blue and green colors represent strong and medium interactions (H-bonding and van der Waals).

Further insights on the higher stability of pseudorotaxanes **2**^**+**^ ⊂ **1f** and **3**^**+**^ ⊂ **1f** were obtained by density functional theory (DFT) calculations at the B3LYP/6-31G(d,p) level of theory using Grimme’s dispersion corrections (IOp(3/124 = 3)). As can be seen from the energy-minimized structures (Figures 6 and 7), the *p*-adamantyl groups of host **1f** are all well disposed around the endo-cavity portion of guest **2**^**+**^ or **3**^**+**^. In detail, pseudo[2]rotaxane **2**^**+**^ ⊂ **1f** is stabilized by two H-bonding interactions (Figure 6) between the ammonium group and the calixarene oxygen atoms with an average N···O distance of 2.05 Å and an average N–H···O angle of approximate to 158. Additional C–H···π interactions were identified between the α and β methylene groups of the benzyl unit of **2**^**+**^ inside the calix cavity and the aromatic rings of **1f** with an average C–H···π centroid distance of 3.16 Å and an average C–H···π centroid angle of 155° (Figure S61). As a result of these contributions, the calix[6]arene macrocycle is fixed in a cone conformation. In particular, two of the six Ar rings (B and D, Figure 6) are almost orthogonally oriented with respect to the average plane defined by the six bridging methylene carbon atoms (canting angles^18^ of 84.9°(1) and 80.3°(1), respectively, while the other A, C, E, and F rings are more outward tilted (canting angles^18^ of 51.6°(1), 42.1°(1), 56.0°(1), and 51.0°(1), respectively) (Figure 6).

To further investigate the energy contribution of noncovalent interactions (NCI),^19^ a second order perturbation theory (SOPT) analysis^20^ of the Fock matrix in the natural bond orbital (NBO) basis was carried out. Interestingly, the SOPT analysis conducted on **2**^**+**^ ⊂ **1f** pseudo[2]rotaxane (Table S3) indicates that there is an articulate network of hydrogen bonding, C–H···π, and π···π interactions. In particular, we evidenced the LP(2) → σ* interaction between O3 and the N248–H277 antibonding orbital (Figure S61) and the LP(2) → σ* interaction between O4 and the N248–H250 antibonding orbital. These interactions give an energetic contribution of 10.36 and 8.34 kcal/mol, respectively, for a total energy of 18.70 kcal/mol (Table S3). Moreover, the interactions of the O6 lone pairs with the C247–H276 antibonding orbital gives an interesting contribution of 2.62 kcal/mol. The overall energy analysis (Table S3) indicates that the total energetic contribution because of secondary NCI is 34.09 kcal/mol (see Supporting Information, page S68 for further detail). Very interestingly, a similar analysis for the corresponding *tert*-butylated **2**^**+**^ ⊂ **1a** pseudo[2]rotaxane gives a lower total energy contribution of 26.66 kcal/mol (Figure S65 and Table S5).

As concerns the DFT-optimized structure of **3**^**+**^ ⊂ **1f** pseudorotaxane (Figure 6), two H-bonding interactions were identified between the NH_2_^+^ group of **3**^**+**^ and the oxygen atoms of calix-wheel **1f**, with a longer average distance of 1.98 Å and an average angle of 161.45°. In this case, the SOPT analysis (Table S4) indicated an energy contribution of 30.18 kcal/mol for these interactions. An interesting contribution on the NCI energy is due to the LP(2) → σ* interaction between O6 and the N261–H281 antibonding orbital (11.61 kcal/mol). Also in this case, the calix[6]arene macrocycle is fixed in a cone conformation with D and E rings (Figure 6) almost orthogonal with respect to the mean plane defined by all bridging methylenes (canting angle 85.4°(1) and 88.8°(1), respectively), while the other A, B, C, and E rings are more opened with canting angle values of 47.2°(1), 62.8°(1), 53.4°(1), and 39.3°(1), respectively (Figure 6). From the above two examples, we can conclude that the presence of *p*-adamantyl groups gives rise to pseudorotaxane complexes with ammonium guests containing either linear alkyl chains or aromatic rings, which are more stable than the corresponding ones with *exo*-*tert*-butyl groups by more than one order of magnitude. In both instances, the higher threading efficiency can be ascribed to the more favorable van der Waals interactions of *exo*-adamantyl groups with the cationic axle, with respect to the *tert*-butyl ones.

### Directional Threading with an Unsymmetrical Axle

During our studies,^8,10,21^ we have realized that the threading of directional calixarene-wheels with directional (or constitutionally asymmetric) alkylbenzylammonium axles could give rise to two diastereoisomeric pseudo[2]rotaxanes differing by the moiety (alkyl or benzyl) included inside the cavity. Thus, we have found several examples of directional threading of calix[6]-wheels^22^ in which the *endo*-alkyl stereoisomer is preferentially formed over the *endo*-benzyl one. These general empirical observations have induced us to introduce the so-called “*endo*-alkyl rule”^23^ to shortly refer to this preferential formation of the *endo*-alkyl stereoisomer.

**Figure 8:**
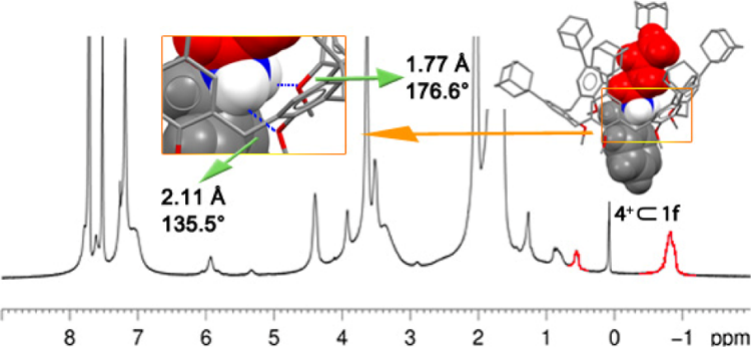
^1^H NMR spectrum (CDCl_3_, 600 MHz) at 298 K of an equimolar mixture of **4**^**+**^ and **1f**. Inset: DFT-optimized structure of **4**^**+**^ ⊂ **1f** pseudorotaxane at the B3LYP/6-31G(d,p)-IOp(3/124 = 3) level of theory.

In order to verify if this “*endo*-alkyl rule” is also valid for *p*-adamantylcalix[6]arenes, we decided to study the threading of **1f** with butylbenzylammonium axle **4**^**+**^. Thus, the addition of the **4**^**+**^·[B(Ar^F^)_4_]^−^ salt to a CDCl_3_ solution of **1f** again caused the appearance of upfield-shifted resonances in the negative region of the ^1^H NMR spectrum, demonstrating the preferential formation of the *endo*-alkyl-**4**^**+**^ ⊂ **1f** pseudorotaxane (Figure 8). Interestingly, no hints of the *endo*-benzyl-**4**^**+**^ ⊂ **1f** pseudorotaxane stereoisomer could be detected in the above experiment. In summary, we can conclude that the “*endo*-alkyl rule” is also valid for *p*-adamantylcalix[6]arenes.

### Influence of *endo*-OH Functions on Calixarene Threading

As is known, the complexation of metal cations by calixarene hosts is less efficient for those bearing free OH functions with respect to the OR-analogues.^6a^ Prompted by this consideration, we turned our attention to the endo-rim, and the question arises as to whether the presence of OH groups impairs the threading efficiency of the calix[6]-wheel. In fact, no information of this kind is currently available for the calixarene threading. Thus, we initially compared the threading properties of 1,2,4,5-tetrahexyloxycalix[6]arene-diol **1h** with the corresponding hexahexyloxy-ether **1b**, both bearing *exo*-*tert*-butyl groups. By addition of the **2**^**+**^·[B(Ar^F^)_4_]^−^ salt to a CDCl_3_ solution of **1h**, it was readily evident the formation of **2**^**+**^ ⊂ **1h** pseudorotaxane (Figure S40). The determination of the apparent association constant of 2.5 ± 0.2 × 10^3^ M^–1^ for **2**^**+**^ ⊂ **1h** revealed its significantly lower thermodynamic stability with respect to **2**^**+**^ ⊂ **1b** (1.2 ± 0.2 × 10^5^ M^–1^) (Figure S56). As expected, this can be attributed to the less favorable interactions between the OH groups and the guest in comparison with the OR ones. Finally, the ^1^H NMR competition experiment between **1b**/**1h** toward **2**^**+**^ in CDCl_3_, indicated the preferential formation of **2**^**+**^ ⊂ **1b** over **2**^**+**^ ⊂ **1h** (Figure S59). A similar result was observed for the complexation of dipentylammonium axle **3**^**+**^ with 1,2,4,5-tetrahexyloxycalix[6]arene-diol **1h**, which gave an apparent association constant of 7.1 ± 0.2 × 10^3^ M^–1^ for **3**^**+**^ ⊂ **1h** with respect to a value of 3.5 ± 0.2 × 10^4^ M^–1^ for the corresponding hexahexyloxy **3**^**+**^ ⊂ **1b** pseudorotaxane (Figure S54).

**Figure 9:**
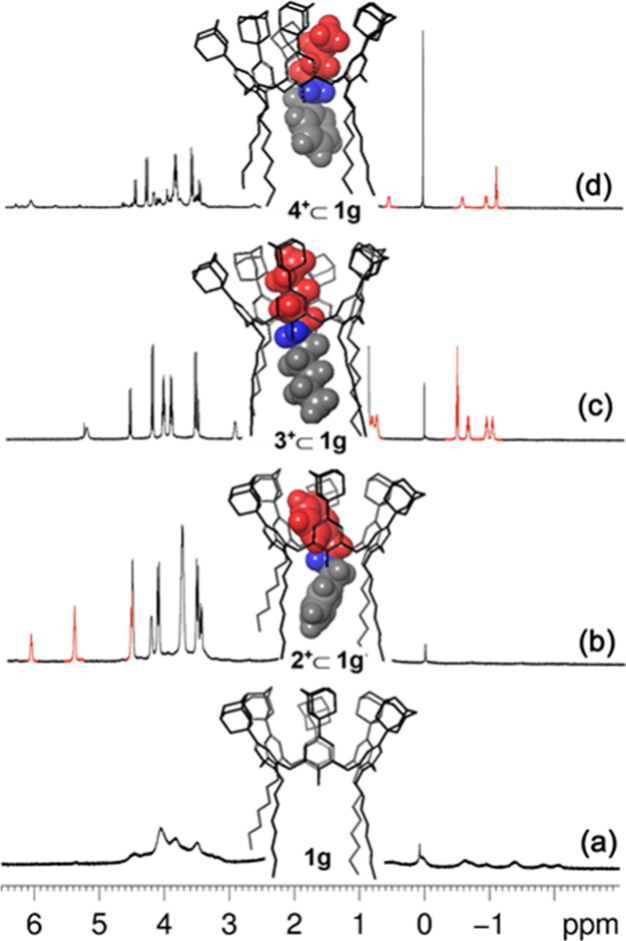
Portions of ^1^H NMR spectra (CDCl_3_, 600 MHz, 298 K) of: **1g** (a), **2**^**+**^ ⊂ **1g** (b), **3**^**+**^ ⊂ **1g** (c), and **4**^**+**^ ⊂ **1g** (d).

**Figure 10:**
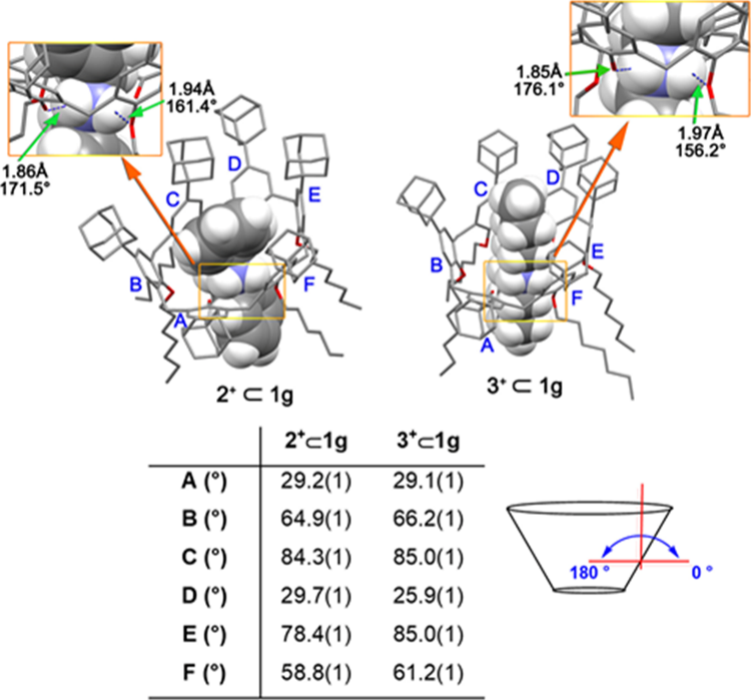
DFT-optimized structure of **2**^**+**^ ⊂ **1g** and **3**^**+**^ ⊂ **1g** pseudorotaxanes at the B3LYP/6-31G(d,p)-IOp(3/124) level of theory.

By moving to the 1,2,4,5-tetrahexyloxy-*p*-adamantyl-calix[6]arene-diol derivative **1g** (Figure 9), we expect a slight improving in the threading efficiency with respect to the *exo*-*tert*-butyl analogue **1h**, due to the above evidenced “adamantyl effect”. In fact, an apparent association constant of 8.5 ± 0.2 × 10^4^ M^–1^ was found for **2**^**+**^ ⊂ **1g** (Figures 9b and S51), which is higher than that of **2**^**+**^ ⊂ **1h** (2.5 ± 0.2 × 10^3^ M^–1^). Similar results were found for the dipentylammonium axle by comparing **3**^**+**^ ⊂ **1g** (3.4 ± 0.2 × 10^5^ M^–1^) (Figures 9c and S52) with **3**^**+**^ ⊂ **1h** (7.1 ± 0.2 × 10^3^ M^–1^). Interestingly, the threading with the unsymmetrical butylbenzylammonium axle **4**^**+**^ confirmed the validity of the “*endo*-alkyl rule” also for *endo*-OH-bearing calix[6]arene wheels **1g** (Figure 9d) and **1h**. Also in these instances, a higher thermodynamic stability was observed for the adamantylated pseudorotaxane **4**^**+**^ ⊂ **1g** (9.3 ± 0.2 × 10^4^ M^–1^) (Figures 9d and S53) with respect to the *tert*-butylated one **4**^**+**^ ⊂ **1h** (1.2 ± 0.2 × 10^3^ M^–1^). DFT calculations at the B3LYP/6-31G(d,p) level of theory using Grimme’s dispersion corrections (IOp(3/124 = 3)) were performed on pseudorotaxanes **2**^**+**^ ⊂ **1g** and **3**^**+**^ ⊂ **1g** and the corresponding energy-minimized structure are reported in Figure 10. For both the two supramolecular adducts, it is possible to observe the typical network of H-bonding interactions between the ammonium group and the oxygen atoms of the calixarene macrocycle (**2**^**+**^ ⊂ **1g**: average N···O distance 1.73 AÅ, average N–H···O angle 166.45°; **3**^**+**^ ⊂ **1g**: average N···O distance 1.91 AÅ, average N–H···O angle 166.15°). Several NCI were identified between the *endo*-cavity benzyl unit of **2**^**+**^ (or the *endo*-cavity alkyl chain of **3**^**+**^) and the aromatic rings of **1g**. All these interactions contribute to fixing the calix[6]arene macrocycle in the cone conformation. An interesting observation was found for the two distal unsubstituted phenolic rings A and D of both pseudorotaxanes. These ArOH units are more tilted outward from cavity with canting angles^18^ in the range 25.9–29.7° (Figure 10).

## Conclusions

In conclusion, we have here reported a study on the influence of the nature of the groups attached at the exo-rim and endo-rim of the calix[6]arene macrocycle on its threading properties with ammonium axles. We have here demonstrated that *exo*-adamantyl groups give rise to a more efficient threading with respect to the *exo*-*tert*-butyl ones leading to apparent association constants more than one order of magnitude higher. This higher thermodynamic stability has been ascribed to the more favorable van der Waals interactions of *exo*-adamantyls versus *exo*-*tert*-butyls with the cationic axle. In addition, we have also demonstrated that *endo*-OH functions give rise to a less efficient threading with respect to the *endo*-OR ones, in line with what was known from the complexation of alkali metal cations. We do believe that these results can be considered useful reference points for future studies on macrocycle threading and related interpenetrated architectures.

## Experimental Section

### General Comments

Reactions under anhydrous conditions were conducted under an inert atmosphere (nitrogen) using dry solvents. The commercial reagents were purchased by Aldrich and TCI Chemicals and were used without further purification. The reactions were controlled by thin-layer chromatography with Macherey-Nagel plates coated with silica gel (0.25 mm) with fluorescence indicator UV_254_ and visualized using UV light and nebulization with an indicator solution of H_2_SO_4_–Ce(SO_4_)_2_. For reactions that require heating, the heat source used was an oil bath. The reaction temperatures were measured externally using electronic thermometers. The reaction products were purified by Macherey-Nagel silica gel chromatography (60, 70–230 mesh). NMR spectra were recorded on a Bruker Avance-600 spectrometer [600 (^1^H) and 150 MHz (^13^C)], Bruker Avance-400 spectrometer [400 (^1^H) and 100 MHz (^13^C)], and Bruker Avance-300 spectrometer [300 (^1^H) and 75 MHz (^13^C)]. Chemical shifts are reported relative to the residual solvent peak (CHCl_3_: δ 7.26, CDCl_3_: δ 77.16). Standard pulse programs, provided by the manufacturer, were used for 2D NMR experiments. Structural assignments were made with additional information from correlation spectroscopy (COSY) and heteronuclear single-quantum correlation spectroscopy (HSQC), experiments. HR MALDI mass spectra were recorded on a Bruker Solarix FT-ICR mass spectrometer equipped with a 7 T magnet. The samples recorded in MALDI were prepared by mixing 10 μL of the analyte in chloroform (1 mg/mL) with 10 μL of the solution of 2,5-dihydroxybenzoic acid (10 mg/mL in methanol). The mass spectra were calibrated externally, and a linear calibration was applied.

### Synthesis of Derivative **1f**

To a stirred suspension of *p*-adamantylcalix[6]arene^13^ (0.43 g, 0.30 mmol) in dimethylformamide (DMF) (50 mL), sodium hydride (60% in mineral oil, 0.72 g, 18.00 mmol) was added. The mixture was stirred for 15 min at room temperature. Dimethylsulfate (2.72 mL, 28.80 mmol) was added and the reaction mixture was stirred at 90 °C for 20 h. After cooling, the reaction was quenched by the addition of methanol (10 mL). The solvents were evaporated under reduced pressure, and the residue was parted between dichloromethane and 2 M HCl. The organic phase was washed with water, dried with MgSO_4_, and concentrated to almost dryness. The residue was purified by column chromatography [SiO_2_, gradient from hexane to hexane/tetrahydrofuran (THF) (25:1)]. Derivative **1f** was obtained as a white solid (0.21 g, 47%). ^1^H NMR (600 MHz, CDCl_3_, 298 K): δ 7.02 (s, 12H, Ar*H*), 3.91 (s, 12H, ArC*H*_2_Ar), 2.92 (s, 18H, OCH_3_), 2.02 (br s, 18H, Ad), 1.83–1.62 (m, 72H; Ad). ^13^C{^1^H} NMR (150 MHz, CDCl_3_, 298 K): δ 154.2, 145.9, 133.5, 125.7, 59.9, 43.4, 36.8, 35.7, 31.4, 29.0. HRMS (MALDI) *m*/*z*: [M + K]^+^ calcd for C_108_H_132_KO_6_, 1564.9689; found, 1564.9690.

### Synthesis of Derivative **1g**

To a stirred suspension of *p*-adamantylcalix[6]arene^13^ (0.43 g, 0.30 mmol) in DMF (15 mL), sodium hydride (60% in mineral oil, 0.29 g, 7.2 mmol) was added. The mixture was stirred for 15 min at room temperature. 1-Bromohexane (1.01 mL, 7.20 mmol) was added and the reaction mixture was stirred at 90 °C for 20 h. After cooling, the reaction was quenched by addition of methanol (30 mL). The solid formed was collected, washed with methanol, dried, and dissolved in dichloromethane. The solution was washed with 2 M HCl, water, dried with MgSO_4_, and the solvent was evaporated under reduced pressure. The residue was purified by column chromatography [SiO_2_, 1st column: gradient from hexane to hexane/THF (50:1), 2nd column: gradient from hexane to hexane/chloroform (3:2)]. Derivative **1g** was obtained as a white solid (0.18 g, 33%). ^1^H NMR (600 MHz, TCDE, 373 K): δ 7.54 (br s, 2H, ArO*H*) 6.98–6.83 (overlapped, 12H, Ar*H*), 3.75–3.49 (overlapped, 20H, ArC*H*_2_Ar, −C*H*_2_(CH_2_)_4_CH_3_), 1.91–0.69 (overlapped, 134H, −CH_2_(C*H*_2_)_4_CH_3_, CH_2_(CH_2_)_4_C*H*_3_, Ad). ^13^C{^1^H} NMR (150 MHz, TCDE, 373 K): δ 150.8, 148.3, 144.2, 140.1, 131.2, 130.8, 125.4, 124.2, 122.4, 72.4, 41.8, 41.6, 35.3, 34.0, 33.8, 30.0, 29.4, 27.7, 27.6, 27.5, 23.4, 20.5, 12.1. HRMS (MALDI) *m*/*z*: [M + Na]^+^ calcd for C_126_H_168_NaO_6_, 1801.2757; found, 1801.2725.

### Synthesis of Derivative **6**

In a dry round flask, derivative **5**(15) (1.05 g, 1.29 mmol) was dissolved in dry acetone (70 mL). Subsequently, Cs_2_CO_3_ (12.65 g, 38.80 mmol) were added at room temperature. Afterward, hexyl iodide (9.55 mL, 64.70 mmol) was added to the reaction mixture. Stirring was continued for 24 h at reflux. After the reaction was stopped by the addition of 1 N HCl and the solution was extracted with chloroform. The organic phase was dried over anhydrous Na_2_SO_4_, filtered, and evaporated of the solvent. The raw was purified through precipitation by methanol. Derivative **6** was obtained as a white solid (1.81 g, 90%). ^1^H NMR (300 MHz, TCDE, 373 K): δ 7.27 (bd, 4H, Ar′*H*), 7.03 (bd, 4H, Ar′*H*), 6.92 (br s, 4H, Ar*H*), 6.89 (br s, 4H, Ar*H*), 6.81 (br s, 4H, Ar*H*), 4.65 (s, 4H, C*H*_2_PhCH_3_), 3.77 (overlapped, 12H, ArC*H*_2_Ar), 3.19 (bt, 8H, −C*H*_2_(CH_2_)_4_CH_3_), 2.23 (s, 6H, −OBnC*H*_3_), 1.31–0.97 (overlapped, 86H, −CH_2_(C*H*_2_)_4_CH_3_, −C(CH_3_)_3_), 0.74 (bt, 12H, −CH_2_(CH_2_)_4_C*H*_3_). ^13^C{^1^H} NMR (75 MHz, TCDE, 373 K): δ 151.8, 151.1, 143.5, 143.1, 135.3, 133.6, 131.3, 131.2, 131.1, 127.1, 126.6, 124.2, 123.9, 72.9, 72.4, 32.1, 30.1, 29.7, 28.0, 24.2, 20.7, 19.2, 12.1 HRMS (MALDI) *m*/*z*: [M + K]^+^ calcd for C_106_H_148_KO_6_, 1557.0941; found, 1557.0958.

### Synthesis of Derivative **1h**

In a round flask, derivative **6** (0.70 g, 0.45 mmol) was dissolved in chloroform (50 mL). Subsequently, Pd/C was added. Stirring was continued for 18 h at room temperature under H_2_. After this time, the reaction was stopped by filtration on Celite. The solvent was evaporated under reduced pressure. Derivative **1h** was obtained as a white solid (0.56 g, 95%). ^1^H NMR (600 MHz, TCDE, 373 K): δ 6.97–6.62 (overlapped, 14H, Ar*H*, ArO*H*), 3.73–3.60 (overlapped, 20H, ArC*H*_2_Ar, −C*H*_2_(CH_2_)_4_CH_3_), 1.15–0.74 (overlapped, 98H, −CH_2_(C*H*_2_)_4_CH_3_, CH_2_(CH_2_)_4_C*H*_3_, −C(CH_3_)_3_). ^13^C{^1^H} NMR (150 MHz, TCDE, 373 K): δ 151.2, 150.4, 146.2, 142.0, 132.9, 132.5, 126.8, 126.1, 125.2, 33.8, 33.6, 31.5, 31.2, 29.8, 29.5, 25.4, 22.3, 13.8. HRMS (MALDI) *m*/*z*: [M + K]^+^ calcd for C_90_H_132_KO_6_, 1347.9655; found, 1347.9635.

### Determination of the Crystallographic Structures of **1f** and **1g**

Colorless single crystals suitable for X-ray investigation were obtained by slow evaporation of CHCl_3_/MeOH solutions containing **1f** or **1g**. Data collections were carried out at the Macromolecular crystallography XRD1 beamline of the Elettra Synchrotron (Trieste, Italy), employing the rotating-crystal method with a Dectris Pilatus 2M area detector. Single crystals investigated were dipped in a cryo-protectant (PEG200 for **1f** and Paratone for **1g**), mounted on a loop, and flash-frozen under a liquid nitrogen stream at 100 K. Diffraction data were indexed and integrated using the XDS package,^24^ while scaling was carried out with XSCALE.^25^ The structures were solved using the SHELXT package;^26^ and structure refinement was performed with SHELXL-14,^27^ operating through the WinGX GUI,^28^ by full-matrix least-squares methods on *F*^2^. Both molecules crystallized in the *P*1̅ space group. The **1f** molecule exhibits a 1,2,3-alternate conformation; while the **1g** molecule has a partial-cone formation, with one of the hexyloxy substituted inverted with respect to the other five aryl groups in the macrocycle. Details of the refinement of the thermal parameters of non-hydrogen atoms are outlined below. All hydrogen atoms were placed at the geometrically calculated positions and refined using the riding model. Crystal data and final refinement details for the structures are reported in Tables S5 and S6.

#### Crystal Structure of **1f**

The triclinic (space group *P*1̅) asymmetric unit contains a 1/2 molecule of **1f** which lies on a center of inversion and two co-crystallized CHCl_3_ solvent molecules encapsulated in the calixarene ring. The structure exhibits no appreciable disorder and all non-hydrogen atoms were refined anisotropically at full occupancy.

#### Crystal Structure of **1g**

The triclinic (space group *P*1̅) asymmetric unit contains one molecule of **1g** and four CHCl_3_ molecules modeled with partial occupancy. The **1g** molecule shows several types of disorder. The most significant disorder is attributed to the superimposition on the same site of the two enantiomeric forms of **1g**, which results in a partial occupation of the hexyloxy groups (2, 3 and 5, 6) located on each side of a plane containing the inverted phenyl group and its facing phenyl group with self-included hexyl chain. Each of the four affected positions (2, 3, 5, and 6) was refined with a hydrogen atom and a hexyl group at 50% of occupancy factors, with the overlapped oxygen atoms at full occupation. In addition, two of these hexyl substituents show two-position disorder for all six carbon atoms, which were refined at 30 and 20% of occupancy factors in both cases. A similar two-position disorder is present for all six carbon atoms of the hexyloxy group bonded to the inverted phenyl group; as well as all six hexyloxy carbon atoms of its facing phenyl group. These were refined at 50% of occupancy factors in both cases. Finally, one of the adamantyl groups shows a two-position disorder of all its carbon atoms (except for the atom bonded to the phenyl ring) which was refined at 60 and 40% of occupancy factors. Significant disorder and partial occupancy are also observed for the solvent molecules. One fully occupied CHCl_3_ molecule site shows a three-position disorder, modeled at 40, 40, and 20% of occupancy factors; while a second, partially occupied site shows a two-position disorder modeled at 50 and 40% of occupancy factors. Finally, two further CHCl_3_ sites were modeled at 20 and 15% of occupancy factors. These two partially occupied sites are superimposed on sites of the partially occupied hexyl groups discussed above. In order to maintain a regular geometry, DFIX and DANG were applied to all partially occupied atoms discussed above. In addition, the thermal parameters of all partially occupied carbon atoms were refined isotropically, while all other non-hydrogen atoms were refined anisotropically.

## Supporting Information Available

Copies of ^1^H and ^13^C NMR spectra for all products, HRMS, 2D NMR, ^1^H VT NMR spectra, NMR titrations, and 2D NMR spectra of complexes, and computational studies (PDF)
Crystallographic data of 1f (CIF)
Crystallographic data of 1g (CIF)

## Supplementary Material

jo0c01769_si_001.pdf

jo0c01769_si_002.cif

jo0c01769_si_003.cif
