# Supplementary material for: Influence of exo-Adamantyl Groups and endo-OH Functions on the Threading of Calix[6]arene Macrocycle
Source: J Org Chem. 2020 Sep 9;85(19):12585–93. doi: 10.1021/acs.joc.0c01769 (PMC8011915; doi:10.1021/acs.joc.0c01769)
Supplement: Supplementary file 1 — jo0c01769_si_001.pdf [file jo0c01769_si_001.pdf]

Supporting Information

Influence of *exo*-Adamantyl Groups and *endo*-OH Functions on the Threading of Calix[6]arene Macrocycle

Veronica Iuliano,[1] Carmen Talotta,[1,] Carmine Gaeta,[1] Neal Hickey,[2] Silvano Geremia,[2] Ivan Vatsouro,[3] Vladimir Kovalev, [3,*] and Placido Neri[1,*]*

[1]Laboratory of Supramolecular Chemistry, Department of Chemistry and Biology "A. Zambelli", University of Salerno, Via Giovanni Paolo II 132, I-84084 Fisciano, Salerno, Italy.

[2] Centro di Eccellenza in Biocristallografia, Dipartimento di Scienze Chimiche e Farmaceutiche, Università di Trieste, via L. Giorgieri 1, I-34127 Trieste, Italy

[3]Department of Chemistry, M. V. Lomonosov Moscow State University, Lenin's Hills 1, 119991 Moscow, Russia.

e-mail: ctalotta@unisa.it, kovalev@petrol.chem.msu.ru, neri@unisa.it,

TABLE OF CONTENTS

Chart 1S S4

^{1}H NMR Spectrum of derivative **1f** S5

^{13}C NMR Spectrum of derivative **1f** S6

2D HSQC Spectrum of derivative **1f** S7

HR-MS Spectrum of derivative **1f** S8

^{1}H NMR Spectrum of derivative **1g** S9

^{13}C NMR Spectrum of derivative **1g** S11

HR-MS Spectrum of derivative **1g** S12

LT NMR Spectra of derivative **1g** S13

2D COSY spectrum of derivative **1g** S14

2D HSQC spectrum of derivative **1g** S15

^{1}H NMR spectrum of derivative **6** S16

^{13}C NMR spectrum of derivative **6** S17

HR-MS Spectrum of derivative **6** S18

^{1}H NMR Spectrum of derivative **1h** S19

HT NMR Spectra of derivative **1h** S20

^{13}C NMR Spectrum of derivative **1h** S21

2D HSQC Spectrum of Derivative **1h** S22

HR-MS Spectrum of derivative **1h** S23

LT NMR Spectra of derivative **1h** S24

2D COSY Spectrum of derivative **1h** S25

2D HSQC NMR Spectrum of derivative **1h** S26

Synthesis of pseudo[2]rotaxanes S27

^{1}H NMR Spectrum of Derivative $\mathbf{4^{+}} \subset$ **1b** S28

^{1}H NMR Spectrum of Derivative $\mathbf{2^{+}} \subset$ **1f** S29

2D COSY Spectrum of Derivative $\mathbf{2^{+}} \subset$ **1f** S30

^{1}H NMR Spectrum of Derivative $\mathbf{3^{+}} \subset$ **1f** S31

2D COSY Spectrum of Derivative $\mathbf{3^{+}} \subset$ **1f** S32

2D HSQC Spectrum of Derivative $\mathbf{3^{+}} \subset$ **1f** S33

^{1}H NMR Spectrum of Derivative $\mathbf{4^{+}} \subset$ **1f** S34

2D COSY Spectrum of Derivative $\mathbf{4^{+}} \subset$ **1f** S35

^{1}H NMR Spectrum of Derivative $\mathbf{2^{+}} \subset$ **1g** S36

2D COSY Spectrum of Derivative $\mathbf{2^{+}} \subset$ **1g** S37

2D HSQC Spectrum of Derivative $\mathbf{2^{+}} \subset$ **1g** S38

^{1}H NMR Spectrum of Derivative **3$^+$$\subset$ 1g**	S39
2D COSY Spectrum of Derivative **3$^+$$\subset$ 1g**	S40
2D HSQC Spectrum of Derivative **3$^+$$\subset$ 1g**	S41
[1]H NMR Spectrum of Derivative **4$^+$$\subset$ 1g**	S42
2D COSY Spectrum of Derivative **4$^+$$\subset$ 1g**	S43
2D HSQC Spectrum of Derivative **4$^+$$\subset$ 1g**	S44
^{1}H NMR Spectrum of Derivative **2$^+$$\subset$ 1h**	S45
[1]H NMR Spectrum of Derivative **3$^+$$\subset$ 1h**	S46
2D COSY Spectrum of Derivative **3$^+$$\subset$ 1h**	S47
2D HSQC Spectrum of Derivative **3$^+$$\subset$ 1h**	S48
^{1}H NMR Spectrum of Derivative **4$^+$$\subset$ 1h**	S49
2D COSY Spectrum of Derivative **4$^+$$\subset$ 1h**	S50
^{1}H NMR determination of K_{ass} values	S51
Competition experiments	S65
Computational studies	S67
Atomic Coordinates	S74
Crystallographic information	S116

Chart 1S

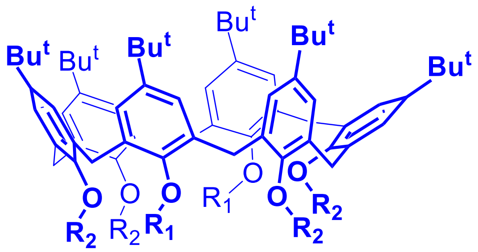

1a) R_1= R_2= Methyl
1b) R_1= R_2= Hexyl
1h) R_1= H; R_2= Hexyl

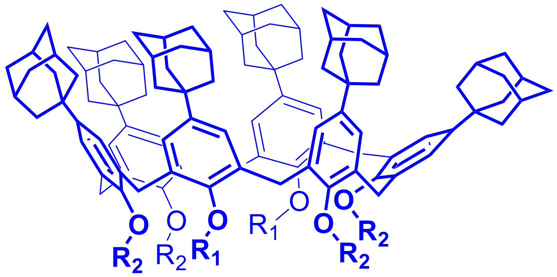

1f) R_1= R_2= Methyl
1g) R_1= H; R_2= Hexyl

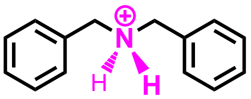

$2^+[B(Ar^F)_4]^-$

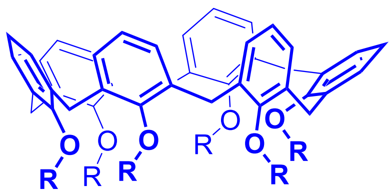

1d) R= Methyl
1e) R= Hexyl

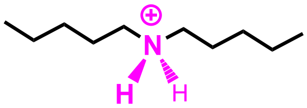

$3^+[B(Ar^F)_4]^-$

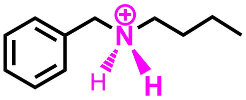

$4^+[B(Ar^F)_4]^-$

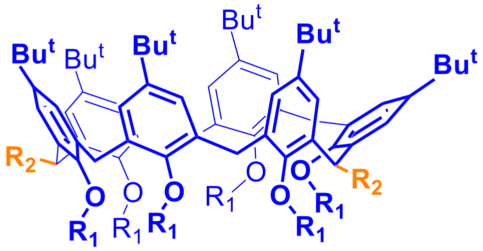

1c) R_1= Methyl; R_2=Butyl

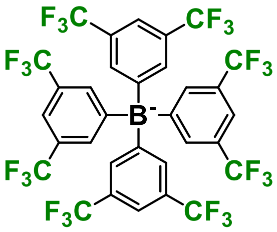

$[B(Ar^F)_4]^-$ Anion

Chart 1 Calix[6]arene derivatives and linear system

^{1}H NMR Spectrum of derivative 1f

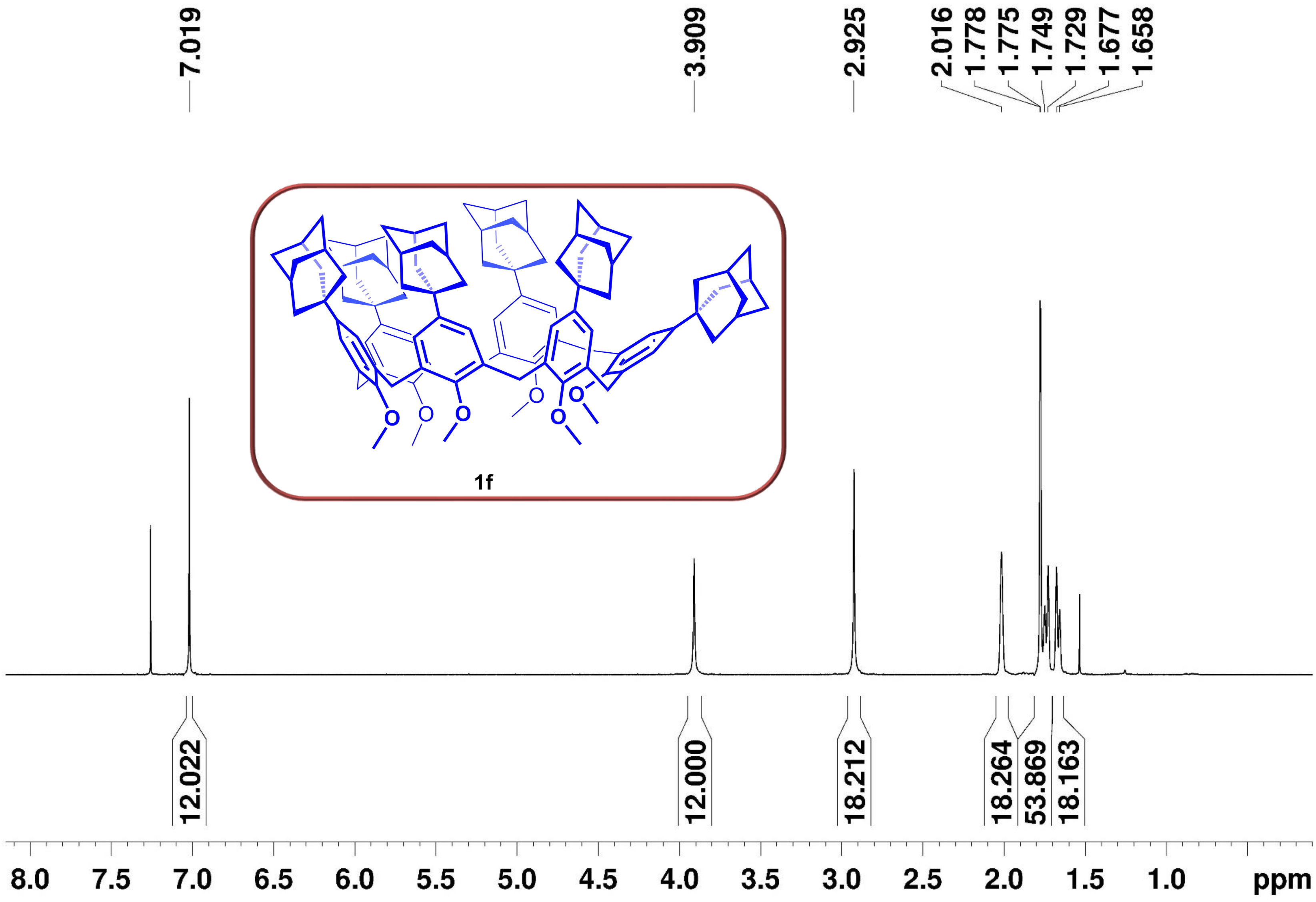

Figure S1. ^{1}H NMR spectrum of derivative **1f** (600 MHz, $CDCl_3$, 298K).

^{13}C NMR Spectrum of derivative 1f

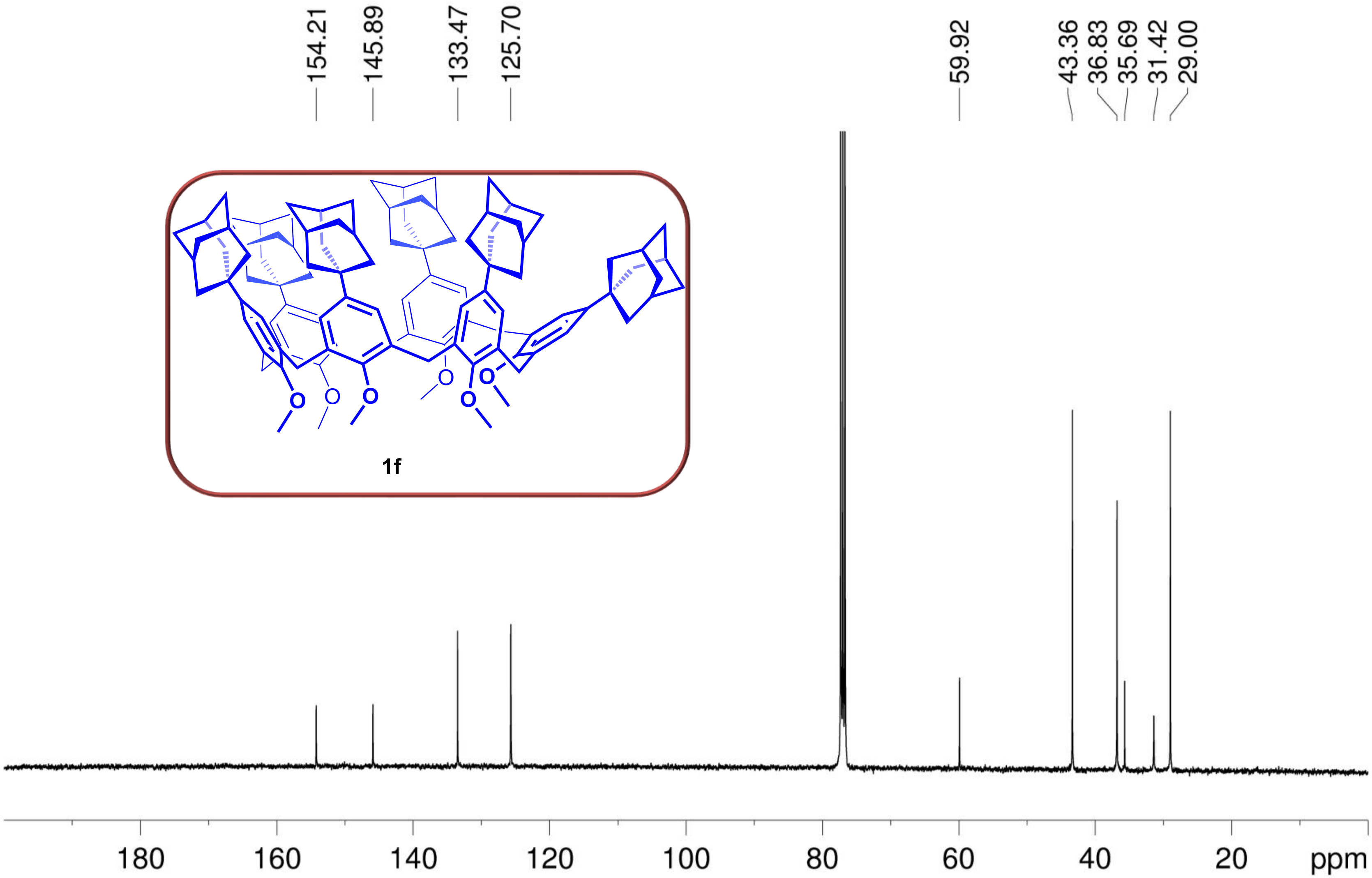

Figure S2. ^{13}C NMR spectrum of derivative **1f** (150 MHz, $CDCl_3$, 298K).

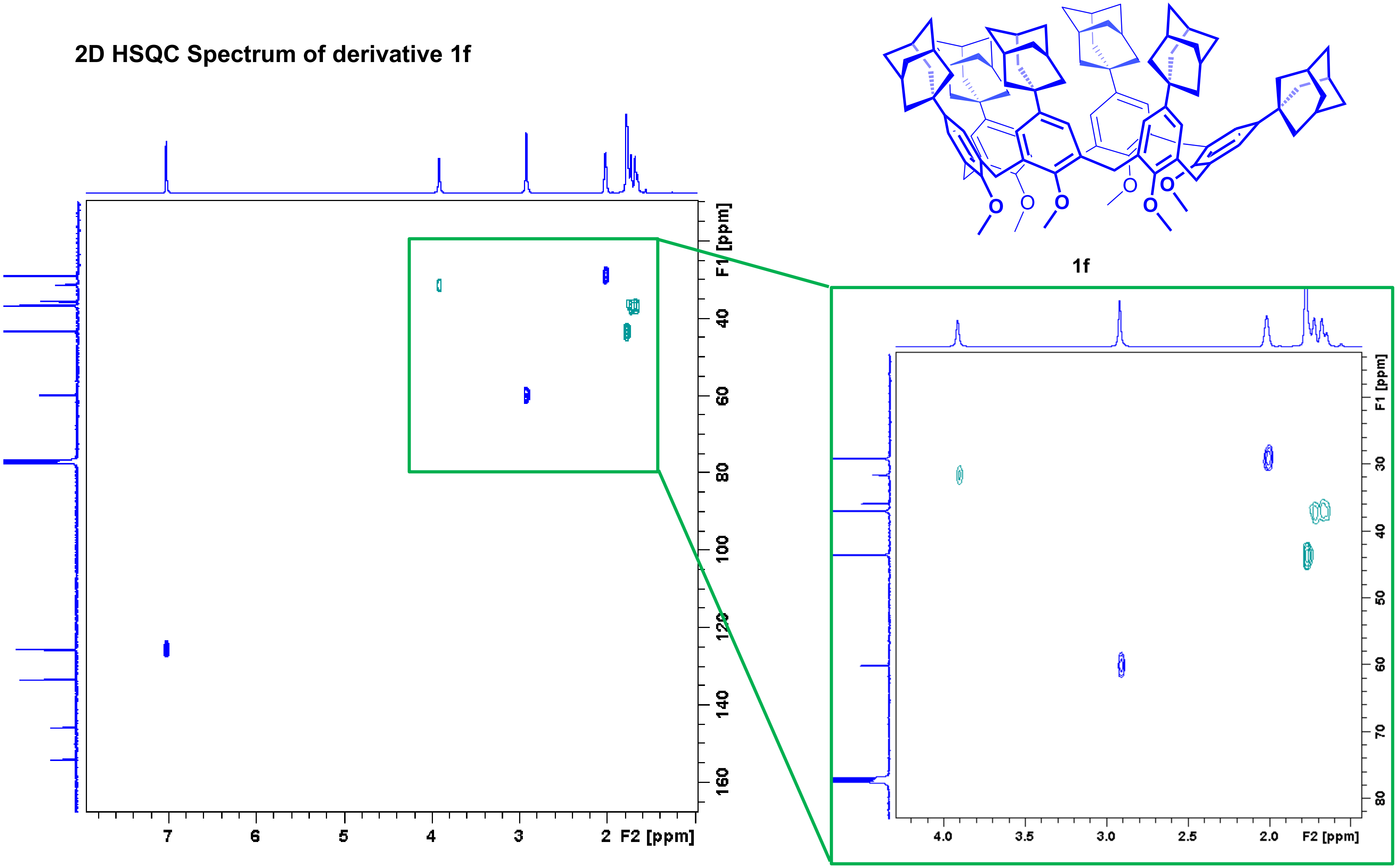

Figure S3. 2D HSQC spectrum of derivative **1f** (600 MHz, $CDCl_3$, 298K).

HR-MS Spectrum of derivative 1f

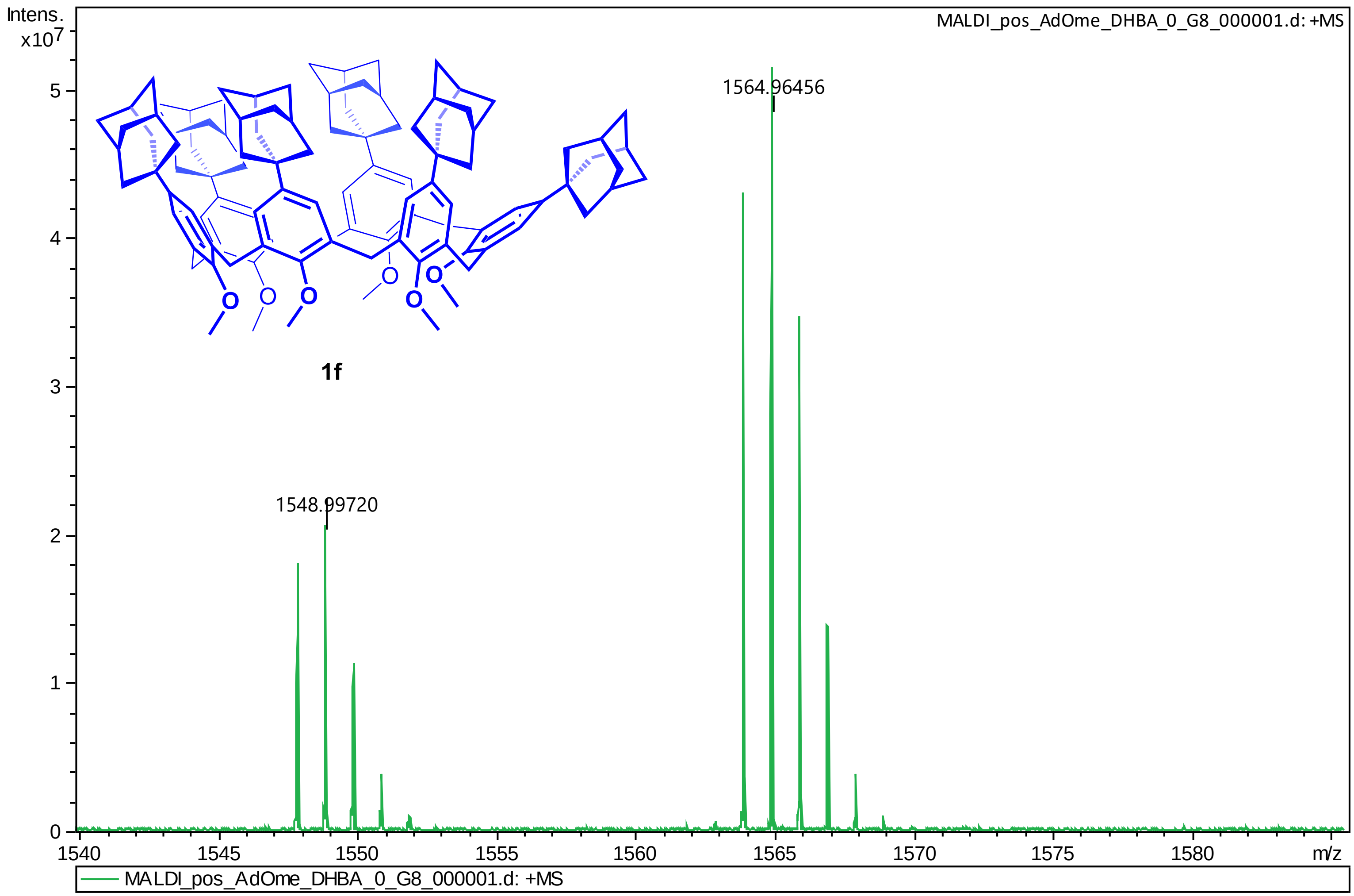

Figure S4. HR-MS spectrum of derivative **1f**.

^{1}H NMR Spectrum of derivative 1g

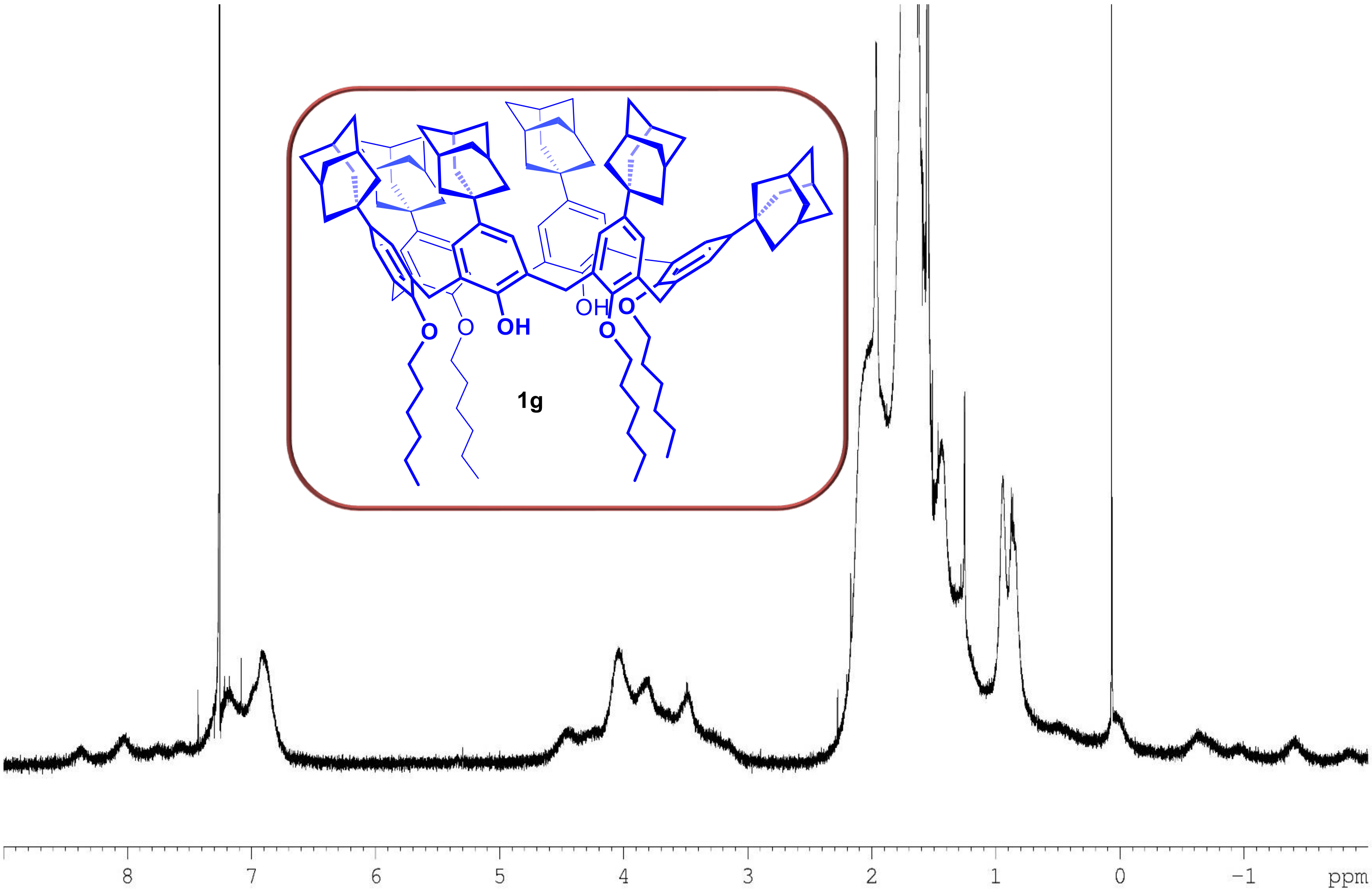

Figure S5. ^{1}H NMR spectrum of derivative **1g** (600 MHz, $CDCl_3$, 298K).

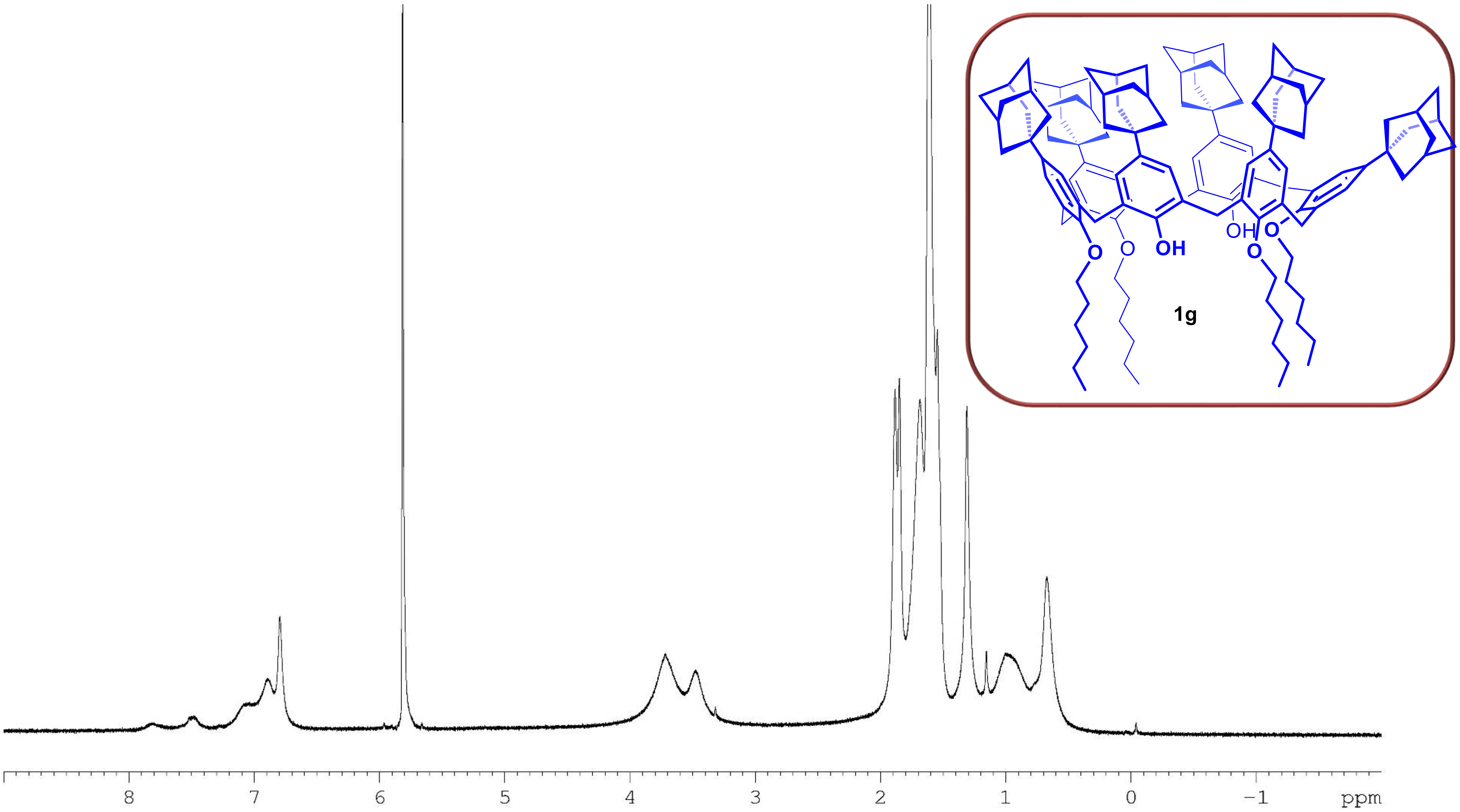

Figure S6. ^{1}H NMR spectrum of derivative **1g** (600 MHz, TCDE, 373K).

^{13}C NMR Spectrum of derivative 1g

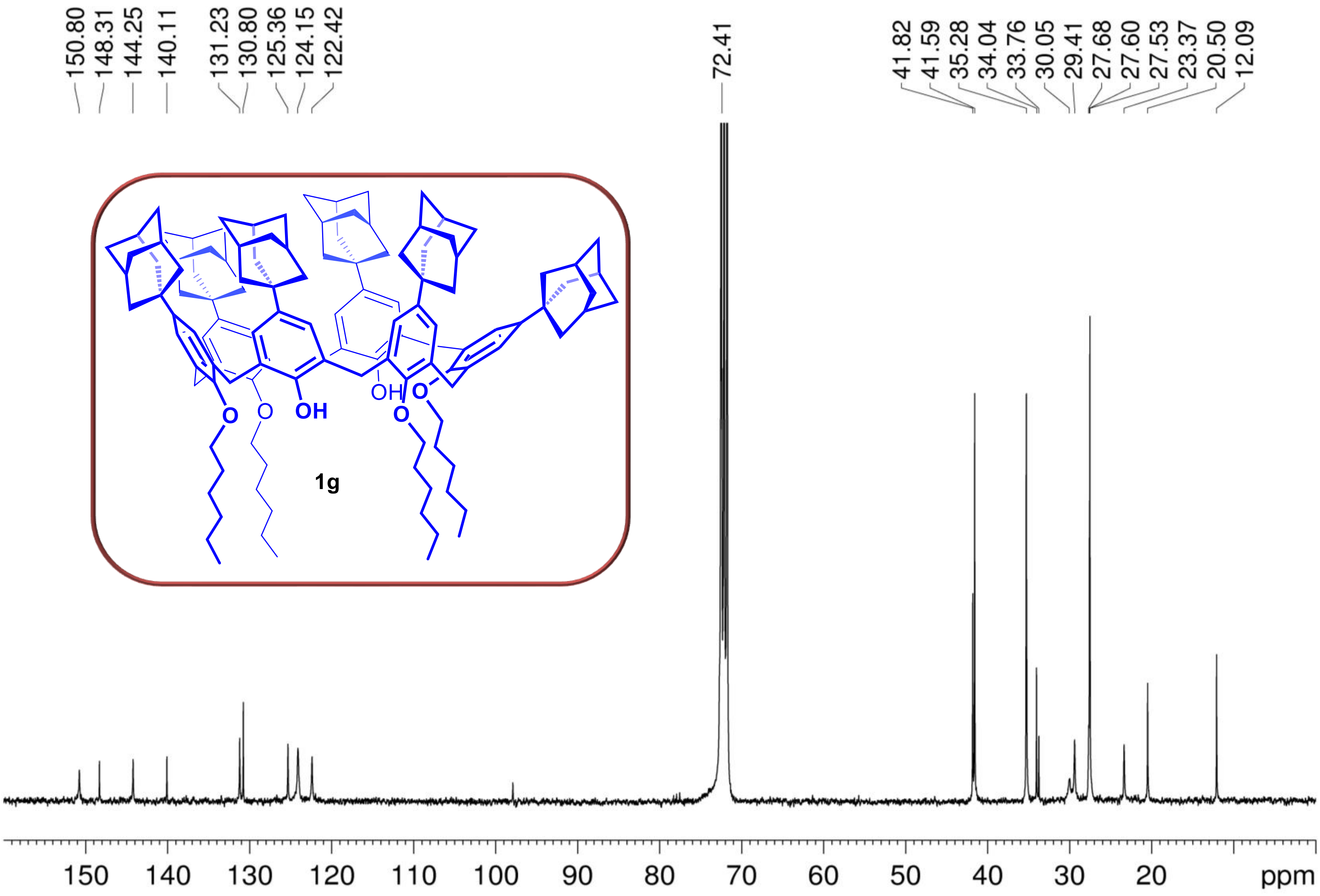

Figure S7. ^{1}H NMR spectrum of derivative **1g** (600 MHz, TCDE, 373K).

HR-MS Spectrum of derivative 1g

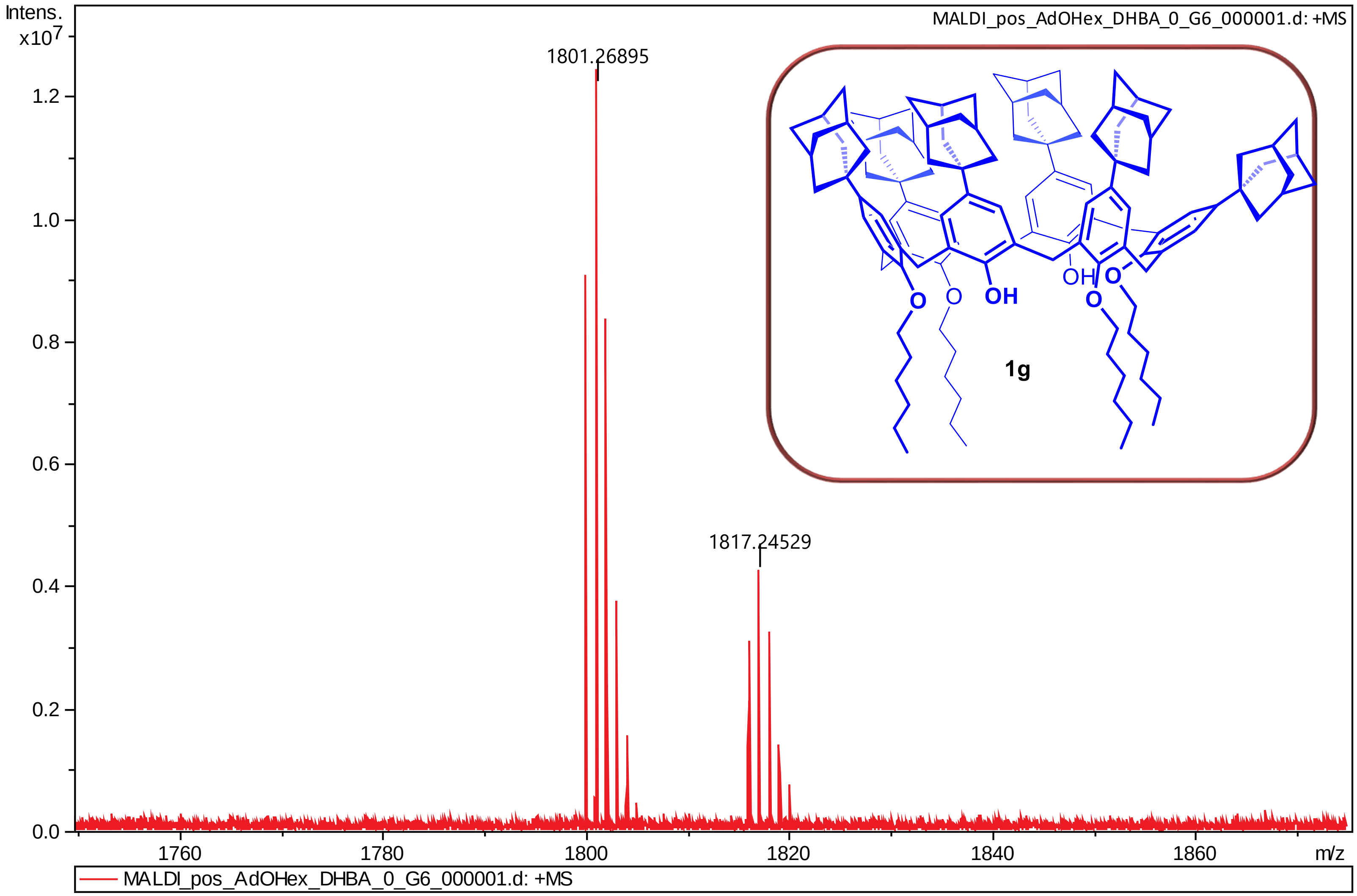

Figure S8. HR-MS spectrum of derivative **1g**.

LT NMR Spectra of derivative 1g

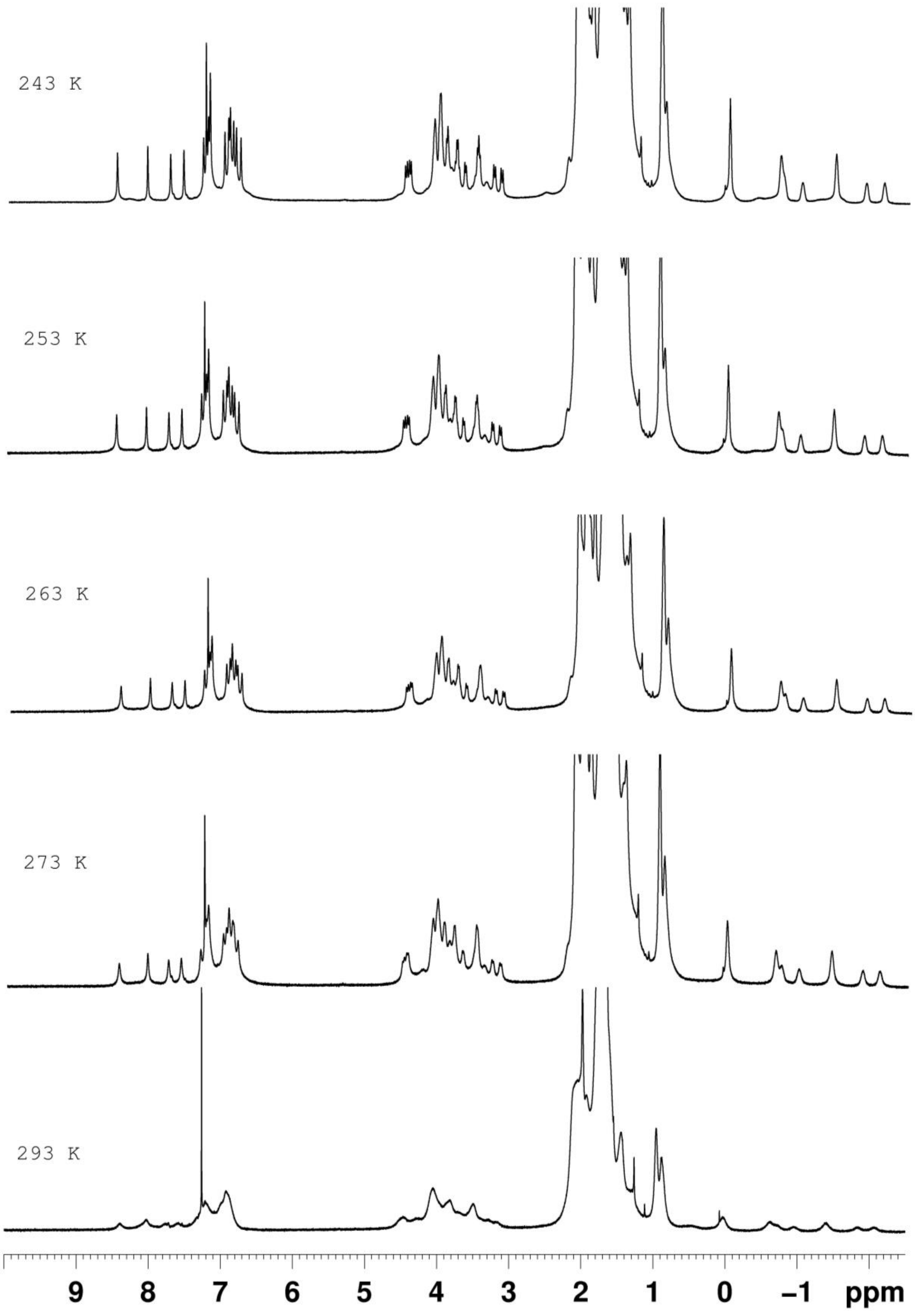

Figure S9. ^{1}H NMR spectrum of derivative **1g** (600 MHz, $CDCl_3$).

2D COSY spectrum of derivative 1g

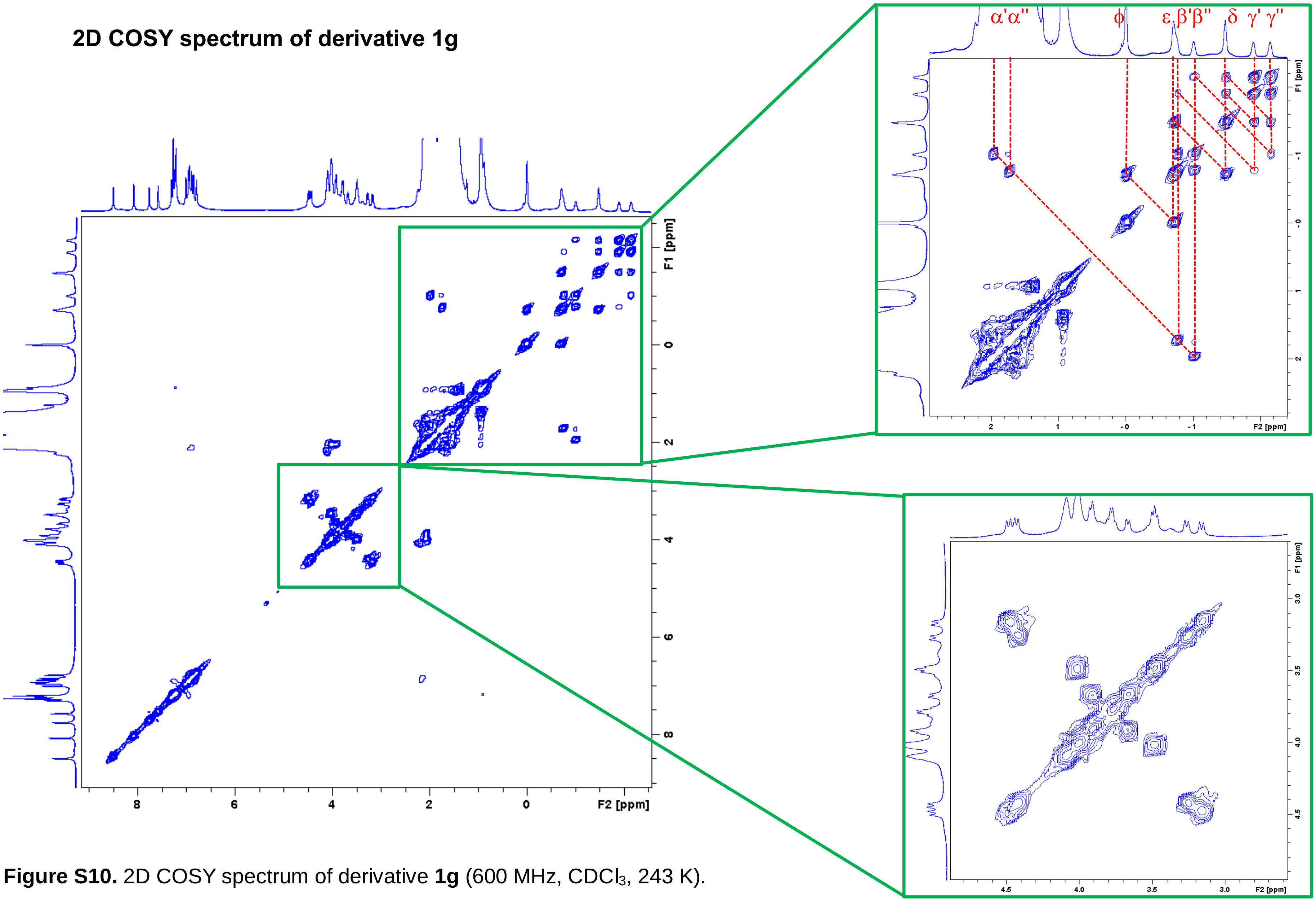

Figure S10. 2D COSY spectrum of derivative **1g** (600 MHz, $CDCl_3$, 243 K).

2D HSQC spectrum of derivative 1g

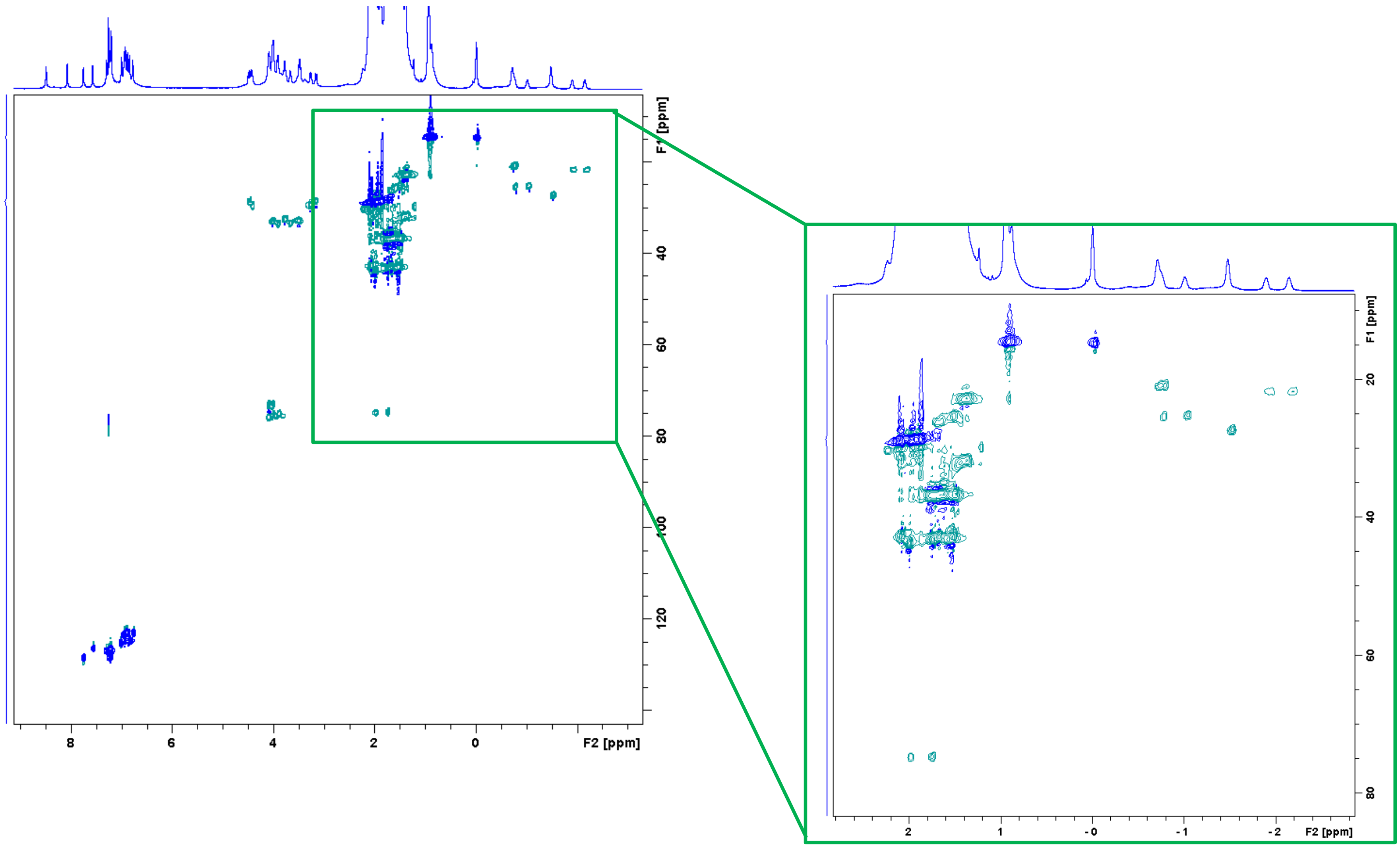

Figure S11. 2D HSQC spectrum of derivative **1g** (600 MHz, $CDCl_3$, 243 K).

^{1}H NMR spectrum of derivative 6

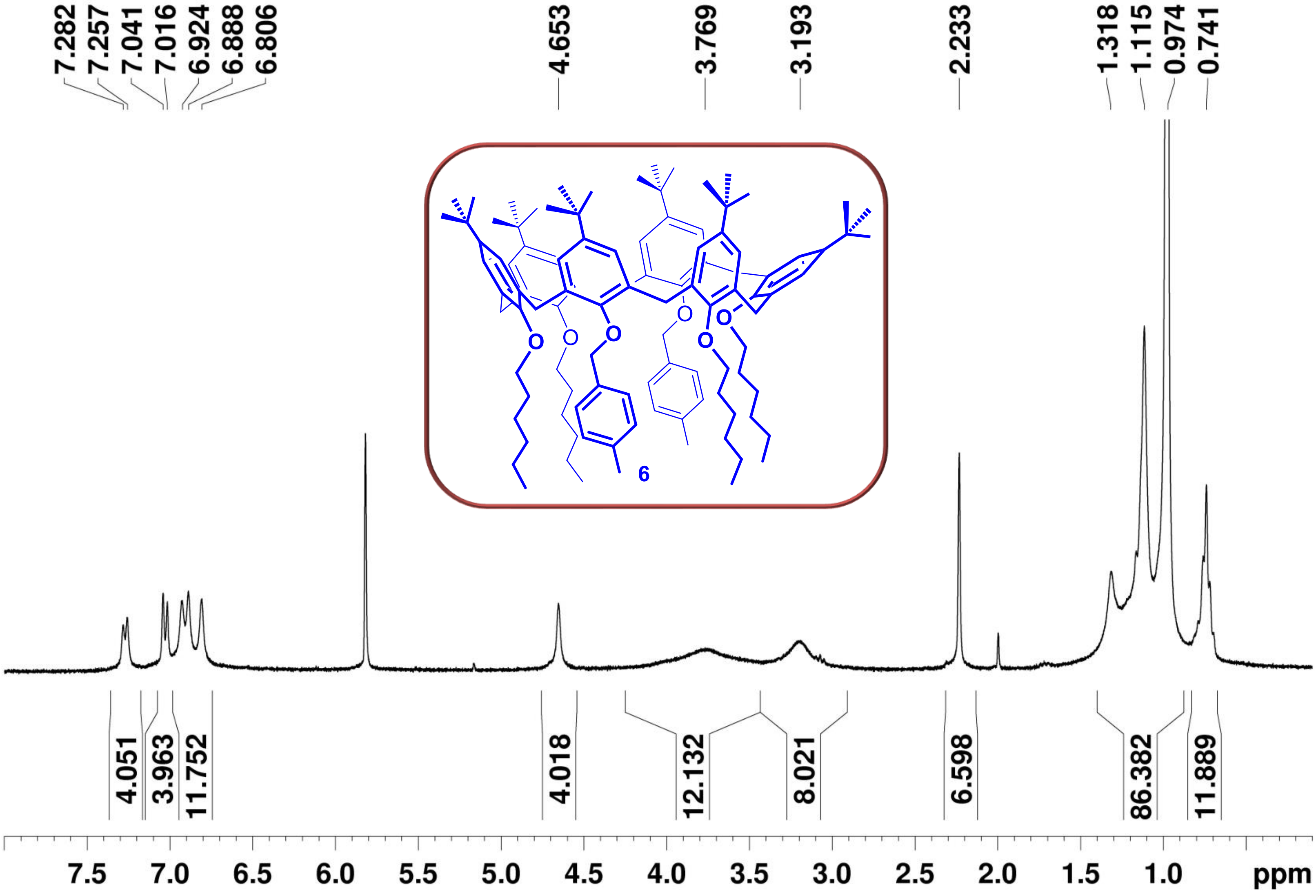

Figure S12. ^{1}H NMR spectrum of derivative **6** (600 MHz, TCDE, 373K).

^{13}C NMR spectrum of derivative 6

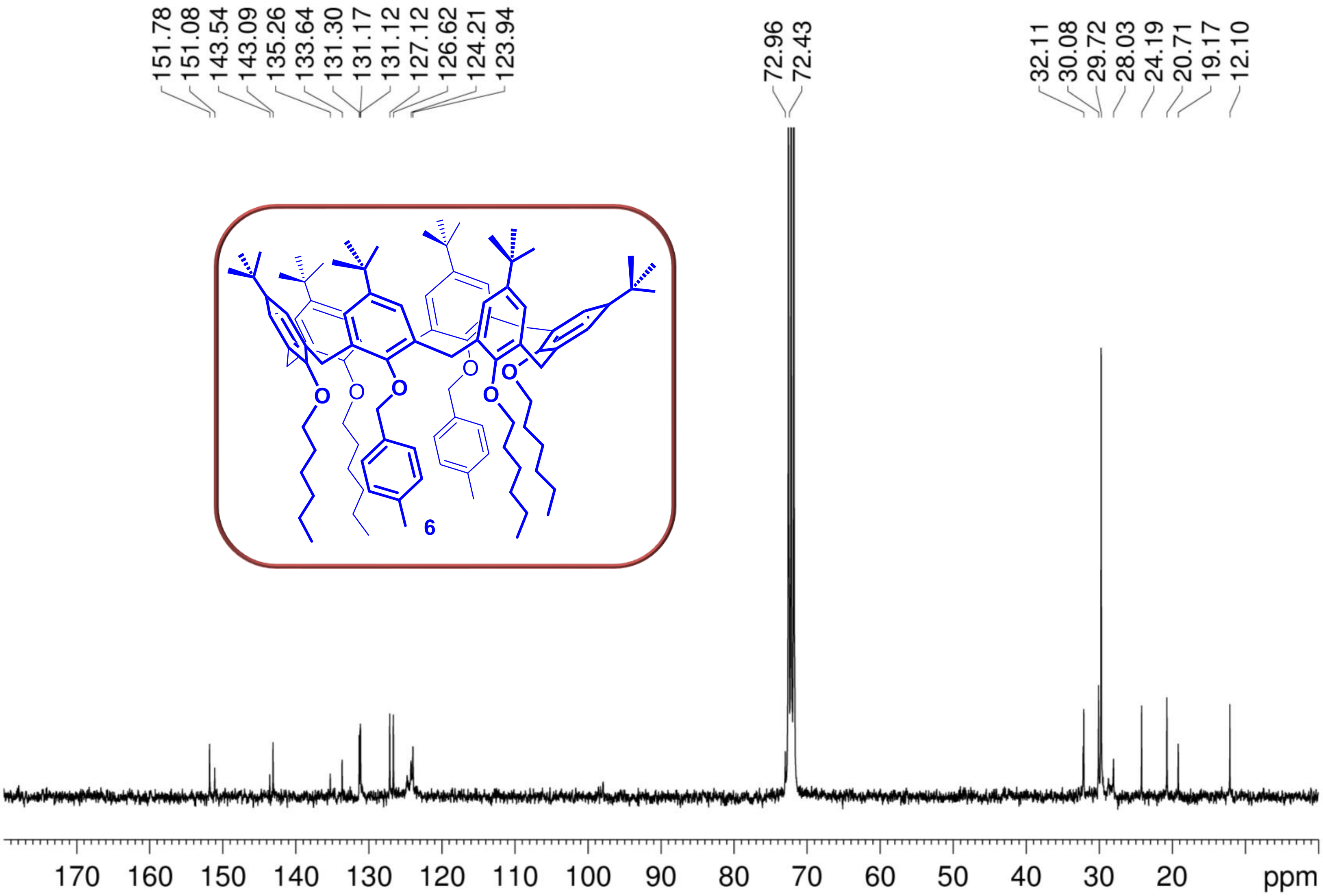

Figure S13. ^{1}H NMR Spectrum of derivative **6** (600 MHz, TCDE, 373 K).

HR-MS Spectrum of derivative 6

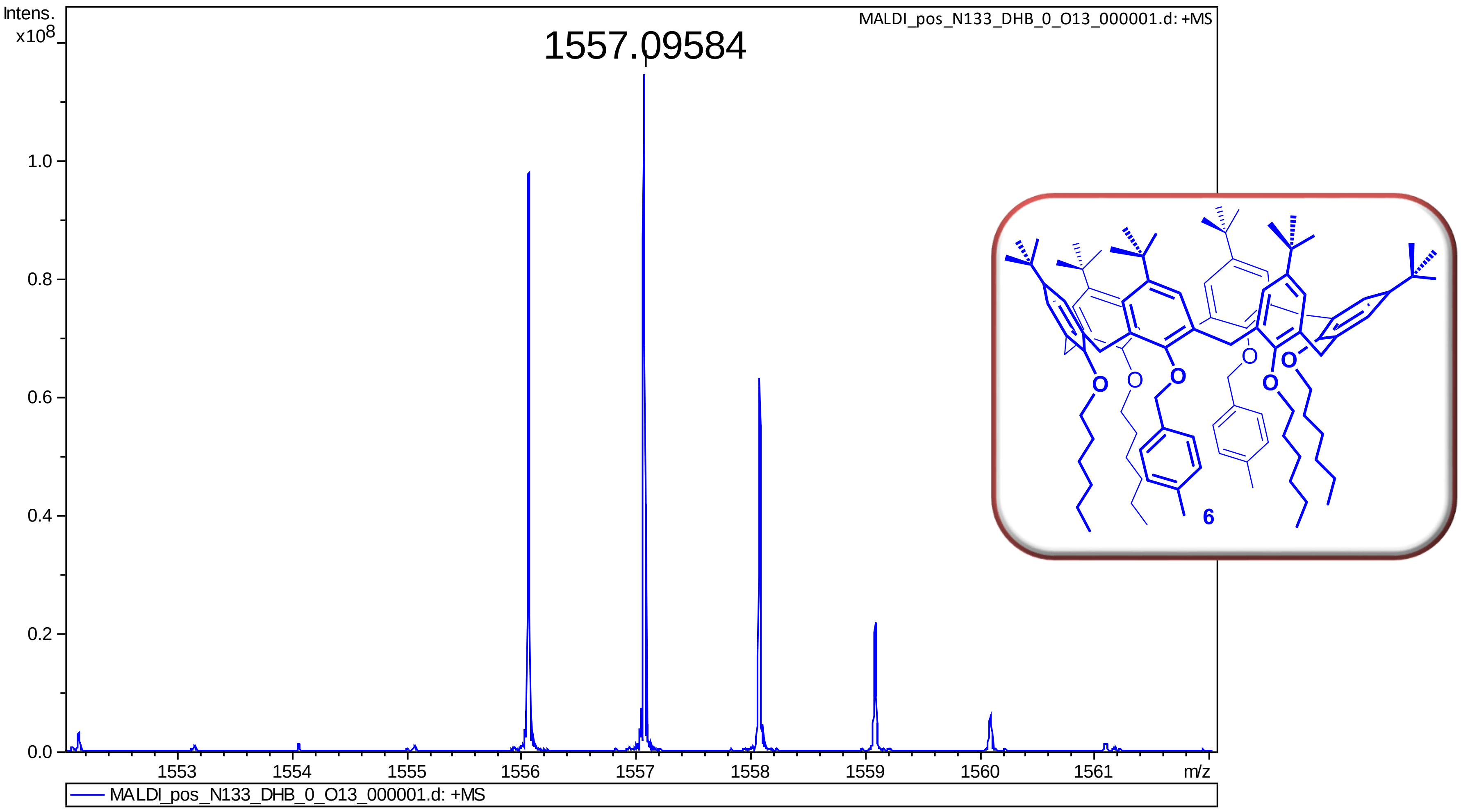

Figure S14. HR-MS spectrum of derivative **6**.

^{1}H NMR Spectrum of derivative 1h

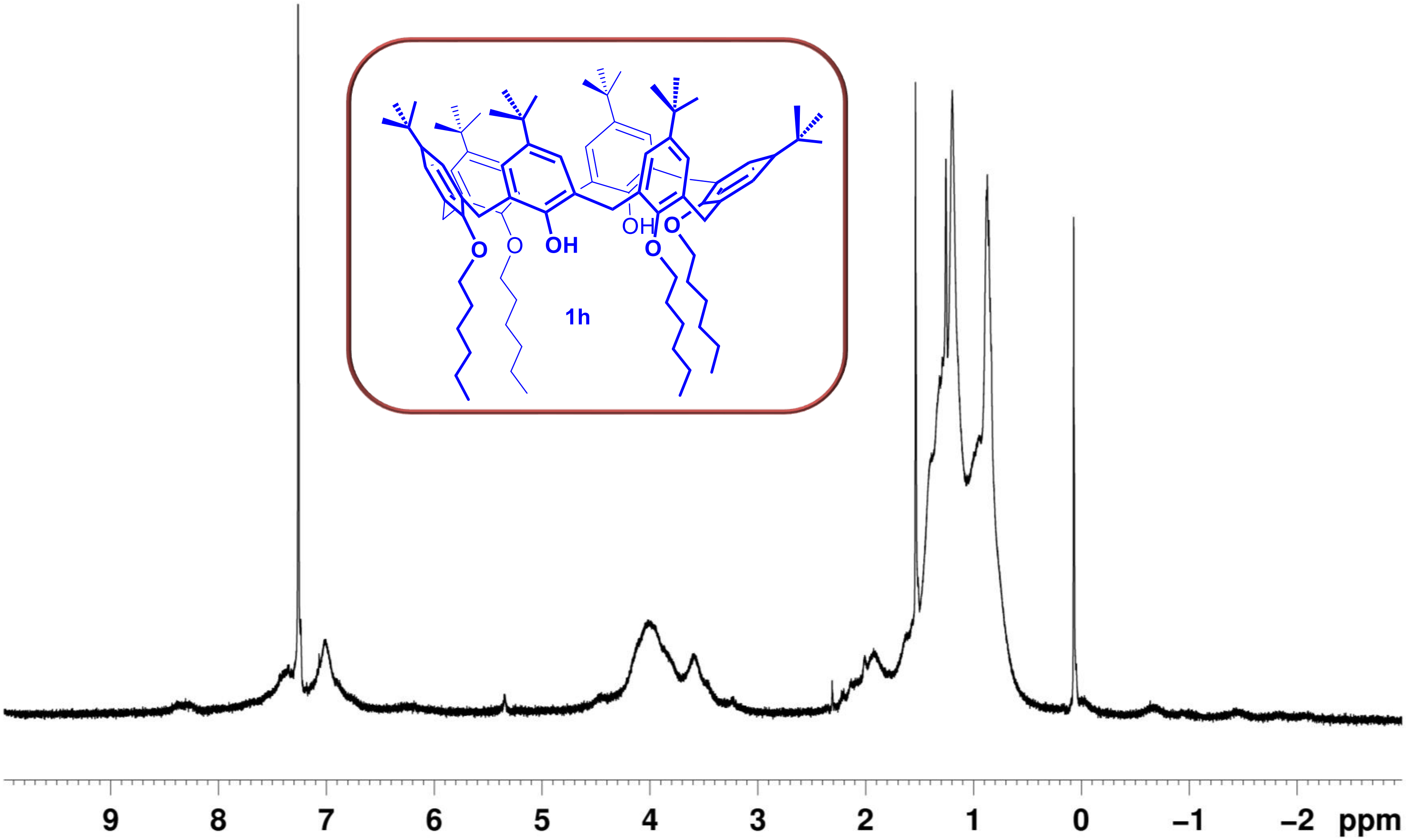

Figure S15. ^{1}H NMR spectrum of derivative **1h** (600 MHz, $CDCl_3$, 298K).

HT NMR Spectra of derivative 1h

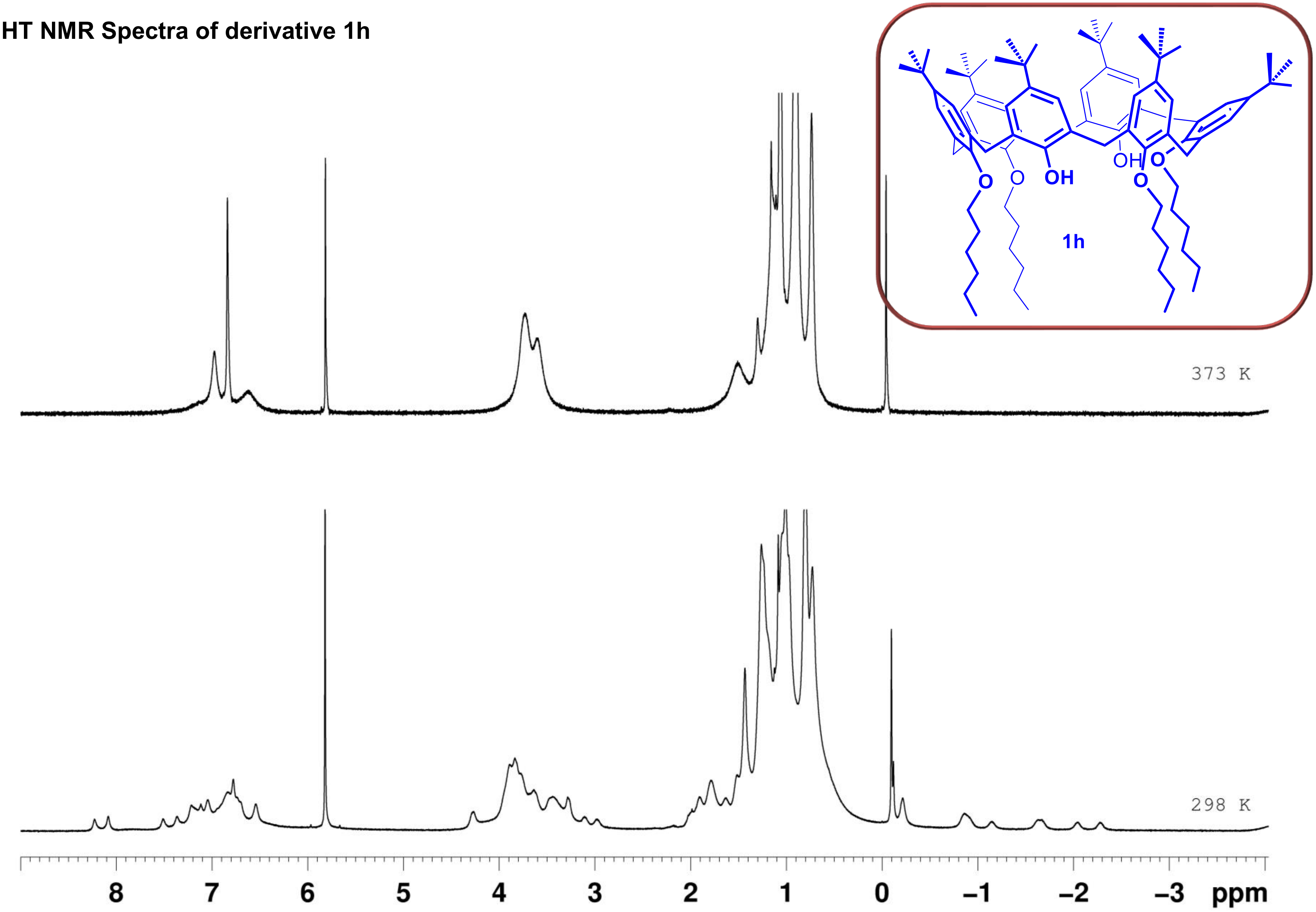

Figure S16. ^{1}H NMR spectra of derivative **1h** (600 MHz, TCDE).

^{13}C NMR Spectrum of derivative 1h

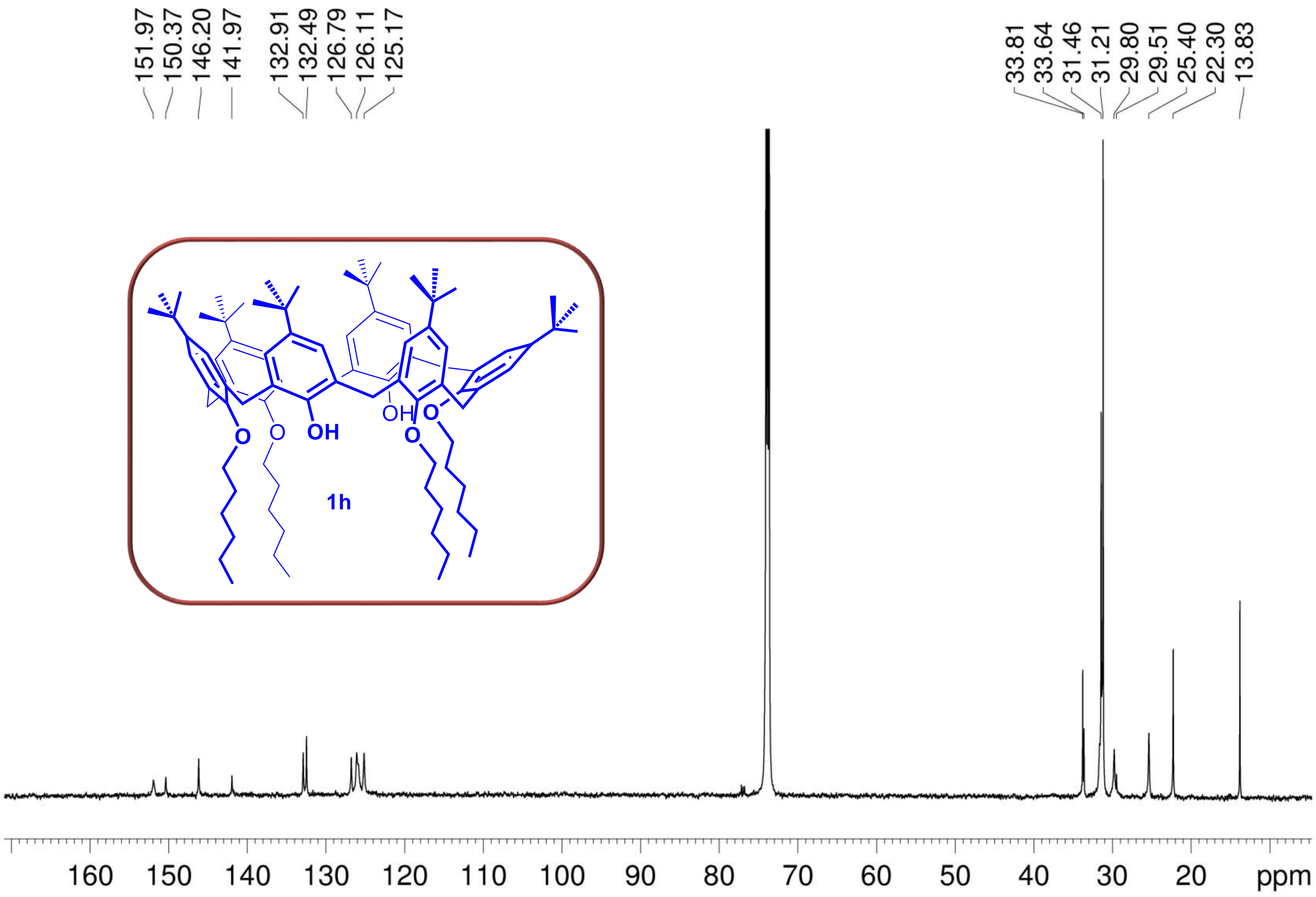

Figure S17. ^{13}C NMR spectrum of derivative **1h** (600 MHz, TCDE, 373 K).

2D HSQC Spectrum of Derivative 1h

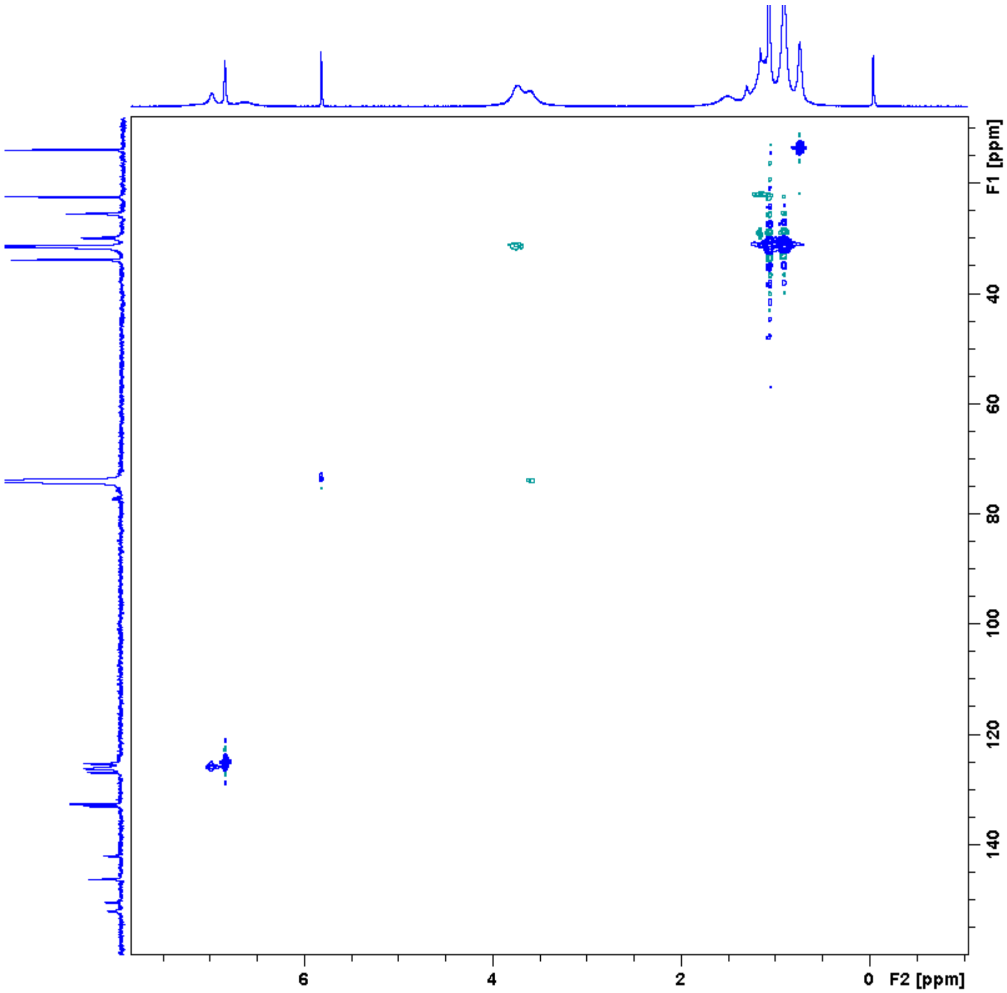

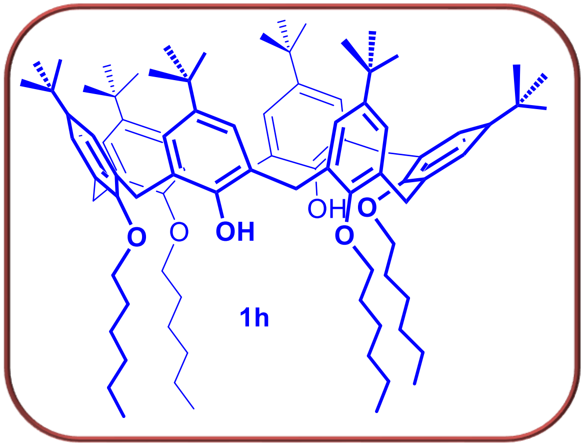

Figure S18. 2D HSQC spectrum of derivative **1h** (600 MHz, TCDE, 373 K).

HR-MS Spectrum of derivative 1h

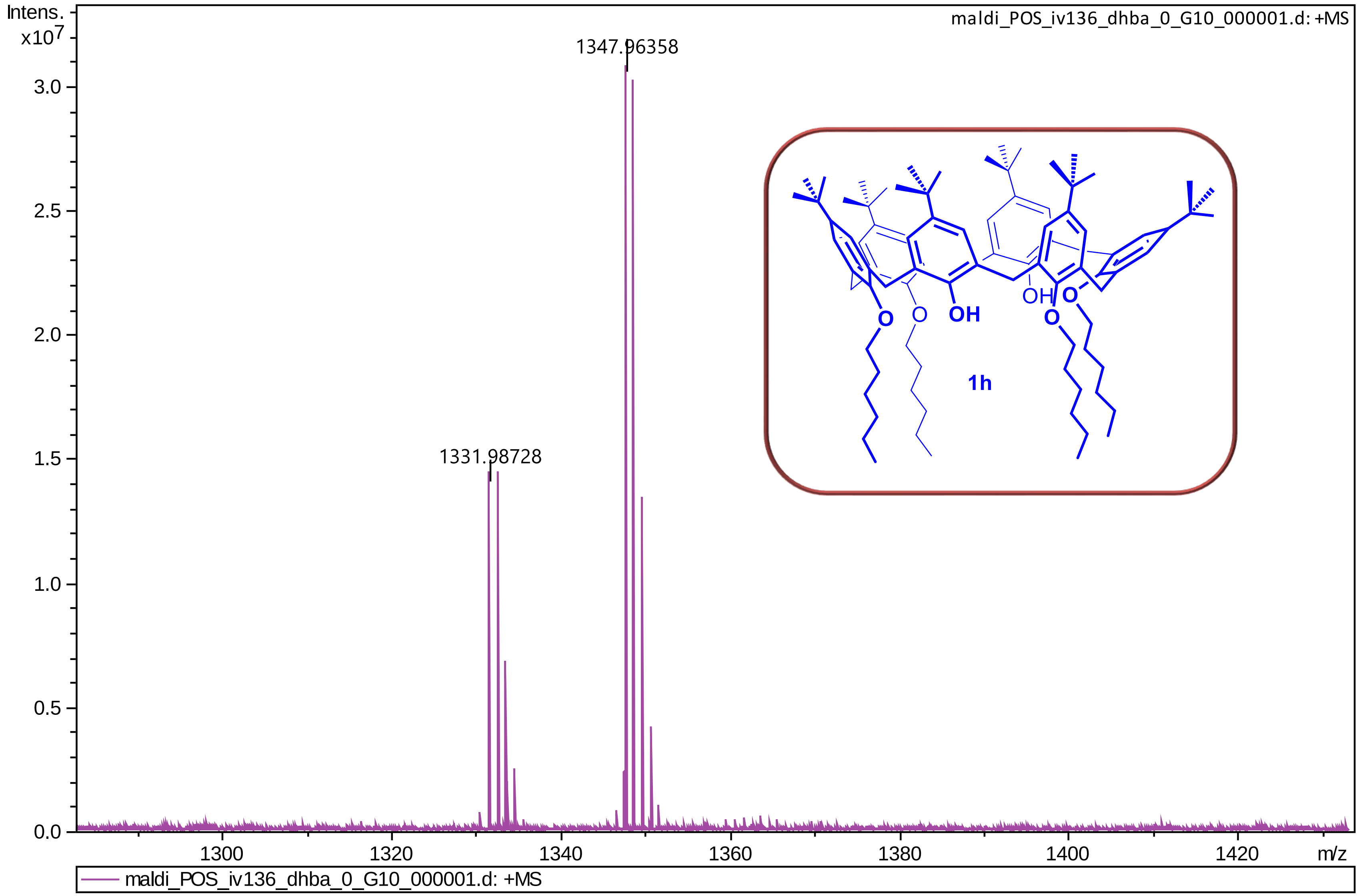

Figure S19. HR-MS spectrum of derivative **1h**.

LT NMR Spectra of derivative 1h

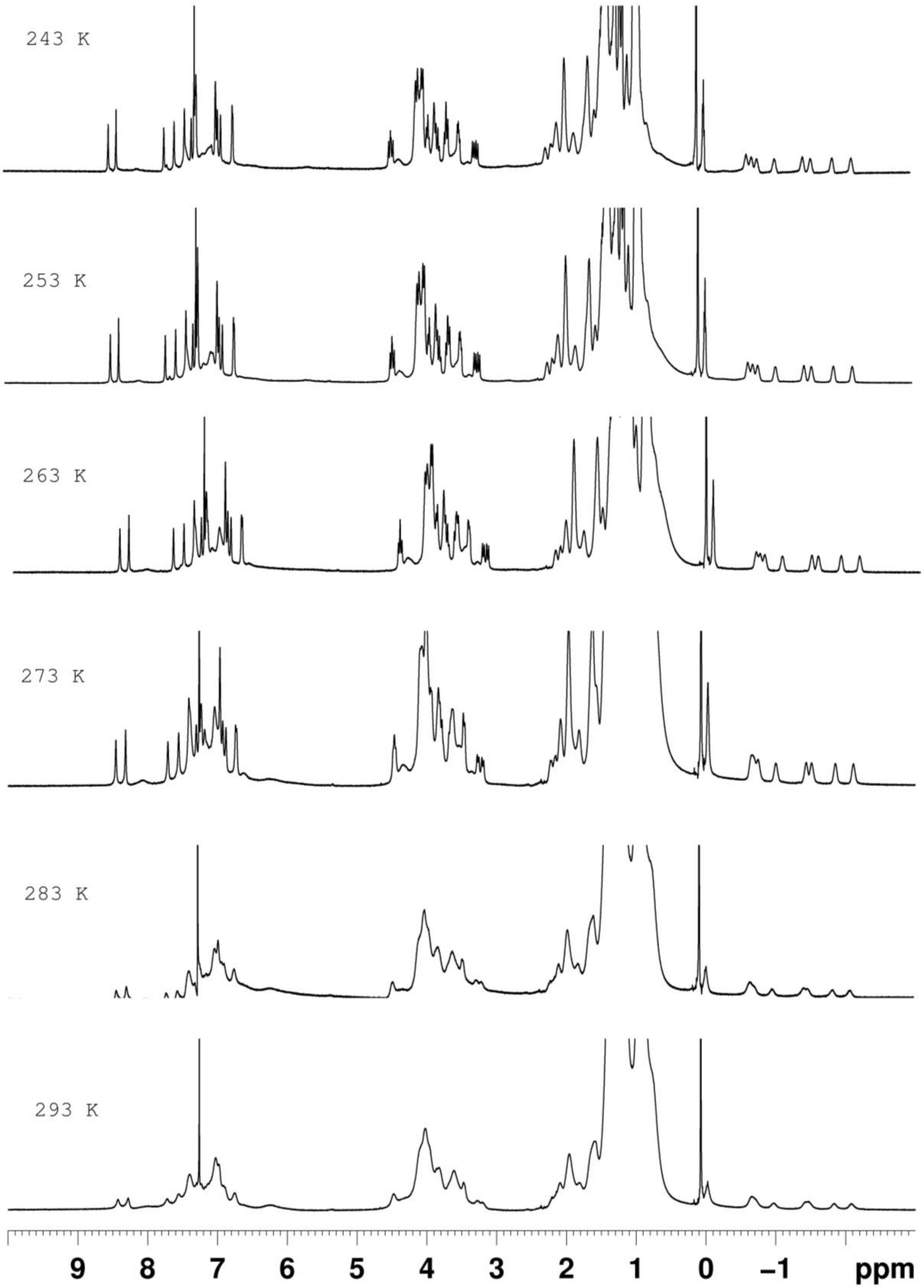

Figure S20. ^{1}H NMR spectrum of derivative **1h** (600 MHz, $CDCl_3$).

2D COSY Spectrum of derivative 1h

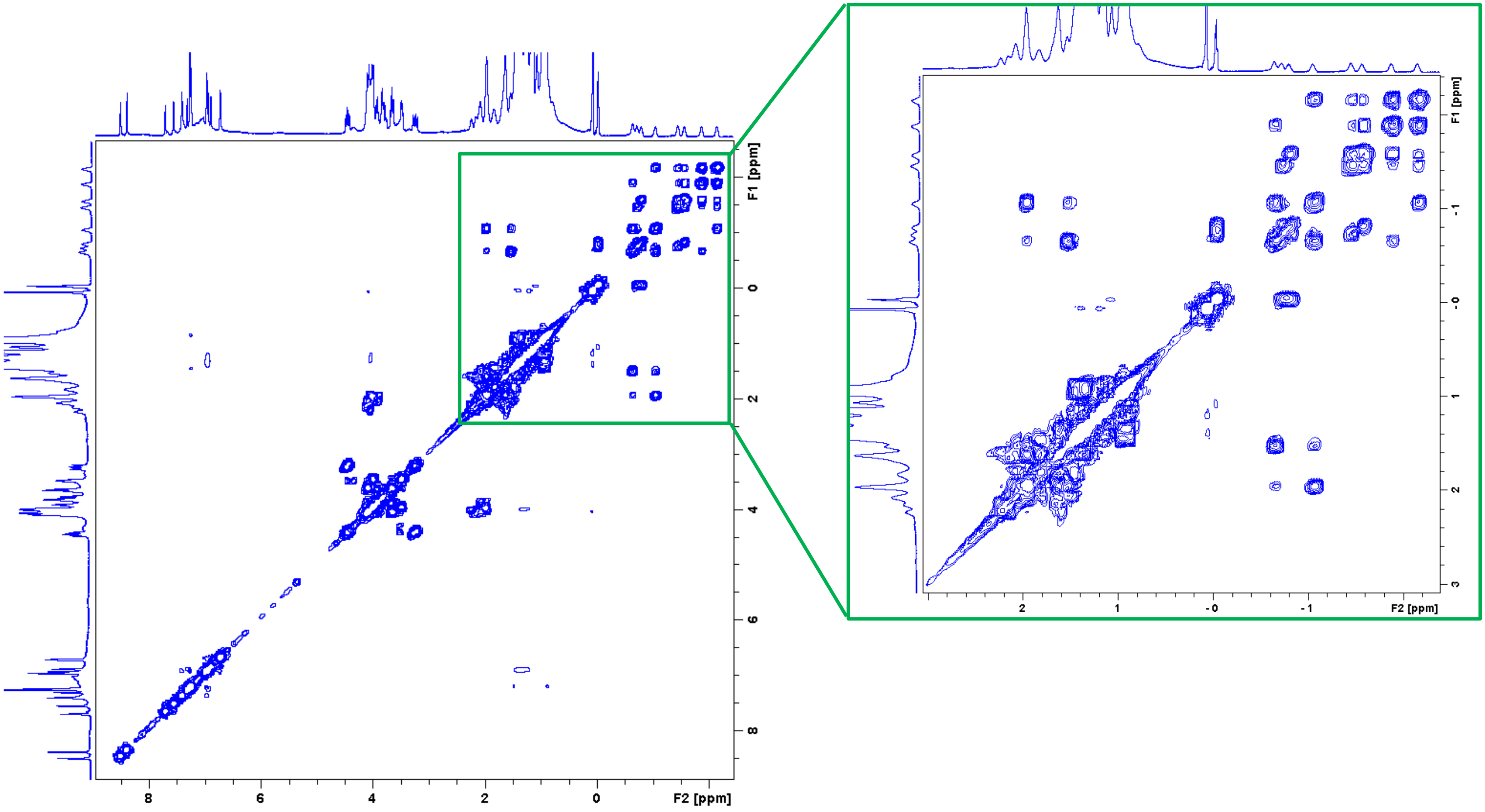

Figure S21. 2D COSY spectrum of derivative **1h** (600 MHz, $CDCl_3$, 243 K).

2D HSQC NMR Spectrum of derivative 1h

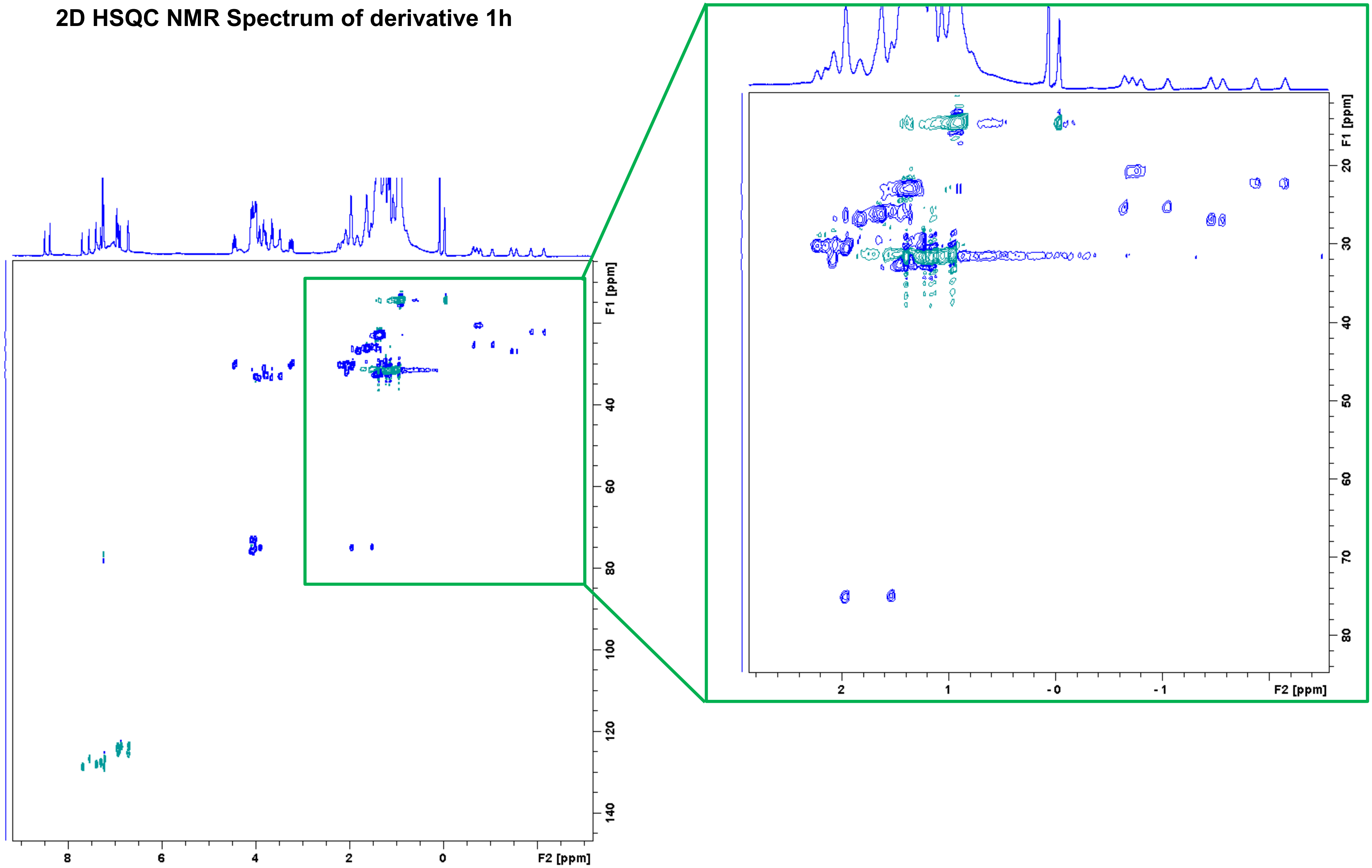

Figure S22. 2D HSQC spectrum of derivative **1h** (600 MHz, $CDCl_3$, 243 K).

Synthesis of pseudo[2]rotaxanes

A 1:1 mixture of calix[6]arene derivative and $[B(Ar^F)_4]^-$ linear system was dissolved in 0.5 mL of $CDCl_3$.In detail, in **Table S1** we reported concentration using for calix[6]arene derivative and for axles. Then, the solution was transferred in an NMR tube for 1D and 2D NMR spectra acquisition.

Table S1.

Pseudo[2]rotaxanes	Calix[6]arenes		Axles		$CDCl_3$
**4⁺⊂1b**	4.02 mg	$2.7 \cdot 10^{-3}$ mmol	2.78 mg	$2.7 \cdot 10^{-3}$ mmol	0.5 mL
**2⁺⊂1f**	4.01 mg	$2.6 \cdot 10^{-3}$ mmol	2.78 mg	$2.6 \cdot 10^{-3}$ mmol	0.5 mL
**3⁺⊂1f**	4.00 mg	$2.6 \cdot 10^{-3}$ mmol	2.68 mg	$2.6 \cdot 10^{-3}$ mmol	0.5 mL
**4⁺⊂1f**	4.01 mg	$2.6 \cdot 10^{-3}$ mmol	2.70 mg	$2.6 \cdot 10^{-3}$ mmol	0.5 mL
**2⁺⊂1g**	4.00 mg	$2.2 \cdot 10^{-3}$ mmol	2.38 mg	$2.2 \cdot 10^{-3}$ mmol	0.5 mL
**3⁺⊂1g**	4.02 mg	$2.2 \cdot 10^{-3}$ mmol	2.30 mg	$2.2 \cdot 10^{-3}$ mmol	0.5 mL
**4⁺⊂1g**	4.01 mg	$2.2 \cdot 10^{-3}$ mmol	2.32 mg	$2.2 \cdot 10^{-3}$ mmol	0.5 mL
**2⁺⊂1h**	4.02 mg	$3.0 \cdot 10^{-3}$ mmol	3.24 mg	$3.0 \cdot 10^{-3}$ mmol	0.5 mL
**3⁺⊂1h**	4.01 mg	$3.0 \cdot 10^{-3}$ mmol	3.12 mg	$3.0 \cdot 10^{-3}$ mmol	0.5 mL
**4⁺⊂1h**	4.03 mg	$3.0 \cdot 10^{-3}$ mmol	3.14 mg	$3.0 \cdot 10^{-3}$ mmol	0.5 mL

^{1}H NMR Spectrum of Derivative $4^+ \subset 1b$

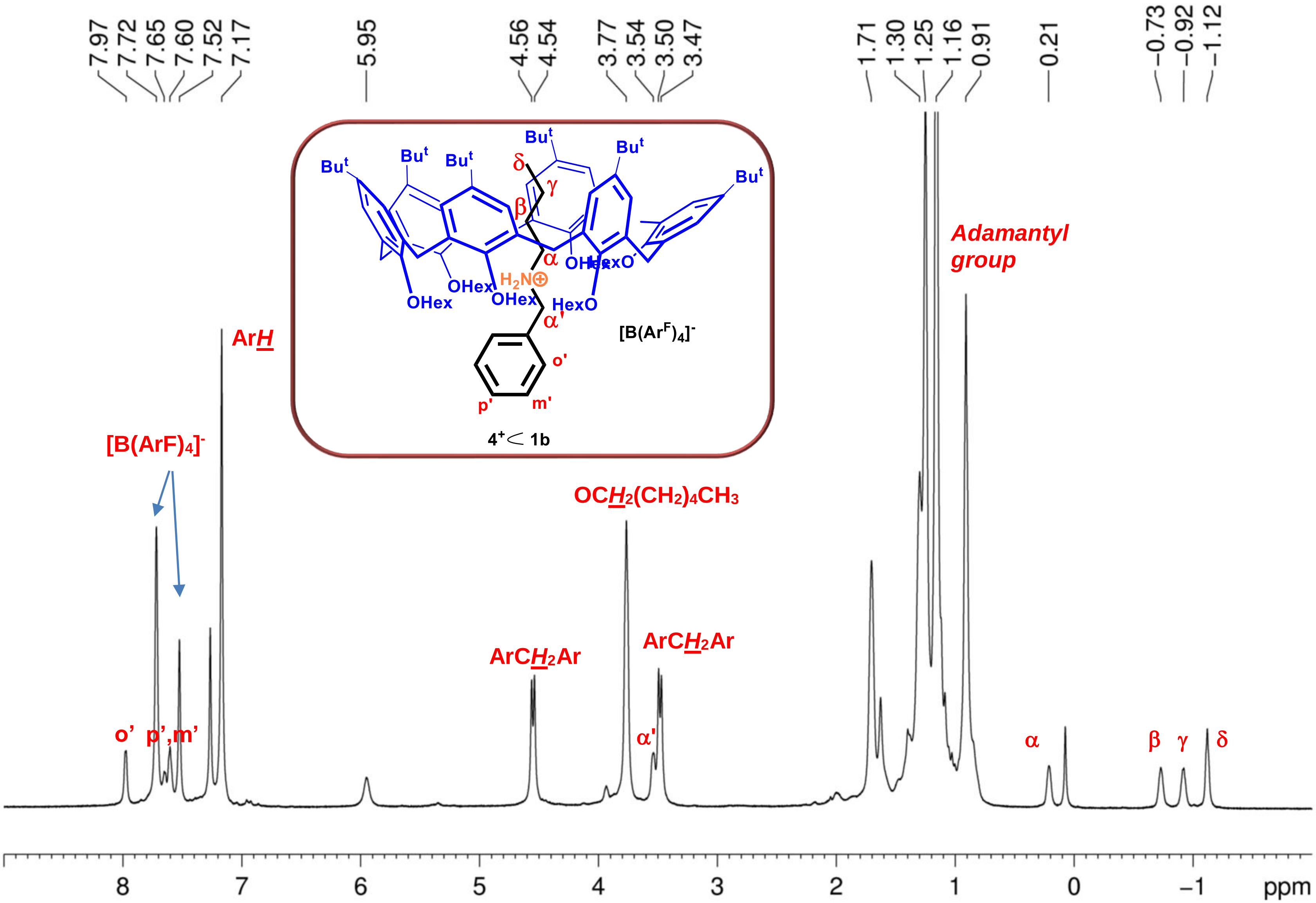

Figure S23. ^{1}H NMR spectrum of derivative **$4^+ \subset$ 1b** (600 MHz, $CDCl_3$, 298 K).

^{1}H NMR Spectrum of Derivative $2^{+}\subset$ 1f

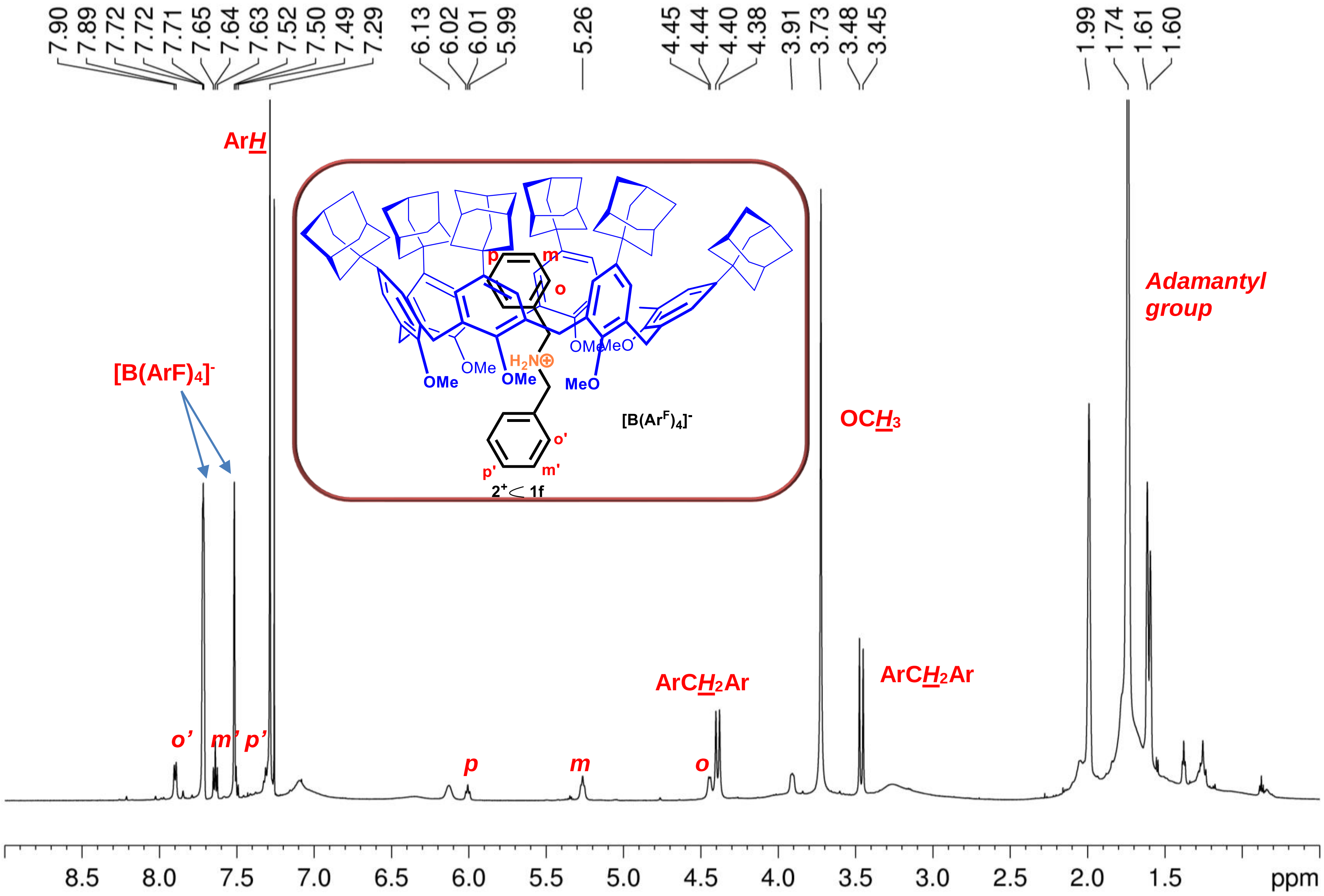

Figure S24. ^{1}H NMR spectrum of derivative **$2^{+}\subset$ 1f** (600 MHz, $CDCl_3$, 298 K).

2D COSY Spectrum of Derivative $2^+ \subset 1f$

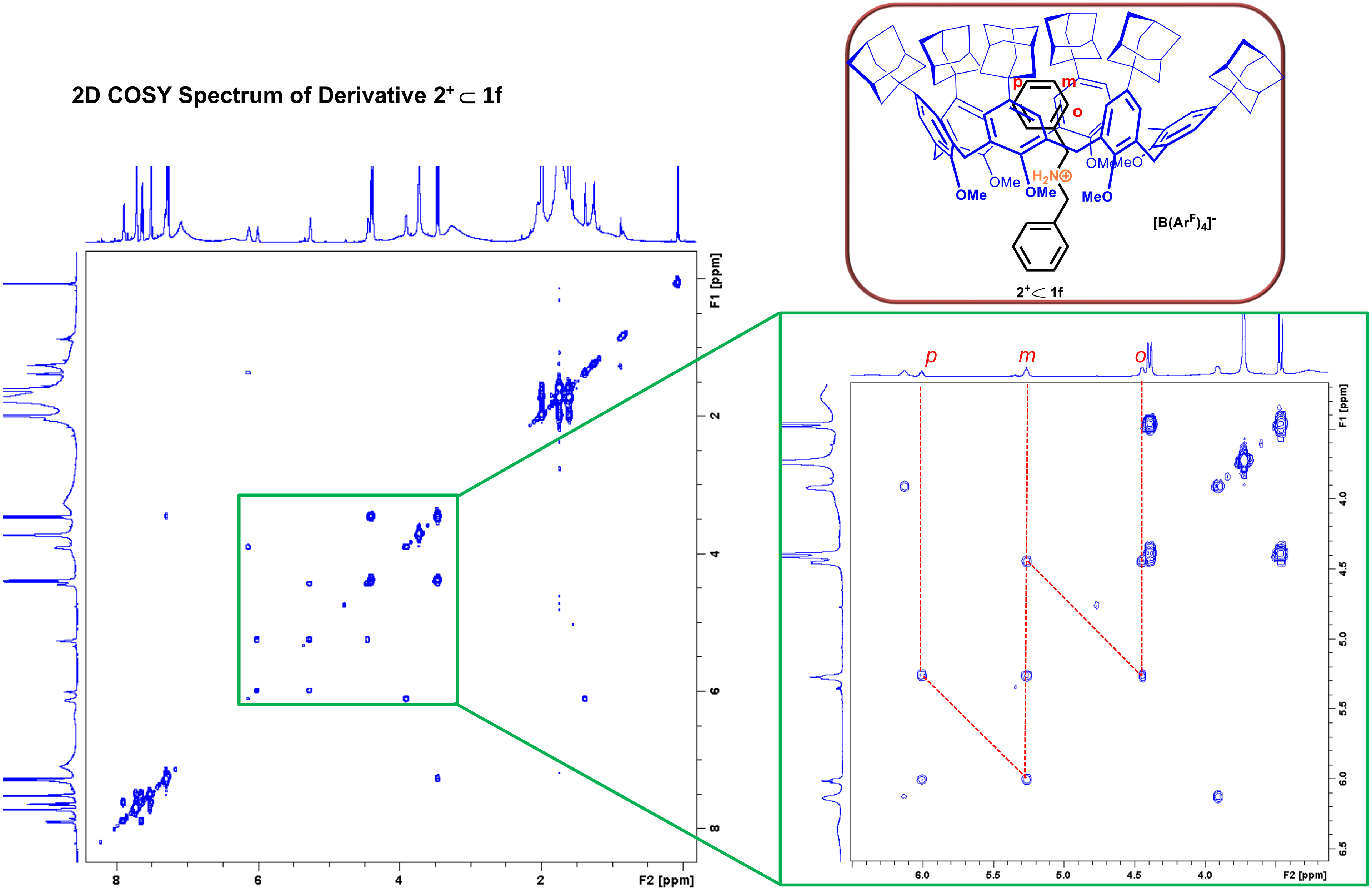

Figure S25. 2D COSY spectrum of derivative $\mathbf{2^+ \subset 1f}$ (600 MHz, $CDCl_3$, 298 K).

^{1}H NMR Spectrum of Derivative $3^{+} \subset$ 1f

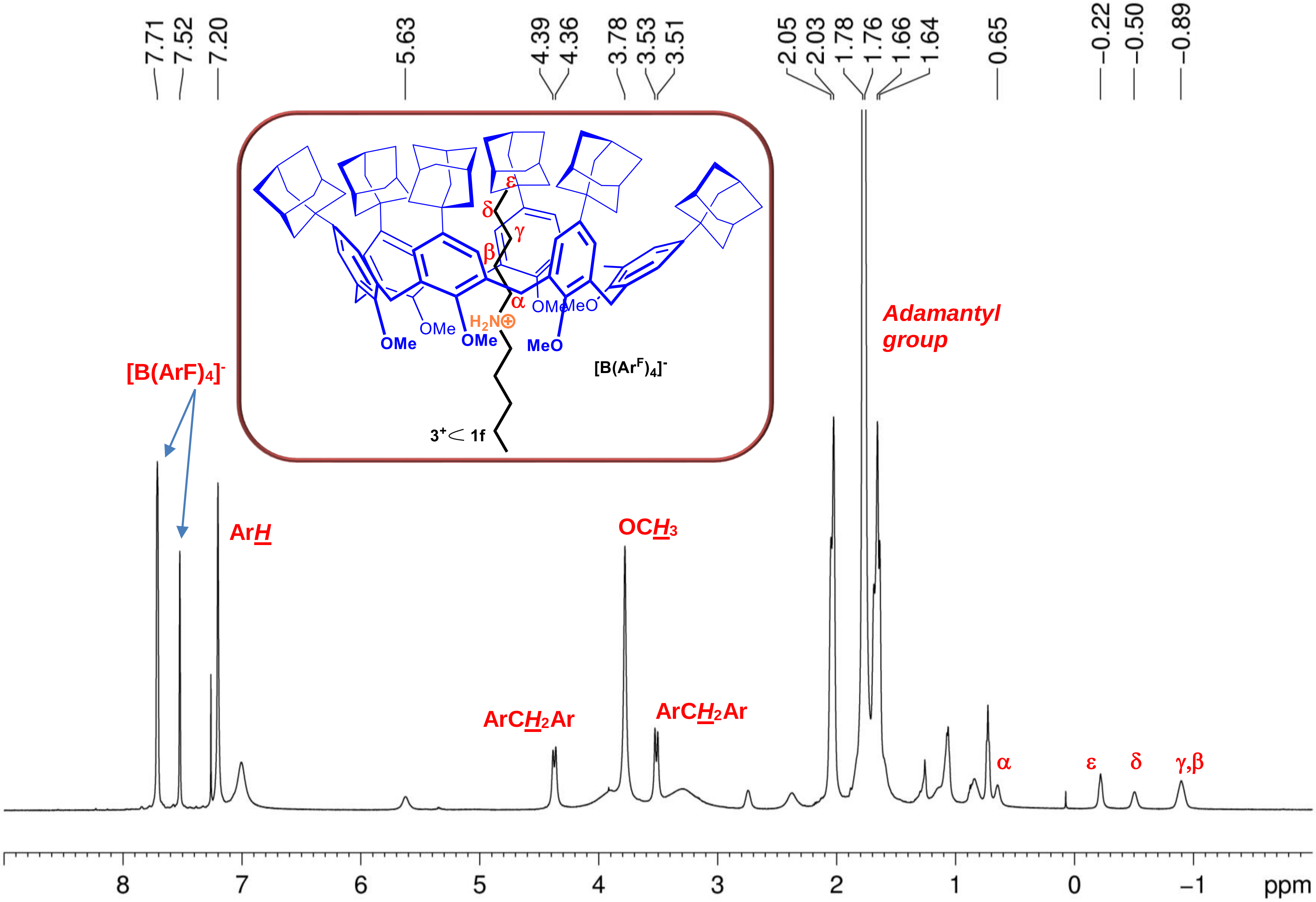

Figure S26. ^{1}H NMR spectrum of derivative **$3^{+} \subset$ 1f** (600 MHz, $CDCl_3$, 298 K).

2D COSY Spectrum of Derivative $3^+ \subset$ 1f

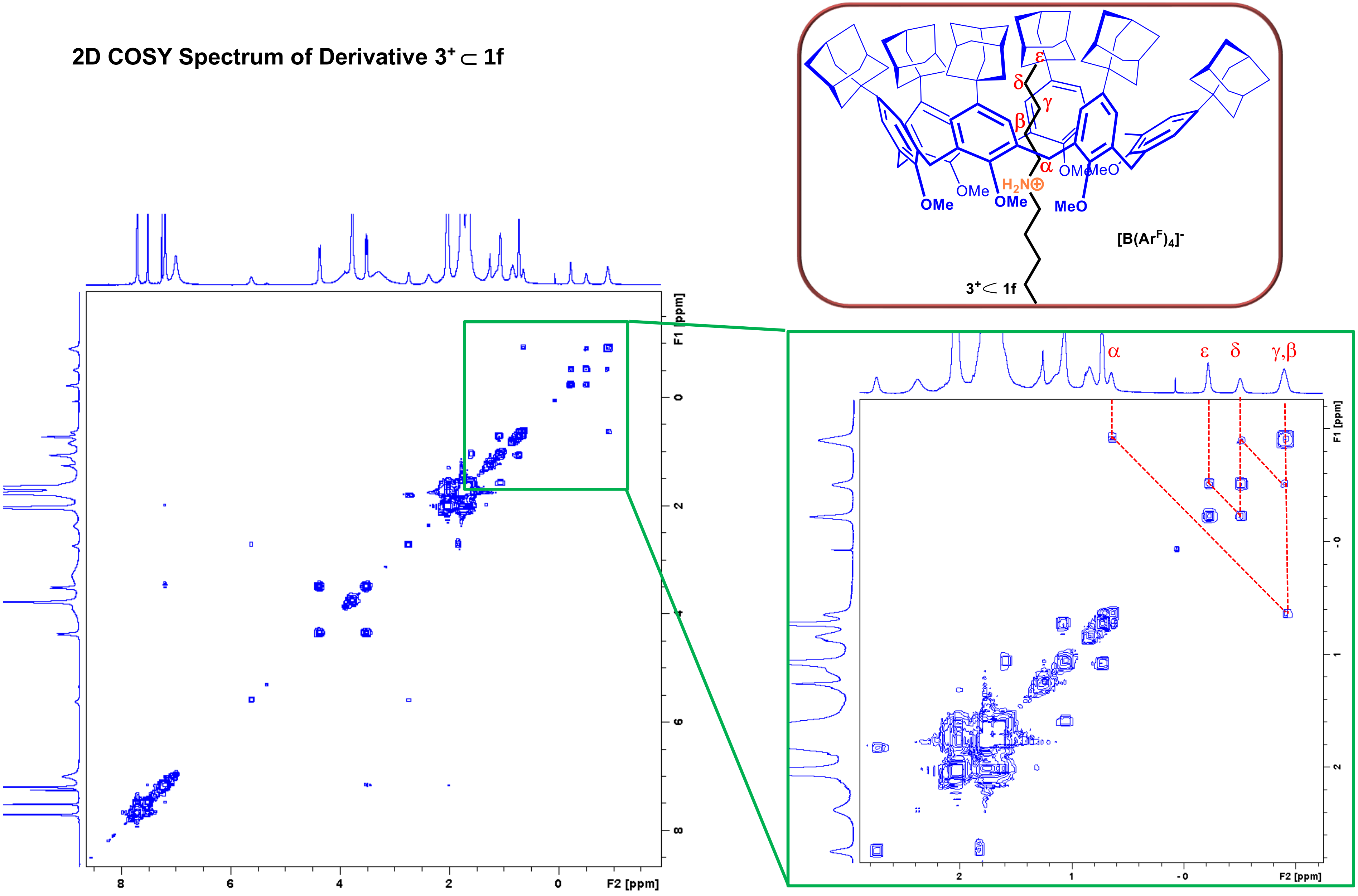

Figure S27. 2D COSY spectrum of derivative $\mathbf{3^+ \subset 1f}$ (600 MHz, $CDCl_3$, 298 K).

2D HSQC Spectrum of Derivative 3⁺⊂ 1f

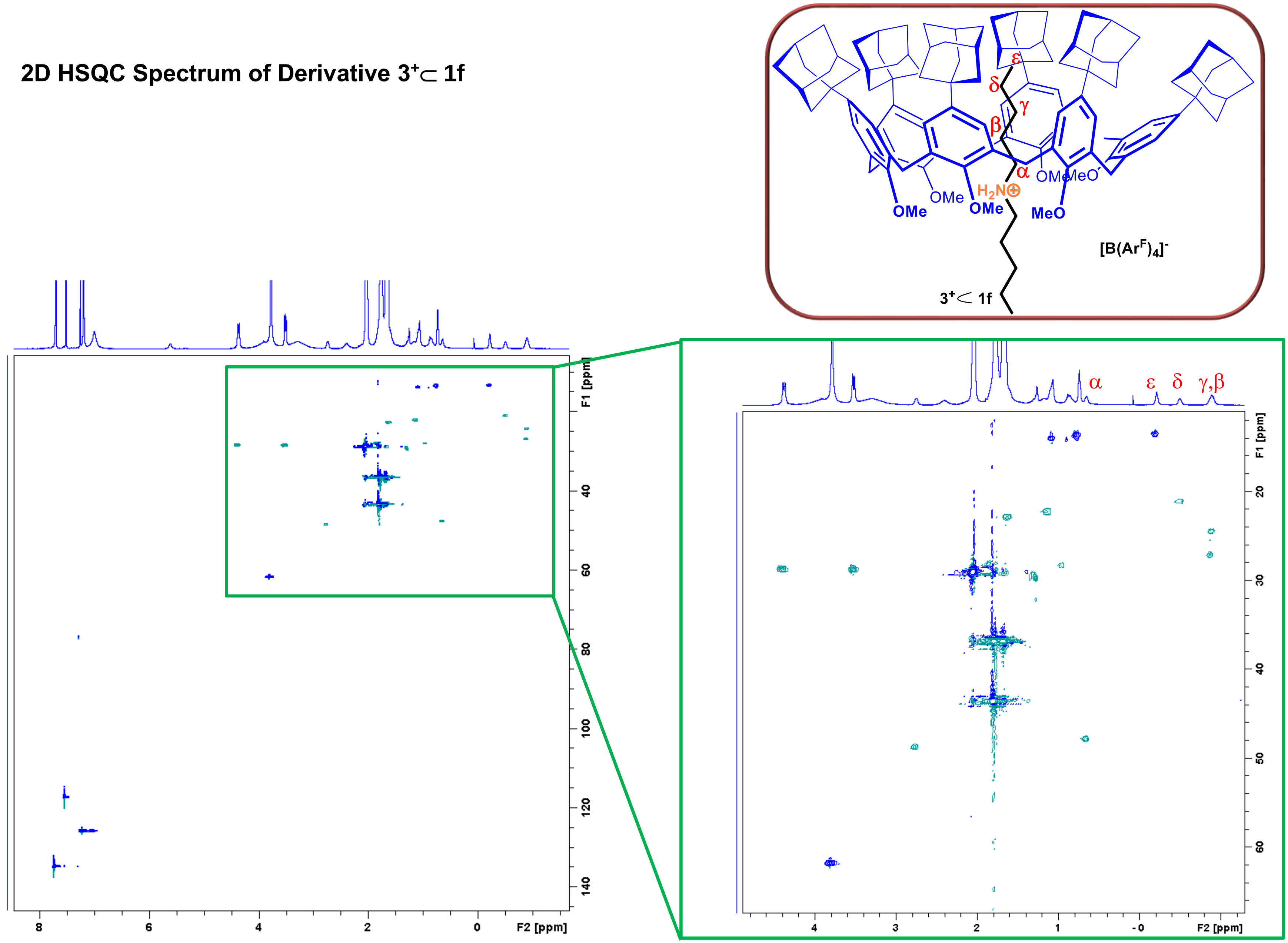

Figure S28. 2D HSQC spectrum of derivative **3⁺⊂ 1f** (600 MHz, $CDCl_3$, 298 K).

^{1}H NMR Spectrum of Derivative $4^+ \subset$ 1f

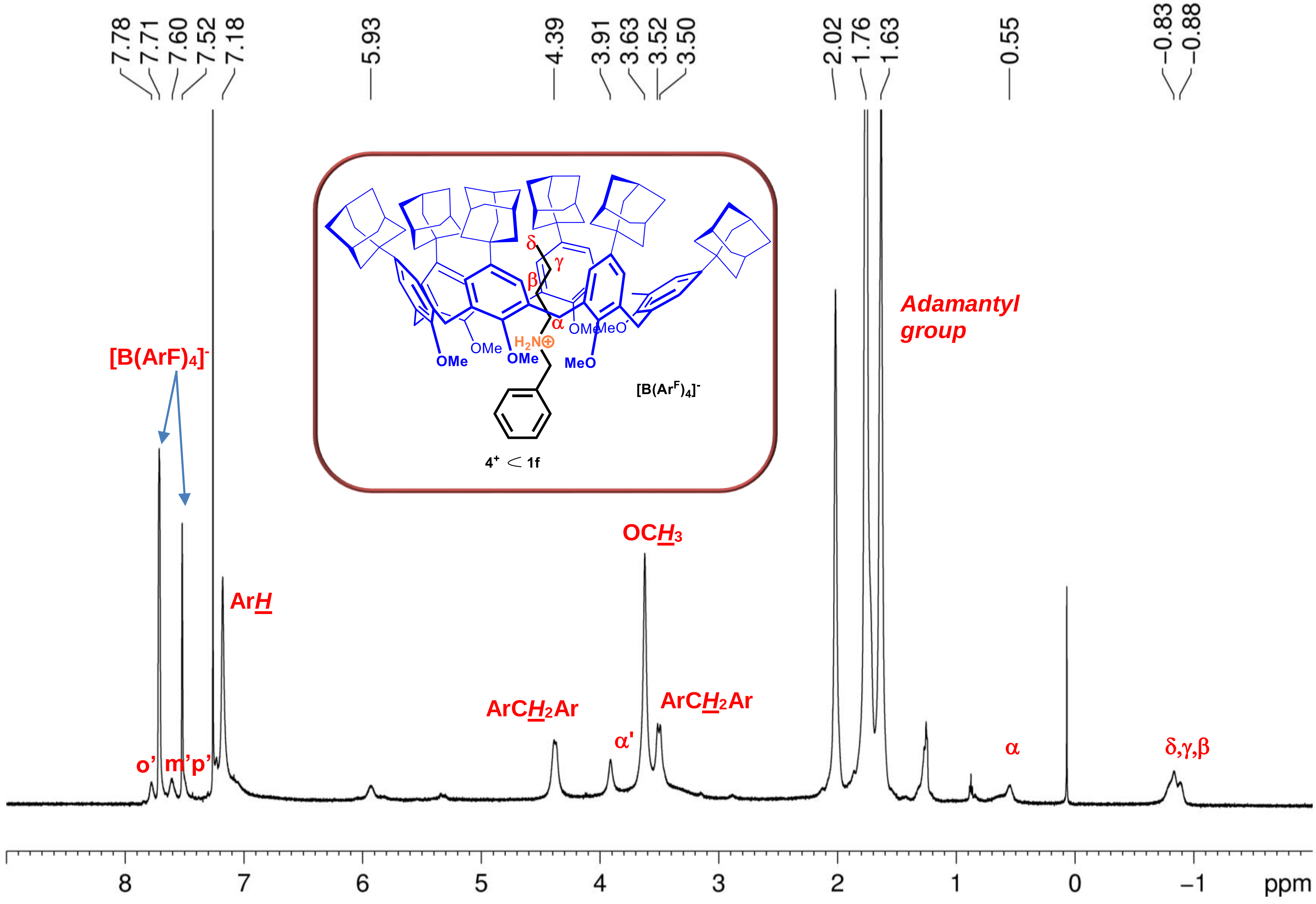

Figure S29. ^{1}H NMR spectrum of derivative **$4^+ \subset$ 1f** (600 MHz, $CDCl_3$, 298 K).

2D COSY Spectrum of Derivative $4^+ \subset 1f$

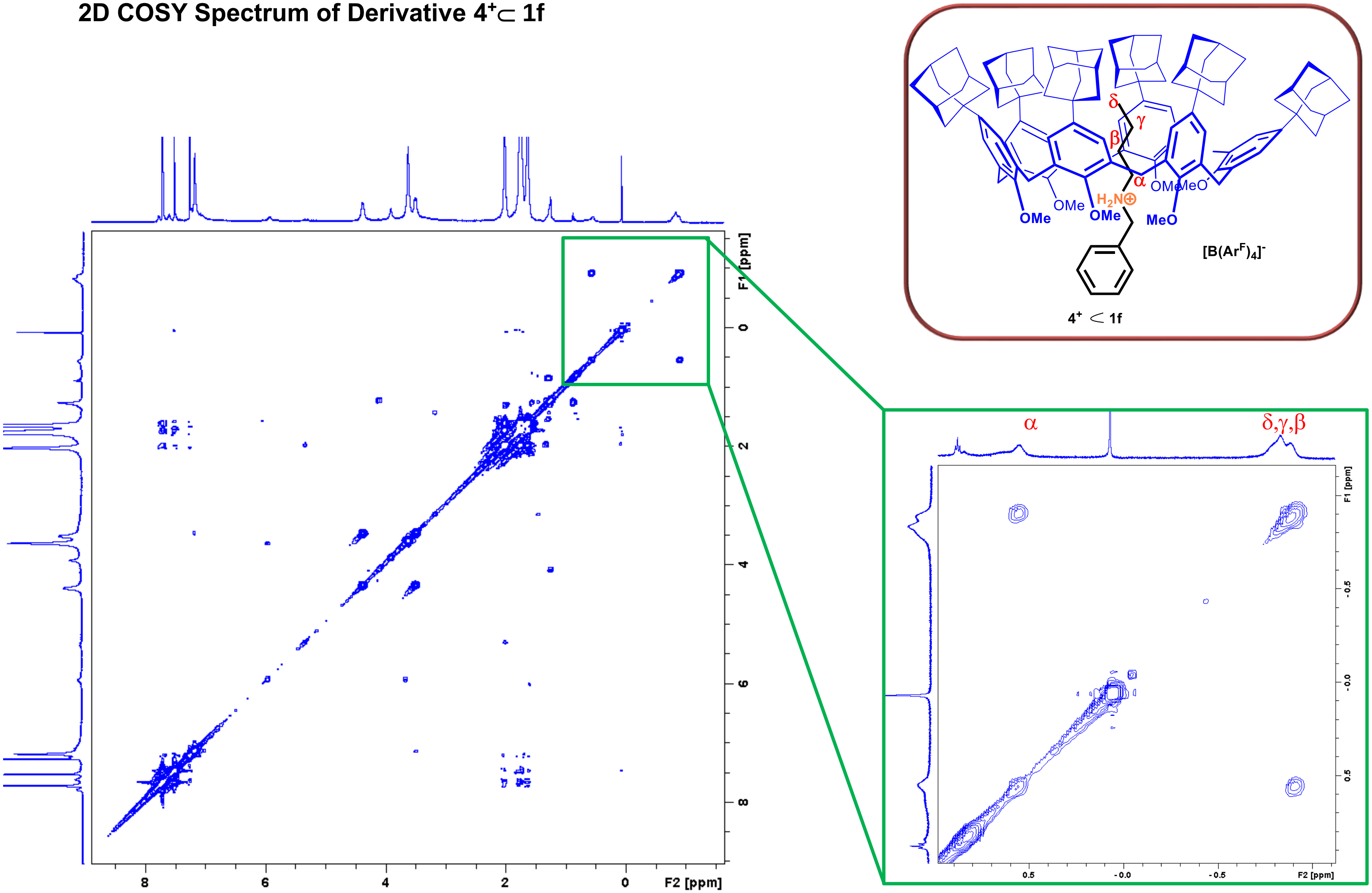

Figure S30. 2D COSY spectrum of derivative **$4^+ \subset 1f$** (600 MHz, $CDCl_3$, 298 K).

^{1}H NMR Spectrum of Derivative $2^+ \subset$ 1g

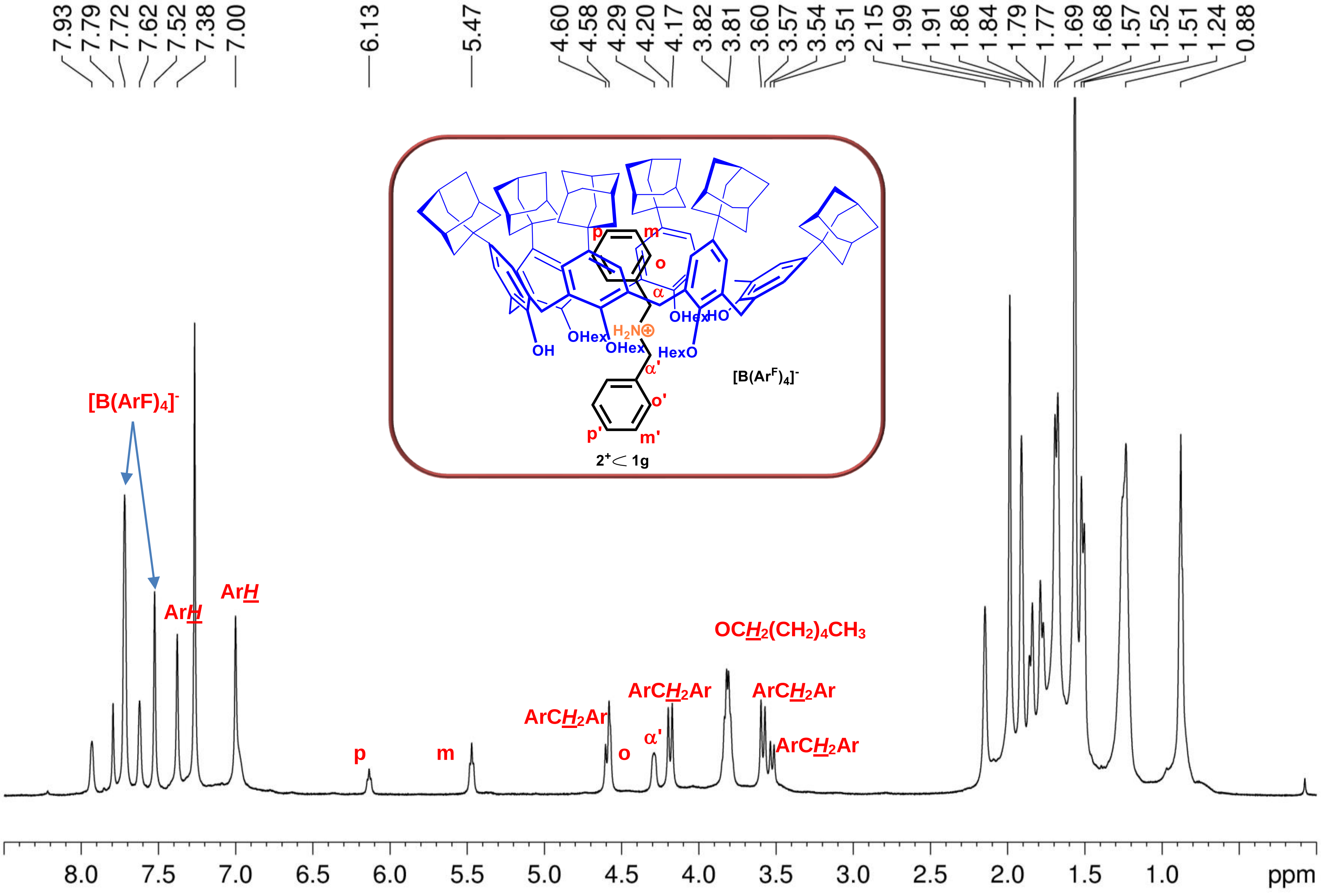

Figure S31. ^{1}H NMR spectrum of derivative **$2^+ \subset$ 1g** (600 MHz, $CDCl_3$, 298 K).

2D COSY Spectrum of Derivative $2^{+}\subset 1g$

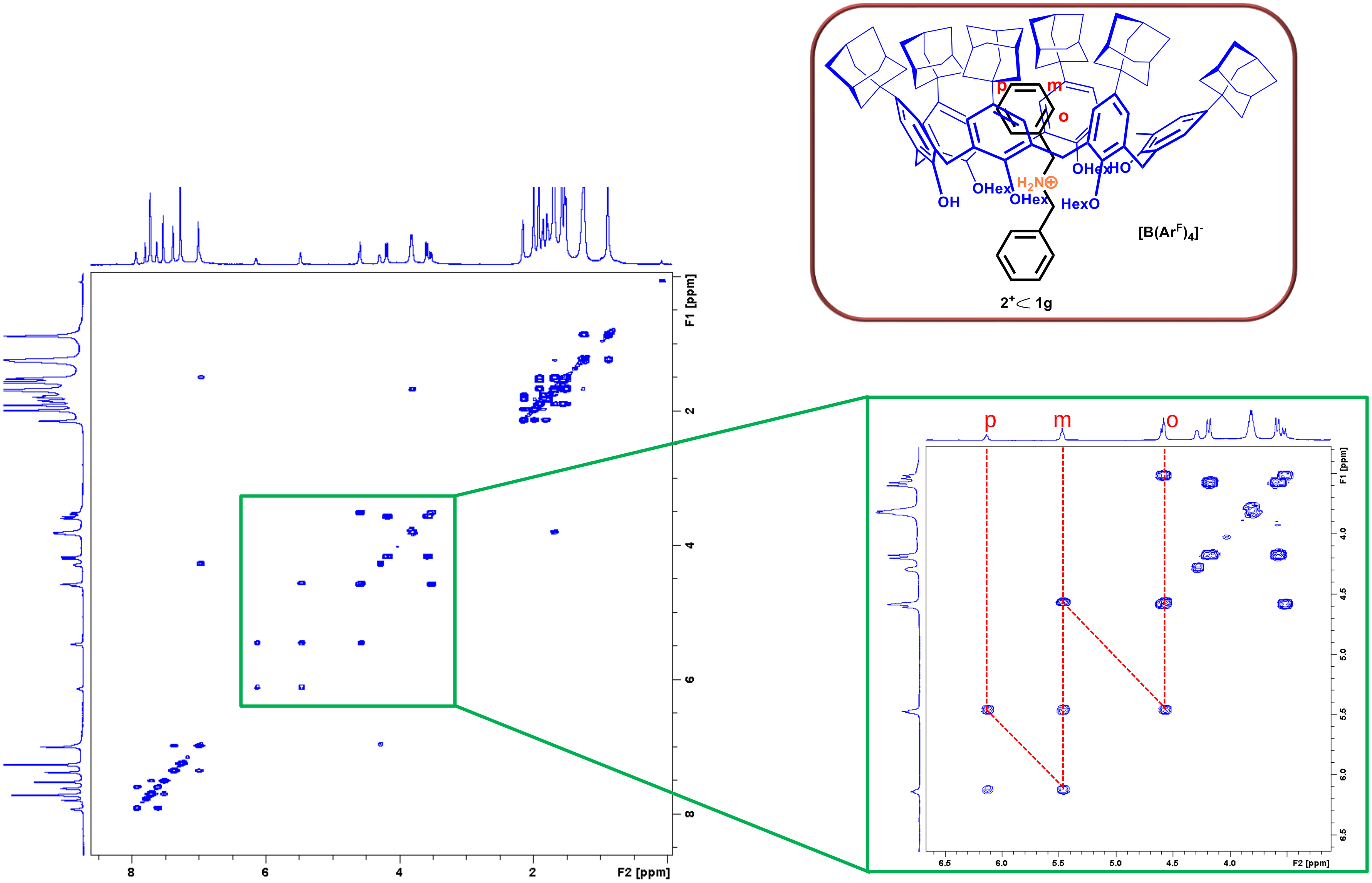

Figure S32. 2D COSY spectrum of derivative $\mathbf{2^{+}\subset 1g}$ (600 MHz, $CDCl_3$, 298 K).

2D HSQC Spectrum of Derivative 2$^+$⊂ 1g

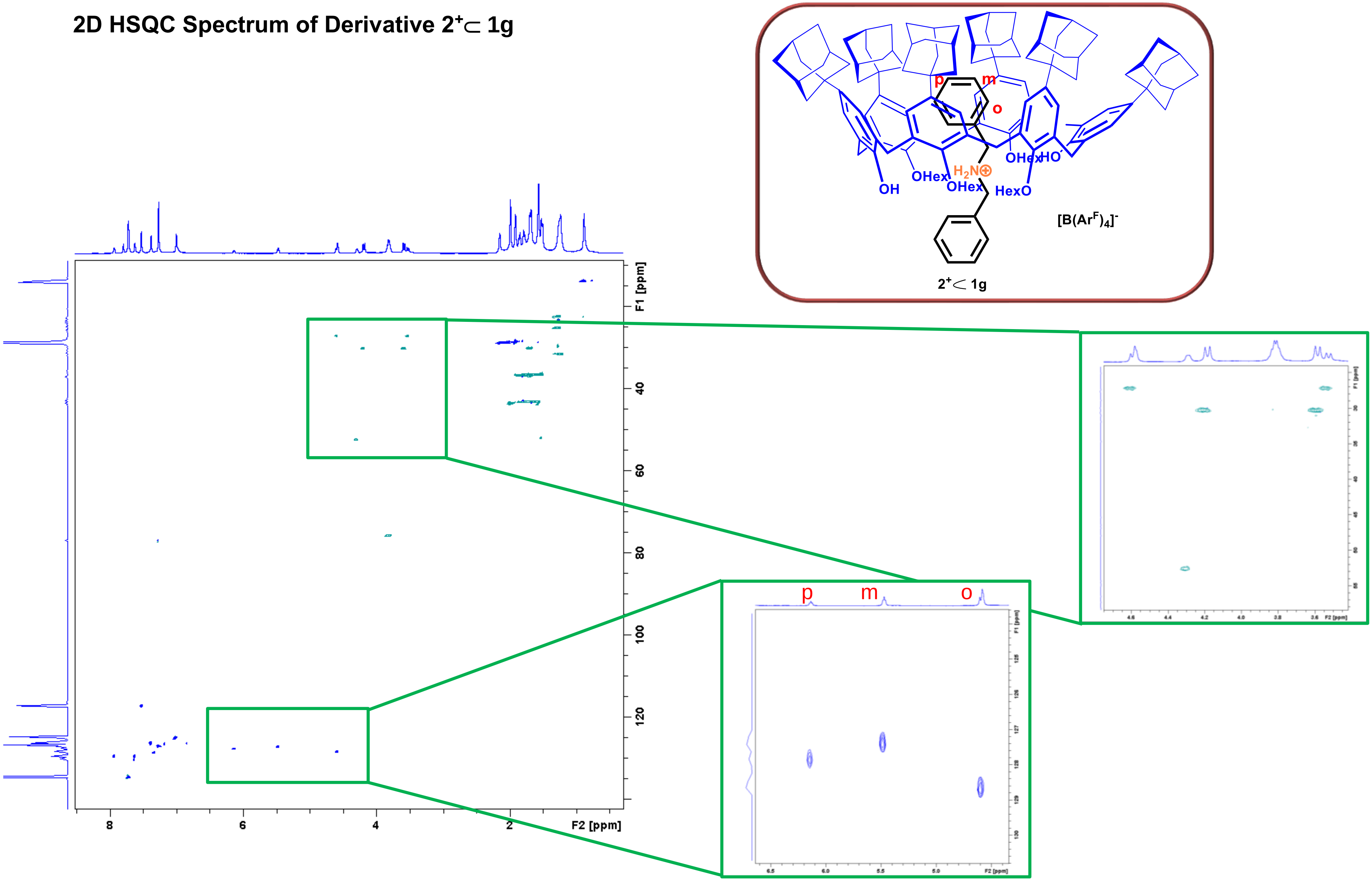

Figure S33. 2D HSQC spectrum of derivative **2$^+$⊂ 1g** (600 MHz, $CDCl_3$, 298 K).

^{1}H NMR Spectrum of Derivative 3⁺⊂ 1g

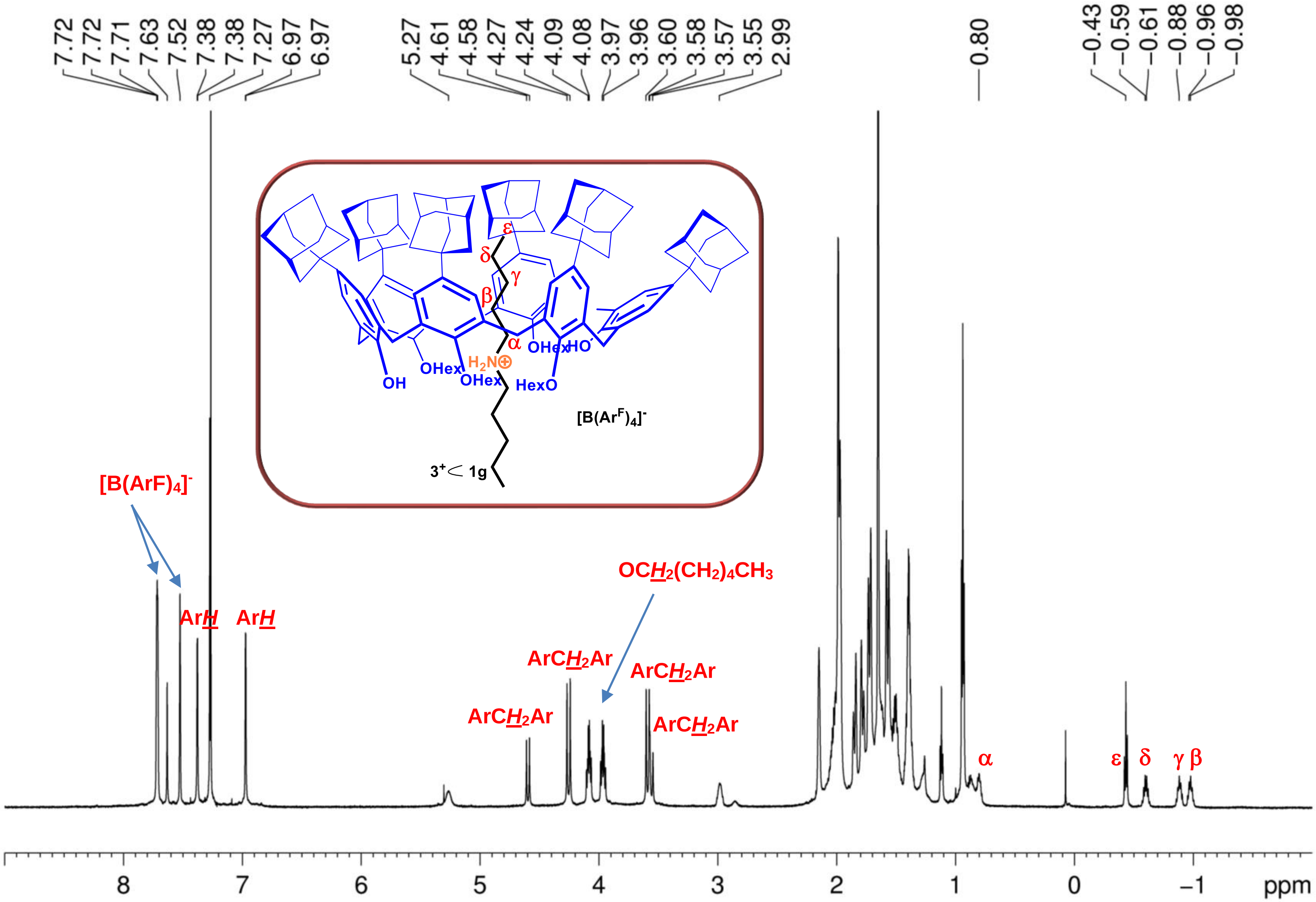

Figure S34. ^{1}H NMR spectrum of derivative **3⁺⊂ 1g** (600 MHz, $CDCl_3$, 298 K).

2D COSY Spectrum of Derivative $3^+ \subset 1g$

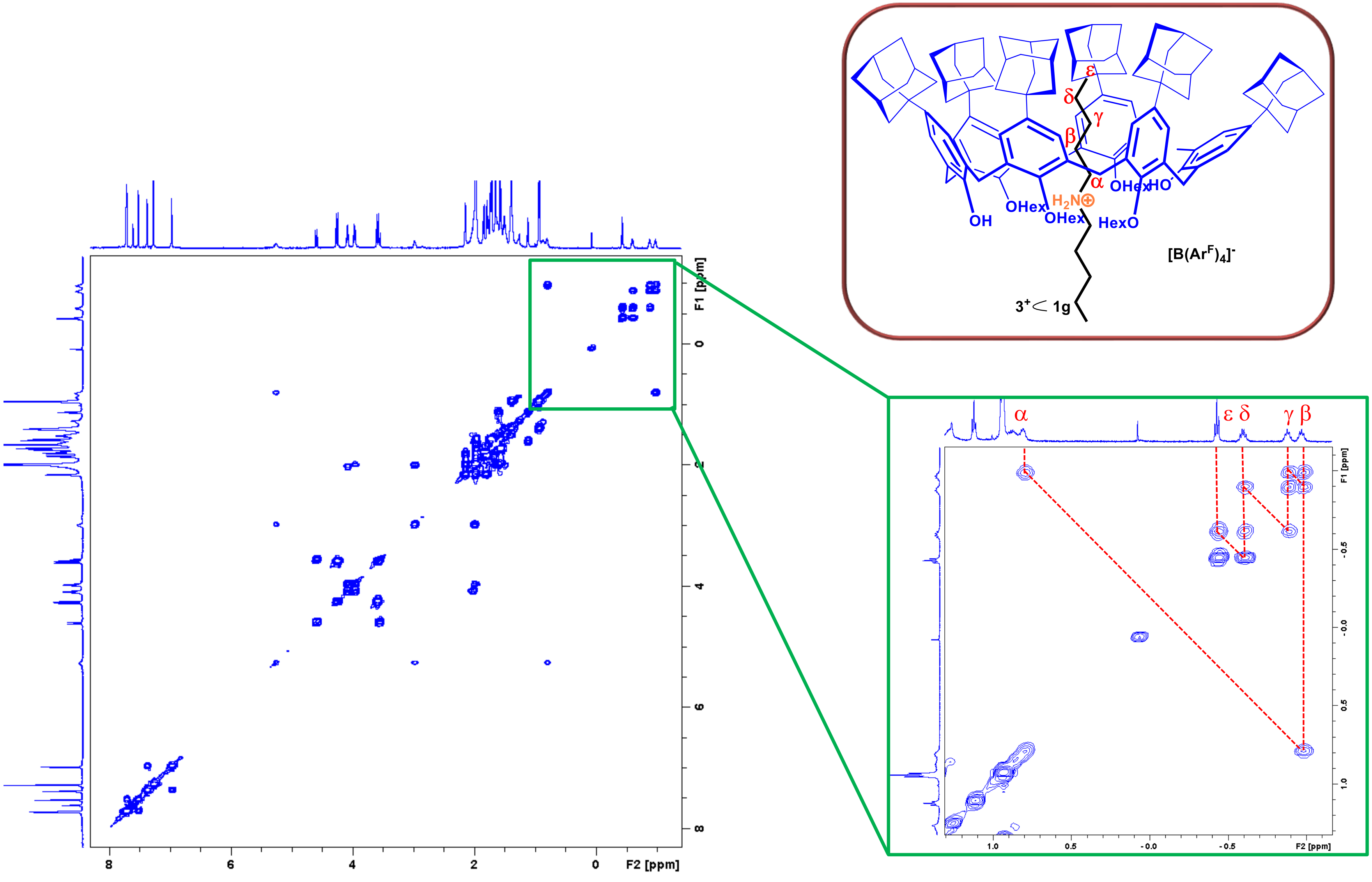

Figure S35. 2D COSY spectrum of derivative $\mathbf{3^+ \subset 1g}$ (600 MHz, $CDCl_3$, 298 K).

2D HSQC Spectrum of Derivative $3^{+}\subset 1g$

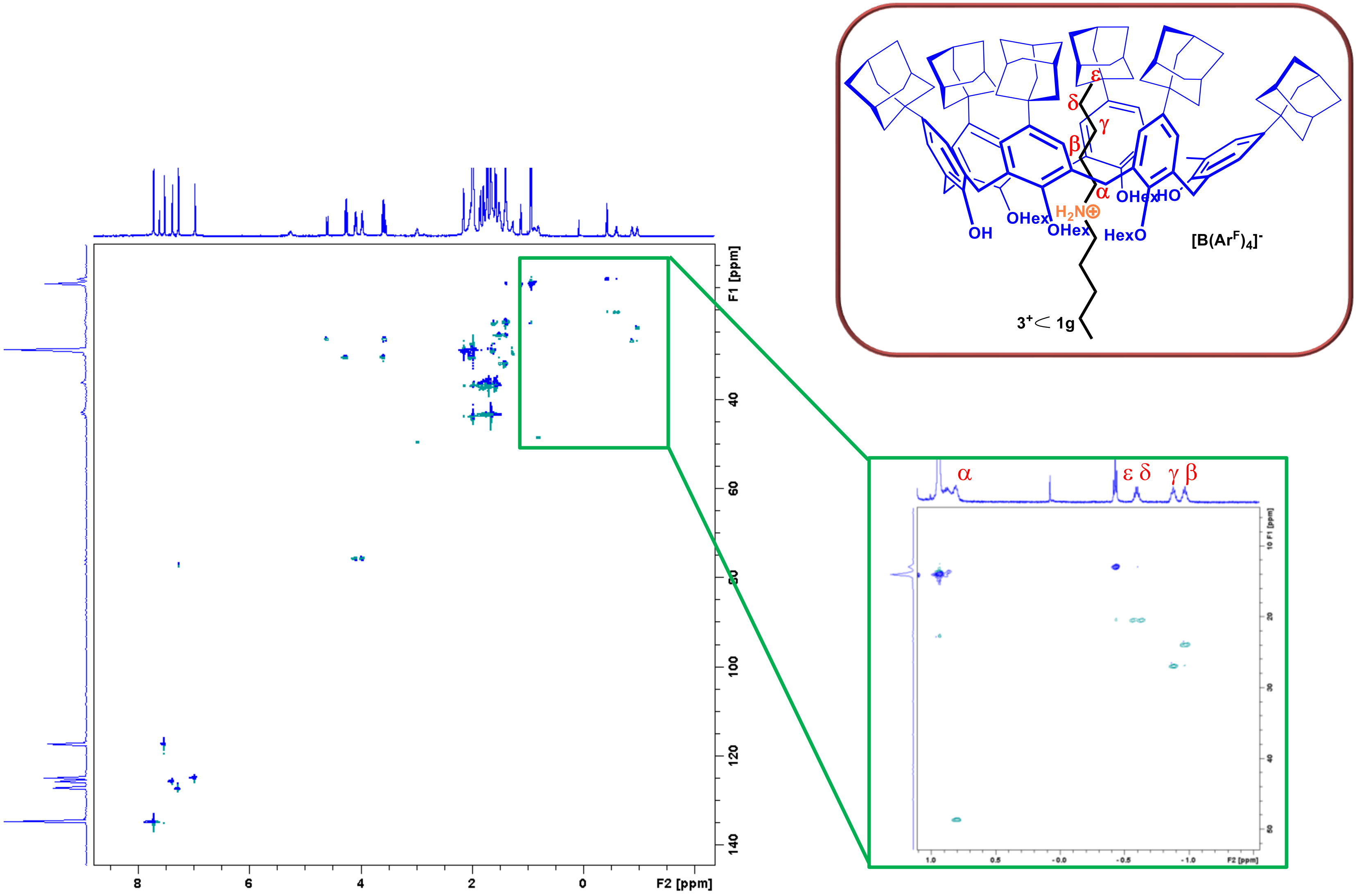

Figure S36. 2D HSQC spectrum of derivative **$3^{+}\subset 1g$** (600 MHz, $CDCl_3$, 298 K).

^{1}H NMR Spectrum of Derivative $4^+ \subset 1g$

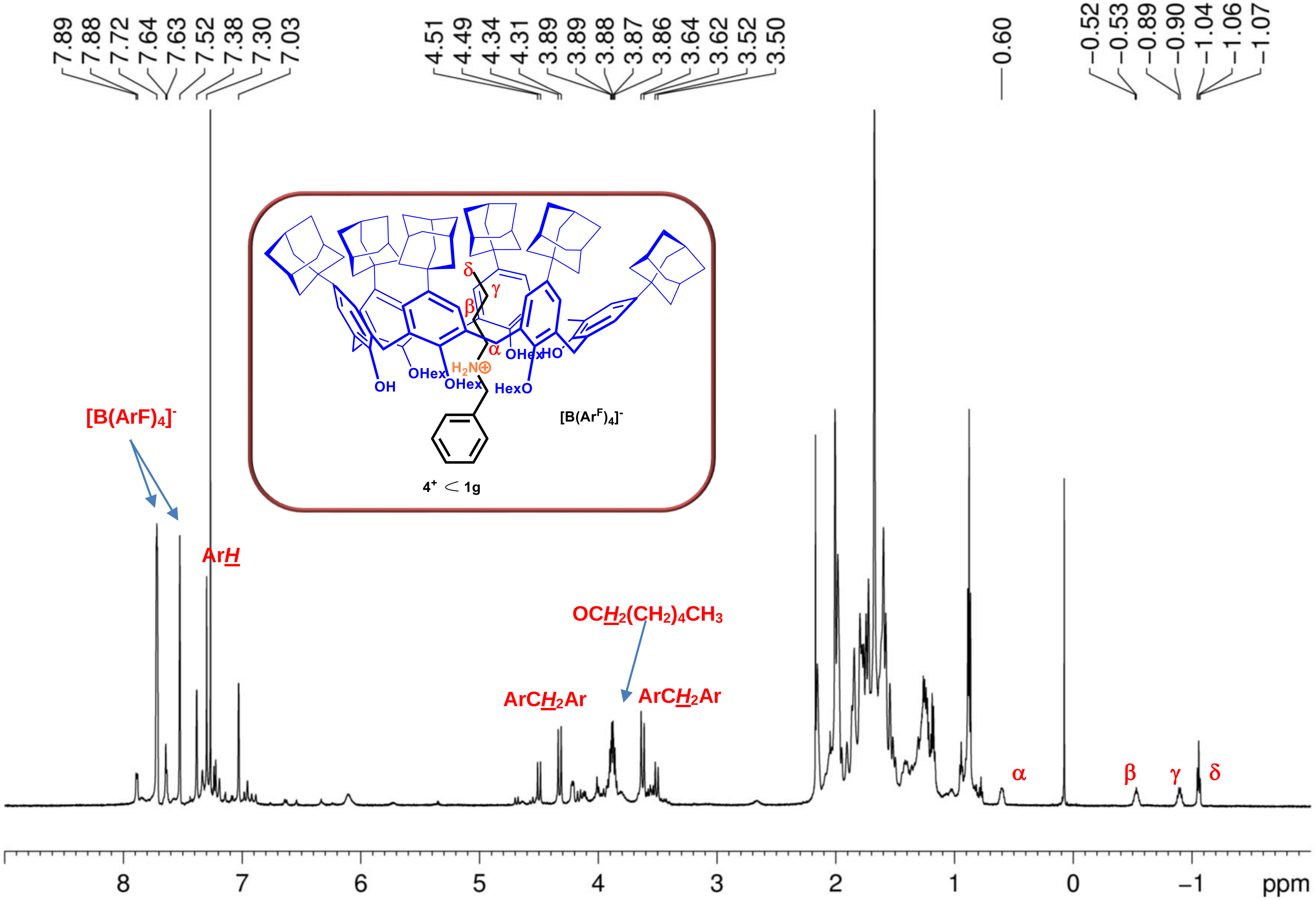

Figure S37. ^{1}H NMR spectrum of derivative $\mathbf{4^+ \subset 1g}$ (600 MHz, $CDCl_3$, 298 K).

2D COSY Spectrum of Derivative $4^{+}\subset 1g$

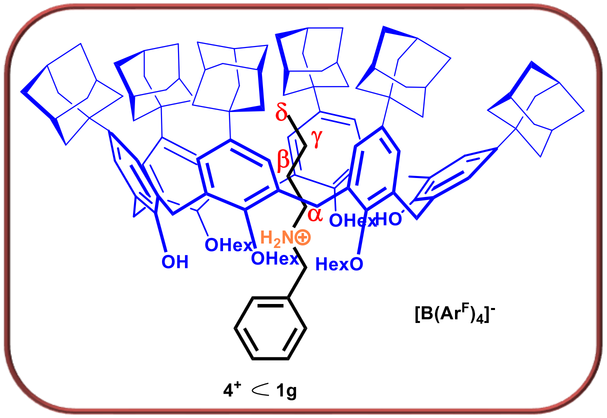

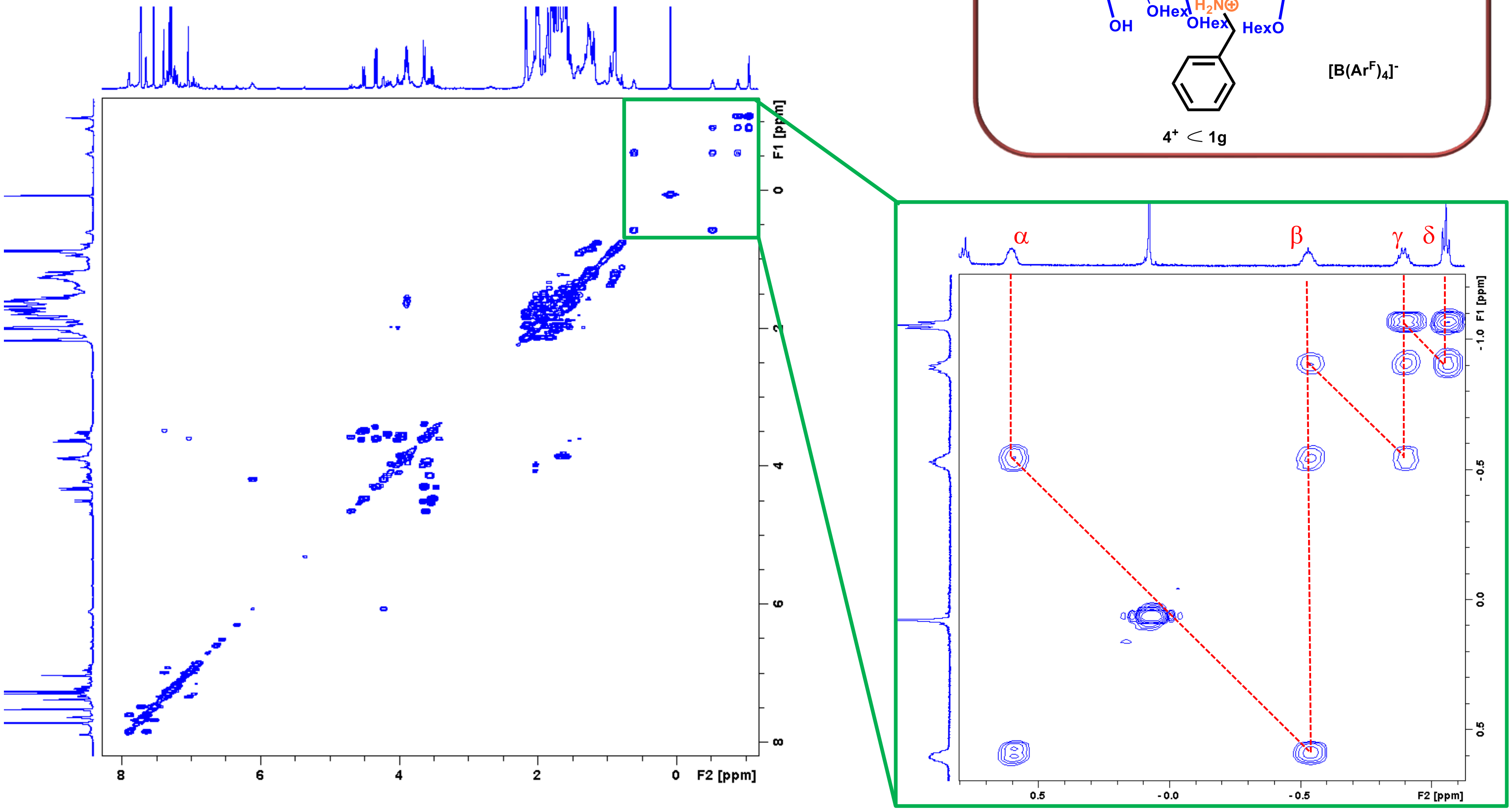

Figure S38. 2D COSY spectrum of derivative **$4^{+}\subset 1g$** (600 MHz, $CDCl_3$, 298 K).

2D HSQC Spectrum of Derivative $4^{+}\subset$ 1g

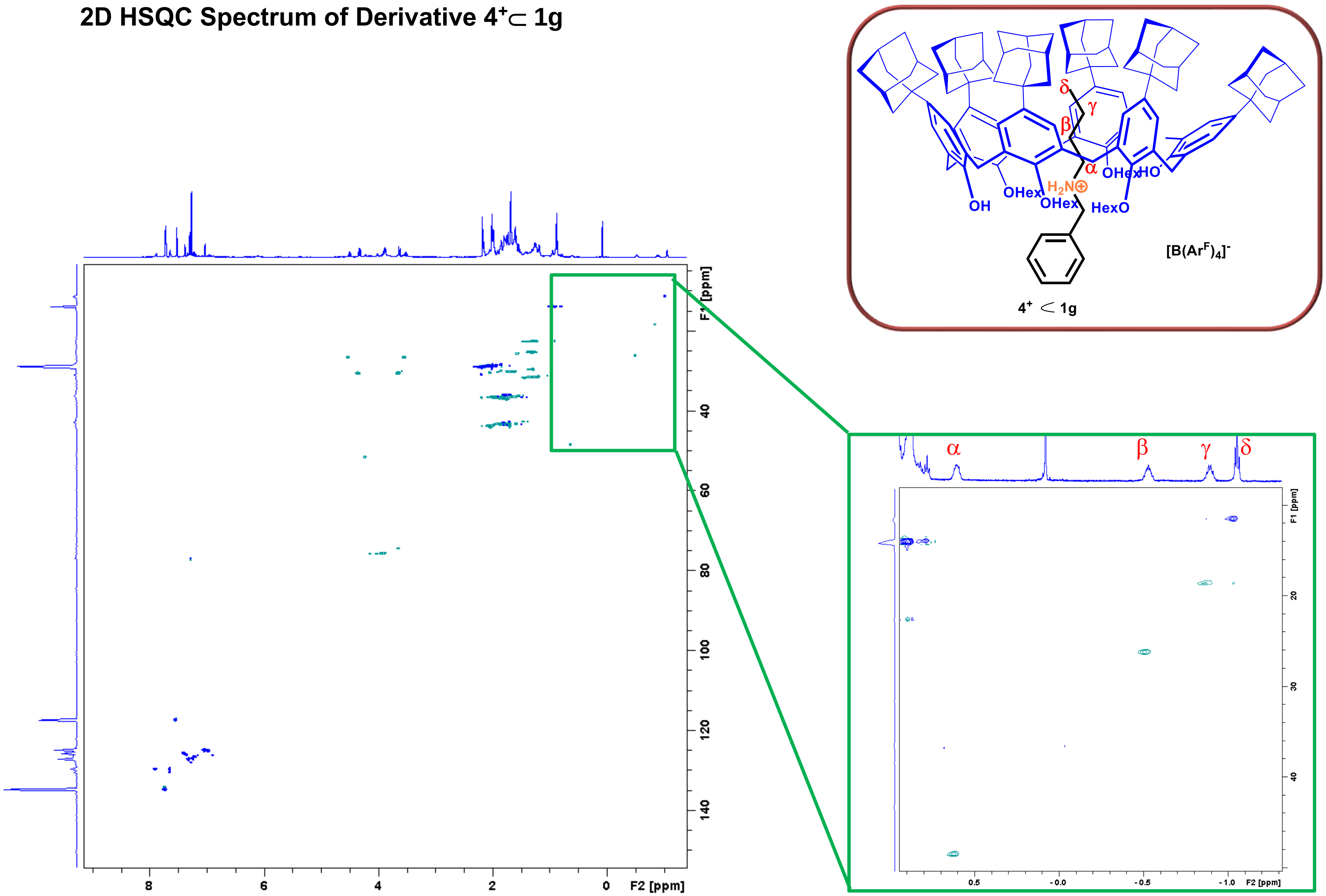

Figure S39. 2D HSQC spectrum of derivative $\mathbf{4^{+}\subset 1g}$ (600 MHz, $CDCl_3$, 298 K).

^{1}H NMR Spectrum of Derivative $2^+ \subset$ 1h

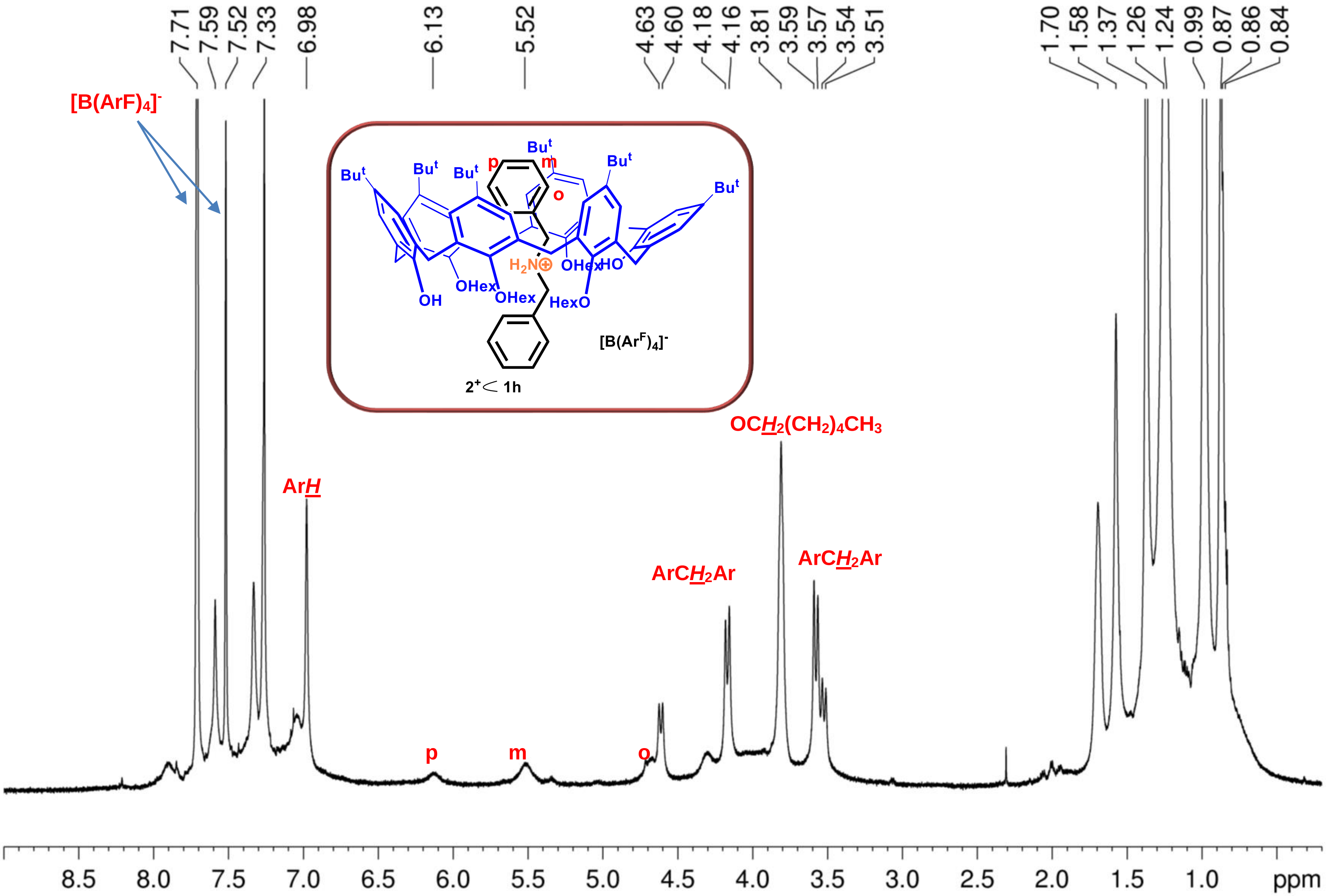

Figure S40. ^{1}H NMR spectrum of derivative **$2^+ \subset$ 1h** (600 MHz, $CDCl_3$, 298 K).

^{1}H NMR Spectrum of Derivative 3$^+$⊂ 1h

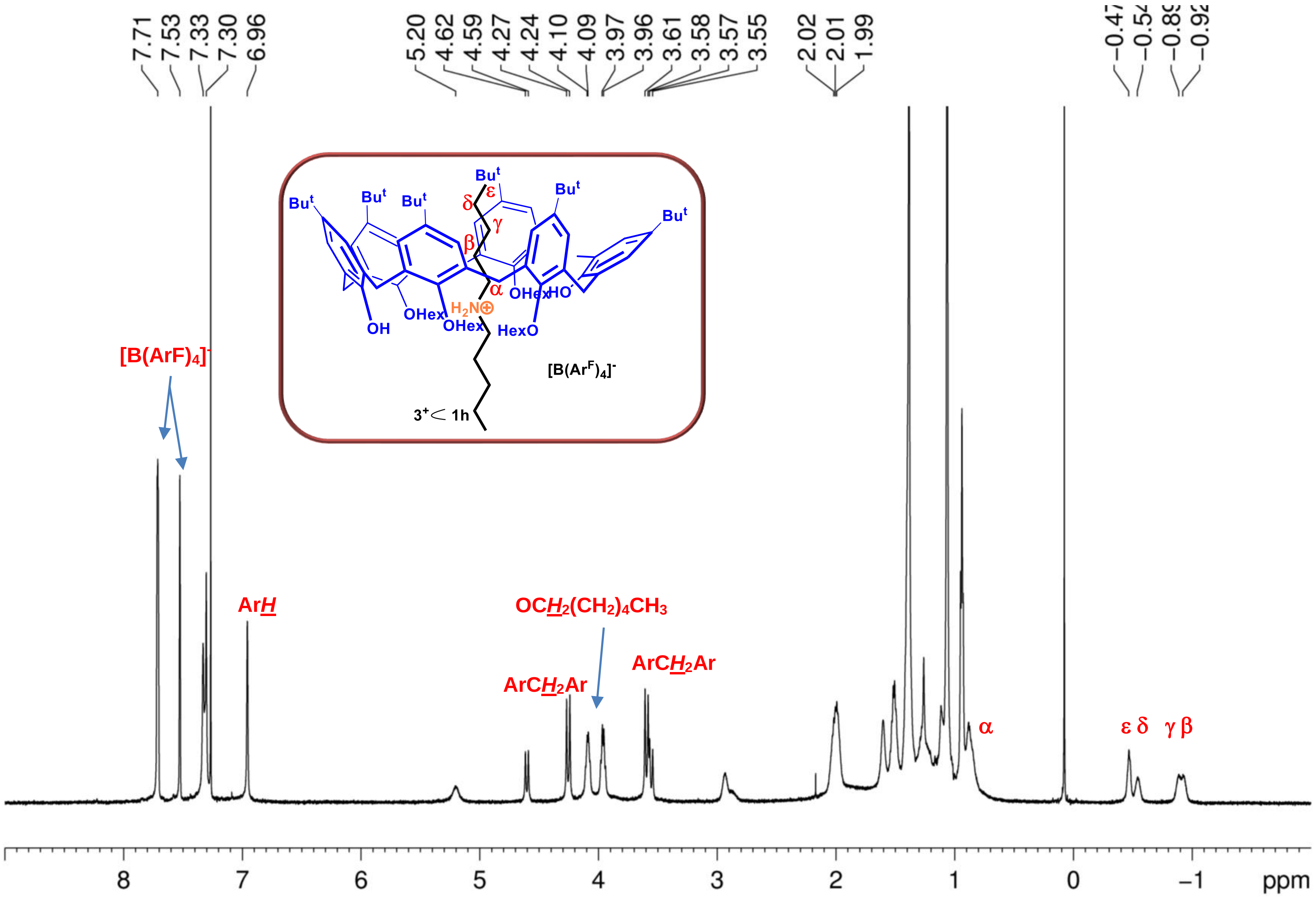

Figure S41. ^{1}H NMR spectrum of derivative **3$^+$⊂ 1h** (600 MHz, $CDCl_3$, 298 K).

2D COSY Spectrum of Derivative 3⁺⊂ 1h

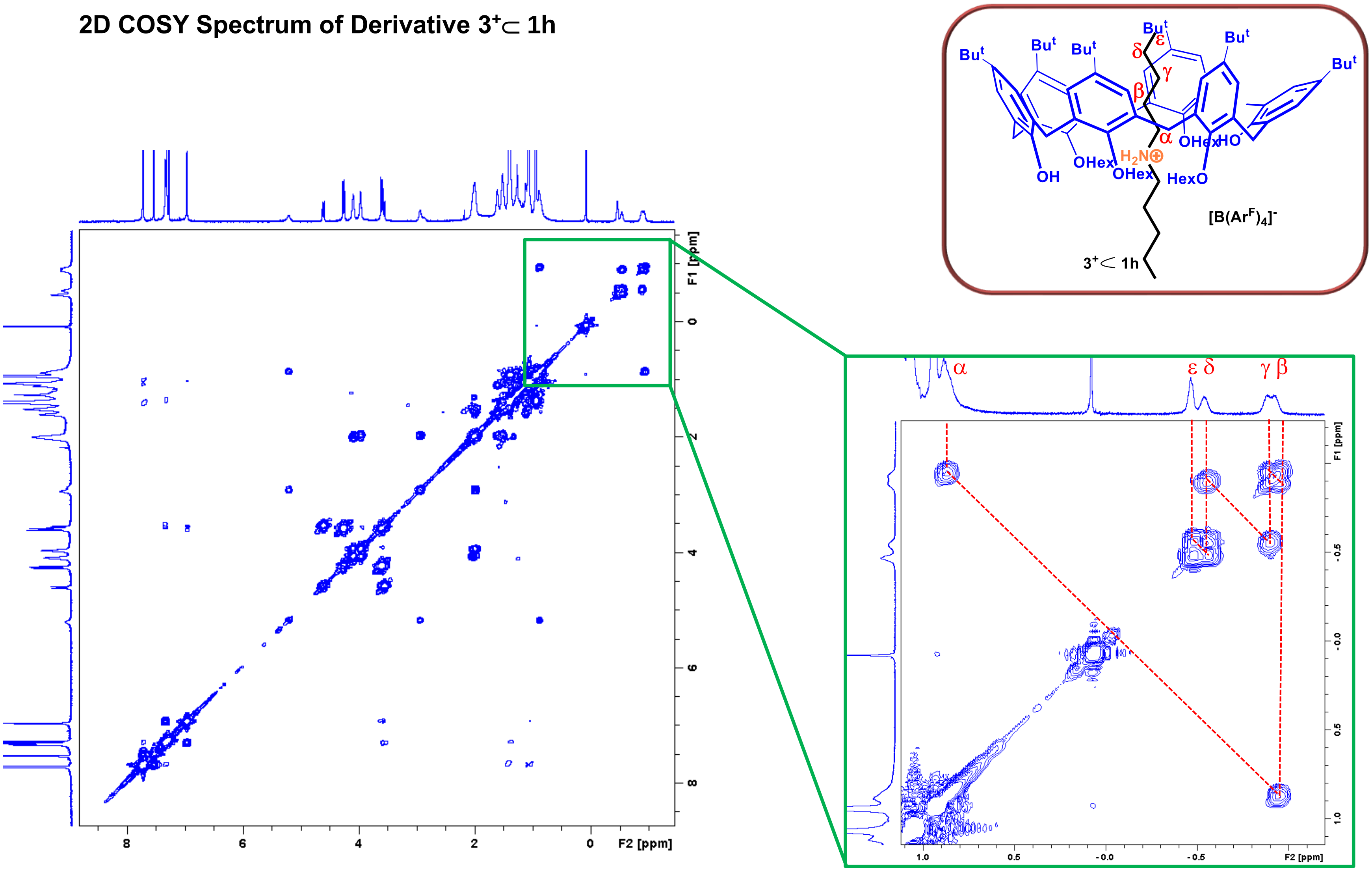

Figure S42. 2D COSY spectrum of derivative **3⁺⊂ 1h** (600 MHz, $CDCl_3$, 298 K).

2D HSQC Spectrum of Derivative $3^{+}\subset 1h$

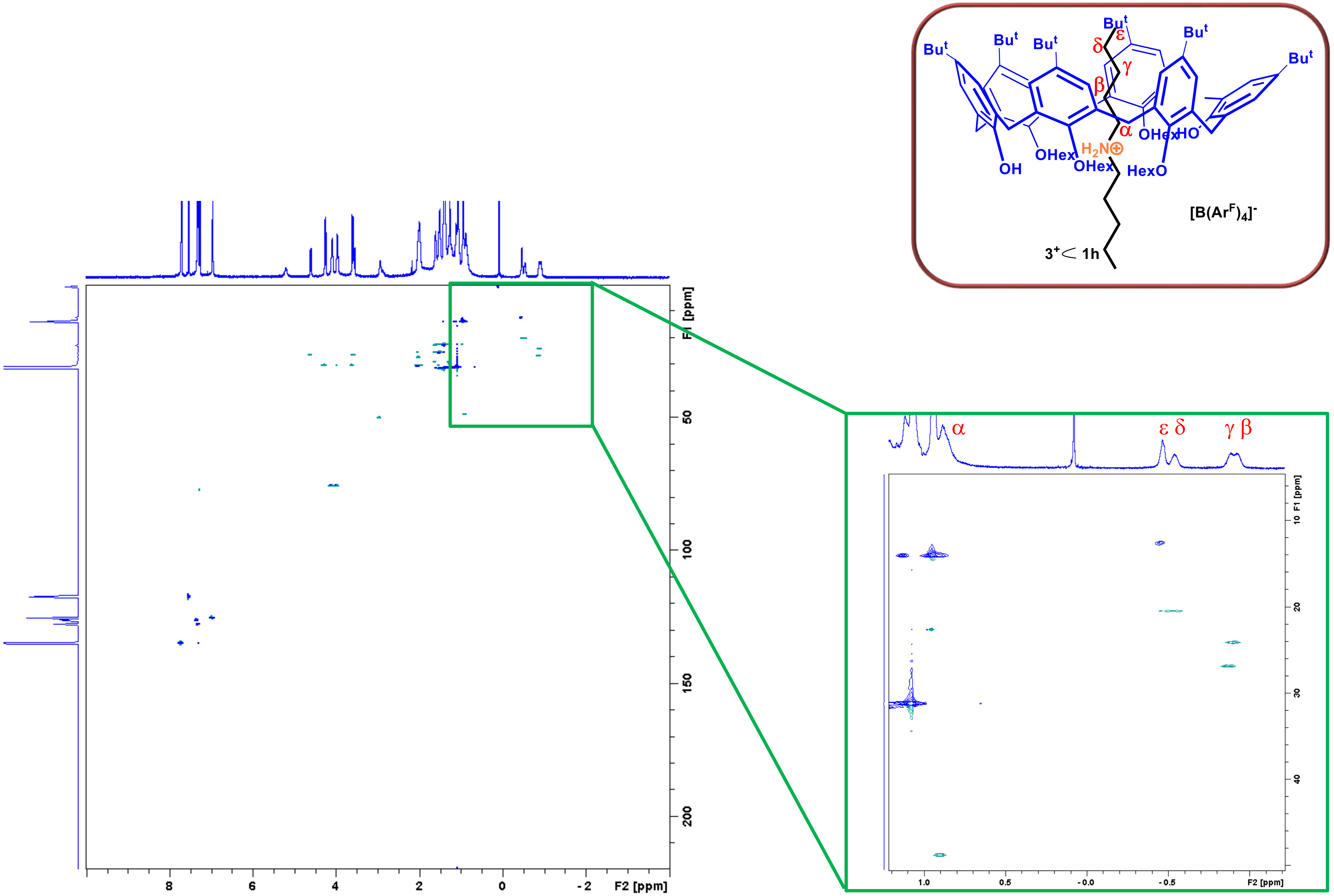

Figure S43. 2D HSQC spectrum of derivative **$3^{+}\subset 1h$** (600 MHz, $CDCl_3$, 298 K).

^{1}H NMR Spectrum of Derivative 4^{+}⊂ 1h

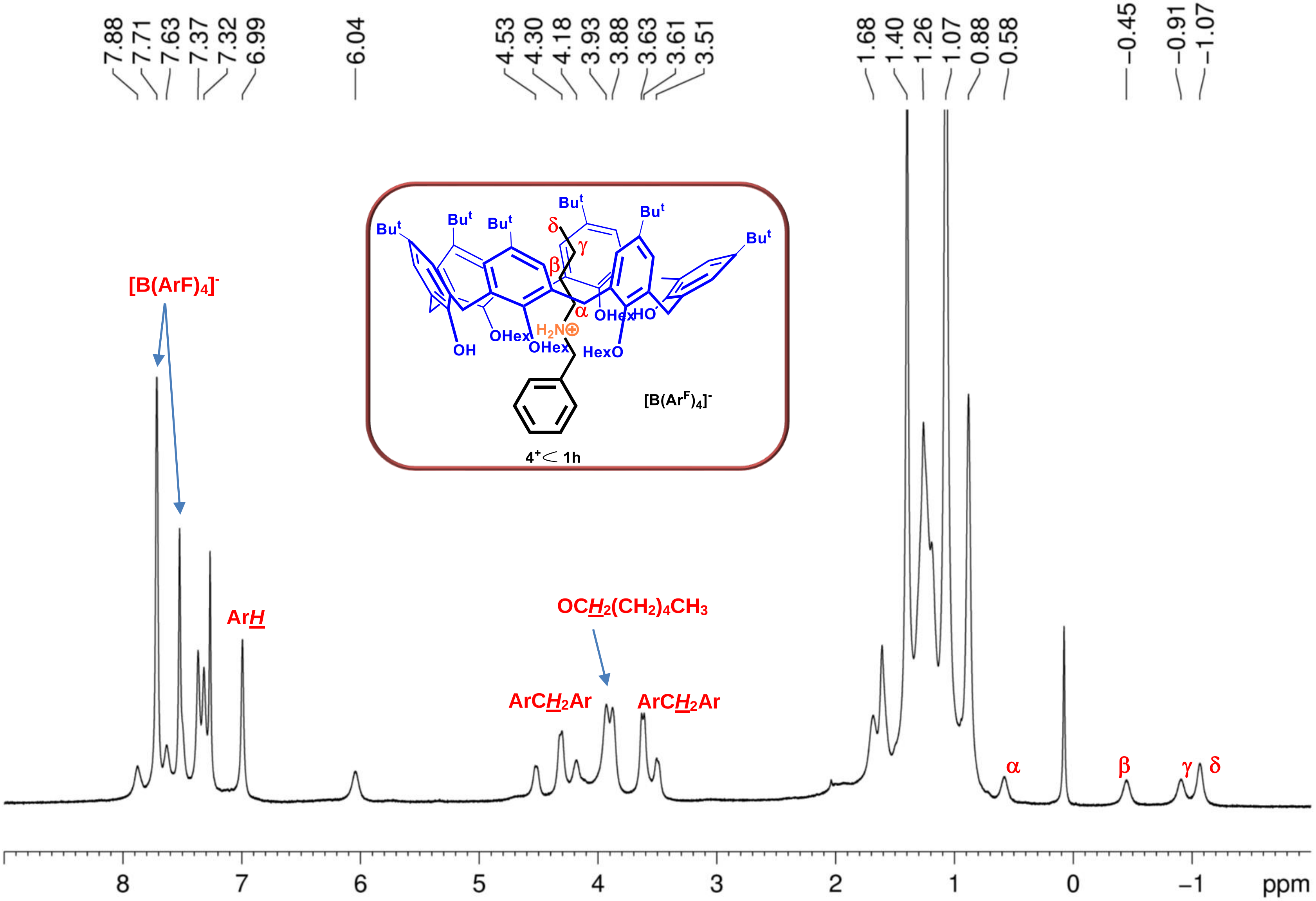

Figure S44. ^{1}H NMR spectrum of derivative **4^{+}⊂ 1h** (600 MHz, $CDCl_3$, 298 K).

2D COSY Spectrum of Derivative 4⁺⊂ 1h

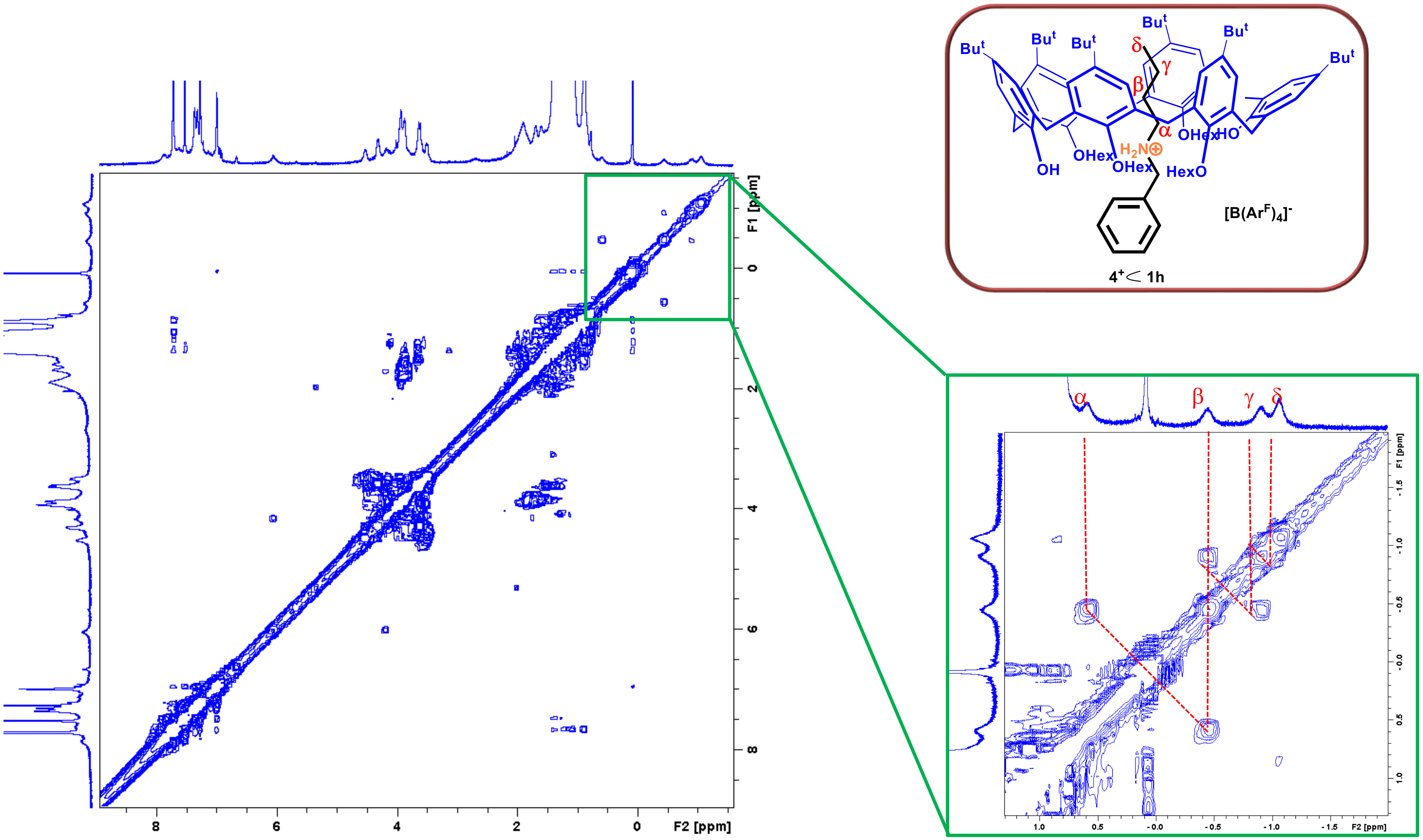

Figure S45. 2D COSY spectrum of derivative **4⁺⊂ 1h** (600 MHz, $CDCl_3$, 298 K).

^{1}H NMR determination of K_{ass} values

The association constant values of complexes were calculated by means of three methods:

a) ^{1}H NMR competition experiments. In this case, was performed an analysis of a 1:1:1 mixture of host, and two guests (or two host and one guest) in an NMR tube using 0.5 mL of $CDCl_3$ as solvent. The following equation was used to obtain K_{ass} value.

$$K_{A\subset H}=\frac{[HG_A]}{[H][G_A]} \text{ and } K_{B\subset H}=\frac{[HG_B]}{[H][G_B]} \longrightarrow K_{rel}=\frac{K_{A\subset H}}{K_{B\subset H}}=\frac{[HG_A][H][G_B]}{[HG_B][H][G_A]} \longrightarrow$$

$$\begin{matrix}[HG_A]=[G_B]\\ [HG_B]=[G_A]\end{matrix} \longrightarrow K_{rel}=\frac{K_{A\subset H}}{K_{B\subset H}}=\frac{[HG_A]^2}{[HG_B]^2}$$

b) Quantitative ^{1}H NMR experiments using 1,1,2,2-tetrachloroethane as the internal standard. In this case, ^{1}H NMR experiments were carried out on a 1:1 mixture of host and guest in 0.5 mL of $CDCl_3$ containing 1 μL of 1,1,2,2-tetrachloroethane (d= 1.586 g/mL) as internal standard. The following equation was used to obtain the moles of the complex:

$$\frac{G_a}{G_b}=\frac{F_a}{F_b}\times\frac{N_b}{N_a}\times\frac{M_a}{M_b}$$

Where:

G_a = grams of 1,1,2,2-Tetrachloroethane**;** G_b= grams of pseudorotaxane.

F_a and F_b = areas of the signal related of 1,1,2,2-tetrachloroethane and pseudorotaxane.

N_a and N_b = numbers of nuclei which cause the signals (N_a for 1,1,2,2-tetrachloroethane**;** N_b for pseudorotaxane)

M_a and M_b = molecular masses of 1,1,2,2-tetrachloroethane (a) and pseudorotaxane (b).

c) Integration of free and complexed ^{1}H NMR signals of host or guest. In this case, an equimolar solution of host and guest was solubilized in $CDCl_3$ and equilibrated in a NMR tube.

Table S2. Relative stability constants (Krel) calculated by ^{1}H NMR competition experiments.

	$K_{4^+\subset 1a}$ $(6.5\pm0.9 \times 10^{4}\ M^{-1})$	$K_{4^+\subset 1b}$ $(5.1\pm0.2 \times 10^{5}\ M^{-1})$	$K_{2^+\subset 1f}$ $(7.4\pm0.2 \times 10^{4}\ M^{-1})$	$K_{3^+\subset 1f}$ $(5.0\pm0.2 \times 10^{4}\ M^{-1})$	$K_{2^+\subset 1g}$ $(8.5\pm0.2 \times 10^{4}\ M^{-1})$	$K_{3^+\subset 1h}$ $(7.1\pm0.2 \times 10^{3}\ M^{-1})$
$K_{2^+\subset 1b}$ $(1.2\pm0.2 \times 10^{5}\ M^{-1})^{a}$			$K_{rel} = \frac{K_{2^+@1f}}{K_{2^+@1b}} = 0.62$		$K_{rel} = \frac{K_{2^+@1g}}{K_{2^+@1b}} = 0.71$	
$K_{3^+\subset 1b}$ $(3.5\pm0.2 \times 10^{4}\ M^{-1})^{a}$				$K_{rel} = \frac{K_{3^+@1f}}{K_{3^+@1b}} = 1.43$		$K_{rel} = \frac{K_{2^+@1g}}{K_{3^+@1g}} = 0.20$
$K_{4^+\subset 1f}$ $(1.4\pm0.2 \times 10^{5}\ M^{-1})$		$K_{rel} = \frac{K_{4^+@1f}}{K_{4^+@1b}} = 3.64$				
$K_{3^+\subset 1g}$ $(3.4\pm0.2 \times 10^{5}\ M^{-1})$					$K_{rel} = \frac{K_{2^+@1g}}{K_{3^+@1g}} = 0.25$	
$K_{4^+\subset 1g}$ $(9.3\pm0.2 \times 10^{4}\ M^{-1})$		$K_{rel} = \frac{K_{4^+@1b}}{K_{4^+@1g}} = 5.48$			$K_{rel} = \frac{K_{2^+@1g}}{K_{3^+@1g}} = 0.91$	
$K_{7^+\subset 1a}$ $(3.6\pm0.5 \times 10^{2}\ M^{-1})^{b}$	$K_{rel} = \frac{K_{4^+@1a}}{K_{7^+@1a}} = 180.6$					

[a] See reference 10b: (Gaeta, C.; Troisi, F.; Neri, P. endo-Cavity Complexation and Through-the-Annulus Threading of Large Calixarenes Induced by Very Loose Alkylammonium Ion Pairs. *Org. Lett.* **2010,** *12*, 2092-2095).

[b]K_{ass} value was calculated by Integration of free and complexed ^{1}H NMR signals of host or guest (see Figure S46).

K_{ass} value of 7^{+}⊂ 1a

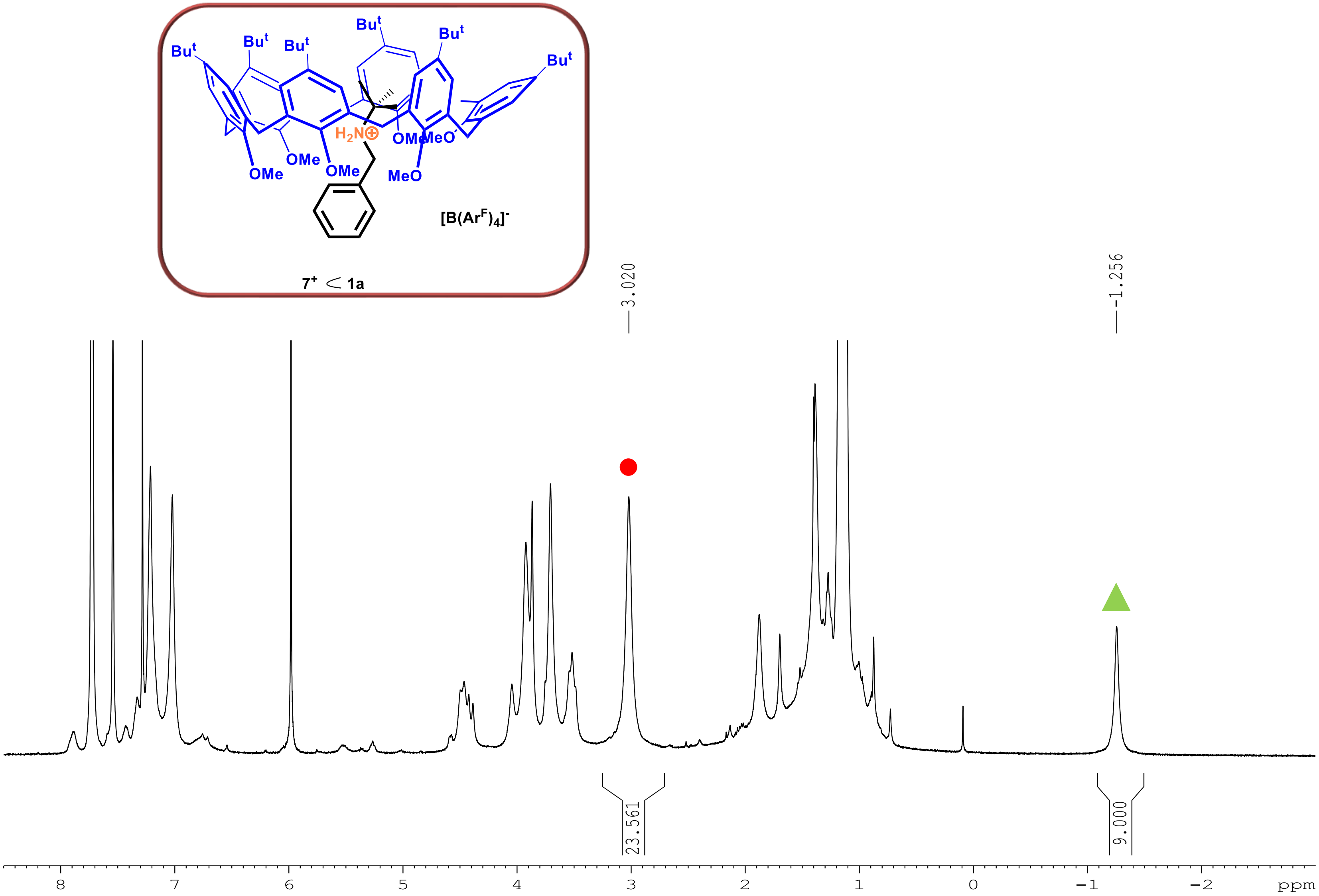

Figure S46. ^{1}H NMR spectrum of equimolar solution ($3.8 \cdot 10^{-3}$ M) of **1a** and **7^{+}·[B(Ar^{F})$_4$]$^{-}$**. The association constant $\boldsymbol{K_{ass}}$ value was calculated by integration of complexed (▲) and free derivative **1a** (●) ($CDCl_3$, 400 MHz, 298 K)

Kass value of $4^+ \subset$ 1b

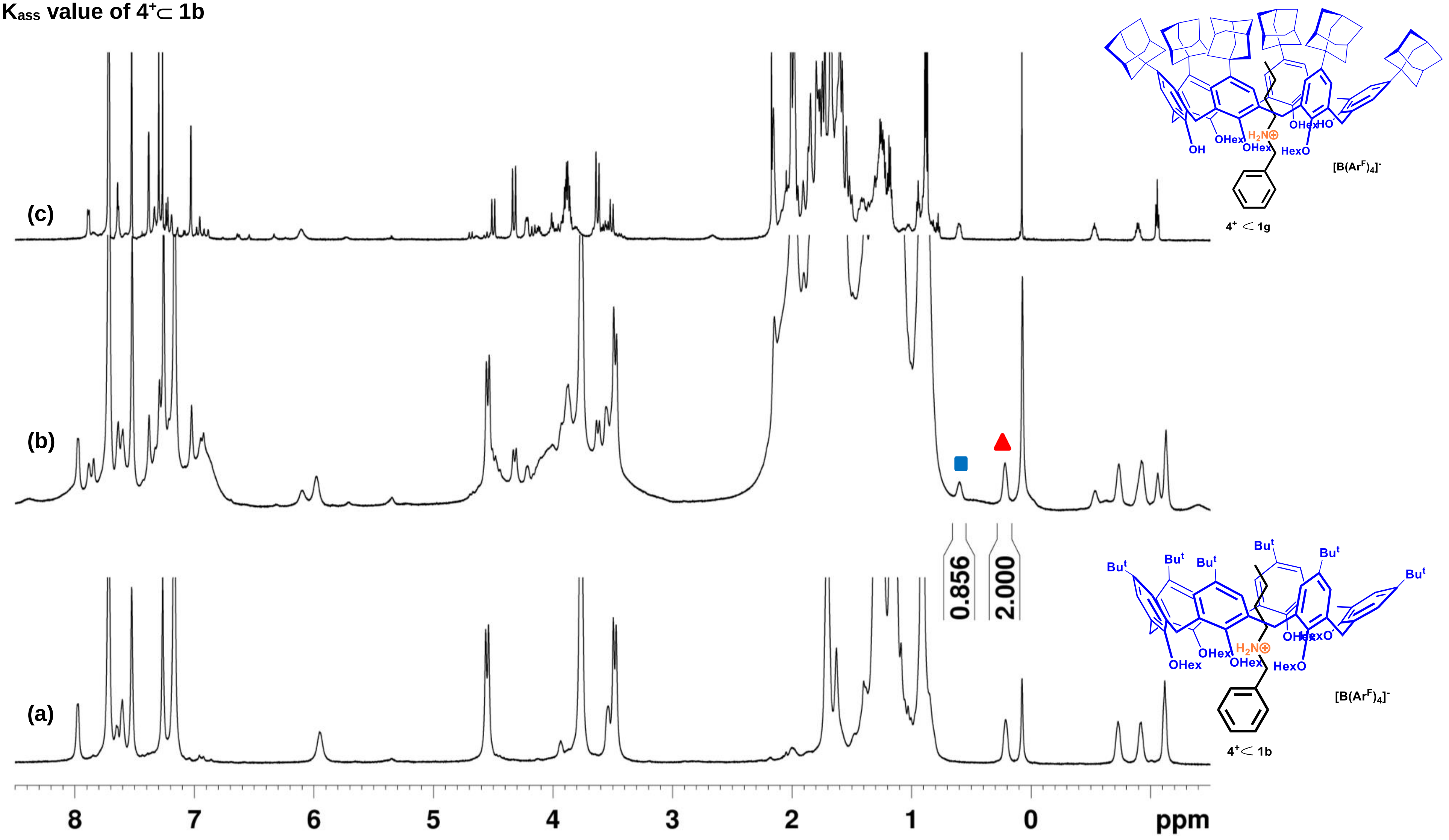

Figure S47. ^{1}H NMR spectrum of **(a)** an equimolar solution (4.5 mM) of **4**$^+$ and **1b** in 0.5 mL of $CDCl_3$; **(b)** an equimolar solution (4.5 mM) of **4**$^+$, **1g** and **1b** in 0.5 mL of $CDCl_3$. **(c)** an equimolar solution (4.5 mM) of **4**$^+$ and **1g** in 0.5 mL of $CDCl_3$.The association constant K_a value was calculated by integration of signal of complex **4**$^+$⊂ **1b** (▲) and complex **4**$^+$⊂ **1g** (■) (600 MHz, $CDCl_3$, 298 K).

K_{ass} value of $2^+ \subset$ 1f

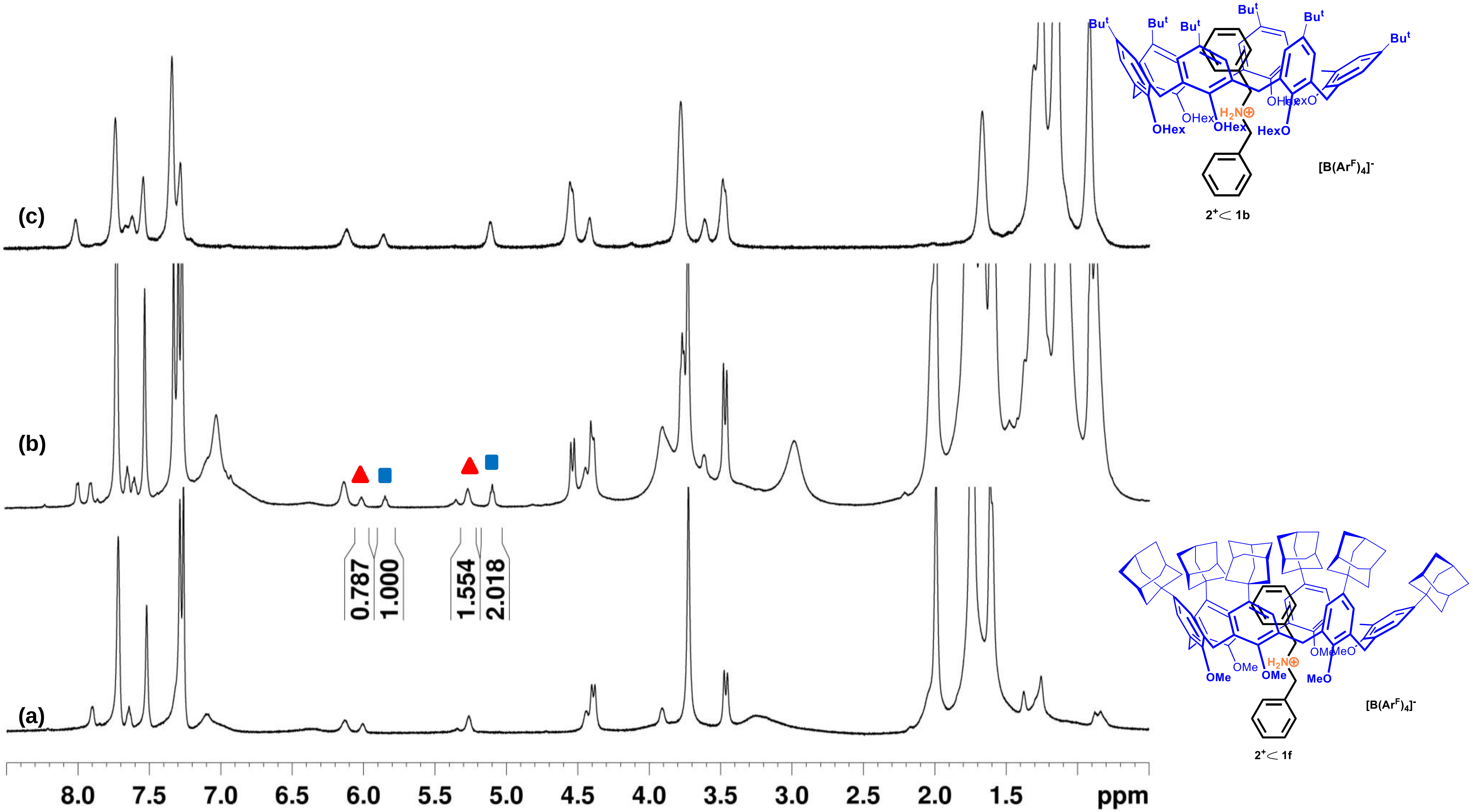

Figure S48. ^{1}H NMR spectrum of **(a)** an equimolar solution (5.2 mM) of **2**$^+$ and **1f** in 0.5 mL of $CDCl_3$; **(b)** an equimolar solution (5.2 mM) of **2**$^+$, **1f** and **1b** in 0.5 mL of $CDCl_3$. **(c)** an equimolar solution (5.2 mM) of **2**$^+$ and **1b** in 0.5 mL of $CDCl_3$.The association constant K_a value was calculated by integration of signal of complex **2**$^+ \subset$ **1f** (▲) and complex **2**$^+ \subset$ **1b** (■) (600 MHz, $CDCl_3$, 298 K).

K_{ass} value of $3^+\subset$ 1f

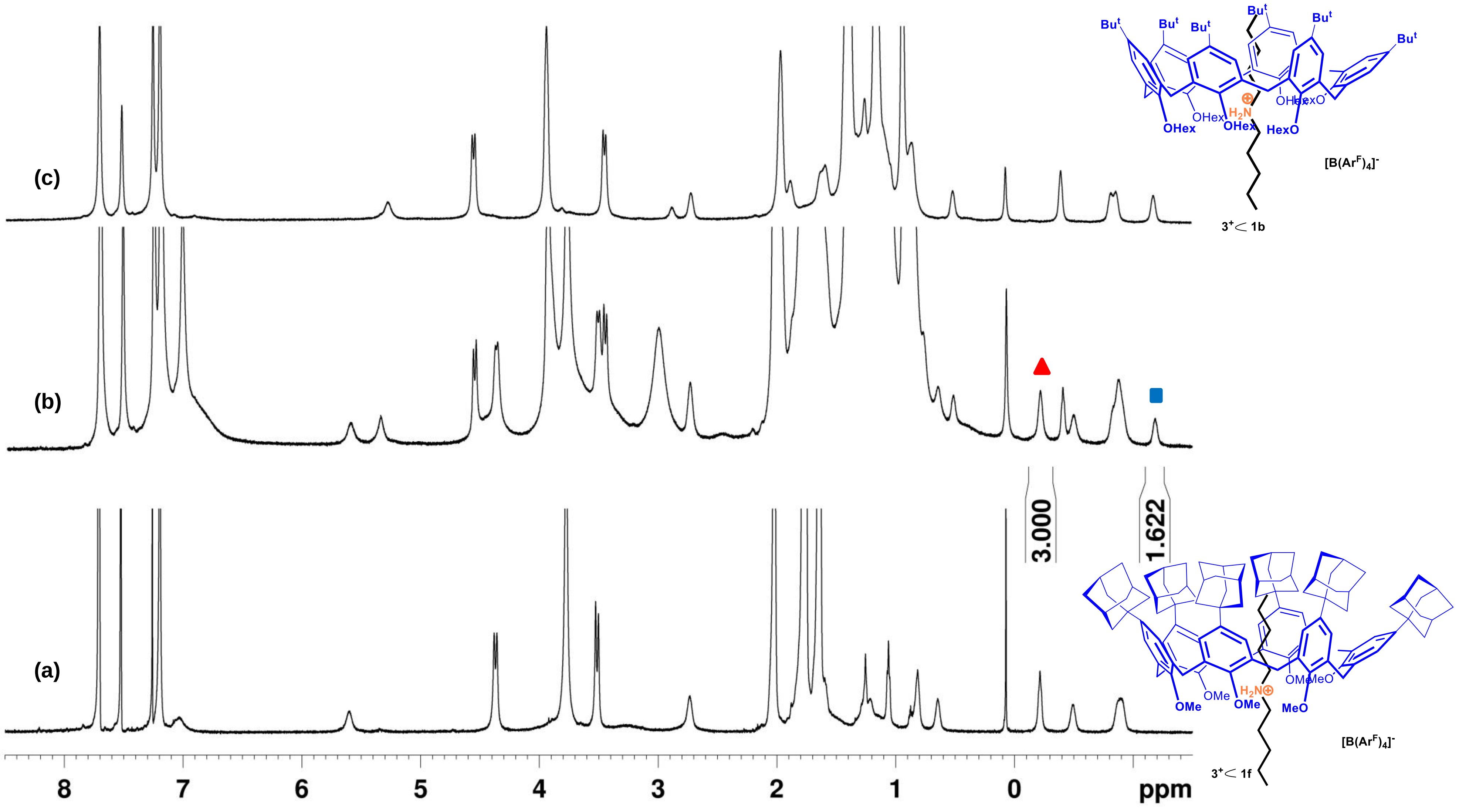

Figure S49. ^{1}H NMR spectrum of **(a)** an equimolar solution (5.2 mM) of $\mathbf{3^+}$ and **1f** in 0.5 mL of $CDCl_3$; **(b)** an equimolar solution (5.2 mM) of $\mathbf{3^+}$, **1f** and **1b** in 0.5 mL of $CDCl_3$. **(c)** an equimolar solution (5.2 mM) of $\mathbf{3^+}$ and **1b** in 0.5 mL of $CDCl_3$.The association constant K_a value was calculated by integration of signal of complex $\mathbf{3^+\subset 1f}$ (▲) and complex $\mathbf{3^+\subset 1b}$ (■) (600 MHz, $CDCl_3$, 298 K).

K_{ass} value of $4^{+}\subset$ 1f

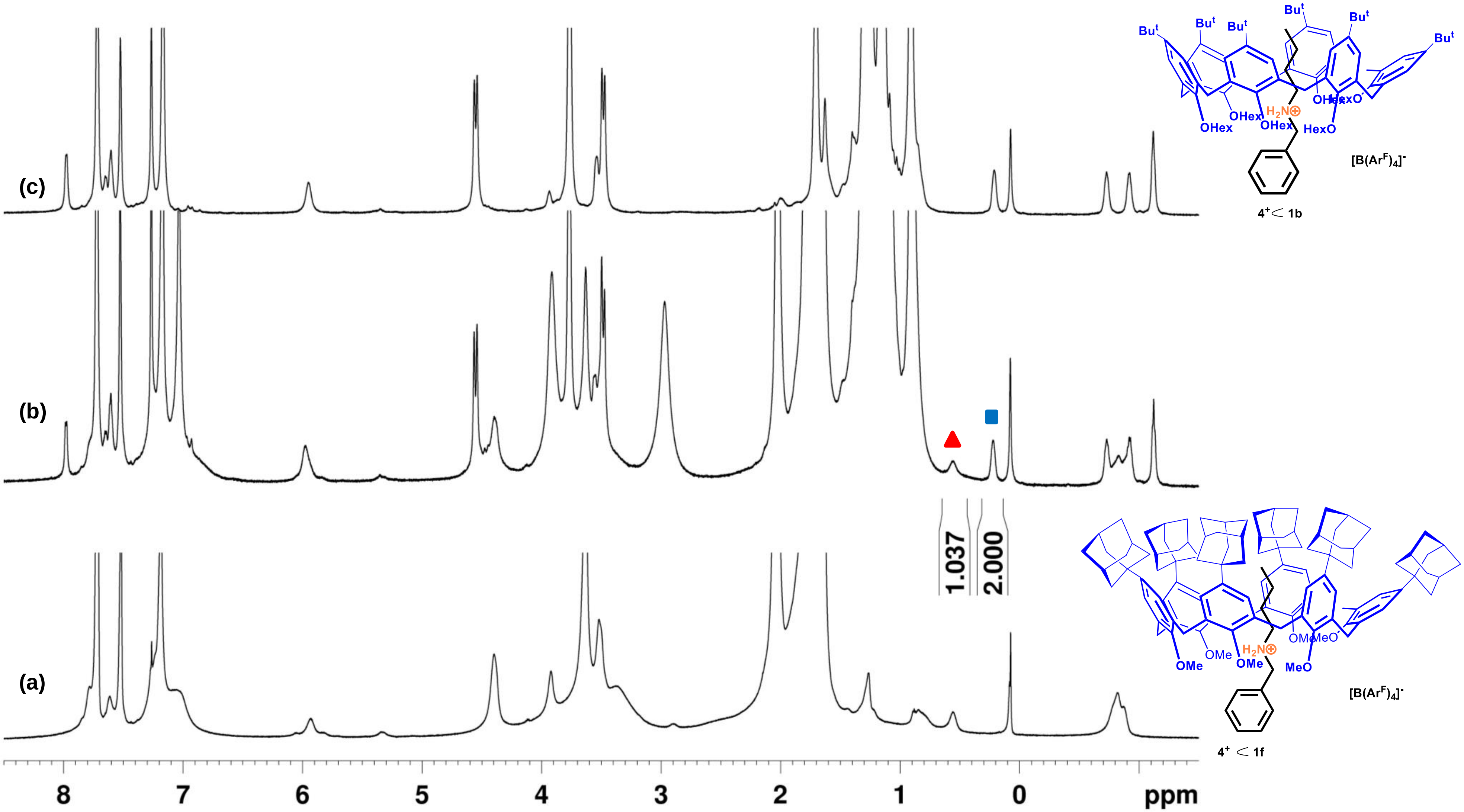

Figure S50. ^{1}H NMR spectrum of **(a)** an equimolar solution (5.2 mM) of $\mathbf{4}^{+}$ and **1f** in 0.5 mL of $CDCl_3$; **(b)** an equimolar solution (5.2 mM) of $\mathbf{4}^{+}$, **1f** and **1b** in 0.5 mL of $CDCl_3$. **(c)** an equimolar solution (5.2 mM) of $\mathbf{4}^{+}$ and **1b** in 0.5 mL of $CDCl_3$.The association constant K_a value was calculated by integration of signal of complex $\mathbf{4}^{+}\subset$ **1f** (▲) and complex $\mathbf{4}^{+}\subset$ **1b** (■) (600 MHz, $CDCl_3$, 298 K).

K_{ass} value of $2^+ \subset$ 1g

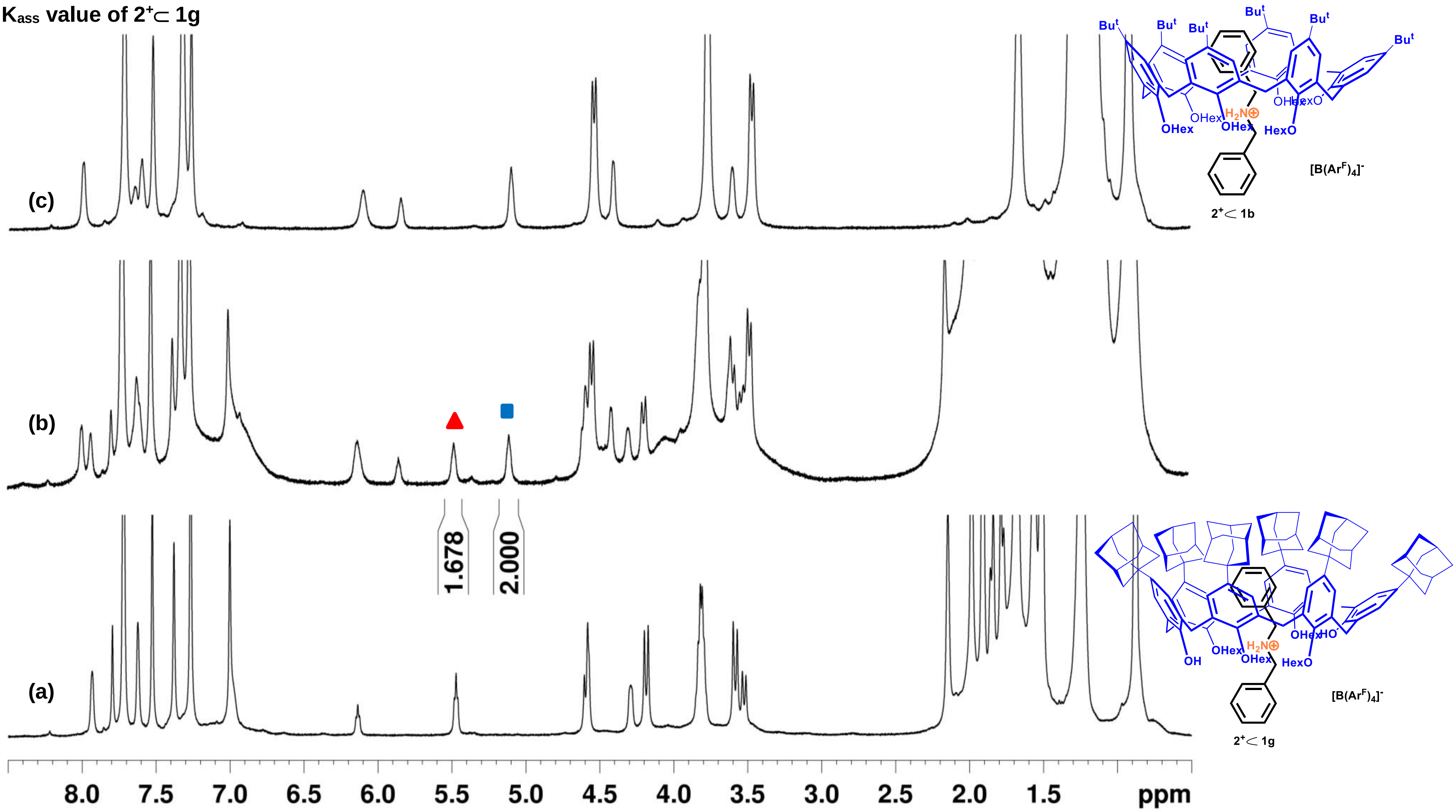

Figure S51. ^{1}H NMR spectrum of **(a)** an equimolar solution (4.5 mM) of **2⁺** and **1g** in 0.5 mL of $CDCl_3$; **(b)** an equimolar solution (4.5 mM) of **2⁺**, **1g** and **1b** in 0.5 mL of $CDCl_3$. **(c)** an equimolar solution (4.5 mM) of **2⁺** and **1b** in 0.5 mL of $CDCl_3$.The association constant K_a value was calculated by integration of signal of complex **$2^+ \subset$ 1g** (▲) and complex **$2^+ \subset$ 1b** (■) (600 MHz, $CDCl_3$, 298 K).

K_{ass} value of $3^+ \subset$ 1g

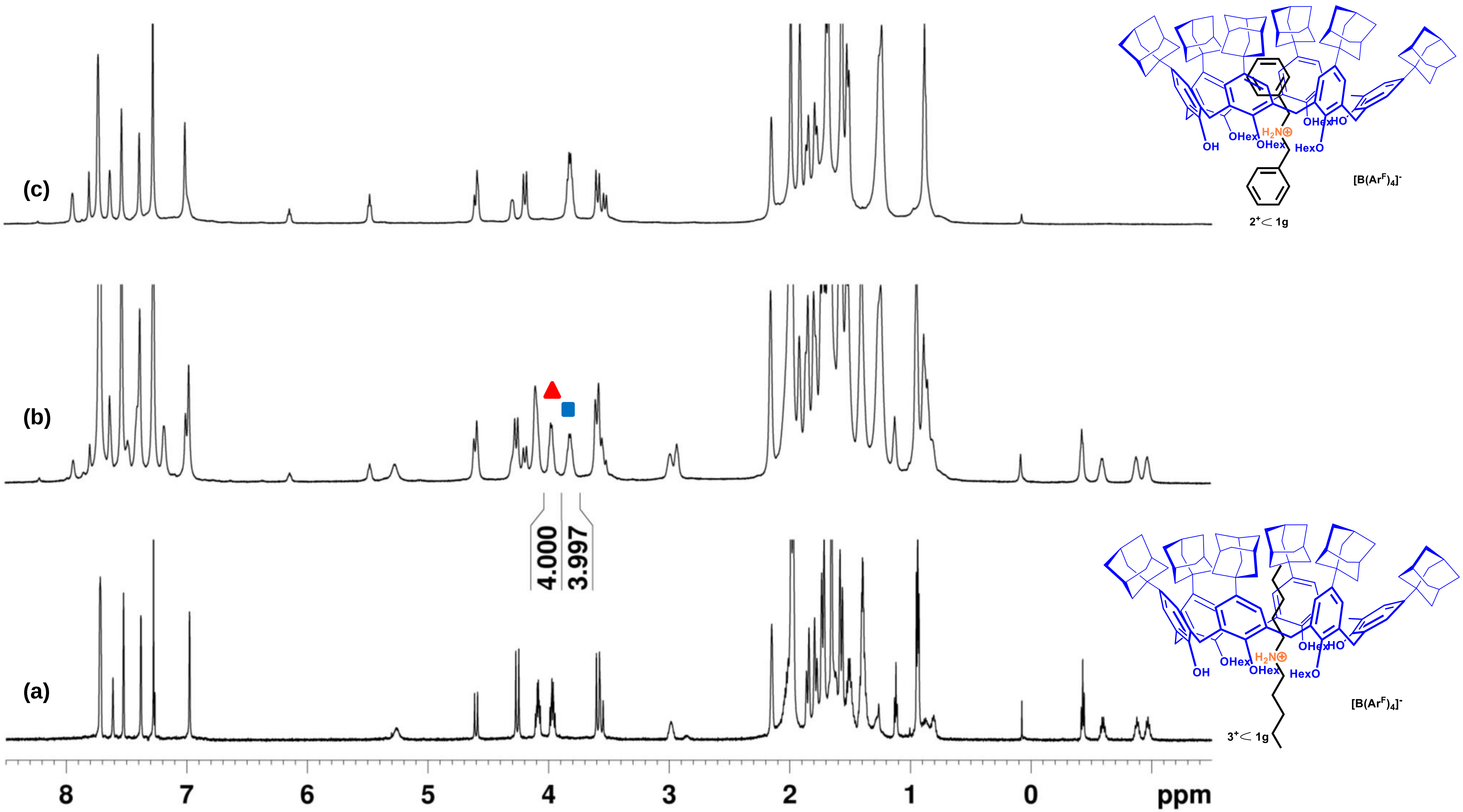

Figure S52. ^{1}H NMR spectrum of **(a)** an equimolar solution (4.5 mM) of **3^+** and **1g** in 0.5 mL of $CDCl_3$; **(b)** an equimolar solution (4.5 mM) of **3^+**, **1g** and **2^+** in 0.5 mL of $CDCl_3$. **(c)** an equimolar solution (4.5 mM) of **2^+** and **1g** in 0.5 mL of $CDCl_3$.The association constant K_a value was calculated by integration of signal of complex **$3^+ \subset$ 1g** (▲) and complex **$2^+ \subset$ 1g** (■) (600 MHz, $CDCl_3$, 298 K).

K_{ass} value of $4^{+}\subset$ 1g

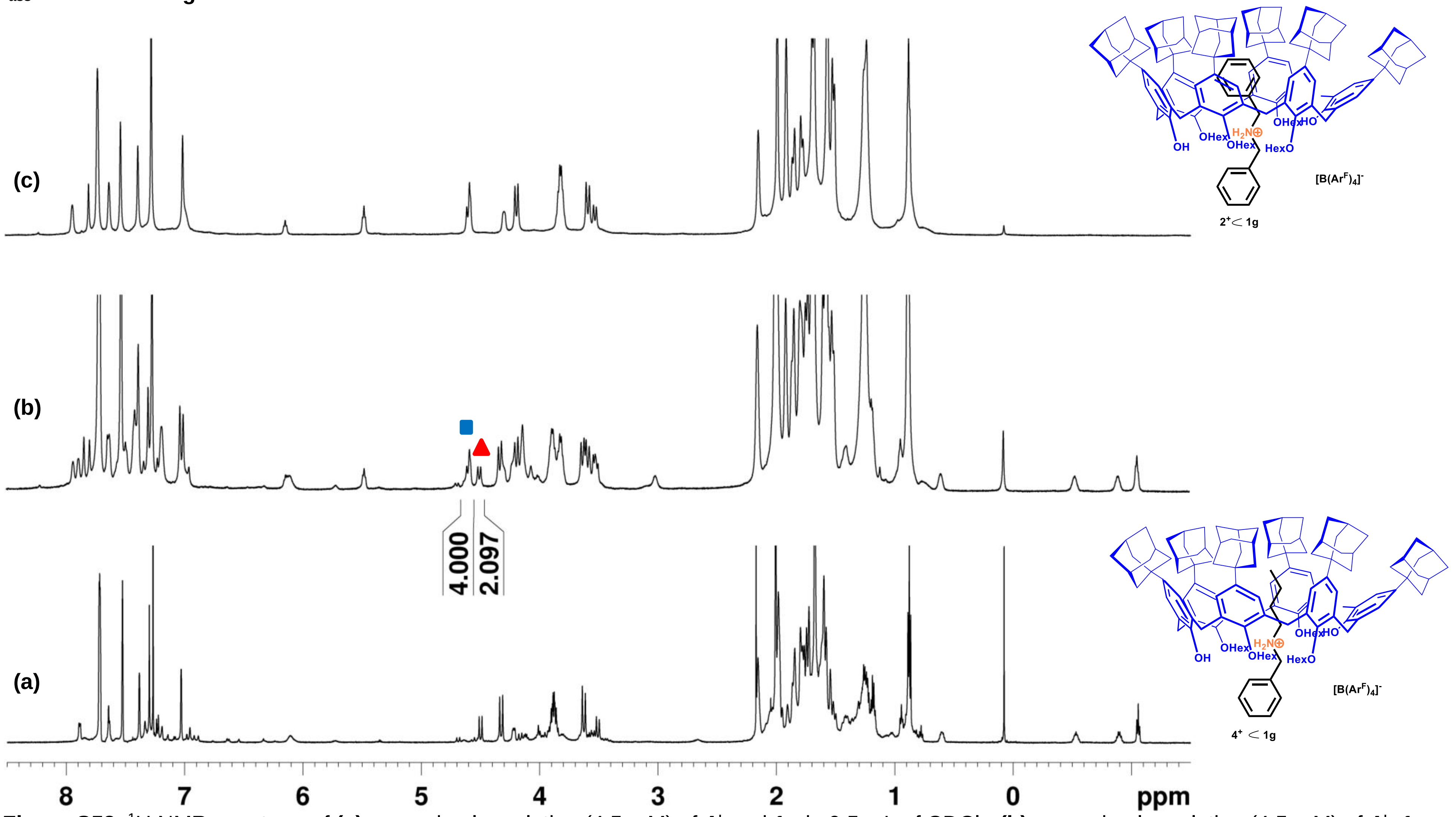

Figure S53. ^{1}H NMR spectrum of **(a)** an equimolar solution (4.5 mM) of **4⁺** and **1g** in 0.5 mL of $CDCl_3$; **(b)** an equimolar solution (4.5 mM) of **4⁺**, **1g** and **2⁺** in 0.5 mL of $CDCl_3$. **(c)** an equimolar solution (4.5 mM) of **2⁺** and **1g** in 0.5 mL of $CDCl_3$.The association constant K_a value was calculated by integration of signal of complex **$4^{+}\subset$ 1g** (▲) and complex **$2^{+}\subset$ 1g** (■) (600 MHz, $CDCl_3$, 298 K).

K_{ass} value of $3^{+}\subset$ 1h

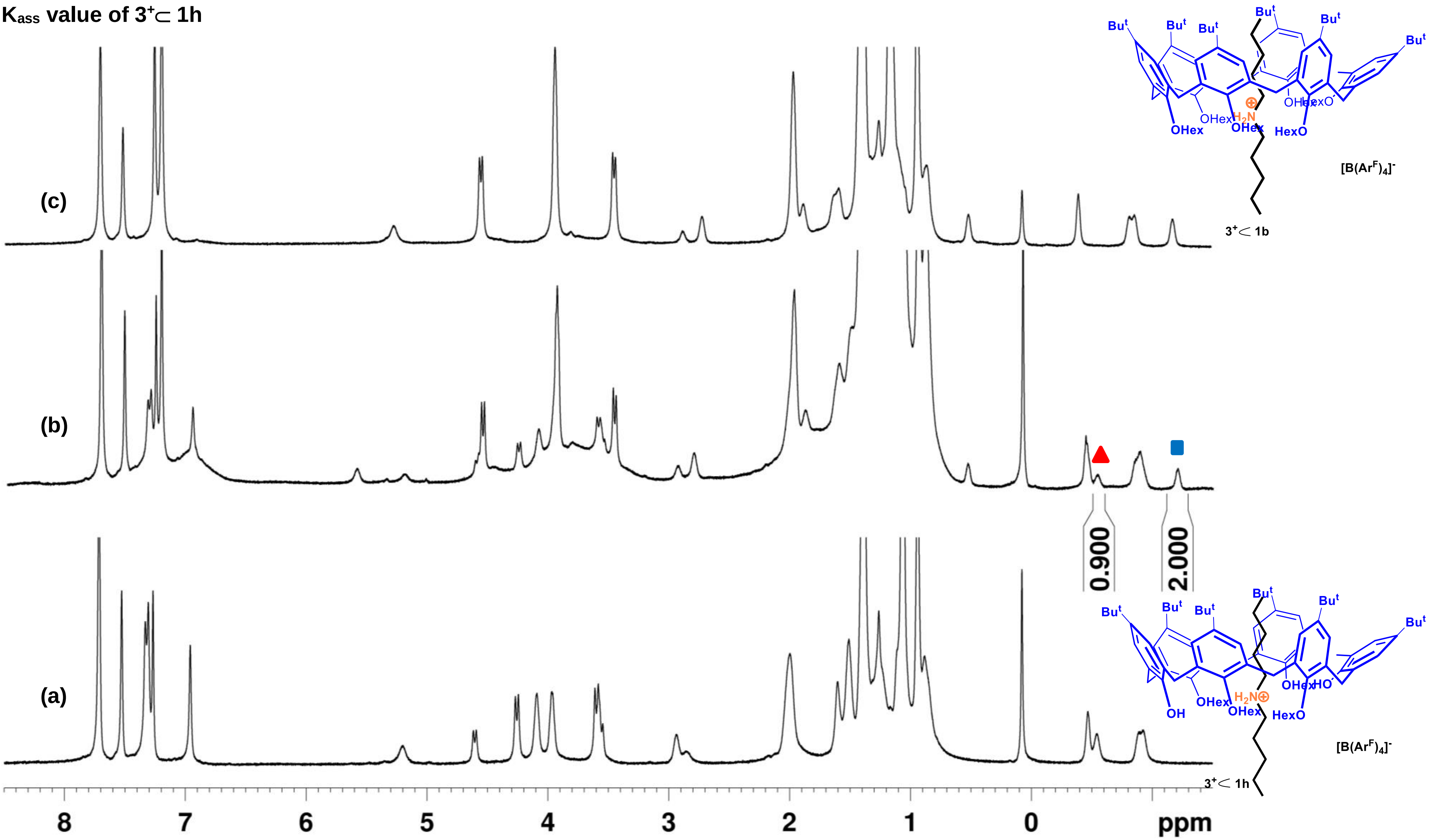

Figure S54. ^{1}H NMR spectrum of **(a)** an equimolar solution (6.1 mM) of $\mathbf{3^{+}}$ and **1h** in 0.5 mL of $CDCl_3$; **(b)** an equimolar solution (6.1 mM) of $\mathbf{3^{+}}$, **1h** and **1b** in 0.5 mL of $CDCl_3$. **(c)** an equimolar solution (6.1 mM) of $\mathbf{3^{+}}$ and **1b** in 0.5 mL of $CDCl_3$.The association constant K_a value was calculated by integration of signal of complex $\mathbf{3^{+}\subset 1h}$ (▲) and complex $\mathbf{3^{+}\subset 1b}$ (■) (600 MHz, $CDCl_3$, 298 K).

K_{ass} value of $4^{+}\subset$ 1a

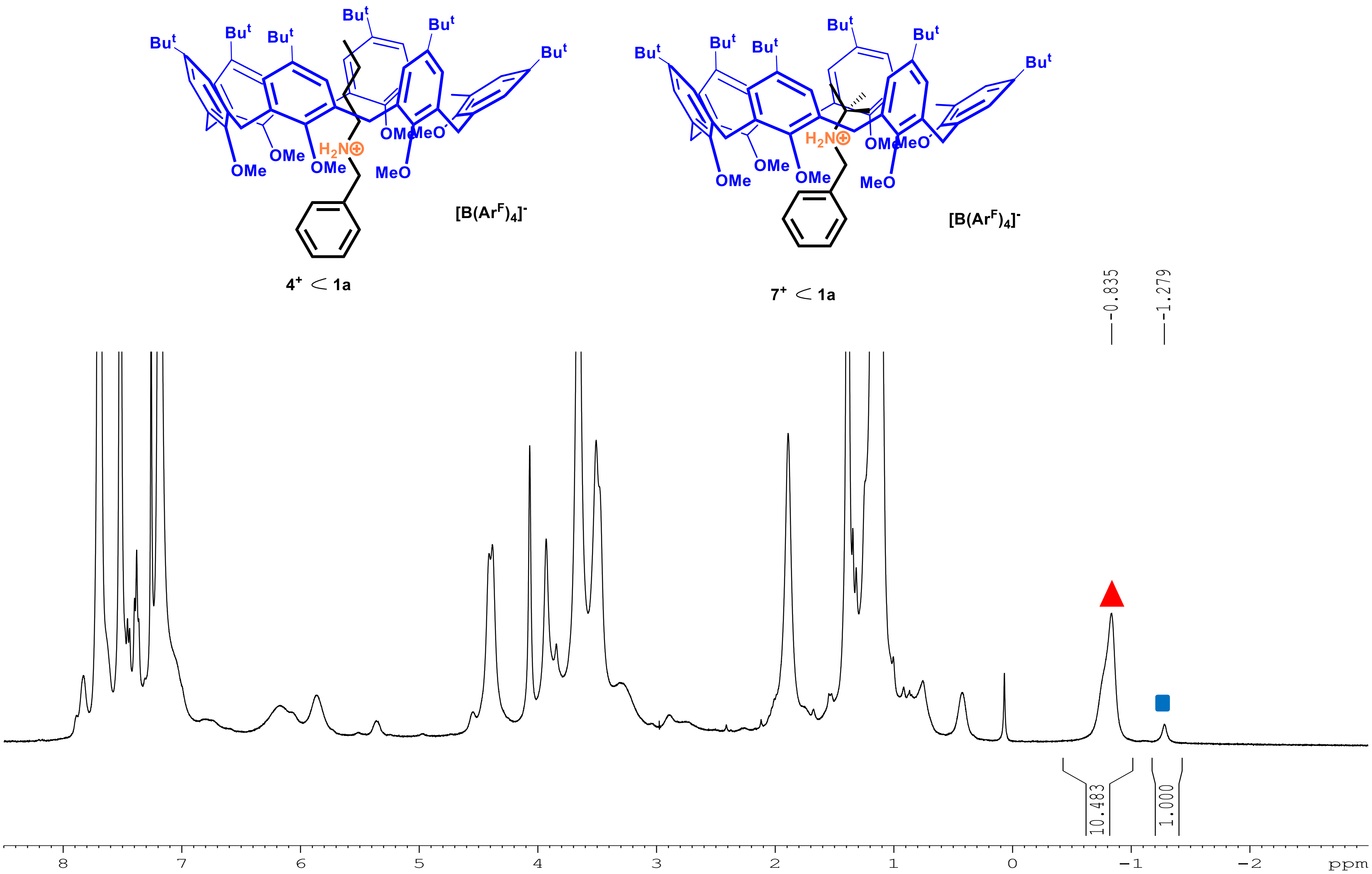

Figure 55. ^{1}H NMR spectrum of an equimolar solution (4.5 mM) of **4^{+}**, **1a** and **7^{+}** in 0.5 mL of $CDCl_3$. The association constant K_a value was calculated by integration of signal of complex **$4^{+}\subset$ 1a** (▲) and complex **$7^{+}\subset$ 1a** (■) (400 MHz, $CDCl_3$, 298 K).

K_{ass} value of $2^+ \subset$ 1h

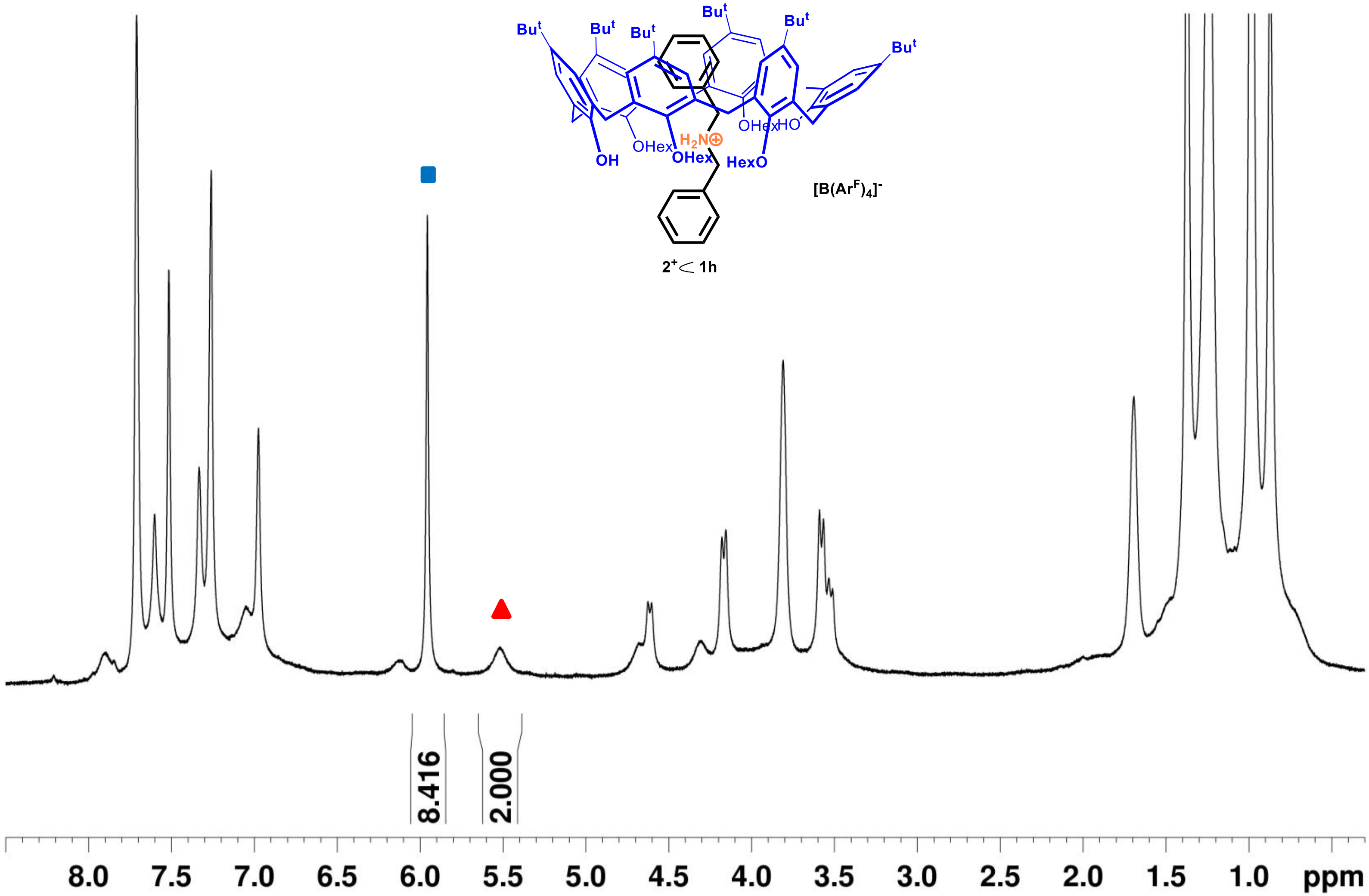

Figure S56. ^{1}H NMR spectrum of an equimolar solution (6.1 mM) of **2**$^+$ and **1h** in 0.5 mL of $CDCl_3$ containing 1 μL of 1,1,2,2-tetrachloroethane. The association constant K_a value was calculated by integration of signal of complex **2**$^+ \subset$ **1h** (▲) and 1,1,2,2-tetrachloroethane (■) (600 MHz, $CDCl_3$, 298 K).

K_{ass} value of $4^{+}\subset$ 1h

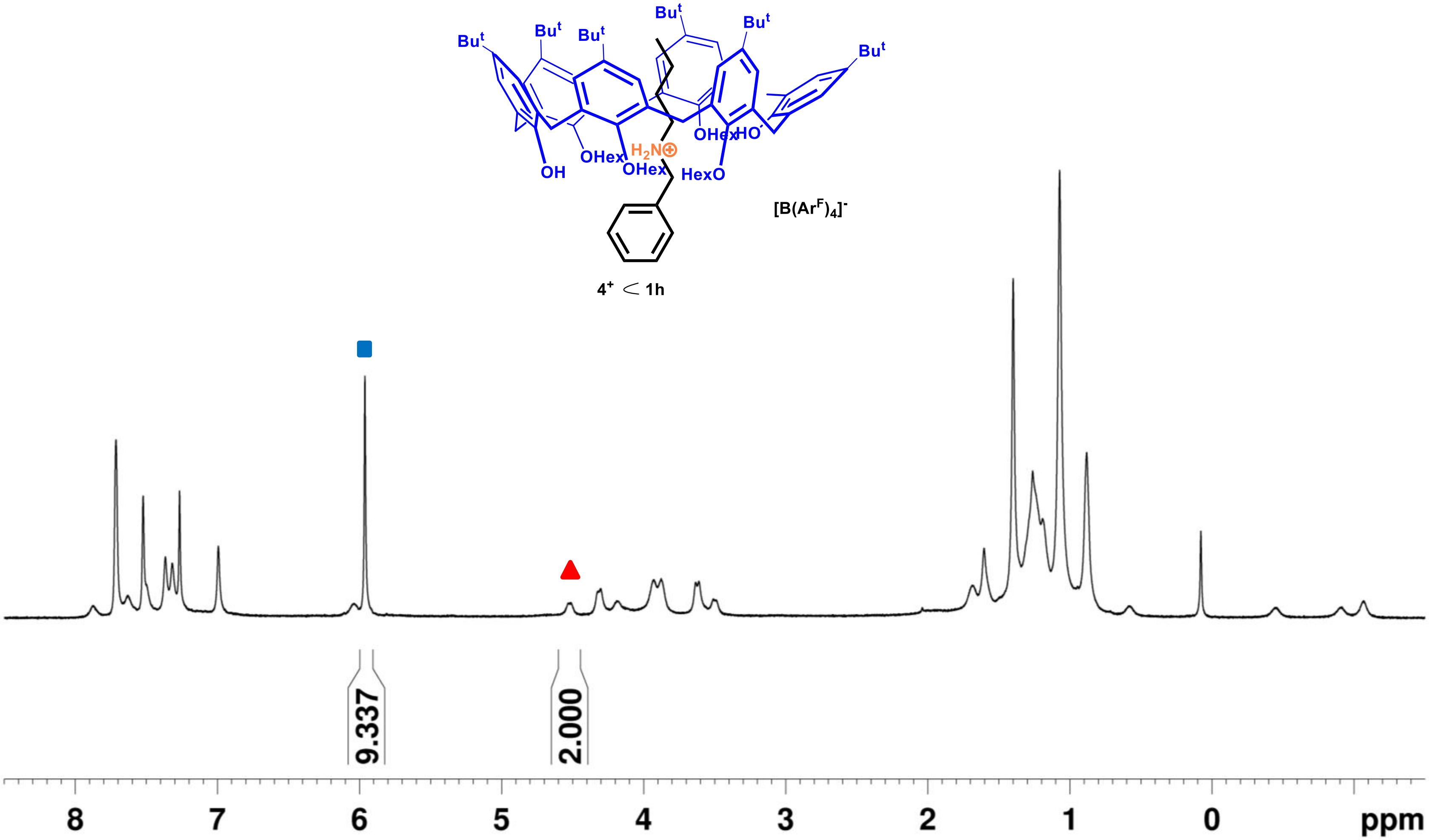

Figure S57. ^{1}H NMR spectrum of an equimolar solution (6.1 mM) of **2**$^{+}$ and **1h** in 0.5 mL of $CDCl_3$ containing 1 μL of 1,1,2,2-tetrachloroethane. The association constant K_a value was calculated by integration of signal of complex **2**$^{+}\subset$ **1h** (▲) and 1,1,2,2-tetrachloroethane (■) (600 MHz, $CDCl_3$, 298 K).

Competition experiments

1a vs 1f in the binding of 2^+.

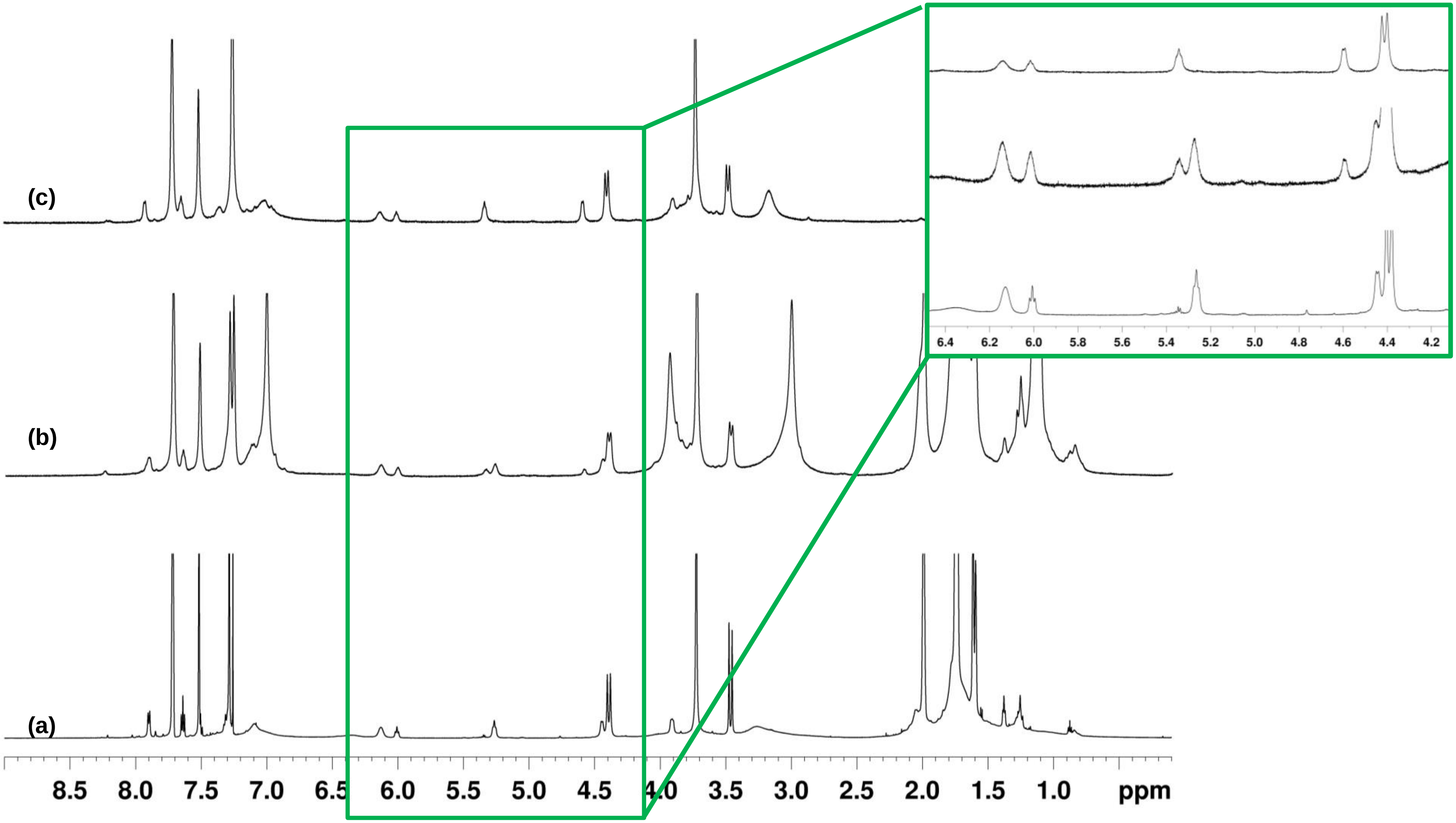

Figure S58. ^{1}H NMR spectrum of **(a)** an equimolar solution (5.2 mM) of **2^+** and **1f** in 0.5 mL of $CDCl_3$; **(b)** an equimolar solution (5.2 mM) of **2^+**, **1f** and **1a** in 0.5 mL of $CDCl_3$. **(c)** an equimolar solution (5.2 mM) of **2^+** and **1a** in 0.5 mL of $CDCl_3$ (600 MHz, $CDCl_3$, 298 K).

1b vs 1h in the binding of 2^+

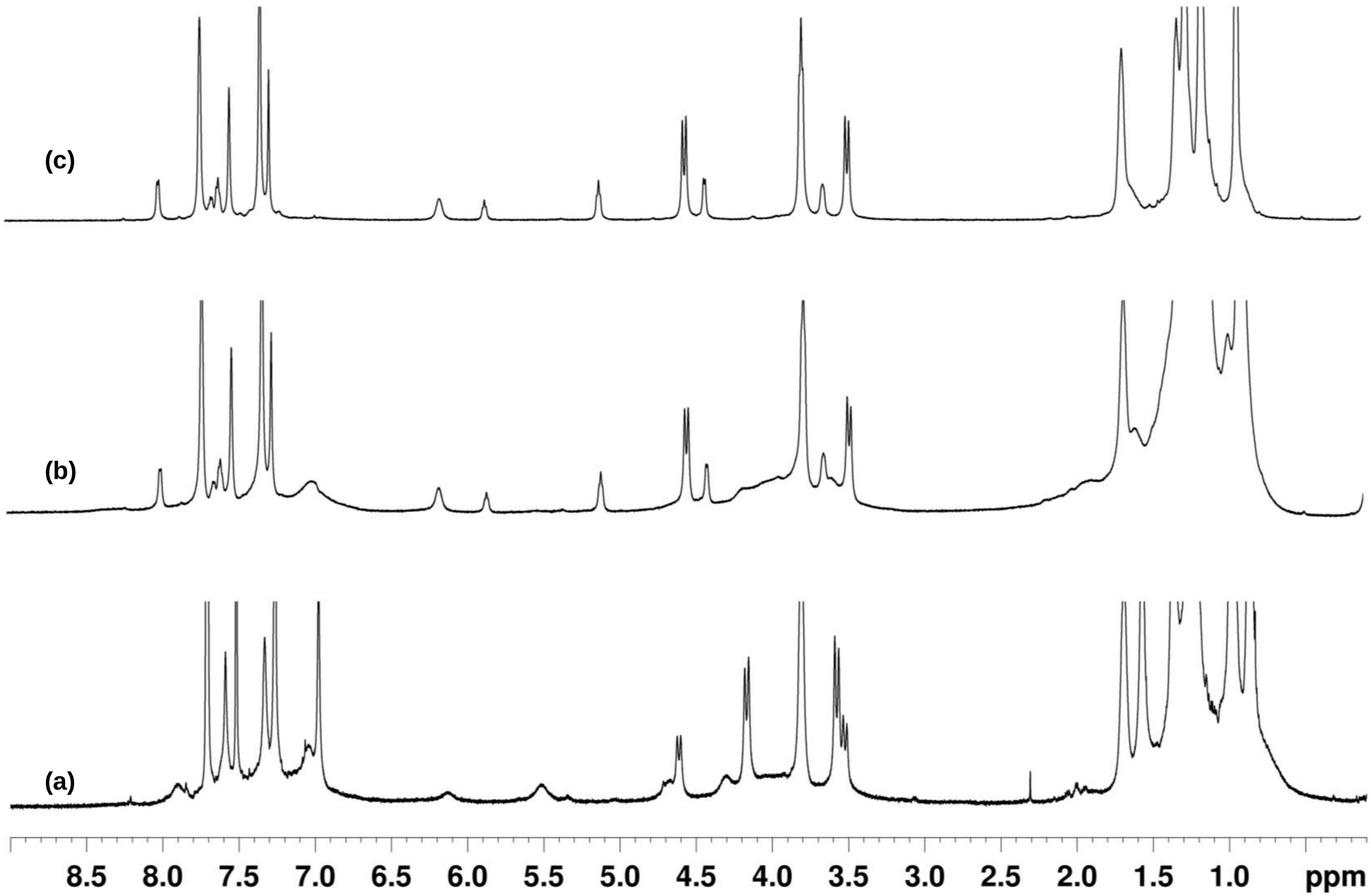

Figure S59. ^{1}H NMR spectrum of **(a)** an equimolar solution (6.1 mM) of $\mathbf{2}^+$ and **1h** in 0.5 mL of $CDCl_3$; **(b)** an equimolar solution (6.1 mM) of $\mathbf{2}^+$, **1h** and **1b** in 0.5 mL of $CDCl_3$. **(c)** an equimolar solution (6.1 mM) of $\mathbf{2}^+$ and **1b** in 0.5 mL of $CDCl_3$ (600 MHz, $CDCl_3$, 298 K).

Computational studies

Non-covalent interactions

NBO second-order perturbation theory analysis was performed via single point energy calculations using the M06-2X/dgdzvp//M06-2X/dgdzvp:PM6 method. The non-covalent interaction (NCI) index was analyzed with the Multiwfn program[1] and its plot was graphed with VMD program.[2] The reduced density gradient (RDG) analysis-based NCI results for **2**$^+$⊂**1f**, and **3**$^+$⊂**1f**. are reported in Figure S60. The strongest interactions associated with the many hydrogen bonds existing between phenolic oxygen and ammonium site.

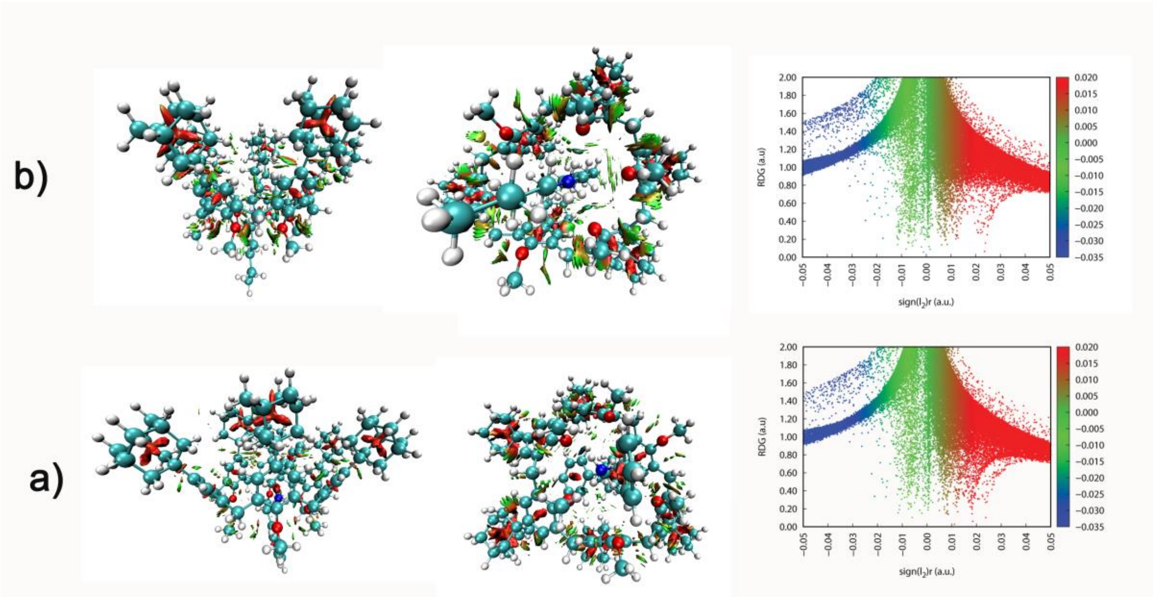

Figure S60. Non-covalent interaction plot and plot of the reduced density gradient (RDG) versus the electron density multiplied by the sign of the second Hessian eigenvalue (gradient isosurfaces (s = 0.6 a.u.) for **2**$^+$ ⊂ **1f** (a) and **3**$^+$ ⊂ **1f** (b). In the coloring scheme blue and green colors represent strong and medium interactions (H-bonding and van der Waals).

---

[1] T. Lu, F. W. Chen, *J. Comput. Chem.* **2012**, *33*, 580-592.

[2] W. Humphrey, A. Dalke, K. Schulten, *J. Mol. Graph.* **1996**, *14*, 33-38.

Table S3. Secondary orbital interactions for $\mathbf{2^{+}\subset 1f}$

Entry	*Type Donor*	*Type Acceptor*	*E(Kcal/mol)*
1	BD (2) C 7 - C 16	/***. BD*(1) C 249 - H 274	0.56
2	BD (2) C 8 - C 64	/***. BD*(1) C 255 - H 261	0.41
3	BD (2) C 13 - C 15	/***. BD*(1) C 255 - H 261	0.08
4	BD (2) C 17 - C 33	/***. BD*(2) C 254 - C 255	0.21
5	BD (2) C 17 - C 33	/***. BD*(1) C 255 - H 261	0.08
6	BD (2) C 18 - C 34	/***. BD*(2) C 251 - C 256	0.09
7	BD (2) C 18 - C 34	/***. BD*(2) C 252 - C 253	0.05
8	BD (2) C 19 - C 40	/***. BD*(1) C 249 - H 274	0.09
9	BD (2) C 22 - C 32	/***. BD*(1) C 251 - H 257	0.22
10	BD (2) C 28 - C 29	/***. BD*(1) C 251 - H 257	0.30
11	BD (1) C 59 - H 61	/***. BD*(1) C 263 - H 269	0.61
12	BD (1) C 59 - H 238	/***. BD*(1) C 263 - H 269	0.08
13	BD (1) C 150 - H 151	/***. BD*(1) C 254 - H 260	0.11
14	BD (1) C 197 - H 207	/***. BD*(1) C 254 - H 260	0.15
15	BD (1) C 239 - H 246	/***. BD*(2) C 263 - C 264	0.17
16	BD (1) C 240 - H 241	/***. BD*(2) C 265 - C 266	0.15
17	CR (1) O 3	/***. BD*(1) N 248 - H 277	0.14
18	LP (1) O 1	/***. BD*(1) C 247 - H 275	0.54
19	LP (1) O 1	/***. BD*(1) N 248 - H 277	0.17
20	LP (2) O 1	/***. BD*(1) C 247 - H 275	0.25
21	LP (1) O 2	/***. BD*(1) C 247 - H 275	0.07
22	LP (1) O 3	/***. BD*(1) N 248 - H 277	0.09
23	LP (2) O 3	/***. BD*(1) N 248 - H 277	10.36
24	LP (1) O 4	/***. BD*(1) N 248 - H 250	0.27
25	LP (2) O 4	/***. BD*(1) N 248 - H 250	8.34
26	LP (1) O 5	/***. BD*(1) C 265 - H 270	1.76
27	LP (2) O 5	/***. BD*(1) C 265 - H 270	0.11
28	LP (1) O 6	/***. BD*(1) C 247 - H 276	2.62
29	LP (2) O 6	/***. BD*(1) C 247 - H 276	0.95
30	BD*(2) C 8 - C 64	/***. BD*(1) C 255 - H 261	0.12
	unit 2 to unit 1		
31	BD (1) N 248 - H 277	/***. BD*(1) O 3 - C 41	0.08
32	BD (1) N 248 - H 277	/***. BD*(1) O 3 - C 239	0.11
33	BD (2) C 251 - C 256	/***. BD*(2) C 41 - C 48	0.05
34	BD (2) C 251 - C 256	/***. BD*(1) C 217 - H 231	0.12
35	BD (2) C 252 - C 253	/***. BD*(1) C 211 - H 219	0.06
36	BD (1) C 252 - H 258	/***. BD*(1) C 96 - H 107	0.06
37	BD (1) C 254 - H 260	/***. BD*(1) C 150 - H 151	0.10
38	BD (1) C 254 - H 260	/***. BD*(1) C 197 - H 207	0.09
39	BD (1) C 255 - H 261	/***. BD*(2) C 8 - C 64	0.07

40	BD (2) C 263 - C 264	/***. BD*(1) C 239 - H 246	0.47
41	BD (1) C 263 - H 269	/***. BD*(1) C 59 - H 61	0.41
42	BD (1) C 263 - H 269	/***. BD*(1) C 239 - H 246	0.14
43	BD (1) C 264 - C 265	/***. RY*(1) H 236	0.07
44	BD (2) C 265 - C 266	/***. BD*(1) C 75 - H 236	1.16
45	BD (2) C 265 - C 266	/***. BD*(1) C 240 - H 241	0.32
46	BD (1) C 265 - H 270	/***. BD*(1) C 240 - H 241	0.07
47	BD*(2) C 251 - C 256	/***. BD*(2) C 18 - C 34	0.70
48	BD*(2) C 251 - C 256	/***. BD*(2) C 19 - C 40	0.12
49	BD*(2) C 252 - C 253	/***. BD*(2) C 57 - C 66	0.26
50	BD*(2) C 263 - C 264	/***. BD*(1) C 239 - H 246	0.31
51	BD*(2) C 265 - C 266	/***. BD*(1) C 240 - H 241	0.17

[a] The numeration is that reported in Figure S61

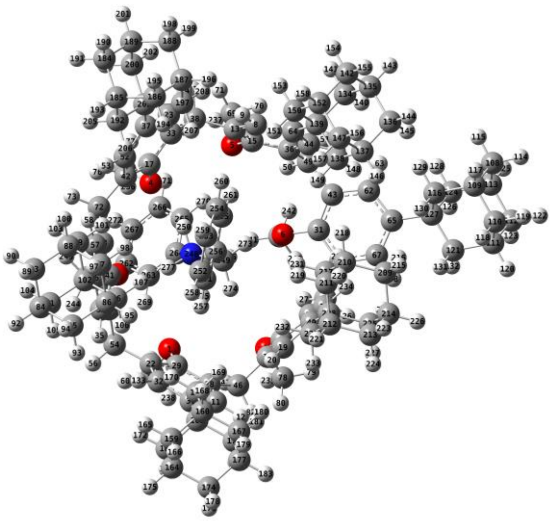

Figure S61. Minimized Structure with labels of **2**$^{+}\subset$**1f**.

Table S4. Secondary orbital interactions for **3**$^{+}\subset$**1f**

Entry	*Type Donor*	*Type Acceptor*	*E(Kcal/mol)*
1	BD (2) C 7 - C 16	***. BD*(1) C 258 - H 262	0,13
2	BD (2) C 8 - C 13	***. BD*(1) C 255 - H 259	0,16
3	BD (2) C 10 - C 14	***. BD*(1) C 258 - H 262	0,16
4	BD (2) C 15 - C 36	***. BD*(1) C 255 - H 260	0,15
5	BD (2) C 17 - C 42	***. BD*(1) C 255 - H 259	0,07
6	BD (2) C 21 - C 31	***. BD*(1) C 255 - H 260	0,17
7	BD (2) C 41 - C 48	***. BD*(1) C 258 - H 263	0,22
8	BD (2) C 57 - C 66	***. BD*(1) C 253 - H 257	0,09

9	BD (1) C 75 - H 76	***. BD*(1) C 265 - H 269	0,06
10	BD (1) C 75 - H 236	***. BD*(1) C 265 - H 269	0,45
11	BD (1) C 87 - H 98	***. BD*(1) C 247 - H 248	0,37
12	BD (1) C 96 - H 107	***. BD*(1) C 253 - H 257	0,06
13	BD (1) C 150 - H 151	***. BD*(1) C 250 - H 254	0,1
14	BD (1) C 210 - H 218	***. BD*(1) C 247 - H 251	0,09
15	BD (1) C 217 - H 231	***. BD*(1) C 253 - H 256	0,22
16	BD (1) C 240 - H 241	***. BD*(1) C 265 - H 270	0,66
17	BD (1) C 240 - H 242	***. BD*(1) C 265 - H 270	0,09
18	CR (1) O 4	***. BD*(1) N 261 - H 280	0,11
19	CR (1) O 6	***. BD*(1) N 261 - H 281	0,15
20	LP (1) O 1	***. BD*(1) C 264 - H 266	0,08
21	LP (1) O 2	***. BD*(1) C 264 - H 266	0,66
22	LP (2) O 2	***. BD*(1) C 264 - H 266	0,09
23	LP (1) O 3	***. BD*(1) C 264 - H 267	0,08
24	LP (1) O 4	***. BD*(1) N 261 - H 280	1,69
25	LP (2) O 4	***. BD*(1) N 261 - H 280	8,68
26	LP (1) O 5	***. BD*(1) N 261 - H 280	0,48
27	LP (1) O 5	***. BD*(1) N 261 - H 281	0,25
28	LP (2) O 5	***. BD*(1) N 261 - H 280	0,2
29	LP (2) O 6	***. BD*(1) N 261 - H 281	11,61
30	BD*(2) C 8 - C 13	***. BD*(1) C 255 - H 259	0,07
31	BD*(2) C 17 - C 42	***. BD*(1) C 255 - H 259	0,07
32	BD*(2) C 21 - C 31	***. BD*(1) C 255 - H 260	0,11
	unit 2 to unit 1		
33	BD (1) C 247 - H 248	***. BD*(1) C 87 - H 98	0,41
34	BD (1) C 247 - H 249	***. BD*(1) C 87 - H 98	0,05
35	BD (1) C 247 - H 251	***. BD*(1) C 210 - H 218	0,09
36	BD (1) C 250 - H 254	***. BD*(1) C 150 - H 151	0,13
37	BD (1) C 253 - H 256	***. BD*(1) C 217 - H 231	0,13
38	BD (1) C 253 - H 257	***. BD*(1) C 96 - H 107	0,07
39	BD (1) C 255 - H 260	***. BD*(2) C 21 - C 31	0,07
40	BD (1) N 261 - H 280	***. BD*(1) O 4 - C 17	0,07
41	BD (1) N 261 - H 280	***. BD*(1) O 4 - C 75	0,07
42	BD (1) N 261 - H 281	***. BD*(1) O 6 - C 31	0,08
43	BD (1) N 261 - H 281	***. BD*(1) O 6 - C 240	0,1
44	BD (1) C 265 - H 269	***. BD*(1) C 75 - H 236	0,5
45	BD (1) C 265 - H 270	***. BD*(1) C 75 - H 236	0,06
46	BD (1) C 265 - H 270	***. BD*(1) C 240 - H 241	0,49
47	BD (1) C 268 - H 272	***. BD*(1) C 59 - H 61	0,07
48	BD (1) C 268 - H 273	***. BD*(1) C 59 - H 61	0,21

The numeration is that reported in Figure S62

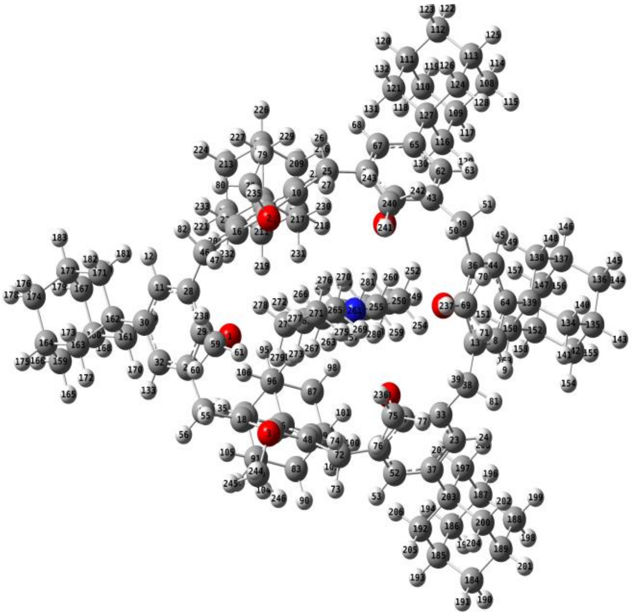

Figure S62. Minimized Structure with labels of **3⁺⊂1f**.

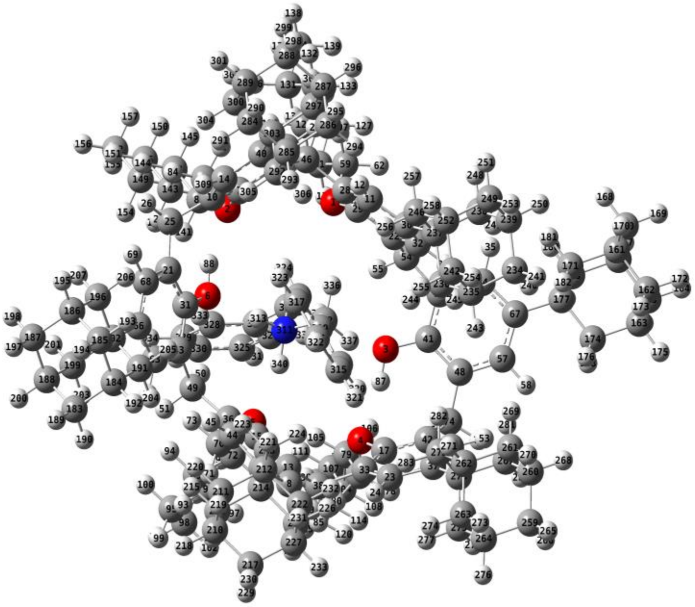

Figure S63. Minimized Structure with labels of **2⁺⊂1g**.

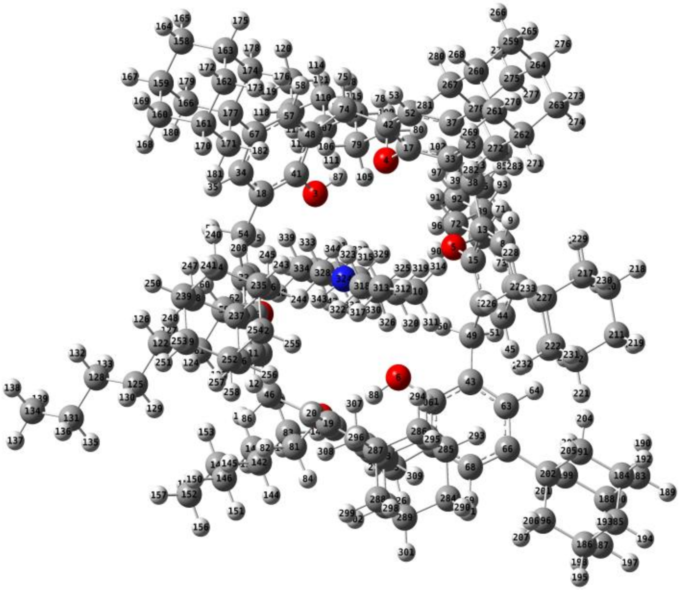

Figure S64. Minimized Structure with labels of **3**$^{+}\subset$ **1g**.

Table S5. Secondary orbital interactions for **2**$^{+}\subset$ **1a**

Entry	*Type Donor*	*Type Acceptor*	*E(Kcal/mol)*
1	BD (2) C 13 - C 16	/***. BD*(2) C 179 - C 184	0,26
2	BD (2) C 13 - C 16	/***. BD*(1) C 179 - H 185	0,11
3	BD (2) C 24 - C 35	/***. BD*(1) C 179 - H 185	1,11
4	BD (2) C 32 - C 45	/***. BD*(2) C 182 - C 183	0,08
5	BD (2) C 38 - C 46	/***. BD*(2) C 180 - C 181	0,08
6	BD (2) C 62 - C 65	/***. BD*(2) C 182 - C 183	0,07
7	BD (1) C 86 - H 89	/***. BD*(1) C 180 - H 186	0,17
8	BD (1) C 90 - H 92	/***. BD*(1) C 193 - H 198	0,07
9	BD (1) C 90 - H 157	/***. BD*(1) C 193 - H 198	0,54
10	BD (1) C 130 - H 131	/***. BD*(1) C 180 - H 186	0,14
11	BD (1) C 134 - H 137	/***. BD*(1) C 182 - H 188	0,05
12	BD (1) C 161 - H 162	/***. BD*(2) C 192 - C 193	0,19
13	BD (1) C 165 - H 167	/***. BD*(2) C 190 - C 191	0,16
14	CR (1) O 5	/***. BD*(1) N 176 - H 205	0,09
15	CR (1) O 6	/***. BD*(1) N 176 - H 178	0,17
16	LP (1) O 1	/***. BD*(1) C 175 - H 204	0,06
17	LP (1) O 2	/***. BD*(1) C 175 - H 204	0,59
18	LP (1) O 2	/***. BD*(1) N 176 - H 178	0,12
19	LP (2) O 2	/***. BD*(1) C 175 - H 204	0,21
20	LP (2) O 3	/***. BD*(1) C 175 - H 203	0,95

21	LP (1) O 4	/***. BD*(1) C 191 - H 197	1,55
22	LP (2) O 4	/***. BD*(1) C 191 - H 197	0,21
23	LP (1) O 5	/***. BD*(1) N 176 - H 205	0,76
24	LP (2) O 5	/***. BD*(1) N 176 - H 205	6,16
25	LP (2) O 6	/***. BD*(1) N 176 - H 178	9,08
26	BD*(2) C 24 - C 35	/***. BD*(1) C 179 - H 185	0,09
	unit 2 to unit 1		
27	BD (1) C 175 - H 203	/***. BD*(1) O 3 - C 43	0,07
28	BD (1) N 176 - H 178	/***. BD*(1) O 6 - C 32	0,1
29	BD (1) N 176 - H 178	/***. BD*(1) O 6 - C 161	0,13
30	BD (1) N 176 - H 205	/***. BD*(1) O 5 - C 16	0,07
31	BD (1) N 176 - H 205	/***. BD*(1) O 5 - C 69	0,08
32	BD (2) C 179 - C 184	/***. BD*(2) C 13 - C 16	0,07
33	BD (2) C 179 - C 184	/***. BD*(2) C 38 - C 46	0,05
34	BD (2) C 179 - C 184	/***. BD*(1) C 75 - H 78	0,18
35	BD (1) C 179 - H 185	/***. BD*(2) C 24 - C 35	0,06
36	BD (1) C 180 - H 186	/***. BD*(1) C 86 - H 89	0,13
37	BD (1) C 180 - H 186	/***. BD*(1) C 130 - H 131	0,09
38	BD (2) C 182 - C 183	/***. BD*(1) C 75 - H 76	0,09
39	BD (2) C 190 - C 191	/***. BD*(1) C 165 - H 167	0,33
40	BD (2) C 192 - C 193	/***. BD*(1) C 161 - H 162	0,61
41	BD (2) C 182 - C 183	/***. BD*(1) C 75 - H 76	0,09
42	BD (2) C 190 - C 191	/***. BD*(1) C 69 - H 70	1,44

[a] The numeration is that reported in Figure S65

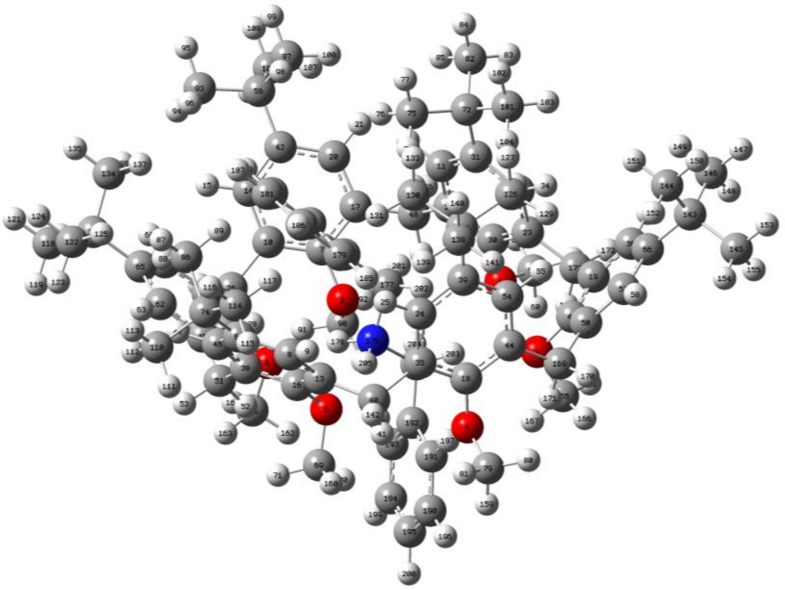

Figure S65. Minimized Structure with labels of **2$^+$**$\subset$**1a**.

Atomic Coordinates

Atomic coordinates of $2^+ \subset 1f$

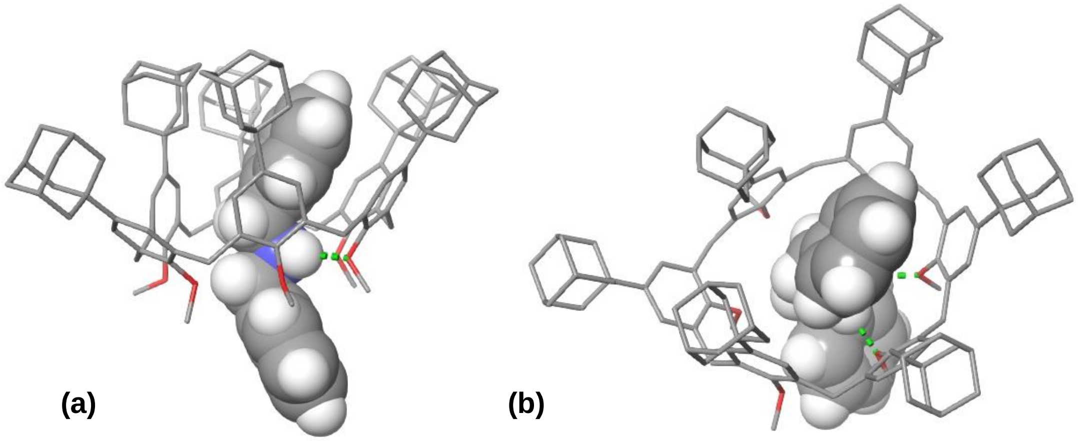

Figure S66. (a) Side view and **(b)** top view of complex **$2^+ \subset$1f.**

O	1.94906300	-3.35100600	3.48040200
O	-1.95177000	-4.54301100	3.35087300
O	3.07972200	-0.09508900	3.71918800
O	0.73302200	2.70082900	3.60103900
O	-3.14748500	2.94777500	3.79683600
O	-3.95509600	-0.77741400	3.37511900
C	-1.89716200	-4.24972600	1.99269400
C	-2.36357500	3.91343300	0.32900900
H	-1.62079600	4.50443000	-0.19161800
C	-2.99881600	-3.62531800	1.37653100
C	2.42832900	-5.30266600	0.39276500
H	1.89260100	-6.06591500	-0.16001800
C	-2.25348300	3.80216000	1.72134100
C	-2.90157700	-3.29781700	0.02298500
C	-3.22105300	3.05162900	2.40903200
C	-0.72597300	-4.53055600	1.27230100
C	1.17167100	3.53768600	2.56595600
C	4.71517800	-0.95911800	2.15928800
C	-0.67981300	-4.18229900	-0.08397200
H	0.22780300	-4.39871100	-0.63098400
C	-5.08842100	-2.17296600	1.76183300
C	3.78468800	-3.37216800	1.89577200
C	0.72060900	5.27120300	0.96701500
H	0.02667500	5.99399800	0.54805000
C	-4.27322300	-3.37880000	2.18206000
H	-4.91202200	-4.26811300	2.09752700
H	-3.98939100	-3.30348300	3.23221100
C	1.81947600	-4.78856700	1.54524100
C	2.52040800	-3.83579200	2.30102300
C	3.68791800	-4.87926200	-0.04632900
C	-4.88070000	-0.91462400	2.34039000
C	4.33539900	-3.90452800	0.72582100
C	0.25654000	4.43371900	1.98446500
C	5.62966300	-0.70262300	1.13530500
H	6.21387200	-1.53308300	0.75238400
C	-4.25413100	2.39012000	1.72019700
C	2.04582700	5.24272700	0.50470800
C	-1.17225100	4.55502200	2.49219000
H	-1.22218400	4.22434800	3.52769900
C	-1.75573000	-3.57577700	-0.73938600
C	4.00088700	0.13063800	2.68671800
C	2.51123800	3.48102600	2.15347000
C	-5.59555900	0.21332900	1.90479100
C	-4.31666300	2.53325400	0.33317100
H	-5.11759700	2.02666700	-0.19420200
C	0.43478900	-5.25880500	1.94826600
H	0.29914900	-5.16705300	3.02519400

C	4.18961000	1.43766300	2.21171700
C	-5.31309600	1.59015600	2.47747700
H	-4.99168300	1.50393900	3.51464400
H	-6.24894500	2.16321100	2.47632500
C	2.91634600	4.33900100	1.12135500
H	3.95694700	4.31928900	0.82450300
C	4.56803000	-2.36616700	2.73239900
H	4.09795500	-2.29987800	3.71037300
H	5.57644200	-2.76864200	2.88432800
C	5.11166600	1.63059100	1.17482200
H	5.27900700	2.64117300	0.82603500
C	2.26902700	-4.15475900	4.62345800
H	3.34000700	-4.11023800	4.85497600
H	1.69921100	-3.75731200	5.46579500
C	-6.56674500	0.03697700	0.91529200
H	-7.14180900	0.90609900	0.60820900
C	-3.39923400	3.30831000	-0.39142000
C	-6.83497700	-1.20951500	0.32798400
C	5.84672000	0.58152200	0.61417700
C	-6.06916300	-2.29515200	0.76697400
H	-6.23471000	-3.28021400	0.34498100
C	-3.89444600	3.94814200	4.49679500
H	-4.96235200	3.89241700	4.25576800
H	-3.53108400	4.95537600	4.26085400
C	3.52818400	2.62611400	2.90489800
H	4.33449100	3.29766600	3.22717300
H	3.04818600	2.25624700	3.80566900
C	0.88624600	3.27896400	4.90929100
H	1.93333500	3.53226500	5.10502900
H	0.28264900	4.18694300	5.00977500
C	-2.46914200	-5.83946100	3.66451600
H	-3.49727000	-5.96018000	3.30386200
H	-1.84828500	-6.63385800	3.23320100
H	-1.43277700	5.62152300	2.48759700
H	0.35653000	-6.32793600	1.71015100
C	9.43331800	1.99809800	-1.51814800
C	9.35781400	0.51828500	-1.09514400
C	8.96774100	-0.34571800	-2.31168300
C	7.59520300	0.10921900	-2.84863800
C	7.67171600	1.58933200	-3.27015400
C	8.05706700	2.44593600	-2.04841900
H	10.19827100	2.13238000	-2.29317300
H	9.73166700	2.62084800	-0.66481200
C	8.29825700	0.35354000	0.01332800
H	10.33179700	0.19348600	-0.70876700
H	8.92991600	-1.40560200	-2.02667300
H	9.72805700	-0.25581500	-3.09756400
H	7.31300100	-0.50765000	-3.71109500
C	6.53156200	-0.05479700	-1.74408800
H	8.41152700	1.71798800	-4.07011000
H	6.70513800	1.91811800	-3.67441900
C	6.99720400	2.27481700	-0.94023200
H	8.09915100	3.50339800	-2.33738800
H	6.02261500	2.62360300	-1.30712200
C	6.89133600	0.79507000	-0.48999800
H	7.26425700	2.90764000	-0.08403100
H	8.57117900	0.95056900	0.89294800
H	8.26889100	-0.69248100	0.34131800
H	6.44789600	-1.11329800	-1.46860600
H	5.54603000	0.25315500	-2.11742400
C	-10.03760100	-0.04849300	-2.41960400
C	-8.69761800	-0.50519000	-3.03191300
C	-8.82721300	-1.95781100	-3.52946700
C	-9.19020400	-2.87111100	-2.34216400
C	-10.53402300	-2.42072500	-1.73667100
C	-10.40499100	-0.96865500	-1.23746800
H	-10.82825100	-0.07760200	-3.17986500
H	-9.96260700	0.99281200	-2.07922800
C	-7.59092500	-0.42623900	-1.96068100
H	-8.43159500	0.15133200	-3.87012200
H	-7.88464000	-2.28683900	-3.98660500
H	-9.59861200	-2.02461000	-4.30693800
H	-9.26999900	-3.90962800	-2.68650600
C	-8.08529900	-2.78635500	-1.26868800
H	-11.33020700	-2.49417300	-2.48815300
H	-10.81659600	-3.08222200	-0.90744400
C	-9.30165800	-0.89073700	-0.16259400
H	-11.35553600	-0.64234300	-0.79750700
H	-9.55760800	-1.52916900	0.69272700
C	-7.92644600	-1.33679300	-0.74236500
H	-9.23299200	0.13442100	0.22061900
H	-7.47363600	0.61311800	-1.62995600
H	-6.62610800	-0.73316300	-2.38646500

H	-7.13577600	-3.13558000	-1.69553600
H	-8.33111700	-3.46027600	-0.43785600
H	5.32045700	-3.56325100	0.42536900
C	-4.94207200	4.22551300	-2.16667000
C	-5.18504400	4.40974700	-3.67801900
C	-5.21245900	3.02916600	-4.36427800
C	-3.86094000	2.31857600	-4.14695300
C	-3.61280500	2.13039600	-2.63600100
C	-3.58683500	3.50210700	-1.90195200
H	-5.76409000	3.64757700	-1.72755700
H	-4.93710900	5.19915800	-1.65984700
C	-4.05122900	5.26147100	-4.28115000
H	-6.14711000	4.91574500	-3.82657100
H	-5.40893600	3.14491200	-5.43767100
H	-6.02956100	2.41994500	-3.95541900
H	-3.88187400	1.33424600	-4.63130900
C	-2.72847200	3.17112300	-4.75102400
H	-4.39617900	1.49077100	-2.21270700
H	-2.65944700	1.61280700	-2.46761200
C	-2.46624500	4.35938400	-2.54391400
H	-1.49120000	3.88056200	-2.38224800
C	-2.70412800	4.54666800	-4.05681000
H	-2.42521000	5.34396300	-2.06019200
H	-4.03523700	6.25507800	-3.81459400
H	-4.22236500	5.41607600	-5.35393700
H	-2.88121600	3.29579400	-5.83051800
H	-1.76298100	2.66412900	-4.62183200
H	-1.89026200	5.15524600	-4.47074400
C	6.69902100	-5.45703900	-3.16827000
C	5.35181700	-4.82761600	-3.57830900
C	4.63158100	-4.29431300	-2.32325000
C	4.36533400	-5.44074200	-1.30303200
C	5.73006400	-6.09391900	-0.93239000
C	6.44782200	-6.62325600	-2.19067900
H	7.34212300	-4.70102900	-2.69804800
H	7.23193400	-5.81807000	-4.05680600
C	4.47217900	-5.89310900	-4.26065200
H	5.52861600	-3.99448100	-4.27024800
H	3.67648500	-3.82908700	-2.60150200
H	5.23971300	-3.51033100	-1.85502800
C	3.50785600	-6.51640100	-2.01798900
H	6.37082900	-5.36352400	-0.42374900
H	5.55844500	-6.91083600	-0.21978700
C	5.56801800	-7.68745800	-2.87463300
H	7.40533300	-7.06907300	-1.89416000
H	5.40025300	-8.53319600	-2.19528300
C	4.22240200	-7.05223300	-3.27611400
H	6.07632800	-8.08700400	-3.76112300
H	4.96441500	-6.26695800	-5.16719500
H	3.51774400	-5.45072100	-4.57551700
H	2.53705400	-6.09100400	-2.30467000
H	3.30451600	-7.34886400	-1.33200800
H	3.58420600	-7.80803300	-3.75018000
C	4.17143200	8.48364600	-1.59334300
C	4.40147600	7.03901000	-2.07867400
C	3.55612500	6.77810100	-3.34061600
C	2.06505400	6.96737700	-3.00093200
C	1.82702200	8.40968700	-2.50911000
C	2.68087300	8.67344700	-1.25207300
H	4.47639100	9.19587400	-2.37026000
H	4.79099500	8.69017500	-0.71099400
C	3.99202800	6.05026500	-0.96715100
H	5.46391100	6.89179500	-2.30912600
H	3.73347200	5.76017300	-3.71231000
H	3.85253700	7.46583300	-4.14247500
H	1.45444800	6.77540100	-3.89219600
C	1.66184900	5.97364100	-1.89250400
H	2.08823800	9.12466700	-3.29927900
H	0.76336700	8.56140600	-2.28267300
C	2.27750700	7.68404000	-0.13984200
H	2.50953700	9.69739800	-0.89770700
H	1.22661500	7.84335700	0.12954000
C	2.49486300	6.21088500	-0.59830500
H	2.86605300	7.87050600	0.76765600
H	4.61587900	6.22349800	-0.08071200
H	4.18577400	5.02231400	-1.30158900
H	1.81542400	4.94180000	-2.23605300
H	0.59197300	6.07862700	-1.67509300
C	-3.10158900	-2.15355200	-4.65580300
C	-1.96313200	-1.37640900	-3.96423300
C	-0.61350100	-1.76902200	-4.59533700
C	-0.39025400	-3.28243500	-4.41179600
C	-1.52289000	-4.06083900	-5.10929800

C	-2.87278200	-3.66833500	-4.47794900
H	-3.13803900	-1.90067400	-5.72320100
H	-4.07097100	-1.86542800	-4.22704000
C	-1.94704400	-1.71503800	-2.45935900
H	-2.12571000	-0.29847800	-4.08567500
H	0.20131900	-1.20553800	-4.12198600
H	-0.60424100	-1.51196900	-5.66235700
H	0.57601600	-3.56900000	-4.84635900
C	-0.37985100	-3.62174700	-2.90723900
H	-1.52802600	-3.83902500	-6.18405600
H	-1.35823000	-5.14147500	-5.00773600
C	-2.85766300	-4.01245100	-2.97477300
H	-3.68407100	-4.22439800	-4.96421700
H	-2.71205300	-5.09116800	-2.83308200
C	-1.72458900	-3.23937900	-2.23611100
H	-3.82930000	-3.76602100	-2.52984200
H	-2.89355000	-1.40299500	-2.00172800
H	-1.15116500	-1.15404800	-1.95321800
H	0.44494800	-3.08747800	-2.41800100
H	-0.19122600	-4.69567700	-2.77884000
H	-3.75104000	-2.81353600	-0.44610800
H	-2.45839500	-5.92470300	4.75314200
H	0.54468100	2.53507800	5.62771400
H	-3.75620900	3.75555500	5.56303600
H	1.98862500	-5.20202900	4.46711100
C	3.64013600	-0.01442600	5.03910600
C	-4.53410300	-0.99586700	4.66946400
H	-3.73396400	-0.87275000	5.40095900
H	-5.32909200	-0.26955900	4.87498700
H	-4.95275600	-2.00514600	4.75330200
H	4.38181000	-0.80352000	5.20304800
H	4.11956000	0.95622700	5.20298200
H	2.81396800	-0.13493500	5.74110100
C	-0.64483400	-0.96304600	4.05705900
N	0.20816500	-0.22437900	3.03556800
C	-0.21293800	-0.63275900	1.62800600
H	0.13453000	0.80059800	3.16289000
C	1.68466500	-0.78639300	-0.02158000
C	2.41078600	-0.28356300	-1.10070500
C	2.08104200	0.95799200	-1.64693600
C	1.02372000	1.69605100	-1.11213900
C	0.29300800	1.19233100	-0.03665400
C	0.62183900	-0.05087600	0.51999800
H	1.93399200	-1.76010000	0.38816600
H	3.22919200	-0.86422100	-1.51325100
H	2.64268000	1.34690700	-2.49123700
H	0.76215000	2.66127400	-1.53321500
H	-0.54430700	1.76145500	0.35651000
C	0.29902300	-0.47302600	7.71937200
C	0.27437000	-0.96284100	6.41278000
C	-0.56850000	-0.39198800	5.45008700
C	-1.39755500	0.67886000	5.81869300
C	-1.37478100	1.16524700	7.12757800
C	-0.52778900	0.59277200	8.07895800
H	0.95005600	-0.93108600	8.45772600
H	0.90164000	-1.80589200	6.13859100
H	-2.05957800	1.13371800	5.08525500
H	-2.02538900	1.98866500	7.40667700
H	-0.51935600	0.96823500	9.09759200
H	-1.25736300	-0.32567900	1.54198500
H	-0.17753200	-1.72364700	1.61745300
H	-0.30645800	-2.00035800	4.02220800
H	-1.66940100	-0.91900300	3.67946700
H	1.21123400	-0.42708500	3.20197000

1 29 1.0 59 1.0
2 7 1.0 78 1.0
3 41 1.0 239 1.0
4 17 1.0 75 1.0
5 15 1.0 69 1.0
6 31 1.0 240 1.0
7 10 1.5 16 1.5
8 9 1.0 13 1.5 64 1.5
9
10 14 1.5 25 1.0
11 12 1.0 28 1.5 30 1.5
12
13 15 1.5 38 1.0
14 40 1.5 234 1.0
15 36 1.5
16 19 1.5 46 1.0
17 33 1.5 42 1.5
18 34 1.5 41 1.5 54 1.0

19 20 1.0 40 1.5
20
21 25 1.0 31 1.5 67 1.5
22 29 1.5 32 1.5 54 1.0
23 24 1.0 33 1.5 37 1.5
24
25 26 1.0 27 1.0
26
27
28 29 1.5 46 1.0
29
30 32 1.5 162 1.0
31 43 1.5
32 133 1.0
33 38 1.0
34 35 1.0 66 1.5
35
36 44 1.5 49 1.0
37 52 1.5 203 1.0
38 39 1.0 81 1.0
39
40 228 1.0
41 48 1.5
42 52 1.5 72 1.0
43 49 1.0 62 1.5
44 45 1.0 64 1.5
45
46 47 1.0 82 1.0
47
48 57 1.5 72 1.0
49 50 1.0 51 1.0
50
51
52 53 1.0
53
54 55 1.0 56 1.0
55
56
57 58 1.0 66 1.5
58
59 60 1.0 61 1.0 238 1.0
60
61
62 63 1.0 65 1.5
63
64 139 1.0
65 67 1.5 127 1.0
66 102 1.0
67 68 1.0
68
69 70 1.0 71 1.0 237 1.0
70
71
72 73 1.0 74 1.0
73
74
75 76 1.0 77 1.0 236 1.0
76
77
78 79 1.0 80 1.0 235 1.0
79
80
81
82
83 84 1.0 88 1.0 89 1.0 90 1.0
84 85 1.0 91 1.0 92 1.0
85 86 1.0 93 1.0 94 1.0
86 87 1.0 95 1.0 96 1.0
87 88 1.0 97 1.0 98 1.0
88 99 1.0 100 1.0
89
90
91 102 1.0 104 1.0 105 1.0
92
93
94
95
96 102 1.0 106 1.0 107 1.0
97
98
99 101 1.0 102 1.0 103 1.0
100
101

102
103
104
105
106
107
108 109 1.0 113 1.0 114 1.0 115 1.0
109 110 1.0 116 1.0 117 1.0
110 111 1.0 118 1.0 119 1.0
111 112 1.0 120 1.0 121 1.0
112 113 1.0 122 1.0 123 1.0
113 124 1.0 125 1.0
114
115
116 127 1.0 129 1.0 130 1.0
117
118
119
120
121 127 1.0 131 1.0 132 1.0
122
123
124 126 1.0 127 1.0 128 1.0
125
126
127
128
129
130
131
132
133
134 135 1.0 139 1.0 140 1.0 141 1.0
135 136 1.0 142 1.0 143 1.0
136 137 1.0 144 1.0 145 1.0
137 138 1.0 146 1.0 147 1.0
138 139 1.0 148 1.0 149 1.0
139 150 1.0
140
141
142 152 1.0 154 1.0 155 1.0
143
144
145
146
147 152 1.0 156 1.0 157 1.0
148
149
150 151 1.0 152 1.0 153 1.0
151
152 158 1.0
153
154
155
156
157
158
159 160 1.0 164 1.0 165 1.0 166 1.0
160 161 1.0 167 1.0 168 1.0
161 162 1.0 169 1.0 170 1.0
162 163 1.0 171 1.0
163 164 1.0 172 1.0 173 1.0
164 174 1.0 175 1.0
165
166
167 177 1.0 179 1.0 180 1.0
168
169
170
171 177 1.0 181 1.0 182 1.0
172
173
174 176 1.0 177 1.0 178 1.0
175
176
177 183 1.0
178
179
180
181
182
183
184 185 1.0 189 1.0 190 1.0 191 1.0

185 186 1.0 192 1.0 193 1.0
186 187 1.0 194 1.0 195 1.0
187 188 1.0 196 1.0 197 1.0
188 189 1.0 198 1.0 199 1.0
189 200 1.0 201 1.0
190
191
192 203 1.0 205 1.0 206 1.0
193
194
195
196
197 203 1.0 207 1.0 208 1.0
198
199
200 202 1.0 203 1.0 204 1.0
201
202
203
204
205
206
207
208
209 210 1.0 214 1.0 215 1.0 216 1.0
210 211 1.0 217 1.0 218 1.0
211 212 1.0 219 1.0 220 1.0
212 213 1.0 221 1.0 222 1.0
213 214 1.0 223 1.0 224 1.0
214 225 1.0 226 1.0
215
216
217 228 1.0 230 1.0 231 1.0
218
219
220
221
222 228 1.0 232 1.0 233 1.0
223
224
225 227 1.0 228 1.0 229 1.0
226
227
228
229
230
231
232
233
234
235
236
237
238
239 244 1.0 245 1.0 246 1.0
240 241 1.0 242 1.0 243 1.0
241
242
243
244
245
246
247 248 1.0 264 1.0 275 1.0 276 1.0
248 249 1.0 250 1.0 277 1.0
249 256 1.0 273 1.0 274 1.0
250
251 252 1.5 256 1.5 257 1.0
252 253 1.5 258 1.0
253 254 1.5 259 1.0
254 255 1.5 260 1.0
255 256 1.5 261 1.0
256
257
258
259
260
261
262 263 1.5 267 1.5 268 1.0
263 264 1.5 269 1.0
264 265 1.5
265 266 1.5 270 1.0
266 267 1.5 271 1.0
267 272 1.0

268
269
270
271
272
273
274
275
276
277

Energy = -5244.36186 a.u.
0 imaginary frequency

Atomic coordinates of $3^{+}\subset$1f

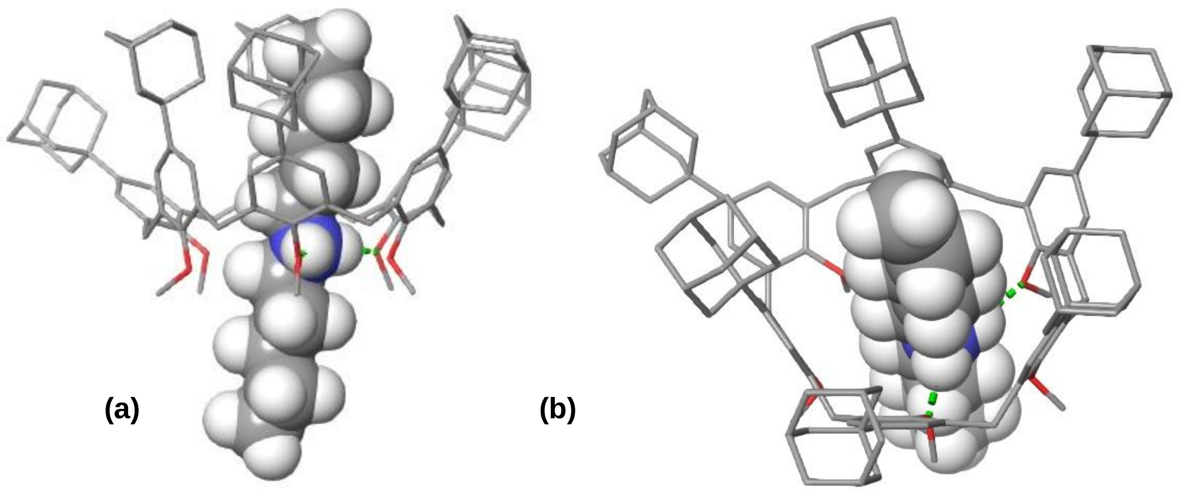

Figure S67. (a) Side view and **(b)** top view of complex **$3^{+}\subset$1f.**

O	-4.00373000	-0.83691400	3.14430300
O	-2.64973200	2.92111300	3.86896100
O	-2.56792600	-3.89509300	3.38674500
O	1.70487800	-2.62620100	3.58958300
O	3.60713800	0.23939300	4.01544900
O	1.48086500	2.79660800	3.76555200
C	-2.71889600	3.06163500	2.48321100
C	5.48703600	-0.84875800	1.04021300
H	5.82665400	-1.77667400	0.59250700
C	-1.68288900	3.73937000	1.81062300
C	-6.53099200	0.67526700	0.93051900
H	-6.92893400	1.66310100	0.72591000
C	4.75972600	-0.92122000	2.23030400
C	-1.75169100	3.82956300	0.41853300
C	4.34356400	0.28191300	2.82906400
C	-3.79191900	2.49036200	1.78296000
C	2.21726200	-3.38333800	2.52229400
C	-3.54230100	-3.46408200	1.21763300
C	-3.81718100	2.61895900	0.38921900
H	-4.64803500	2.17307400	-0.14150100
C	0.82198700	4.42163900	2.09429400
C	-5.48895700	-1.84368200	1.53495800
C	4.05560300	-4.01757800	1.11355800
H	5.10349800	-3.91767600	0.84908200
C	-0.59816700	4.43912800	2.62932900
H	-0.89512700	5.49080300	2.74445100
H	-0.61729500	4.00194300	3.62603300
C	-5.49942900	0.57254700	1.87634800
C	-5.00220000	-0.69768600	2.18559300
C	-7.05900800	-0.43923400	0.27084000
C	1.81346400	3.62144300	2.67941600
C	-6.50584000	-1.69005800	0.59117600
C	3.56381000	-3.22157400	2.15136800
C	-3.37429700	-3.38423600	-0.16602700
H	-4.23979500	-3.11662500	-0.76475700
C	4.63586800	1.52140200	2.24051000
C	3.26879400	-4.96402900	0.44077200
C	4.49153400	-2.26858800	2.89396200
H	4.08842700	-2.08539800	3.88699200
C	-2.81904800	3.29420800	-0.32007600
C	-2.43557900	-3.82080500	2.00262700
C	1.38460600	-4.30934500	1.87737800
C	3.14397000	3.64450300	2.22592600
C	5.36702900	1.53588300	1.04570200
H	5.60555100	2.49901300	0.61141800
C	-4.93278300	1.81207900	2.53832900
H	-4.57403500	1.57013000	3.53857200
C	-1.18102600	-4.07243700	1.41831400
C	4.24022900	2.83417800	2.90776700
H	3.94139100	2.62334200	3.93177900
H	5.13398200	3.46649100	2.95822600

C	1.93811600	-5.09011800	0.85174500
H	1.30048900	-5.83456200	0.38971700
C	-4.91039200	-3.21362400	1.83781400
H	-4.83397800	-3.36000500	2.91526600
H	-5.60930400	-3.97038100	1.46258300
C	-1.05972700	-3.96436000	0.02731300
H	-0.09168000	-4.15423600	-0.41686800
C	-4.51383000	-1.08587100	4.45634500
H	-5.22174100	-1.92195800	4.46397600
H	-3.66047200	-1.34008600	5.08620100
C	3.45555100	4.49460600	1.16174400
H	4.48791600	4.53516800	0.82875900
C	5.81674900	0.36710400	0.42341000
C	2.50525400	5.32305500	0.54639300
C	-2.14979500	-3.64332900	-0.79465500
C	1.19860800	5.27024300	1.04290800
H	0.44170300	5.92696100	0.63108500
C	4.40861500	0.26079700	5.20247100
H	5.01335600	1.17312800	5.26172800
H	5.07982200	-0.60458700	5.25077500
C	-0.04442900	-4.54390100	2.32538500
H	-0.16367200	-5.62707600	2.47026600
H	-0.20695100	-4.09337800	3.30388500
C	1.85600600	-3.27491700	4.86247500
H	1.33610100	-4.23804400	4.87548200
H	2.91213300	-3.43952500	5.10049300
C	-3.33070000	3.94095200	4.60710500
H	-2.91078600	4.93232500	4.39934200
H	-4.40187900	3.95537100	4.37653200
H	5.45364800	-2.77735600	3.02566500
H	-5.74590000	2.54026400	2.65968900
C	-1.57931100	-5.00526900	-4.94211000
C	-3.00606200	-4.69198100	-4.45173700
C	-3.43625800	-3.30251800	-4.96425300
C	-2.46052600	-2.23322300	-4.43218500
C	-1.03576800	-2.54934200	-4.92670000
C	-0.61091700	-3.93462000	-4.40210300
H	-1.54918000	-5.02215200	-6.03891900
H	-1.27244600	-6.00206800	-4.59938700
C	-3.03145400	-4.69922900	-2.91047600
H	-3.70125700	-5.45447800	-4.82459500
H	-4.45934700	-3.07866800	-4.63455400
H	-3.44611800	-3.29084700	-6.06151300
H	-2.76615400	-1.24296600	-4.79417200
C	-2.48358800	-2.23453200	-2.88951600
H	-1.00307100	-2.53542400	-6.02355300
H	-0.33552900	-1.77905700	-4.57851300
C	-0.64284400	-3.93877700	-2.85905000
H	0.41023300	-4.15869000	-4.73611900
H	0.07373100	-3.20047300	-2.47468300
C	-2.06418000	-3.62418900	-2.32733700
H	-0.31691300	-4.92109500	-2.49271000
H	-2.74370600	-5.68754400	-2.52928800
H	-4.05239200	-4.51259000	-2.55746100
H	-3.48781400	-1.97290500	-2.53590600
H	-1.80394900	-1.46537200	-2.49911600
C	5.06581400	7.29824600	-2.40131700
C	3.97354100	6.34269100	-2.92422200
C	2.77813700	7.16331200	-3.44573500
C	2.20913600	8.01856000	-2.29699000
C	3.29763900	8.97998900	-1.78083700
C	4.49291400	8.16019200	-1.25789300
H	5.42422600	7.94138100	-3.21474800
H	5.93176200	6.72444100	-2.04537400
C	3.50447600	5.42252400	-1.77884100
H	4.38036600	5.72475400	-3.73447800
H	2.00243000	6.49354400	-3.84035800
H	3.09400500	7.80776600	-4.27560600
H	1.34793200	8.59481500	-2.65684500
C	1.74673200	7.09890400	-1.14782800
H	3.62144300	9.65092100	-2.58630600
H	2.89458800	9.61427400	-0.98067900
C	4.02533900	7.24437600	-0.10830600
H	5.26959000	8.83693700	-0.88077600
H	3.62947400	7.84548300	0.72036500
C	2.92507600	6.25550100	-0.59706900
H	4.88222000	6.68730100	0.28924500
H	4.34571100	4.81122200	-1.42974400
H	2.73748400	4.72494500	-2.14046100
H	0.94960900	6.43579000	-1.50995500
H	1.31634700	7.71067900	-0.34441900
H	-6.88550800	-2.58396400	0.10402000
C	8.07128900	-0.25429100	-0.53986200

C	8.95032700	-0.29410600	-1.80628000
C	9.16749300	1.14009100	-2.32651500
C	7.80207300	1.77055300	-2.66468900
C	6.92262000	1.80492100	-1.39726200
C	6.67925200	0.37642400	-0.84609300
H	8.56604700	0.32676600	0.24902300
H	7.94325900	-1.27039900	-0.14735700
C	8.24901900	-1.13492400	-2.89228100
H	9.91738800	-0.74718000	-1.55517900
H	9.80815700	1.12737500	-3.21714900
H	9.68617700	1.74266100	-1.56958600
H	7.94826600	2.79751900	-3.02176200
C	7.10536600	0.93579700	-3.75668300
H	7.41462500	2.42137400	-0.63380600
H	5.96181100	2.28387000	-1.62910000
C	6.00313200	-0.45636900	-1.97416800
H	5.02609100	-0.01709600	-2.21161400
C	6.88729200	-0.49835800	-3.23714500
H	5.81161500	-1.47854100	-1.62563500
H	8.10629700	-2.16519500	-2.53980900
H	8.87713500	-1.19112800	-3.79017200
H	7.71656400	0.92065500	-4.66778400
H	6.14301700	1.39086300	-4.02591000
H	6.38327000	-1.09793400	-4.00554200
C	-10.04784100	-1.70073200	-2.66647500
C	-8.85929400	-0.85624200	-3.16994300
C	-7.71972800	-0.90292700	-2.13176500
C	-8.19256900	-0.34191800	-0.75788000
C	-9.41908000	-1.17738800	-0.28433400
C	-10.55501000	-1.13008300	-1.32636100
H	-9.74113900	-2.74745800	-2.53987800
H	-10.85571800	-1.69431700	-3.40903400
C	-9.31264800	0.60300700	-3.36900000
H	-8.49373400	-1.26351200	-4.12108800
H	-6.85959200	-0.31805600	-2.48393800
H	-7.37084800	-1.93610200	-2.01482600
C	-8.67479300	1.11366000	-0.98551900
H	-9.11976700	-2.21818900	-0.11234000
H	-9.77063500	-0.78848800	0.68000800
C	-11.00729000	0.32925300	-1.52682300
H	-11.39747000	-1.73233400	-0.96406100
H	-11.39052000	0.73949300	-0.58343900
C	-9.81353200	1.16731900	-2.02512700
H	-11.82977300	0.37554600	-2.25175300
H	-10.11050200	0.65309200	-4.12068800
H	-8.47935300	1.20969300	-3.74778300
H	-7.83745700	1.73518300	-1.32971300
H	-9.02578700	1.54325200	-0.03830500
H	-10.12461000	2.21122800	-2.15611800
C	4.74063700	-8.44843100	-1.82275600
C	3.53126200	-7.67826400	-2.38782600
C	3.99836800	-6.74291900	-3.52012900
C	5.02232000	-5.73706900	-2.95893000
C	6.23391500	-6.50055000	-2.38632000
C	5.76416200	-7.44357800	-1.25987200
H	5.20119900	-9.05809500	-2.61015700
H	4.41544700	-9.13925100	-1.03407900
C	2.87874300	-6.84391900	-1.26590700
H	2.79063500	-8.38775600	-2.77688100
H	3.14012900	-6.20933000	-3.94960600
H	4.44807300	-7.32811000	-4.33200100
H	5.35456800	-5.06206400	-3.75760600
C	4.36435900	-4.90463300	-1.83969200
H	6.72540600	-7.07710700	-3.17990700
H	6.97939200	-5.79229000	-2.00065700
C	5.10719900	-6.61585100	-0.13642100
H	6.62530400	-7.98368800	-0.84736800
H	5.84656100	-5.92788600	0.29073100
C	3.88154700	-5.81972800	-0.67535400
H	4.78139900	-7.27326500	0.67991100
H	2.51883800	-7.51398400	-0.47434200
H	2.00153200	-6.31971900	-1.66755100
H	3.50945100	-4.34126700	-2.23581600
H	5.08020900	-4.16450000	-1.46074800
C	-1.68981900	4.66566300	-4.32292700
C	-1.65702100	3.14746300	-4.05353400
C	-2.91278900	2.49069400	-4.65861600
C	-4.16865800	3.10166600	-4.00736700
C	-4.20686300	4.61727100	-4.28485700
C	-2.95185300	5.27594900	-3.68008100
H	-1.68597000	4.85684300	-5.40342800
H	-0.78867900	5.14190100	-3.91295400
C	-1.62838500	2.89436200	-2.53209000

H	-0.75719800	2.71203900	-4.50706100
H	-2.88913500	1.40551300	-4.49117700
H	-2.93605900	2.64566600	-5.74468400
H	-5.06705200	2.62787200	-4.42234600
C	-4.13447300	2.85510700	-2.48463900
H	-4.24938700	4.80275000	-5.36555200
H	-5.11247600	5.05937200	-3.84970800
C	-2.92363000	5.02785100	-2.15840800
H	-2.97505900	6.35698500	-3.86595500
H	-3.80796600	5.47173500	-1.68353300
C	-2.88176600	3.50292300	-1.83999900
H	-2.04987700	5.52551400	-1.72033700
H	-0.71533100	3.32616600	-2.10380000
H	-1.59467500	1.81578900	-2.33012700
H	-4.14013500	1.77521700	-2.28741500
H	-5.04362400	3.26896000	-2.02989900
H	-0.95507700	4.34734600	-0.10264500
H	-3.19435100	3.70634000	5.66509100
H	1.41382800	-2.61739700	5.61320500
H	3.71926700	0.22962500	6.04953200
H	-5.02065300	-0.20146900	4.86376900
C	-2.94342600	-5.18412000	3.88037200
C	1.64419800	3.44021100	5.03920400
H	1.49723700	2.67828700	5.80637200
H	2.64823500	3.86376400	5.13960200
H	0.90821100	4.24012300	5.17317500
H	-3.01852500	-5.09393000	4.96632600
H	-3.91280400	-5.50021400	3.47699100
H	-2.19581800	-5.94723800	3.63374800
C	1.66859000	0.34147500	-2.96855000
H	1.23781900	-0.62311400	-3.25825300
H	2.55452800	0.50673700	-3.59004100
C	2.02201300	0.36516500	-1.47973400
H	0.93849200	1.11894900	-3.21836500
H	2.49313700	1.32450600	-1.22798100
C	0.81360300	0.14744800	-0.55987100
H	2.77471200	-0.40481900	-1.26842600
C	1.21334100	0.16465500	0.92090500
H	0.06444600	0.92775000	-0.74550700
H	0.33262900	-0.81085900	-0.79843800
C	0.01529300	-0.00837200	1.84645900
H	1.94202100	-0.63245400	1.10991200
H	1.71543900	1.11274100	1.14734500
N	0.43888500	0.03800900	3.29493000
H	-0.72154600	0.78754000	1.71087800
H	-0.48161800	-0.97049400	1.69555200
C	-0.70143100	-0.16648800	4.25757700
C	-0.25393500	0.04661600	5.70167600
H	-1.49024300	0.53730800	3.98232700
H	-1.07421500	-1.17995700	4.08766000
C	-1.34961100	-0.30954300	6.71499600
H	0.64387600	-0.55130600	5.90776100
H	0.03095400	1.09643800	5.83554000
C	-0.92496700	-0.06674700	8.16924100
H	-2.24937700	0.27999300	6.49542600
H	-1.63190300	-1.36401900	6.59035700
C	-2.01735600	-0.42619400	9.18015800
H	-0.01944300	-0.65012700	8.38452100
H	-0.64391500	0.98786300	8.29198300
H	-1.68466100	-0.24113800	10.20563000
H	-2.92356400	0.16623000	9.01300200
H	-2.29431700	-1.48359700	9.10675400
H	1.14326700	-0.70320900	3.45354400
H	0.89955000	0.94743000	3.48556800

1 29 1.0 59 1.0
2 7 1.0 78 1.0
3 41 1.0 239 1.0
4 17 1.0 75 1.0
5 15 1.0 69 1.0
6 31 1.0 240 1.0
7 10 1.5 16 1.5
8 9 1.0 13 1.5 64 1.5
9
10 14 1.5 25 1.0
11 12 1.0 28 1.5 30 1.5
12
13 15 1.5 38 1.0
14 40 1.5 234 1.0
15 36 1.5
16 19 1.5 46 1.0
17 33 1.5 42 1.5
18 34 1.5 41 1.5 54 1.0

19 20 1.0 40 1.5
20
21 25 1.0 31 1.5 67 1.5
22 29 1.5 32 1.5 54 1.0
23 24 1.0 33 1.5 37 1.5
24
25 26 1.0 27 1.0
26
27
28 29 1.5 46 1.0
29
30 32 1.5 162 1.0
31 43 1.5
32 133 1.0
33 38 1.0
34 35 1.0 66 1.5
35
36 44 1.5 49 1.0
37 52 1.5 203 1.0
38 39 1.0 81 1.0
39
40 228 1.0
41 48 1.5
42 52 1.5 72 1.0
43 49 1.0 62 1.5
44 45 1.0 64 1.5
45
46 47 1.0 82 1.0
47
48 57 1.5 72 1.0
49 50 1.0 51 1.0
50
51
52 53 1.0
53
54 55 1.0 56 1.0
55
56
57 58 1.0 66 1.5
58
59 60 1.0 61 1.0 238 1.0
60
61
62 63 1.0 65 1.5
63
64 139 1.0
65 67 1.5 127 1.0
66 102 1.0
67 68 1.0
68
69 70 1.0 71 1.0 237 1.0
70
71
72 73 1.0 74 1.0
73
74
75 76 1.0 77 1.0 236 1.0
76
77
78 79 1.0 80 1.0 235 1.0
79
80
81
82
83 84 1.0 88 1.0 89 1.0 90 1.0
84 85 1.0 91 1.0 92 1.0
85 86 1.0 93 1.0 94 1.0
86 87 1.0 95 1.0 96 1.0
87 88 1.0 97 1.0 98 1.0
88 99 1.0 100 1.0
89
90
91 102 1.0 104 1.0 105 1.0
92
93
94
95
96 102 1.0 106 1.0 107 1.0
97
98
99 101 1.0 102 1.0 103 1.0
100
101

102
103
104
105
106
107
108 109 1.0 113 1.0 114 1.0 115 1.0
109 110 1.0 116 1.0 117 1.0
110 111 1.0 118 1.0 119 1.0
111 112 1.0 120 1.0 121 1.0
112 113 1.0 122 1.0 123 1.0
113 124 1.0 125 1.0
114
115
116 127 1.0 129 1.0 130 1.0
117
118
119
120
121 127 1.0 131 1.0 132 1.0
122
123
124 126 1.0 127 1.0 128 1.0
125
126
127
128
129
130
131
132
133
134 135 1.0 139 1.0 140 1.0 141 1.0
135 136 1.0 142 1.0 143 1.0
136 137 1.0 144 1.0 145 1.0
137 138 1.0 146 1.0 147 1.0
138 139 1.0 148 1.0 149 1.0
139 150 1.0
140
141
142 152 1.0 154 1.0 155 1.0
143
144
145
146
147 152 1.0 156 1.0 157 1.0
148
149
150 151 1.0 152 1.0 153 1.0
151
152 158 1.0
153
154
155
156
157
158
159 160 1.0 164 1.0 165 1.0 166 1.0
160 161 1.0 167 1.0 168 1.0
161 162 1.0 169 1.0 170 1.0
162 163 1.0 171 1.0
163 164 1.0 172 1.0 173 1.0
164 174 1.0 175 1.0
165
166
167 177 1.0 179 1.0 180 1.0
168
169
170
171 177 1.0 181 1.0 182 1.0
172
173
174 176 1.0 177 1.0 178 1.0
175
176
177 183 1.0
178
179
180
181
182
183
184 185 1.0 189 1.0 190 1.0 191 1.0

185 186 1.0 192 1.0 193 1.0
186 187 1.0 194 1.0 195 1.0
187 188 1.0 196 1.0 197 1.0
188 189 1.0 198 1.0 199 1.0
189 200 1.0 201 1.0
190
191
192 203 1.0 205 1.0 206 1.0
193
194
195
196
197 203 1.0 207 1.0 208 1.0
198
199
200 202 1.0 203 1.0 204 1.0
201
202
203
204
205
206
207
208
209 210 1.0 214 1.0 215 1.0 216 1.0
210 211 1.0 217 1.0 218 1.0
211 212 1.0 219 1.0 220 1.0
212 213 1.0 221 1.0 222 1.0
213 214 1.0 223 1.0 224 1.0
214 225 1.0 226 1.0
215
216
217 228 1.0 230 1.0 231 1.0
218
219
220
221
222 228 1.0 232 1.0 233 1.0
223
224
225 227 1.0 228 1.0 229 1.0
226
227
228
229
230
231
232
233
234
235
236
237
238
239 244 1.0 245 1.0 246 1.0
240 241 1.0 242 1.0 243 1.0
241
242
243
244
245
246
247 248 1.0 249 1.0 250 1.0 251 1.0
248
249
250 252 1.0 253 1.0 254 1.0
251
252
253 255 1.0 256 1.0 257 1.0
254
255 258 1.0 259 1.0 260 1.0
256
257
258 261 1.0 262 1.0 263 1.0
259
260
261 264 1.0 280 1.0 281 1.0
262
263
264 265 1.0 266 1.0 267 1.0
265 268 1.0 269 1.0 270 1.0
266
267

268 271 1.0 272 1.0 273 1.0
269
270
271 274 1.0 275 1.0 276 1.0
272
273
274 277 1.0 278 1.0 279 1.0
275
276
277
278
279
280
281

Energy = -5096.78470 a.u.
0 imaginary frequency

Atomic coordinates of $2^{+}\subset$1g

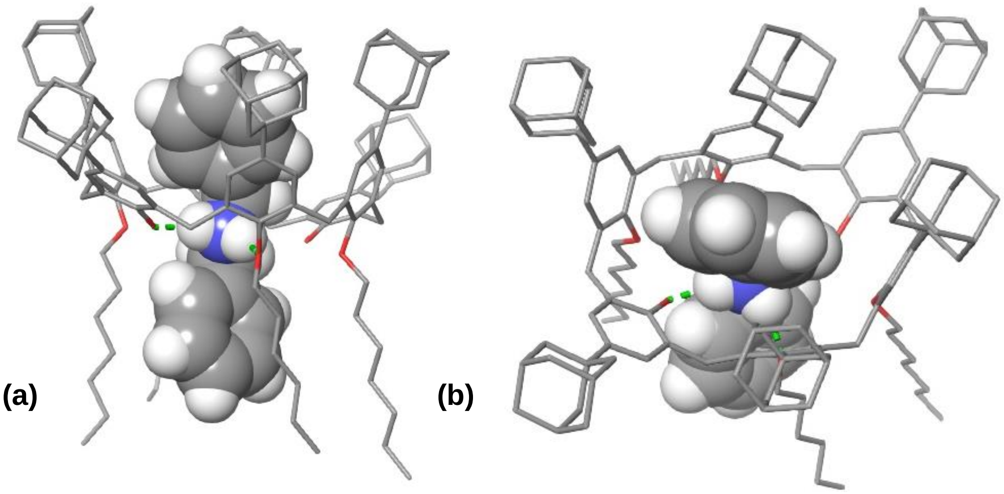

Figure S68. (a) Side view and **(b)** top view of complex **$2^{+}\subset$1g.**

O	-2.93958800	2.86748200	-2.22744000
O	0.81715800	4.19662500	-1.54954800
O	-3.62773400	-0.55038100	0.91983600
O	-2.75495000	-1.49205700	3.46595000
O	0.42874700	0.54486000	3.75862500
O	1.96381200	2.99592300	0.65247500
C	1.58237800	3.67442700	-2.60399100
C	2.40311800	-2.55453000	3.80616000
H	2.26553100	-3.58578100	4.10675100
C	2.98118000	3.70337600	-2.49368300
C	-0.70834900	0.52787100	-3.99869600
H	0.21168800	0.56356300	-4.56585400
C	1.29576000	-1.69731600	3.87914000
C	3.73675700	3.16055500	-3.53551300
C	1.50279100	-0.33850400	3.59623100
C	0.94262200	3.12878300	-3.72620700
C	-2.20493200	-2.70138100	3.02630300
C	-4.76760800	-0.90064900	-1.13902600
C	1.75169500	2.64277500	-4.76367500
H	1.25674300	2.27140400	-5.65325100
C	4.19577000	3.44158100	-0.20074600
C	-3.05991300	0.47284800	-2.48322200
C	-0.38123700	-4.24334200	2.82919400
H	0.63069900	-4.53055500	3.09008600
C	3.67963400	4.35505300	-1.30631700
H	4.53822700	4.91215700	-1.69169800
H	3.01949400	5.10763600	-0.86259500
C	-1.22859900	1.73586100	-3.51174700
C	-2.41551000	1.69554600	-2.76267600
C	-1.33436700	-0.70135900	-3.77637000
C	3.34104900	2.85249200	0.74342200

C	-2.50745400	-0.69735600	-3.00410500
C	-0.88171500	-3.04849300	3.35689700
C	-5.55812300	-1.71433300	-1.95427800
H	-5.85300600	-1.31477000	-2.92098300
C	2.75214900	0.15021500	3.16539200
C	-1.13123400	-5.08978200	1.99661100
C	-0.06379500	-2.20906200	4.34127600
H	-0.66787500	-1.35466300	4.63677400
C	3.15042900	2.63222700	-4.69481300
C	-4.38770500	-1.38221700	0.12705900
C	-2.99996800	-3.52412100	2.21545500
C	3.85863000	2.15292700	1.84992300
C	3.80345400	-0.76217200	3.06112800
H	4.76579700	-0.39830400	2.72312800
C	-0.57170800	3.07668700	-3.84243500
H	-1.02329300	3.83000700	-3.19854200
C	-4.78921600	-2.66189800	0.54513400
C	2.94999700	1.65627300	2.96268700
H	1.97167200	2.11872600	2.84350400
H	3.34857100	2.05653800	3.90553500
C	-2.44203000	-4.70069100	1.70394900
H	-3.06903800	-5.32311300	1.07582200
C	-4.33405700	0.46117600	-1.63761600
H	-4.20474200	1.15134000	-0.80029900
H	-5.15347300	0.87828100	-2.23738700
C	-5.56761400	-3.44174500	-0.31985800
H	-5.87037900	-4.42161100	0.03223600
C	-3.90571800	3.53810400	-3.06166100
H	-3.41515300	3.89115700	-3.97967900
C	-4.49790700	4.70125500	-2.28137200
H	-4.69037600	2.83045300	-3.35964900
C	5.24821400	2.05417500	1.96967900
H	5.64396900	1.57487200	2.85961000
C	3.67000200	-2.11589900	3.40873200
C	6.14157500	2.57890700	1.02555400
C	-5.97476800	-2.99971400	-1.58195000
C	5.57754400	3.27271900	-0.04830300
H	6.21997700	3.74025100	-0.78584600
C	0.47597400	1.33737800	4.98022100
H	1.17457800	0.85366500	5.67120700
C	-0.90192000	1.46331500	5.61021700
H	0.87157200	2.33024600	4.73842000
C	-4.46211500	-3.19601600	1.93141700
H	-5.04128100	-4.11328600	2.07417200
H	-4.83439700	-2.49360800	2.68277000
C	-3.47619700	-1.55433800	4.72228000
H	-4.04713400	-2.48969400	4.76536600
C	-4.39564000	-0.34739200	4.82464800
H	-2.75108800	-1.57117600	5.54601000
C	0.44670700	5.59304500	-1.72353400
H	0.01938400	5.71446600	-2.72497100
C	-0.55505800	5.98450300	-0.65187000
H	1.35103300	6.21317200	-1.67281200
H	0.09518200	-2.82005700	5.23925300
H	-0.83834300	3.34218300	-4.87400500
H	-3.49686800	-0.91004500	1.81924000
H	1.68854500	3.55766500	-0.10617300
C	-0.86367900	2.35654200	6.85929200
H	-1.59819200	1.87978000	4.87480800
H	-1.27158500	0.46691500	5.87975800
C	-2.22387700	2.48822600	7.55483400
H	-0.13061500	1.95713600	7.57421300
H	-0.50410600	3.35583100	6.57855100
C	-2.18821900	3.38140400	8.80114400
H	-2.95700600	2.88915800	6.84078200
H	-2.58635100	1.48928900	7.83689400
C	-3.54827000	3.50659900	9.49363700
H	-1.45142500	2.98189600	9.51073200
H	-1.82647100	4.37974700	8.51986800
H	-4.29926500	3.93609000	8.82068600
H	-3.91838700	2.52826600	9.82059500
H	-3.48883900	4.14965900	10.37699800
C	-5.18300400	-0.32145100	6.14165300
H	-3.79601400	0.56516100	4.72601100
H	-5.09280300	-0.35822300	3.97634400
C	-6.13384200	0.87724600	6.25043800
H	-5.76025200	-1.25100000	6.24172800
H	-4.48184600	-0.30571100	6.98747500
C	-6.92671000	0.91017100	7.56232800
H	-5.55532100	1.80702000	6.15212200
H	-6.83338900	0.86231300	5.40277400
C	-7.87854300	2.10769000	7.67257800
H	-7.50284300	-0.02060500	7.66178000

H	-6.22673800	0.92515000	8.40986900
C	-8.66992200	2.13105300	8.98341300
H	-7.30136300	3.03775100	7.57654300
H	-8.57562000	2.09456000	6.82372000
H	-9.33830300	2.99634300	9.03102300
H	-9.28457700	1.23048100	9.09133500
H	-8.00102600	2.17974800	9.84994100
C	-5.54961800	5.47563800	-3.08637800
H	-4.94144800	4.31411500	-1.35560100
H	-3.68649400	5.37703400	-1.98132500
C	-6.15273900	6.65599400	-2.31436400
H	-5.09956500	5.84493900	-4.01848400
H	-6.35505400	4.79189900	-3.38838400
C	-7.20395000	7.43477900	-3.11397500
H	-6.60404700	6.28648500	-1.38274600
H	-5.34707300	7.33980600	-2.01058700
C	-7.80867900	8.61565700	-2.34447800
H	-6.75236700	7.80316100	-4.04613300
H	-8.00889700	6.75071500	-3.41811800
C	-8.85690500	9.38807600	-3.15057600
H	-8.26075900	8.24677400	-1.41372700
H	-7.00389400	9.29882000	-2.04016300
H	-9.26867900	10.22264300	-2.57457600
H	-8.42603900	9.79972300	-4.07015400
H	-9.69182700	8.73983000	-3.43924100
H	-1.43113800	5.32941800	-0.73185800
H	-0.11669200	5.80724900	0.33761000
C	-0.98314100	7.45435900	-0.77017900
C	-2.01773500	7.86551900	0.28530800
H	-0.09931500	8.10145500	-0.68415300
H	-1.39632000	7.63667000	-1.77210800
C	-2.43512400	9.33754900	0.18629500
H	-2.90695400	7.22707400	0.18743000
H	-1.61279900	7.66640800	1.28737300
C	-3.47216400	9.75191800	1.23758400
H	-2.83887600	9.53449900	-0.81717100
H	-1.54540300	9.97568500	0.28409100
C	-3.88385300	11.22316600	1.13209800
H	-4.36122700	9.11429400	1.13864600
H	-3.06820000	9.55525800	2.24011200
H	-4.62319700	11.48688400	1.89473000
H	-3.02167100	11.88678300	1.26182100
H	-4.32418400	11.44257800	0.15299900
C	-9.37102700	-5.31269700	-3.16591800
C	-9.07860200	-3.92266900	-3.76394900
C	-8.33501800	-4.08630600	-5.10519300
C	-7.00853700	-4.83807300	-4.87142800
C	-7.30248400	-6.22714900	-4.27280700
C	-8.04110200	-6.05579400	-2.93110600
H	-10.01140500	-5.89028400	-3.84453700
H	-9.91948800	-5.21001800	-2.22046400
C	-8.20116000	-3.11597100	-2.78511900
H	-10.02068900	-3.38447200	-3.92739400
H	-8.13817700	-3.10224700	-5.55094900
H	-8.96022000	-4.63852500	-5.81821300
H	-6.47528500	-4.95046100	-5.82386800
C	-6.12780600	-4.03441500	-3.89319100
H	-7.91308400	-6.81818600	-4.96712100
H	-6.36589600	-6.78002600	-4.12205000
C	-7.16352700	-5.24435200	-1.95557200
H	-8.24171300	-7.04094200	-2.49181400
H	-6.22932400	-5.78816000	-1.76223300
C	-6.84582200	-3.83805300	-2.52528800
H	-7.68479500	-5.14903800	-0.99421700
H	-8.72471400	-2.97633100	-1.83053400
H	-8.01814000	-2.11385400	-3.19185300
H	-5.88632500	-3.05951300	-4.33360100
H	-5.17287600	-4.55156400	-3.72889900
C	9.94739600	1.43996600	2.86561600
C	9.55372700	0.74850100	1.54427100
C	10.33254000	1.38524900	0.37710500
C	9.98939500	2.88555700	0.29664200
C	10.39181400	3.57831200	1.61346100
C	9.61209800	2.94307500	2.78108000
H	11.01894600	1.30293000	3.05781800
H	9.41253100	0.97984400	3.70713600
C	8.04010800	0.92160800	1.30517100
H	9.78664100	-0.32236300	1.60419300
H	10.07532100	0.88619000	-0.56644400
H	11.41174200	1.25212600	0.52391800
H	10.52975000	3.34328100	-0.54120300
C	8.47385400	3.05908900	0.06591300
H	11.47172200	3.47633100	1.77886300

H	10.17840000	4.65362800	1.55643000
C	8.09808100	3.11975200	2.54535700
H	9.88724100	3.43613800	3.72173300
H	7.84163700	4.18599000	2.49930700
C	7.65548500	2.42819500	1.22172300
H	7.54224000	2.69975600	3.39255300
H	7.48323000	0.43874500	2.11787200
H	7.74070500	0.41924900	0.37599100
H	8.19158500	2.59408500	-0.88798100
H	8.24139000	4.12875400	-0.01601400
H	-3.01316900	-1.63284200	-2.79656400
C	5.94428300	-2.50320300	4.44084100
C	7.21330300	-3.37937500	4.44716500
C	7.85239000	-3.36760300	3.04381800
C	6.84865500	-3.92863000	2.01623800
C	5.57763200	-3.05497700	2.00544400
C	4.90334700	-3.03189300	3.40759900
H	6.21181900	-1.46663400	4.20205200
H	5.48725300	-2.48729900	5.43871000
C	6.83843900	-4.82457500	4.82869600
H	7.92271400	-2.97523300	5.18004200
H	8.76998400	-3.96932200	3.04199700
H	8.14253900	-2.34465600	2.76880500
H	7.29928500	-3.91541000	1.01572400
C	6.47429100	-5.37361000	2.39844100
H	5.83326000	-2.03624900	1.69130000
H	4.86239700	-3.43853000	1.26783800
C	4.56661900	-4.49564600	3.79309200
H	3.83469200	-4.90956900	3.08609000
C	5.83287300	-5.37720700	3.79941500
H	4.10428200	-4.52431500	4.78808900
H	6.40207100	-4.84927300	5.83567600
H	7.73620600	-5.45482300	4.85513200
H	7.36648900	-6.01225800	2.38990300
H	5.77646800	-5.79168800	1.66080400
H	5.54971800	-6.40103300	4.07359000
C	-1.16438200	-4.95360100	-4.80542600
C	-0.10874700	-4.41498000	-3.81872800
C	-0.58771600	-3.07122700	-3.23263700
C	-0.80773700	-2.01951200	-4.35926100
C	-1.84581400	-2.59524200	-5.36864100
C	-1.36252500	-3.93952900	-5.95035900
H	-2.11573100	-5.12512700	-4.28426600
H	-0.84414600	-5.92204800	-5.21061400
C	1.22829900	-4.20603500	-4.55533000
H	0.02952900	-5.13241300	-2.99888400
H	0.14800400	-2.68729900	-2.51585800
H	-1.51939600	-3.22794300	-2.67657200
C	0.53649300	-1.84773000	-5.11209200
H	-2.81290900	-2.73280700	-4.87001100
H	-2.00819300	-1.86857800	-6.17528400
C	-0.02523700	-3.73102300	-6.68756700
H	-2.11668700	-4.31744100	-6.65195600
H	-0.15790100	-3.02712900	-7.51955200
C	1.02354800	-3.19013900	-5.69579900
H	0.31930000	-4.67757700	-7.12313000
H	1.59342100	-5.15901500	-4.95929800
H	1.99049500	-3.84036600	-3.85480500
H	1.29564400	-1.44381800	-4.42956900
H	0.41692100	-1.12379900	-5.92887700
H	1.97457800	-3.02534400	-6.21842500
C	-0.49291800	-9.38916500	1.28876900
C	-0.83373000	-8.50368100	0.07399000
C	0.45395100	-8.18716800	-0.71087600
C	1.43548600	-7.43359000	0.20711300
C	1.77810300	-8.31181300	1.42785700
C	0.48786900	-8.63580200	2.20828300
H	-0.04819200	-10.33474400	0.95402500
H	-1.40738300	-9.64459300	1.83961900
C	-1.48073700	-7.18986400	0.55918600
H	-1.54359400	-9.02817100	-0.57739400
H	0.21944700	-7.57896600	-1.59465100
H	0.91243500	-9.11501000	-1.07545700
H	2.35288500	-7.19711900	-0.34686400
C	0.78487900	-6.11996400	0.68654200
H	2.26369500	-9.23953300	1.10006000
H	2.49369200	-7.79189900	2.07905700
C	-0.16402700	-7.32420500	2.69144300
H	0.72955800	-9.25662100	3.07989400
H	0.51798800	-6.80227200	3.37373100
C	-0.52016100	-6.40220100	1.48704200
H	-1.07554200	-7.54023200	3.26330200
H	-2.41011600	-7.42167100	1.09489600

H	-1.75378600	-6.57221000	-0.30642300
H	0.55257500	-5.47420900	-0.17054500
H	1.49580400	-5.56887500	1.31505700
C	6.77082300	1.51847300	-6.88286200
C	5.84487800	0.38246300	-6.40184900
C	5.00005200	-0.12918100	-7.58467000
C	4.14972100	1.02961600	-8.14090600
C	5.07542000	2.16038700	-8.63058000
C	5.91950400	2.67369100	-7.44816700
H	7.45684200	1.14418500	-7.65298400
H	7.39212600	1.87766600	-6.05173600
C	4.91121100	0.91519400	-5.29580200
H	6.44837800	-0.43862500	-5.99491500
H	4.35123500	-0.95279200	-7.25816400
H	5.65230700	-0.53004600	-8.37060800
H	3.53440700	0.66908100	-8.97435600
C	3.21990800	1.56585400	-7.03238000
H	5.72898200	1.79272500	-9.43153500
H	4.48104700	2.97972200	-9.05531700
C	4.98535700	3.21154100	-6.34519600
H	6.57595200	3.48504500	-7.78611000
H	4.38382400	4.04556600	-6.72895200
C	4.03671400	2.09111500	-5.82397400
H	5.58493500	3.61118200	-5.51860300
H	5.50924500	1.24899900	-4.43888200
H	4.25604400	0.11261700	-4.93151300
H	2.54275200	0.76497700	-6.70725100
H	2.59475200	2.37114500	-7.43952700
H	4.81871300	3.18487500	-3.44302400
C	-1.51242800	2.11019700	0.86314300
N	-0.29166500	1.22159800	0.99154500
C	-0.35143800	0.09010400	-0.02962900
H	0.57034800	1.77937200	0.84063800
C	1.21467900	-1.87960400	0.15714700
C	2.39543900	-2.53634800	-0.19217200
C	3.34326100	-1.89914900	-0.99345900
C	3.10728300	-0.60091600	-1.45103000
C	1.92059300	0.04826900	-1.11762100
C	0.96783600	-0.58182800	-0.30371000
H	0.47920800	-2.38589200	0.77464400
H	2.56863500	-3.54789400	0.15941600
H	4.26095400	-2.41274600	-1.26374500
H	3.83591200	-0.09760400	-2.07847300
H	1.72723400	1.04163300	-1.50970300
C	-3.32845700	3.98780200	3.61498700
C	-3.02817600	3.07834900	2.59920000
C	-1.74837500	3.04001500	2.02953500
C	-0.76532500	3.91937200	2.50721600
C	-1.06209800	4.81667800	3.53444200
C	-2.34510100	4.85904500	4.08556800
H	-4.32857900	4.01382700	4.03676400
H	-3.79267100	2.39598100	2.23787700
H	0.23190800	3.90975300	2.07689500
H	-0.29287900	5.49265800	3.89610000
H	-2.57625700	5.56828500	4.87433200
H	-0.73521900	0.54960300	-0.94181900
H	-1.10730100	-0.60482800	0.33714200
H	-2.36050300	1.43462100	0.74526800
H	-1.38506800	2.66017000	-0.07360200
H	-0.21877500	0.86077000	1.96019800

1 29 1.0 59 1.0
2 7 1.0 81 1.0
3 41 1.0 87 1.0
4 17 1.0 77 1.0
5 15 1.0 70 1.0
6 31 1.0 88 1.0
7 10 1.5 16 1.5
8 9 1.0 13 1.5 65 1.5
9
10 14 1.5 25 1.0
11 12 1.0 28 1.5 30 1.5
12
13 15 1.5 38 1.0
14 40 1.5 309 1.0
15 36 1.5
16 19 1.5 46 1.0
17 33 1.5 42 1.5
18 34 1.5 41 1.5 54 1.0
19 20 1.0 40 1.5
20
21 25 1.0 31 1.5 68 1.5
22 29 1.5 32 1.5 54 1.0

23 24 1.0 33 1.5 37 1.5
24
25 26 1.0 27 1.0
26
27
28 29 1.5 46 1.0
29
30 32 1.5 237 1.0
31 43 1.5
32 208 1.0
33 38 1.0
34 35 1.0 67 1.5
35
36 44 1.5 49 1.0
37 52 1.5 278 1.0
38 39 1.0 85 1.0
39
40 303 1.0
41 48 1.5
42 52 1.5 74 1.0
43 49 1.0 63 1.5
44 45 1.0 65 1.5
45
46 47 1.0 86 1.0
47
48 57 1.5 74 1.0
49 50 1.0 51 1.0
50
51
52 53 1.0
53
54 55 1.0 56 1.0
55
56
57 58 1.0 67 1.5
58
59 60 1.0 61 1.0 62 1.0
60
61 122 1.0 123 1.0 124 1.0
62
63 64 1.0 66 1.5
64
65 214 1.0
66 68 1.5 202 1.0
67 177 1.0
68 69 1.0
69
70 71 1.0 72 1.0 73 1.0
71
72 89 1.0 90 1.0 91 1.0
73
74 75 1.0 76 1.0
75
76
77 78 1.0 79 1.0 80 1.0
78
79 104 1.0 105 1.0 106 1.0
80
81 82 1.0 83 1.0 84 1.0
82
83 140 1.0 141 1.0 142 1.0
84
85
86
87
88
89 92 1.0 93 1.0 94 1.0
90
91
92 95 1.0 96 1.0 97 1.0
93
94
95 98 1.0 99 1.0 100 1.0
96
97
98 101 1.0 102 1.0 103 1.0
99
100
101
102
103
104 107 1.0 108 1.0 109 1.0
105

106
107 110 1.0 111 1.0 112 1.0
108
109
110 113 1.0 114 1.0 115 1.0
111
112
113 116 1.0 117 1.0 118 1.0
114
115
116 119 1.0 120 1.0 121 1.0
117
118
119
120
121
122 125 1.0 126 1.0 127 1.0
123
124
125 128 1.0 129 1.0 130 1.0
126
127
128 131 1.0 132 1.0 133 1.0
129
130
131 134 1.0 135 1.0 136 1.0
132
133
134 137 1.0 138 1.0 139 1.0
135
136
137
138
139
140
141
142 143 1.0 144 1.0 145 1.0
143 146 1.0 147 1.0 148 1.0
144
145
146 149 1.0 150 1.0 151 1.0
147
148
149 152 1.0 153 1.0 154 1.0
150
151
152 155 1.0 156 1.0 157 1.0
153
154
155
156
157
158 159 1.0 163 1.0 164 1.0 165 1.0
159 160 1.0 166 1.0 167 1.0
160 161 1.0 168 1.0 169 1.0
161 162 1.0 170 1.0 171 1.0
162 163 1.0 172 1.0 173 1.0
163 174 1.0 175 1.0
164
165
166 177 1.0 179 1.0 180 1.0
167
168
169
170
171 177 1.0 181 1.0 182 1.0
172
173
174 176 1.0 177 1.0 178 1.0
175
176
177
178
179
180
181
182
183 184 1.0 188 1.0 189 1.0 190 1.0
184 185 1.0 191 1.0 192 1.0
185 186 1.0 193 1.0 194 1.0
186 187 1.0 195 1.0 196 1.0
187 188 1.0 197 1.0 198 1.0
188 199 1.0 200 1.0

189
190
191 202 1.0 204 1.0 205 1.0
192
193
194
195
196 202 1.0 206 1.0 207 1.0
197
198
199 201 1.0 202 1.0 203 1.0
200
201
202
203
204
205
206
207
208
209 210 1.0 214 1.0 215 1.0 216 1.0
210 211 1.0 217 1.0 218 1.0
211 212 1.0 219 1.0 220 1.0
212 213 1.0 221 1.0 222 1.0
213 214 1.0 223 1.0 224 1.0
214 225 1.0
215
216
217 227 1.0 229 1.0 230 1.0
218
219
220
221
222 227 1.0 231 1.0 232 1.0
223
224
225 226 1.0 227 1.0 228 1.0
226
227 233 1.0
228
229
230
231
232
233
234 235 1.0 239 1.0 240 1.0 241 1.0
235 236 1.0 242 1.0 243 1.0
236 237 1.0 244 1.0 245 1.0
237 238 1.0 246 1.0
238 239 1.0 247 1.0 248 1.0
239 249 1.0 250 1.0
240
241
242 252 1.0 254 1.0 255 1.0
243
244
245
246 252 1.0 256 1.0 257 1.0
247
248
249 251 1.0 252 1.0 253 1.0
250
251
252 258 1.0
253
254
255
256
257
258
259 260 1.0 264 1.0 265 1.0 266 1.0
260 261 1.0 267 1.0 268 1.0
261 262 1.0 269 1.0 270 1.0
262 263 1.0 271 1.0 272 1.0
263 264 1.0 273 1.0 274 1.0
264 275 1.0 276 1.0
265
266
267 278 1.0 280 1.0 281 1.0
268
269
270
271

272 278 1.0 282 1.0 283 1.0
273
274
275 277 1.0 278 1.0 279 1.0
276
277
278
279
280
281
282
283
284 285 1.0 289 1.0 290 1.0 291 1.0
285 286 1.0 292 1.0 293 1.0
286 287 1.0 294 1.0 295 1.0
287 288 1.0 296 1.0 297 1.0
288 289 1.0 298 1.0 299 1.0
289 300 1.0 301 1.0
290
291
292 303 1.0 305 1.0 306 1.0
293
294
295
296
297 303 1.0 307 1.0 308 1.0
298
299
300 302 1.0 303 1.0 304 1.0
301
302
303
304
305
306
307
308
309
310 311 1.0 327 1.0 338 1.0 339 1.0
311 312 1.0 313 1.0 340 1.0
312 319 1.0 336 1.0 337 1.0
313
314 315 1.5 319 1.5 320 1.0
315 316 1.5 321 1.0
316 317 1.5 322 1.0
317 318 1.5 323 1.0
318 319 1.5 324 1.0
319
320
321
322
323
324
325 326 1.5 330 1.5 331 1.0
326 327 1.5 332 1.0
327 328 1.5
328 329 1.5 333 1.0
329 330 1.5 334 1.0
330 335 1.0
331
332
333
334
335
336
337
338
339
340

Energy = -6070.07984 a.u.
0 imaginary frequency

Atomic coordinates of $3^{+}\subset$1g

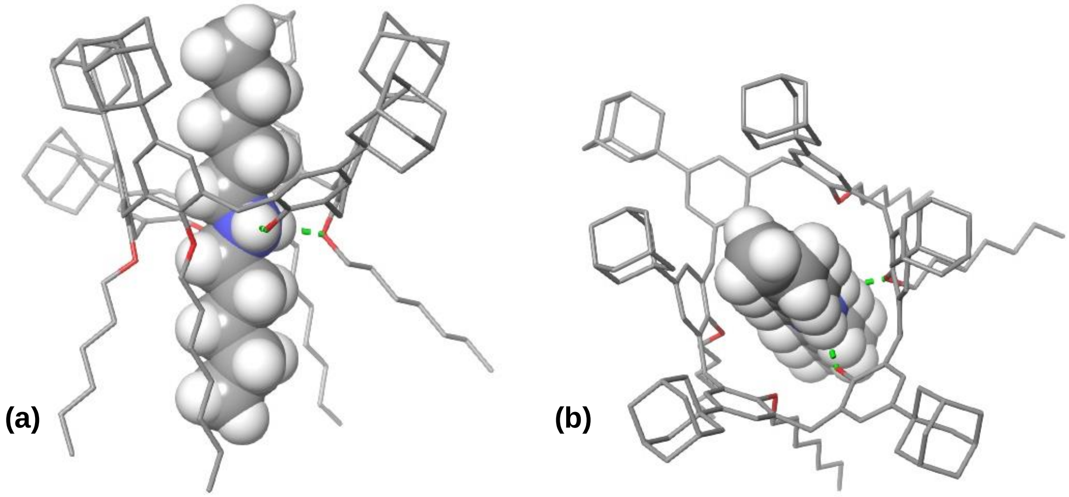

Figure S69. (a) Side view and **(b)** top view of complex **3⁺⊂1g.**

O	-1.08033400	-3.49913700	1.12055800
O	2.55843800	-3.07442700	2.37696500
O	-2.65013300	-1.36732500	-2.11363200
O	-1.46851600	-0.10852500	-4.24865800
O	2.44932600	0.02846700	-3.95335900
O	3.52759200	-1.35961900	0.27467100
C	2.34972100	-1.99610700	3.24478400
C	1.55127700	3.34458100	-2.62956500
H	0.82810700	4.12865700	-2.82132200
C	3.41910800	-1.11154300	3.46195600
C	-2.24040300	-1.70866600	4.12179600
H	-1.88665900	-1.51788300	5.12731200
C	1.49443700	2.18504000	-3.40982500
C	3.18969100	0.02627400	4.23482700
C	2.45581000	1.18408000	-3.17656300
C	1.08591900	-1.78028300	3.81675400
C	-1.89740900	1.22375900	-4.12713500
C	-4.49076900	-1.69838300	-0.61947900
C	0.91154400	-0.61997300	4.58435300
H	-0.05981900	-0.44445100	5.02563500
C	5.27449500	-0.52866700	1.75203900
C	-3.16147100	-2.32255600	1.54790200
C	-1.47015300	3.57995600	-4.11400700
H	-0.77741400	4.40201600	-4.26272300
C	4.81366600	-1.39386200	2.91405800
H	5.52901400	-1.25984600	3.73132300
H	4.88779900	-2.44735500	2.62702600
C	-1.39466200	-2.41496400	3.25352400
C	-1.89480900	-2.76073300	1.98743200
C	-3.51731400	-1.28098400	3.74674900
C	4.63188600	-0.56977700	0.50438000
C	-3.93467800	-1.57691300	2.43958300
C	-0.97431300	2.27814100	-4.23844800
C	-5.82780400	-1.44925700	-0.29393700
H	-6.24922500	-1.99028600	0.54797300
C	3.40601900	1.31027700	-2.14854600
C	-2.81690400	3.86628700	-3.83849000
C	0.49366400	2.03169100	-4.55505500
H	0.61314100	1.02638400	-4.95393100
C	1.94036600	0.30366500	4.80783600
C	-3.95711700	-1.07009700	-1.76053800
C	-3.25668200	1.45710400	-3.88589700
C	5.11143200	0.20515700	-0.56621500
C	3.41595800	2.48966400	-1.39645300
H	4.15968500	2.58210300	-0.61649500
C	-0.00513500	-2.84716800	3.69773300
H	0.35126700	-3.60559100	3.00684800
C	-4.75304500	-0.22277100	-2.54793400
C	4.42941000	0.19769600	-1.91733400
H	3.94437300	-0.76519800	-2.09067500
H	5.20817700	0.28222000	-2.68715800
C	-3.68790600	2.77912800	-3.72209100
H	-4.74214500	2.94469300	-3.53172900
C	-3.68955700	-2.72240700	0.16624800
H	-2.85332600	-3.05579500	-0.44778300

H	-4.33376000	-3.60250400	0.29889500
C	-6.07294900	0.01978200	-2.14810500
H	-6.66962400	0.67164900	-2.77595600
C	-1.43816900	-4.89576100	0.94144700
H	-2.52215500	-4.97552100	0.81215600
C	-0.98000700	-5.80882200	2.07309400
H	-0.96763200	-5.18769800	-0.00211900
C	6.25118800	0.98782100	-0.36403000
H	6.62003200	1.56022700	-1.21106800
C	2.52012800	3.53944400	-1.63544800
C	6.92709200	1.05682700	0.86219700
C	-6.64670300	-0.57564000	-1.02169700
C	6.40381900	0.28534800	1.90436800
H	6.88664100	0.28975300	2.87533700
C	3.28699100	0.09275600	-5.12488600
H	2.94065500	0.90535100	-5.77880400
C	3.22122000	-1.24537400	-5.84407900
H	4.31846400	0.32490500	-4.82948500
C	-4.27903500	0.32649800	-3.88717400
H	-5.16507500	0.68919100	-4.41587900
H	-3.90297700	-0.50611500	-4.49045100
C	-1.47060600	-0.62690400	-5.60568700
H	-2.44081400	-0.41345500	-6.07052900
C	-1.19863100	-2.12138200	-5.56242900
H	-0.70304300	-0.10120400	-6.18516900
C	3.01672300	-4.30222300	2.99086700
H	2.22057000	-4.69843000	3.63373600
C	3.37371300	-5.28856900	1.89013900
H	3.88354100	-4.09180500	3.62981500
H	0.78135000	2.72755700	-5.35406600
H	-0.10075600	-3.32702300	4.68116300
H	-2.38122300	-0.94301700	-2.95955600
H	3.38785700	-2.00881700	0.98925500
C	4.08144500	-1.27970600	-7.11397700
H	3.54392700	-2.03302800	-5.15188900
H	2.17423800	-1.45854100	-6.09813700
C	4.00997300	-2.62155900	-7.85341000
H	3.76646100	-0.47496900	-7.79280500
H	5.12697700	-1.06540700	-6.85305800
C	4.87025900	-2.66827900	-9.12211900
H	4.32198300	-3.42757800	-7.17387400
H	2.96389300	-2.83512700	-8.11727400
C	4.79052300	-4.01017600	-9.85551900
H	4.55962100	-1.86115000	-9.79911300
H	5.91489500	-2.45656600	-8.85759900
H	5.12926800	-4.83303000	-9.21597900
H	3.76306800	-4.23336600	-10.16476400
H	5.41438800	-4.01078000	-10.75448900
C	-1.19868400	-2.75517900	-6.96063800
H	-0.22963600	-2.28975600	-5.07644300
H	-1.95875100	-2.60513300	-4.93466900
C	-0.94362200	-4.26753000	-6.93608000
H	-2.16164000	-2.55862800	-7.45151200
H	-0.43486700	-2.26827700	-7.58272000
C	-0.93418300	-4.90785500	-8.32932800
H	0.01756500	-4.46470400	-6.43997800
H	-1.71142300	-4.75358500	-6.31757900
C	-0.68374100	-6.42077200	-8.30514700
H	-1.89292500	-4.70722600	-8.82796100
H	-0.16465800	-4.42297700	-8.94684700
C	-0.67334300	-7.05293900	-9.69992500
H	0.27380900	-6.62043500	-7.80487800
H	-1.45426500	-6.90431200	-7.68936200
H	-0.49293700	-8.13107300	-9.64806900
H	-1.63019500	-6.90060400	-10.21146200
H	0.11013300	-6.61494600	-10.32837900
C	-1.39916800	-7.26603100	1.82760300
H	0.11102400	-5.75058300	2.16946100
H	-1.40699400	-5.45856100	3.02045300
C	-0.93649800	-8.22137200	2.93514700
H	-2.49279000	-7.32060100	1.73607700
H	-0.99589900	-7.60737600	0.86363600
C	-1.35593000	-9.67729000	2.69952900
H	0.15810000	-8.16862300	3.02486200
H	-1.33673500	-7.87846100	3.89945400
C	-0.88924900	-10.63458300	3.80325000
H	-2.45046800	-9.73030100	2.61311100
H	-0.95935100	-10.01838700	1.73252800
C	-1.31286400	-12.08621000	3.56154900
H	0.20471000	-10.58132000	3.88893700
H	-1.28554500	-10.29298500	4.76900400
H	-0.96485000	-12.74243500	4.36519200
H	-2.40343400	-12.17732700	3.50737700

H	-0.90273700	-12.46796500	2.61981600
H	2.49674700	-5.43014400	1.24505600
H	4.15774000	-4.84985100	1.25932900
C	3.84310100	-6.64114800	2.44227100
C	4.22155600	-7.64263200	1.34377400
H	4.70662800	-6.48627700	3.10360100
H	3.05262300	-7.07395700	3.07147300
C	4.68772000	-8.99776800	1.88909900
H	3.35914400	-7.79505200	0.67904600
H	5.01450700	-7.21010100	0.71766900
C	5.06939700	-10.00039100	0.79304200
H	3.89442200	-9.43021900	2.51552000
H	5.54857000	-8.84435500	2.55508800
C	5.53418600	-11.35072100	1.34597500
H	4.20883300	-10.15332000	0.12738700
H	5.86245600	-9.56767100	0.16824500
H	5.79890200	-12.04228900	0.54015500
H	6.41535300	-11.23470200	1.98674300
H	4.74989100	-11.82499600	1.94656200
C	-11.05062100	-0.49785400	-1.11994200
C	-10.34845100	-1.49137400	-0.17414500
C	-10.36385400	-0.93178600	1.26302800
C	-9.62722400	0.42289400	1.29702600
C	-10.32901700	1.41536500	0.34992200
C	-10.30604800	0.85122500	-1.08411600
H	-12.09613100	-0.36017300	-0.81675000
H	-11.06535100	-0.89537000	-2.14311300
C	-8.88944500	-1.69344600	-0.63162400
H	-10.86798600	-2.45733200	-0.19976800
H	-9.88338600	-1.64091700	1.95021900
H	-11.39798000	-0.80702000	1.60792500
H	-9.63265300	0.82065100	2.31993800
C	-8.16712700	0.22505300	0.84163200
H	-11.36416800	1.58101600	0.67389800
H	-9.82499100	2.39021500	0.38213700
C	-8.84533100	0.64597400	-1.53647000
H	-10.79186400	1.55915800	-1.76694400
H	-8.32375400	1.61220100	-1.54130900
C	-8.10512200	-0.34793000	-0.60480700
H	-8.83510700	0.26940800	-2.56754600
H	-8.86268000	-2.10716100	-1.64791100
H	-8.39881700	-2.42764100	0.01895800
H	-7.65583500	-0.45541900	1.53411500
H	-7.62419800	1.17869900	0.87714200
C	10.17635600	3.78656200	-0.21513700
C	9.11342700	4.29533100	0.78004600
C	9.67542300	4.22644100	2.21323100
C	10.03707000	2.76599800	2.54734800
C	11.10608000	2.26063800	1.55857000
C	10.54489300	2.32799800	0.12490000
H	11.06997200	4.42186700	-0.16955500
H	9.79344900	3.85011400	-1.24237800
C	7.85233800	3.41288700	0.68031100
H	8.84592100	5.33142000	0.53669300
H	8.93445000	4.60661300	2.92880500
H	10.56288600	4.86552600	2.30383400
H	10.42493800	2.70702900	3.57194600
C	8.77645000	1.88297400	2.43989800
H	12.01416800	2.87139900	1.63966500
H	11.39233100	1.22973400	1.80458700
C	9.28622500	1.44260300	0.02356300
H	11.29779700	1.96261500	-0.58472700
H	9.53669700	0.39754100	0.24706300
C	8.18155400	1.92677200	1.00906000
H	8.90418600	1.46041900	-1.00438100
H	7.43071000	3.48794200	-0.32953500
H	7.07984400	3.77361100	1.37230100
H	8.02706600	2.22474000	3.16611000
H	9.03349900	0.84939400	2.70573400
H	-4.90240900	-1.22445900	2.10478300
C	2.92001400	6.01586500	-1.92555000
C	2.98788800	7.39098200	-1.23110000
C	4.10333800	7.37495800	-0.16798000
C	3.79587900	6.28253800	0.87521400
C	3.72316700	4.90816000	0.17778600
C	2.60808300	4.88472800	-0.89932800
H	3.87054700	5.79574900	-2.42821300
H	2.14963200	6.03348400	-2.70615700
C	1.63566600	7.69140700	-0.55316200
H	3.20360800	8.16096600	-1.98238100
H	4.17561400	8.35485600	0.32072700
H	5.07456500	7.18279600	-0.64210200
H	4.59441800	6.25591000	1.62719900

C	2.44944600	6.58901700	1.55895000
H	4.69367400	4.68517700	-0.28262300
H	3.53730100	4.12549900	0.92494400
C	1.26511400	5.22522200	-0.19342200
H	1.03931300	4.44600000	0.54423600
C	1.33328000	6.60295000	0.49666700
H	0.44340500	5.21943900	-0.91999100
H	0.83513000	7.72477300	-1.30451200
H	1.66373700	8.67897700	-0.07552000
H	2.49709400	7.55776900	2.07236900
H	2.23277800	5.83193100	2.32475200
H	0.36831600	6.81061900	0.97647700
C	-7.12224500	0.72135400	5.21897700
C	-5.84567900	1.57216800	5.06114300
C	-4.85435600	0.84608300	4.12855300
C	-4.46430600	-0.54618500	4.70579200
C	-5.76805700	-1.37789600	4.89893000
C	-6.75739800	-0.64719900	5.82916300
H	-7.60724000	0.58342000	4.24337300
H	-7.84405200	1.24038300	5.86210900
C	-5.19358400	1.78147200	6.44161300
H	-6.10203600	2.54422800	4.62143100
H	-3.94774500	1.45041600	3.99754600
H	-5.30137200	0.73057500	3.13393700
C	-3.84219500	-0.31836400	6.10754900
H	-6.24215400	-1.56026700	3.92693000
H	-5.51201100	-2.36172000	5.31302300
C	-6.10494400	-0.43717000	7.20917000
H	-7.66291200	-1.25723800	5.93672100
H	-5.86012300	-1.40597400	7.66366300
C	-4.82855400	0.41065100	7.04361200
H	-6.80610800	0.06473000	7.88765700
H	-5.88132400	2.31598900	7.10887600
H	-4.29454500	2.40451200	6.34509500
H	-2.92155700	0.27330700	6.01672500
H	-3.56341700	-1.28163400	6.55418400
H	-4.35002400	0.55173100	8.02060500
C	-4.94662400	7.64136200	-4.62253900
C	-5.22573400	6.92097000	-3.28880700
C	-4.43384800	7.60443000	-2.15702000
C	-2.92757600	7.52408400	-2.47148600
C	-2.64070300	8.23928900	-3.80763300
C	-3.44050900	7.56055300	-4.93830500
H	-5.26486100	8.68949500	-4.55929600
H	-5.52816500	7.18009500	-5.43130000
C	-4.79667000	5.44327400	-3.40168900
H	-6.29889800	6.96136000	-3.06497000
H	-4.64684400	7.11633300	-1.19686400
H	-4.74488100	8.65195500	-2.05783300
H	-2.35468900	8.00157700	-1.66651200
C	-2.50390000	6.04513000	-2.57949200
H	-2.91656000	9.29871000	-3.73441800
H	-1.56616300	8.20601500	-4.03155000
C	-3.01701300	6.08178300	-5.05178600
H	-3.23406700	8.06453200	-5.89057600
H	-1.95330900	6.02280200	-5.31217000
C	-3.28359500	5.32288200	-3.71766300
H	-3.56701600	5.58950100	-5.86409600
H	-5.38288500	4.95571500	-4.19133400
H	-5.02475300	4.92206900	-2.46263400
H	-2.68647700	5.52607000	-1.62924700
H	-1.42507400	5.98642300	-2.76888800
C	2.95270600	3.99123900	6.93428700
C	1.98753400	4.09953500	5.73590500
C	0.54099800	4.22931300	6.25056300
C	0.18577400	2.98436500	7.08642500
C	1.14368100	2.88117000	8.28963300
C	2.59099700	2.75119500	7.77696700
H	2.89113600	4.89712900	7.55037100
H	3.98918100	3.91807400	6.57953900
C	2.11036100	2.83528900	4.86085000
H	2.24628100	4.97872900	5.13201600
H	-0.15410400	4.32997900	5.40644200
H	0.43499400	5.13573400	6.85985700
H	-0.84867900	3.06249000	7.44392400
C	0.31656800	1.71850700	6.21356100
H	1.04519700	3.76865400	8.92732700
H	0.88226300	2.01322100	8.90886200
C	2.71613100	1.48456600	6.90631800
H	3.27905200	2.66927200	8.62746700
H	2.47789900	0.59085800	7.49733900
C	1.76148300	1.55579800	5.67671200
H	3.75298900	1.37004600	6.56864500

H	3.12875300	2.76131100	4.46171800
H	1.43848800	2.90576400	3.99486000
H	-0.38940000	1.77646000	5.37405000
H	0.03778000	0.83912000	6.80863900
H	4.02163600	0.70574900	4.39597900
C	-1.64699400	3.99182500	2.23232900
H	-0.70091300	4.18984300	2.74868600
H	-2.45750600	4.33723600	2.88191200
C	-1.79992800	2.50595300	1.89746400
H	-1.66287000	4.60729000	1.32612300
H	-2.76779700	2.34027900	1.40613100
C	-0.68187000	1.96170700	0.99898500
H	-1.82813400	1.91915900	2.82495100
C	-0.90100000	0.48854000	0.63142900
H	-0.62084300	2.56128400	0.08177100
H	0.28537900	2.07400000	1.50737700
C	0.20905900	-0.05398100	-0.26354500
H	-0.96613600	-0.11135700	1.54552700
H	-1.86473400	0.38875600	0.11660900
N	-0.10855300	-1.44586700	-0.75420300
H	0.33822000	0.56189200	-1.15742900
H	1.17187800	-0.11362200	0.24912800
C	0.90943800	-1.99286000	-1.72216700
C	0.63413400	-3.45624600	-2.05660100
H	0.87934800	-1.35882800	-2.61093100
H	1.88503600	-1.86992700	-1.24755000
C	1.68968700	-4.02988300	-3.01207900
H	0.63312700	-4.04393200	-1.12995500
H	-0.36545900	-3.55669700	-2.49992700
C	1.44578200	-5.50257200	-3.36456700
H	1.71292800	-3.43278500	-3.93297600
H	2.68326400	-3.92346600	-2.55597500
C	2.51024200	-6.07743000	-4.30337400
H	1.41432200	-6.09540000	-2.44024600
H	0.45478900	-5.60482000	-3.82667500
H	2.30866400	-7.12693500	-4.53768600
H	2.54423500	-5.52644700	-5.25007900
H	3.50772800	-6.02269500	-3.85359100
H	-0.20811100	-2.10493800	0.03531600
H	-1.04430800	-1.42881200	-1.20426700

1 29 1.0 59 1.0
2 7 1.0 81 1.0
3 41 1.0 87 1.0
4 17 1.0 77 1.0
5 15 1.0 70 1.0
6 31 1.0 88 1.0
7 10 1.5 16 1.5
8 9 1.0 13 1.5 65 1.5
9
10 14 1.5 25 1.0
11 12 1.0 28 1.5 30 1.5
12
13 15 1.5 38 1.0
14 40 1.5 309 1.0
15 36 1.5
16 19 1.5 46 1.0
17 33 1.5 42 1.5
18 34 1.5 41 1.5 54 1.0
19 20 1.0 40 1.5
20
21 25 1.0 31 1.5 68 1.5
22 29 1.5 32 1.5 54 1.0
23 24 1.0 33 1.5 37 1.5
24
25 26 1.0 27 1.0
26
27
28 29 1.5 46 1.0
29
30 32 1.5 237 1.0
31 43 1.5
32 208 1.0
33 38 1.0
34 35 1.0 67 1.5
35
36 44 1.5 49 1.0
37 52 1.5 278 1.0
38 39 1.0 85 1.0
39
40 303 1.0
41 48 1.5
42 52 1.5 74 1.0

43 49 1.0 63 1.5
44 45 1.0 65 1.5
45
46 47 1.0 86 1.0
47
48 57 1.5 74 1.0
49 50 1.0 51 1.0
50
51
52 53 1.0
53
54 55 1.0 56 1.0
55
56
57 58 1.0 67 1.5
58
59 60 1.0 61 1.0 62 1.0
60
61 122 1.0 123 1.0 124 1.0
62
63 64 1.0 66 1.5
64
65 214 1.0
66 68 1.5 202 1.0
67 177 1.0
68 69 1.0
69
70 71 1.0 72 1.0 73 1.0
71
72 89 1.0 90 1.0 91 1.0
73
74 75 1.0 76 1.0
75
76
77 78 1.0 79 1.0 80 1.0
78
79 104 1.0 105 1.0 106 1.0
80
81 82 1.0 83 1.0 84 1.0
82
83 140 1.0 141 1.0 142 1.0
84
85
86
87
88
89 92 1.0 93 1.0 94 1.0
90
91
92 95 1.0 96 1.0 97 1.0
93
94
95 98 1.0 99 1.0 100 1.0
96
97
98 101 1.0 102 1.0 103 1.0
99
100
101
102
103
104 107 1.0 108 1.0 109 1.0
105
106
107 110 1.0 111 1.0 112 1.0
108
109
110 113 1.0 114 1.0 115 1.0
111
112
113 116 1.0 117 1.0 118 1.0
114
115
116 119 1.0 120 1.0 121 1.0
117
118
119
120
121
122 125 1.0 126 1.0 127 1.0
123
124
125 128 1.0 129 1.0 130 1.0

126
127
128 131 1.0 132 1.0 133 1.0
129
130
131 134 1.0 135 1.0 136 1.0
132
133
134 137 1.0 138 1.0 139 1.0
135
136
137
138
139
140
141
142 143 1.0 144 1.0 145 1.0
143 146 1.0 147 1.0 148 1.0
144
145
146 149 1.0 150 1.0 151 1.0
147
148
149 152 1.0 153 1.0 154 1.0
150
151
152 155 1.0 156 1.0 157 1.0
153
154
155
156
157
158 159 1.0 163 1.0 164 1.0 165 1.0
159 160 1.0 166 1.0 167 1.0
160 161 1.0 168 1.0 169 1.0
161 162 1.0 170 1.0 171 1.0
162 163 1.0 172 1.0 173 1.0
163 174 1.0 175 1.0
164
165
166 177 1.0 179 1.0 180 1.0
167
168
169
170
171 177 1.0 181 1.0 182 1.0
172
173
174 176 1.0 177 1.0 178 1.0
175
176
177
178
179
180
181
182
183 184 1.0 188 1.0 189 1.0 190 1.0
184 185 1.0 191 1.0 192 1.0
185 186 1.0 193 1.0 194 1.0
186 187 1.0 195 1.0 196 1.0
187 188 1.0 197 1.0 198 1.0
188 199 1.0 200 1.0
189
190
191 202 1.0 204 1.0 205 1.0
192
193
194
195
196 202 1.0 206 1.0 207 1.0
197
198
199 201 1.0 202 1.0 203 1.0
200
201
202
203
204
205
206
207
208

209 210 1.0 214 1.0 215 1.0 216 1.0
210 211 1.0 217 1.0 218 1.0
211 212 1.0 219 1.0 220 1.0
212 213 1.0 221 1.0 222 1.0
213 214 1.0 223 1.0 224 1.0
214 225 1.0
215
216
217 227 1.0 229 1.0 230 1.0
218
219
220
221
222 227 1.0 231 1.0 232 1.0
223
224
225 226 1.0 227 1.0 228 1.0
226
227 233 1.0
228
229
230
231
232
233
234 235 1.0 239 1.0 240 1.0 241 1.0
235 236 1.0 242 1.0 243 1.0
236 237 1.0 244 1.0 245 1.0
237 238 1.0 246 1.0
238 239 1.0 247 1.0 248 1.0
239 249 1.0 250 1.0
240
241
242 252 1.0 254 1.0 255 1.0
243
244
245
246 252 1.0 256 1.0 257 1.0
247
248
249 251 1.0 252 1.0 253 1.0
250
251
252 258 1.0
253
254
255
256
257
258
259 260 1.0 264 1.0 265 1.0 266 1.0
260 261 1.0 267 1.0 268 1.0
261 262 1.0 269 1.0 270 1.0
262 263 1.0 271 1.0 272 1.0
263 264 1.0 273 1.0 274 1.0
264 275 1.0 276 1.0
265
266
267 278 1.0 280 1.0 281 1.0
268
269
270
271
272 278 1.0 282 1.0 283 1.0
273
274
275 277 1.0 278 1.0 279 1.0
276
277
278
279
280
281
282
283
284 285 1.0 289 1.0 290 1.0 291 1.0
285 286 1.0 292 1.0 293 1.0
286 287 1.0 294 1.0 295 1.0
287 288 1.0 296 1.0 297 1.0
288 289 1.0 298 1.0 299 1.0
289 300 1.0 301 1.0
290
291

292 303 1.0 305 1.0 306 1.0
293
294
295
296
297 303 1.0 307 1.0 308 1.0
298
299
300 302 1.0 303 1.0 304 1.0
301
302
303
304
305
306
307
308
309
310 311 1.0 312 1.0 313 1.0 314 1.0
311
312
313 315 1.0 316 1.0 317 1.0
314
315
316 318 1.0 319 1.0 320 1.0
317
318 321 1.0 322 1.0 323 1.0
319
320
321 324 1.0 325 1.0 326 1.0
322
323
324 327 1.0 343 1.0 344 1.0
325
326
327 328 1.0 329 1.0 330 1.0
328 331 1.0 332 1.0 333 1.0
329
330
331 334 1.0 335 1.0 336 1.0
332
333
334 337 1.0 338 1.0 339 1.0
335
336
337 340 1.0 341 1.0 342 1.0
338
339
340
341
342
343
344

Energy = -5922.50258 a.u.
0 imaginary frequency

Atomic coordinates of $2^{+}\subset 1a$

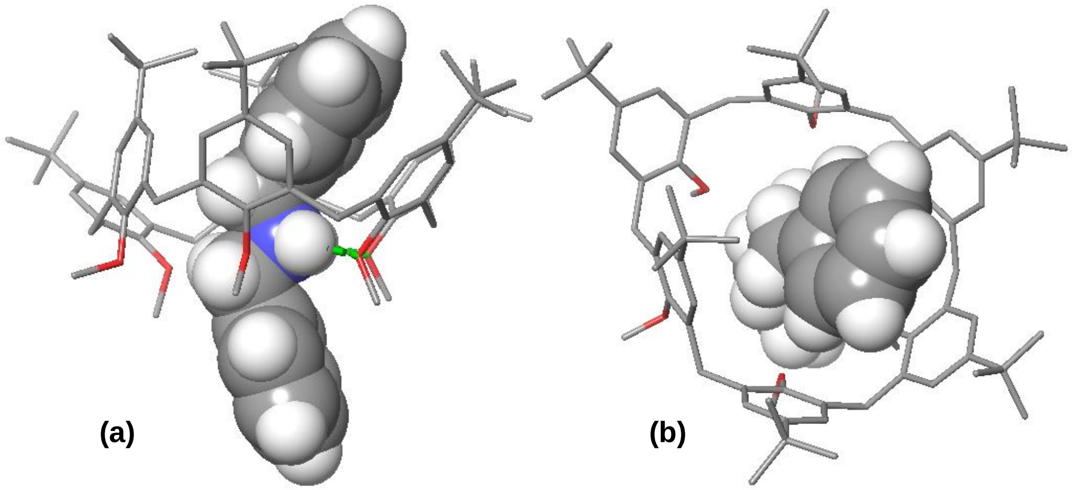

(a) (b)

Figure S70. (a) Side view and **(b)** top view of complex **$2^{+}\subset 1a$.**

O	2.10354300	-4.25711300	2.20165200
O	-1.81854600	-3.24451700	2.40789200
O	3.90857900	-0.42692100	2.12990900
O	2.90749100	3.39136700	2.35844800
O	-0.88652500	2.93951600	2.27744800
O	-3.08435800	-0.00604400	2.59311500
C	-2.39402300	-3.72098000	1.22834300
C	-1.13915000	5.30341500	-0.53504700
H	-0.49925200	6.01861200	-1.03956400
C	-3.68707800	-3.31365800	0.86757300
C	0.85023500	-3.92905200	-1.24081400
H	-0.04707600	-4.16368800	-1.80261800
C	-0.58531600	4.56581500	0.52022400
C	-4.24086400	-3.83333300	-0.31228100
H	-5.24643300	-3.52963200	-0.57475400
C	-1.42103500	3.67660100	1.21272000
C	-1.65720800	-4.60850700	0.41986700
C	2.94847000	3.34766800	0.96651900
C	5.17805500	-1.80803300	0.61064300
C	-2.26589800	-5.10828900	-0.73363200
H	-1.70940200	-5.82561700	-1.32997600
C	-4.78929200	-0.98510300	1.18589400
C	3.14541600	-3.32609900	0.23458100
C	2.00965900	3.94577900	-1.16765000
H	1.24217500	4.46355500	-1.73412000
C	-4.50373400	-2.37409500	1.75064400
H	-5.46786800	-2.85514800	1.95346700
H	-3.98898200	-2.27092000	2.70281100
C	0.88986600	-4.27291900	0.11320000
C	2.05452700	-3.96649900	0.84314800
C	1.92531200	-3.30587500	-1.89264800
C	-4.09061000	0.15105700	1.63021000

C	3.05622200	-3.00664900	-1.12565600
H	3.90329300	-2.51191300	-1.58316200
C	1.93869800	3.99274000	0.22712000
C	6.20210300	-1.90588900	-0.34233700
H	6.44780800	-2.89423300	-0.71500500
C	-2.77875100	3.52287800	0.87719500
C	3.03706800	3.27567800	-1.84946700
C	0.85796700	4.80016200	0.94170600
H	0.96266900	4.61025900	2.00815300
C	-3.56116300	-4.74009200	-1.12952900
C	4.86737700	-0.54344500	1.12450400
C	3.98121500	2.64499100	0.32637900
C	-4.38139600	1.43592000	1.14689200
C	-3.27281400	4.28229000	-0.18700600
H	-4.32566300	4.19649400	-0.43202000
C	-0.24900800	-5.03563800	0.79011500
H	-0.10246100	-4.95245900	1.86597500
C	5.51046000	0.61203800	0.65304700
C	-3.70724300	2.67020500	1.74095800
H	-3.14447000	2.35108300	2.61309900
H	-4.50601100	3.33093100	2.10199100
C	3.99710500	2.61787700	-1.07367200
H	4.79484700	2.06824500	-1.55673600
C	-4.17904800	-5.34825300	-2.40260700
C	6.52798100	0.46100000	-0.29331300
H	7.04605300	1.35538700	-0.62855600
C	2.65973000	-5.53595200	2.52230200
H	2.64239100	-5.61938400	3.61093500
H	2.06841200	-6.35071000	2.08734600
C	-5.40416000	1.55991800	0.19685100
H	-5.65577900	2.55313200	-0.15173600
C	-2.47979000	5.17954500	-0.91637800
C	-6.13370200	0.46442900	-0.27340000
C	6.90924300	-0.79002600	-0.80293700
C	-5.80529100	-0.79621000	0.24641400
H	-6.37946600	-1.66252300	-0.06656700
C	-1.00879700	3.59835800	3.55064700
H	-0.56244800	2.93837000	4.29327500
H	-2.05989700	3.77950300	3.79859700
C	1.84389700	-3.01244300	-3.40360500
C	-7.28777700	0.60170400	-1.28512600
C	-3.11050600	6.02513600	-2.04023200
C	0.62453500	-2.11170100	-3.70579500
H	-0.31628300	-2.57377700	-3.39427900
H	0.55676600	-1.91690700	-4.78169900
H	0.70580600	-1.14864400	-3.19192000
C	3.64911700	4.47181400	2.93512100
H	4.71322500	4.40845900	2.68058100
H	3.26297600	5.44251000	2.60219500
C	1.69094800	-4.34946300	-4.16853000
H	2.54300500	-5.01032000	-3.97943300
H	1.63541100	-4.16724900	-5.24729500
H	0.78221600	-4.88344900	-3.87463400

C	-3.74637700	5.09845200	-3.10275100
H	-4.22099700	5.69479100	-3.88910200
H	-4.51285100	4.44632700	-2.67383400
H	-2.99039600	4.46185400	-3.57416300
C	-2.07689100	-4.08829100	3.53794000
H	-3.14507900	-4.10769000	3.78459300
H	-1.74090300	-5.11495100	3.35587700
C	-5.59784900	-4.81544600	-2.67733900
H	-6.29074400	-5.05840100	-1.86517300
H	-5.99157500	-5.27235400	-3.59014700
H	-5.60567300	-3.73004400	-2.82479500
C	-3.29152200	-5.00571700	-3.62274500
H	-3.21070100	-3.92209400	-3.75954500
H	-3.72099500	-5.43071100	-4.53613000
H	-2.27925800	-5.40744400	-3.51783700
C	3.10540900	-2.30195000	-3.92984000
H	2.99868600	-2.11041400	-5.00197800
H	4.00530600	-2.91024400	-3.79337000
H	3.26589300	-1.33777700	-3.43567400
C	3.10856900	3.32992200	-3.38822700
C	-4.26330900	-6.88555200	-2.24630700
H	-3.27803900	-7.33532800	-2.09275300
H	-4.69633000	-7.33523200	-3.14612200
H	-4.89305000	-7.15955400	-1.39385200
C	-4.20756500	6.93462800	-1.43576800
H	-3.78785800	7.60598200	-0.67987800
H	-5.00395500	6.35328000	-0.96167000
H	-4.66555400	7.54907000	-2.21822200
C	-2.07809000	6.92365900	-2.74716300
H	-1.63084700	7.65169600	-2.06287600
H	-2.56778200	7.48697400	-3.54712700
H	-1.27159300	6.33941200	-3.20295400
C	-8.60370700	0.11546900	-0.63193500
H	-8.84615600	0.71204300	0.25328300
H	-8.54450100	-0.93234200	-0.32307200
H	-9.43477700	0.20569000	-1.33948000
C	-7.49125300	2.05652400	-1.74859200
H	-7.75633700	2.71798600	-0.91741100
H	-8.30958100	2.10022300	-2.47348900
H	-6.59714200	2.45761100	-2.23783400
C	4.23856200	2.44850800	-3.95315300
H	4.23794500	2.50620000	-5.04589200
H	4.11238300	1.39638600	-3.67672300
H	5.22498400	2.77633900	-3.61051500
C	1.77545500	2.85151900	-4.00666700
H	0.93485500	3.48382900	-3.70596700
H	1.54524800	1.82345500	-3.71003300
H	1.83346900	2.88414000	-5.09984400
C	-6.98731700	-0.26119600	-2.53346600
H	-7.80298400	-0.17690600	-3.25934500
H	-6.87861400	-1.32050500	-2.28187600
H	-6.06402300	0.06600500	-3.02288000
C	3.37208700	4.79258300	-3.82178400

H	2.57698700	5.46396800	-3.48320400
H	3.42734100	4.86330700	-4.91357800
H	4.31740900	5.15996700	-3.40981700
H	1.07443000	5.86518200	0.78834000
C	8.06278400	-0.89003900	-1.81864600
C	7.72440600	-0.04996900	-3.07289400
C	9.35987600	-0.34635000	-1.17317100
C	8.32140000	-2.34017300	-2.26886100
H	9.14688800	-2.36027900	-2.98676200
H	8.60115600	-2.98514300	-1.42963000
H	7.44700700	-2.77753400	-2.76217400
H	8.53838000	-0.11279400	-3.80300900
H	6.80981500	-0.41246300	-3.55373900
H	7.58028200	1.00718900	-2.83055100
H	10.19152000	-0.40564600	-1.88344400
H	9.25725700	0.69959800	-0.86928300
H	9.63006400	-0.92767600	-0.28577300
H	-0.14275300	-6.09929600	0.53878800
H	-1.51780800	-3.67586400	4.38027700
H	3.69413100	-5.62414600	2.17062800
H	3.53311900	4.38942100	4.01795000
H	-0.47995800	4.55684100	3.54756100
C	-3.55363200	0.08116600	3.94867900
H	-2.67715400	0.00230300	4.59273700
H	-4.05497000	1.03791300	4.12784100
H	-4.25354800	-0.72984000	4.17605400
C	4.47199600	-0.55729500	3.44338400
H	5.20901700	0.23097400	3.63668400
H	3.64938500	-0.46540700	4.15375000
H	4.95896900	-1.53043700	3.57227800
C	5.09686200	1.98774700	1.13865700
H	5.97709300	2.64299500	1.11108100
H	4.77104900	1.93730500	2.17733400
C	4.41108200	-3.04143800	1.04156300
H	5.08023700	-3.90791000	0.95731200
H	4.12597100	-2.97322100	2.09221900
C	0.65364100	-0.66735800	2.78357500
N	-0.27596000	0.01398700	1.79107100
C	0.09918500	-0.39220100	0.37016800
H	-1.26099700	-0.23170200	2.00469200
C	-0.66426100	1.29994000	-1.33742600
C	-1.51521100	1.69504600	-2.36905400
C	-2.56257400	0.86536400	-2.77087400
C	-2.75884700	-0.36215800	-2.13707100
C	-1.90996400	-0.75843700	-1.10491500
C	-0.85883700	0.07163000	-0.69349200
H	0.16151900	1.94160700	-1.04619500
H	-1.35334100	2.64763100	-2.86127000
H	-3.21724700	1.17113400	-3.58163000
H	-3.56766500	-1.01610200	-2.44573900
H	-2.05436700	-1.72382000	-0.63223600
C	1.30738900	1.53644900	5.82056800
C	1.31935400	1.05154700	4.51095400

C	0.58641000	-0.09853200	4.17823200
C	-0.14627600	-0.75108900	5.17841700
C	-0.15900700	-0.26395000	6.48637100
C	0.56870900	0.88245700	6.80919600
H	1.88439300	2.42157700	6.07130300
H	1.90320900	1.56671000	3.75113800
H	-0.69565000	-1.65463400	4.93069900
H	-0.72377800	-0.78516100	7.25329800
H	0.56951000	1.25773300	7.82790400
H	0.17003200	-1.48148100	0.38675300
H	1.10139800	0.01162200	0.21102800
H	1.66148900	-0.56569300	2.37310000
H	0.37600700	-1.72303300	2.76506300
H	-0.24507800	1.04311800	1.90015000

1 30 1.0 59 1.0
2 7 1.0 90 1.0
3 43 1.0 165 1.0
4 18 1.0 79 1.0
5 16 1.0 69 1.0
6 32 1.0 161 1.0
7 10 1.5 17 1.5
8 9 1.0 13 1.5 64 1.5
9
10 14 1.5 26 1.0
11 12 1.0 29 1.5 31 1.5
12
13 16 1.5 40 1.0
14 15 1.0 42 1.5
15
16 38 1.5
17 20 1.5 48 1.0
18 35 1.5 44 1.5
19 36 1.5 43 1.5 172 1.0
20 21 1.0 42 1.5
21
22 26 1.0 32 1.5 67 1.5
23 30 1.5 33 1.5 172 1.0
24 25 1.0 35 1.5 39 1.5
25
26 27 1.0 28 1.0
27
28
29 30 1.5 48 1.0
30
31 33 1.5 72 1.0
32 45 1.5
33 34 1.0
34
35 40 1.0
36 37 1.0 66 1.5
37
38 46 1.5 51 1.0

39 54 1.5 105 1.0
40 41 1.0 142 1.0
41
42 56 1.0
43 50 1.5
44 54 1.5 169 1.0
45 51 1.0 62 1.5
46 47 1.0 64 1.5
47
48 49 1.0 156 1.0
49
50 57 1.5 169 1.0
51 52 1.0 53 1.0
52
53
54 55 1.0
55
56 93 1.0 97 1.0 106 1.0
57 58 1.0 66 1.5
58
59 60 1.0 61 1.0 158 1.0
60
61
62 63 1.0 65 1.5
63
64 74 1.0
65 67 1.5 73 1.0
66 143 1.0
67 68 1.0
68
69 70 1.0 71 1.0 160 1.0
70
71
72 75 1.0 82 1.0 101 1.0
73 118 1.0 122 1.0 134 1.0
74 86 1.0 110 1.0 114 1.0
75 76 1.0 77 1.0 78 1.0
76
77
78
79 80 1.0 81 1.0 159 1.0
80
81
82 83 1.0 84 1.0 85 1.0
83
84
85
86 87 1.0 88 1.0 89 1.0
87
88
89
90 91 1.0 92 1.0 157 1.0
91

92
93 94 1.0 95 1.0 96 1.0
94
95
96
97 98 1.0 99 1.0 100 1.0
98
99
100
101 102 1.0 103 1.0 104 1.0
102
103
104
105 126 1.0 130 1.0 138 1.0
106 107 1.0 108 1.0 109 1.0
107
108
109
110 111 1.0 112 1.0 113 1.0
111
112
113
114 115 1.0 116 1.0 117 1.0
115
116
117
118 119 1.0 120 1.0 121 1.0
119
120
121
122 123 1.0 124 1.0 125 1.0
123
124
125
126 127 1.0 128 1.0 129 1.0
127
128
129
130 131 1.0 132 1.0 133 1.0
131
132
133
134 135 1.0 136 1.0 137 1.0
135
136
137
138 139 1.0 140 1.0 141 1.0
139
140
141
142
143 144 1.0 145 1.0 146 1.0
144 150 1.0 151 1.0 152 1.0

145 153 1.0 154 1.0 155 1.0
146 147 1.0 148 1.0 149 1.0
147
148
149
150
151
152
153
154
155
156
157
158
159
160
161 162 1.0 163 1.0 164 1.0
162
163
164
165 166 1.0 167 1.0 168 1.0
166
167
168
169 170 1.0 171 1.0
170
171
172 173 1.0 174 1.0
173
174
175 176 1.0 192 1.0 203 1.0 204 1.0
176 177 1.0 178 1.0 205 1.0
177 184 1.0 201 1.0 202 1.0
178
179 180 1.5 184 1.5 185 1.0
180 181 1.5 186 1.0
181 182 1.5 187 1.0
182 183 1.5 188 1.0
183 184 1.5 189 1.0
184
185
186
187
188
189
190 191 1.5 195 1.5 196 1.0
191 192 1.5 197 1.0
192 193 1.5
193 194 1.5 198 1.0
194 195 1.5 199 1.0
195 200 1.0
196
197

198
199
200
201
202
203
204
205

Energy = -3850.69576 a.u.
0 imaginary frequency

Crystallographic information

Table S6. Dihedral angles, θ, between the mean plane of the bridging methylene groups and the mean planes of the six aryl rings for **1f** and **1g.** Absolute angle value greater/smaller than 90° indicates outward/inward orientation of the adamantly group, while a negative sign indicates an inverted orientation of the adamantly group with respect to a given orientation of the macrocycle.

Crystal	Dihedral angles, θ [°]					
**1f**[a]	1	2	3	1’	2’	3’
	96	132	72	-96	-132	-72
**1g**	1	2	3	4	5	6
	-46	94	98	158	96	98

[a] 1’, 2’, and 3’ are generated by inversion of 1, 2, and 3, respectively. See the main text and Figure 1 for full details on the designation of the six aryl rings.

Table S7. Crystal data and structure refinement for **1f** and **1g**.

	**1f**	**1g**
**Empirical formula**	$C_{108}H_{136}O_6$, 4($CHCl_3$)	$C_{126}H_{168}O_6$, 2.25($CHCl_3$)
**Formula weight**	2003.6	2047.17
**Temperature (K)**	100(2)	100(2)
**Wavelength (Å)**	0.7	0.7
**Crystal system**	Triclinic	Triclinic
**Space group**	P -1	P -1
**Unit cell dimensions (Å, °)**	*a* = 12.338 (2)<br>*b* = 15.813 (3)<br>*c* = 15.8860 (6)<br>*α* = 62.331(11)<br>*β* = 80.881 (10)<br>*γ* = 68.02 (3)	*a* = 12.959 (4)<br>*b* = 15.165 (3)<br>*c* = 29.378 (7)<br>*α* = 75.80 (3)<br>*β* = 86.69 (2)<br>*γ* = 86.896 (10)
**Volume (Å^3)**	2544.9 (9)	5583 (3)
**Z**	1	2
ρ_{calcd} **(g/cm^3)**	1.307	1.218
μ **(mm^{-1})**	0.361	0.216
**F(000)**	1060	2205
**Reflections collected**	48310	45006
**Independent reflections**	13952	12925
**Data / restraints / parameters**	13952 / 0 / 590	12925 / 1496 / 1417
**GooF**	1.038	1.042
**R1 / wR2 [I>2σ(I)]**	0.042 / 0.0436	0.1007/ 0.1598
**R1 / wR2 all data**	0.1192 / 0.1208	0.2737 / 0.3254
**Largest. Diff. peak/hole (e Å^{-3})**	0.798 / -1.035	0.666 / -0.413
**CCDC code**	1988954	1988955

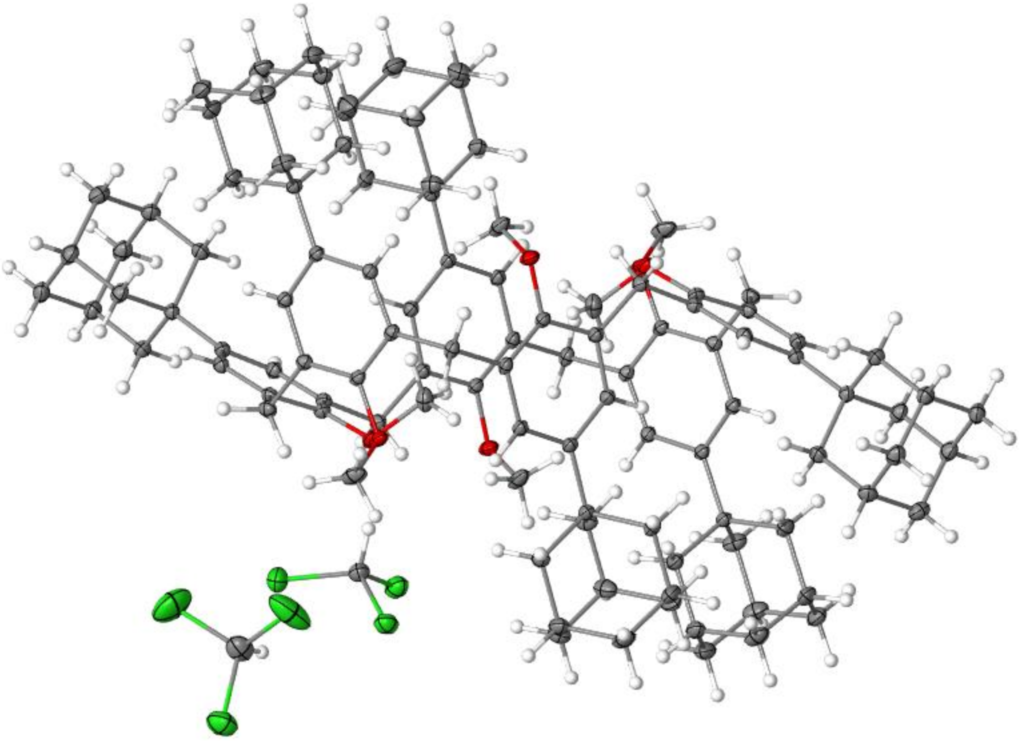

a)

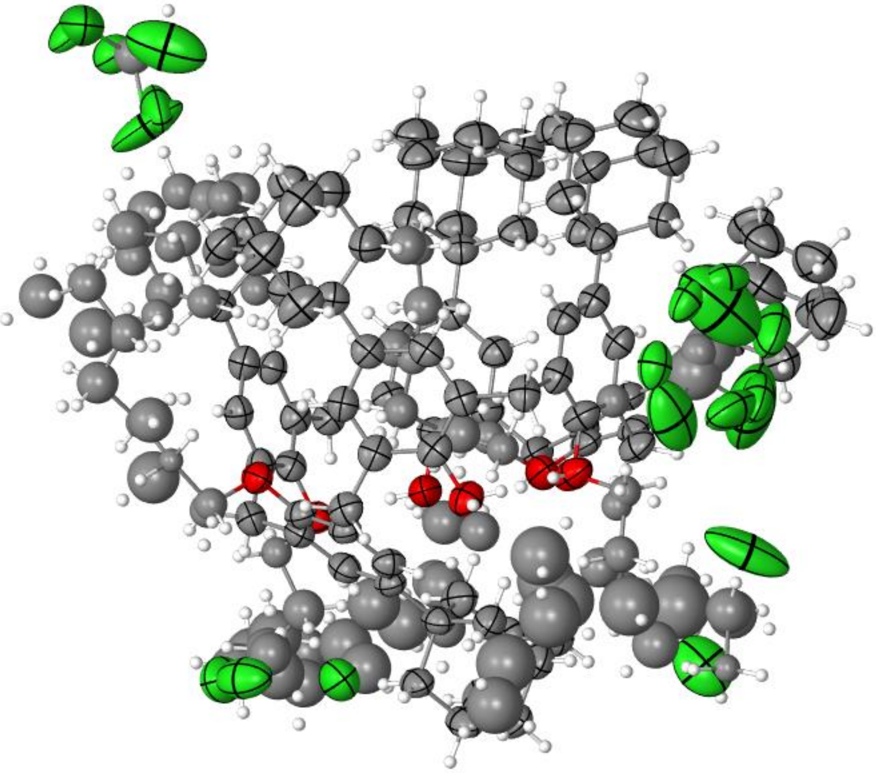

b)

Figure S71. ORTEP drawing of **1f** (a) and **1g** (b) (ellipsoids at 50% probability).

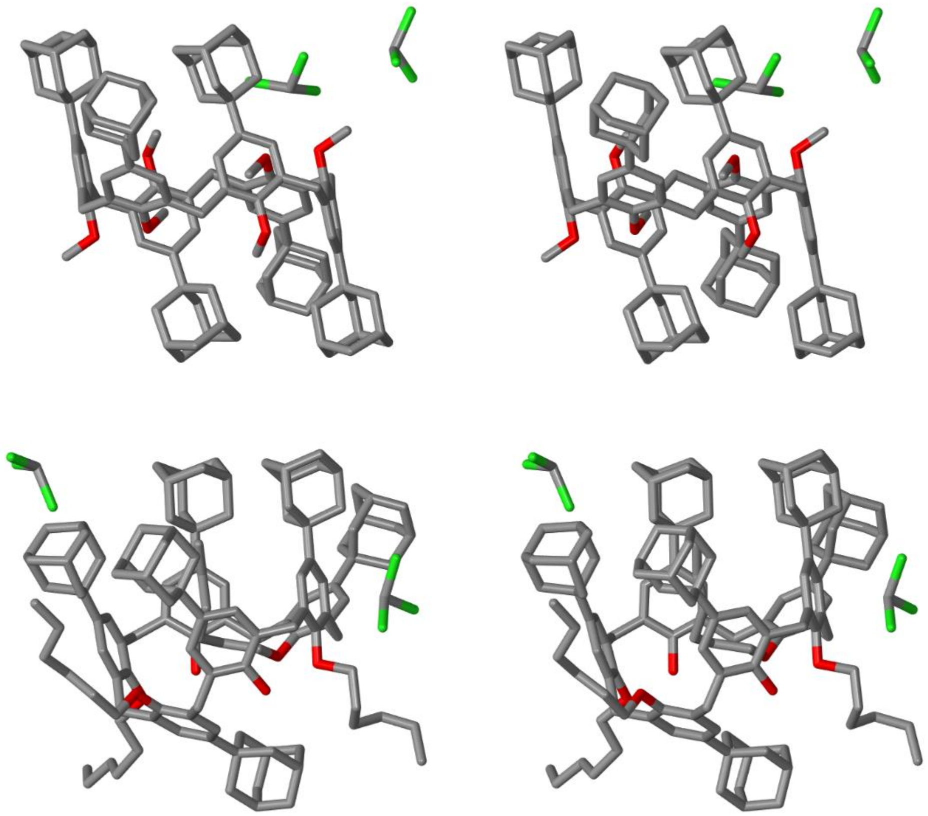

a)

b)

Figure S72. Stereo view of **1f** (a) and **1g** (b).
